# New *Mycobacterium tuberculosis* Beijing clonal complexes in China revealed by phylogenetic and Bayesian population structure analyses of 24-loci MIRU-VNTRs

**DOI:** 10.1038/s41598-017-06346-1

**Published:** 2017-07-20

**Authors:** Chao Zheng, Yann Reynaud, Changsong Zhao, Thierry Zozio, Song Li, Dongxia Luo, Qun Sun, Nalin Rastogi

**Affiliations:** 1 Key Laboratory of Bio-resources and Eco-environment of the Ministry of Education, College of Life Sciences, Sichuan University, Chengdu, Sichuan 610065 PR China; 2 WHO Supranational TB Reference Laboratory, Tuberculosis and Mycobacteria Unit, Institut Pasteur de la Guadeloupe, Morne Jolivière, 97183 Abymes, Guadeloupe France; 3 Public Health Clinical Center of Chengdu, Chengdu, Sichuan 610000 PR China

## Abstract

Beijing lineage of *Mycobacterium tuberculosis* constitutes the most predominant lineage in East Asia. Beijing epidemiology, evolutionary history, genetics are studied in details for years revealing probable origin from China followed by worldwide expansion, partially linked to higher mutation rate, hypervirulence, drug-resistance, and association with cases of mixed infections. Considering huge amount of data available for 24-loci Mycobacterial Interspersed Repetitive Units-Variable Number of Tandem Repeats, we performed detailed phylogenetic and Bayesian population structure analyses of Beijing lineage strains in mainland China and Taiwan using available 24-loci MIRU-VNTR data extracted from publications or the SITVIT2 database (n = 1490). Results on genetic structuration were compared to previously published data. A total of three new Beijing clonal complexes tentatively named BSP1, BPS2 and BSP3 were revealed with surprising phylogeographical specificities to previously unstudied regions in Sichuan, Chongqing and Taiwan, proving the need for continued investigations with extended datasets. Such geographical restriction could correspond to local adaptation of these “ecological specialist” Beijing isolates to local human host populations in contrast with “generalist pathogens” able to adapt to several human populations and to spread worldwide.

## Introduction

Tuberculosis (TB) is one of the main public health problems in the world and its morbidity and mortality rank first among infectious diseases. According to the World Health Organization (WHO), TB caused an estimated 10.4 million new (incident) cases in 2015, including 480,000 cases of multidrug-resistant TB (MDR-TB), and 1.8 million deaths^1^. Beijing lineage of *Mycobacterium tuberculosis* complex (MTBC) which belongs to the lineage 2 (East-Asian) as defined by Regions of Differences-Large Sequence Polymorphisms (RD-LSPs), constitutes the most predominant lineage in East Asia^2–4^. Partially attributed to its properties of hypervirulence, multi drug-resistance (MDR), and association with cases of mixed infections^5–8^, it has today spread worldwide^9–11^, leading to much effort to monitor its epidemiology within a broadened concept of its evolutionary genetics. In this context, we decided to perform a detailed mapping of available genotyping data on Beijing lineage strains in mainland China and Taiwan by means of phylogenetic and Bayesian population structure analyses to delineate tentative Beijing clonal complexes with distinct genetic and phylogeographical characteristics. In view of the huge amount of data available for Mycobacterial Interspersed Repetitive Units-Variable Number of Tandem Repeats (MIRU-VNTRs)^11–13^, we decided to exclusively focus on 24-loci format considered a robust classical genotyping marker for *M*. *tuberculosis* Beijing lineage epidemiology, phylogeny, and clonal heterogeneity^14–17^. Although, limited homoplasy due to convergent evolution events as regards to 24-loci MIRU-VNTR typing was underlined in an earlier study^18^, a recent study focusing on whole genome sequencing (WGS) based phylogeographical structure of Beijing isolates (n = 4987 strains from 99 countries, including 615 strains from China), showed congruent results for clusterization obtained by 24-loci MIRU-VNTRs and WGS^19^, defining a total six clonal complexes (CC1 to CC6) and a basal sublineage (BL7). The authors showed that CC1-CC5 comprised typical/modern Beijing strains as opposed to CC6 and BL7 which comprised atypical/ancestral Beijing variants, an observation further corroborated by a deeper branching of CC6 and BL7 in the genome-based trees^19^. Subsequently, this approach was considered relevant for further studying phylogenetic sublineages of Beijing strains in a limited collection from China (n = 302 clinical isolates)^20^.

Nonetheless, considering the relatively smaller numbers of Chinese Beijing strains in the above studies (615 and 302 isolates, respectively), we decided to evaluate these findings on an enlarged 24-loci MIRU-VNTR dataset (n = 1490 isolates from mainland China and Taiwan; data recovered from the SITVIT2 database and/or from published literature). Following phylogenetic and Bayesian population structure analyses performed on this dataset led to the characterization of tentatively three new Beijing clonal complexes with phylogeographical specificities to previously unstudied geographical regions, proving the need for continued investigations with extended datasets worldwide. Lastly, as a spin-off of this larger study, we also studied the phenomenon of clonal heterogeneity (CH) defined as “inpatient” microevolution of an infecting clone, to explore if any given clonal complex could be more prone to variability (and subsequent geographical adaptability) among the involved isolates^21, 22^. Interestingly, CH cases exclusively mapped with ubiquitous Beijing lineages, suggesting higher adaptability.

## Results

### Population structure of MTB Beijing lineage

A total of 16090*M*. *tuberculosis* isolates were collected from almost every province (excluding Macao) in mainland China as well as from Taiwan. Spoligotype profiles were available for 12674 isolates, and among these 9676 (76.35%) strains belonged to the Beijing lineage based on their lineage specific signatures^10, 11, 23^. When focusing on geographical distribution (Supplementary Figure S1), we found that Beijing lineage strains were largely predominant in each region, representing more than three quarter of all isolates in the north, east, west and center of China and a lesser proportion in southern provinces. Conversely, non-Beijing strains accounted for a quarter or more of all isolates in southern provinces (Chongqing, Guizhou, Guangxi, Hong Kong) and Taiwan, as well as south-west (Sichuan).

**Figure 1:**
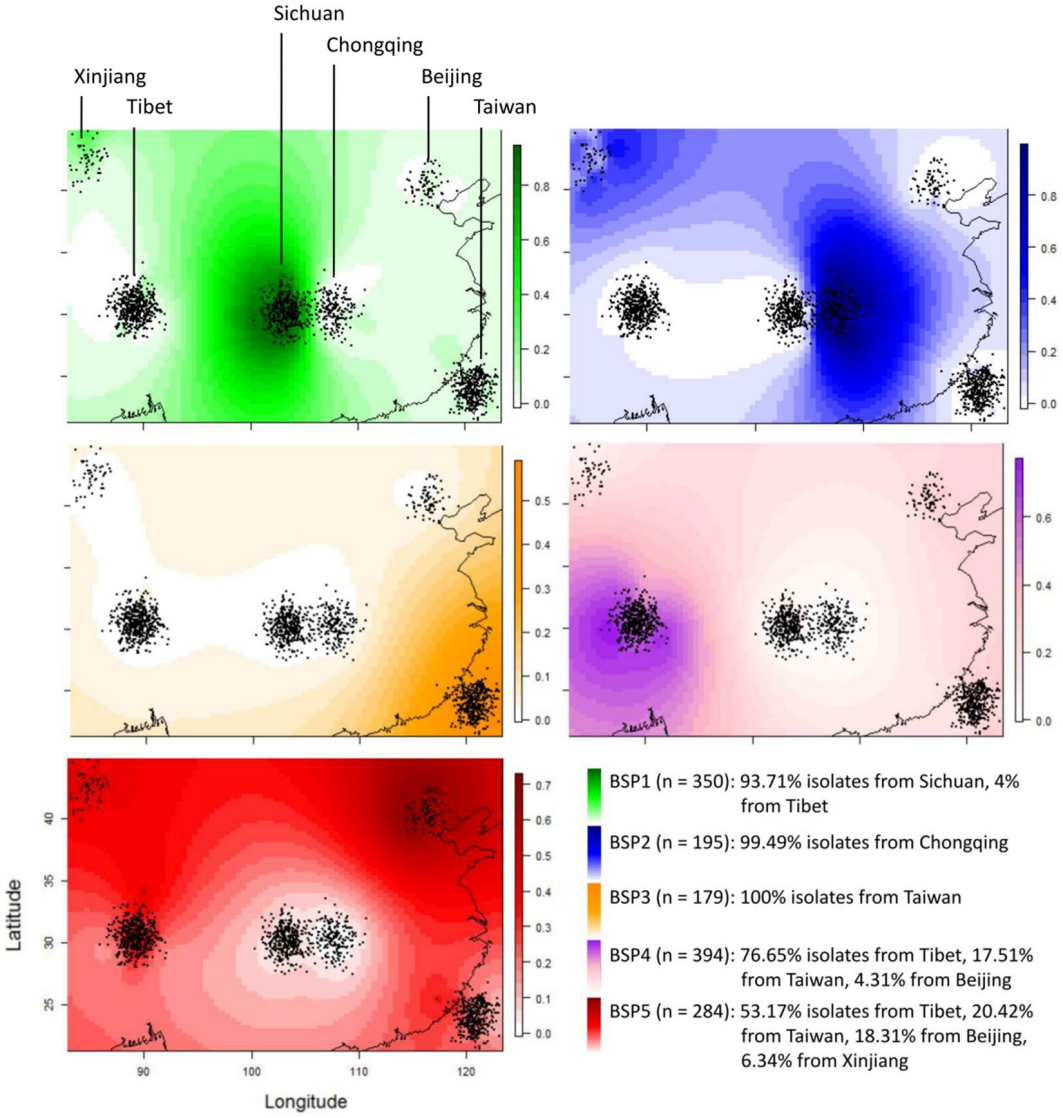
Bayesian population structure analyses based on 24 loci MIRU-VNTRs on 1490 *M*. *tuberculosis* Beijing isolates from mainland China and Taiwan. The figure shows STRUCTURE ancestry coefficient (Q-matrix) displayed spatially by universal kriging on separate maps for each K (subpopulation) showing the presence of 5 clonal complexes named BSP1 to BSP5. Briefly, BSP1 and BSP2 were predominant in Sichuan and Chongqing, BSP3 was exclusively found in Taiwan, while BSP4 and BSP5 were present in Tibet, Taiwan, Beijing and Xinjiang; black dots represent spatial coordinates of individuals.

In the next step, we performed the phylogenetic and Bayesian population structure analyses of 24-loci MIRU-VNTR data of 1490 Beijing isolates recovered from 6 regions, including Tibet n = 484; Sichuan n = 348; Taiwan n = 338; Chongqing n = 199; Beijing n = 72; and Xinjiang n = 49 (Supplementary Table S2). As illustrated in Fig. 1, the STRUCTURE ancestry coefficient (Q-matrix) effectively divided the Beijing population into 5 groups named Beijing subpopulations 1 to 5 (BSP1 to BSP5). In this figure, the geographical distribution patterns of each of these clonal complexes can be visualized spatially by universal kriging on separate maps. Briefly, BSP1 and BSP2 isolates were predominant in Sichuan and Chongqing representing 94.25% (328/348) and 97.49% (194/199) of isolates respectively, while BSP3 isolates were exclusively found in Taiwan where they accounted for 52.96% (179/338) of all isolates. As opposed to these region-specific Beijing clonal complexes, BSP4 and BSP5 were broadly distributed being present in Tibet, Taiwan, Beijing and Xinjiang. Thus BSP4 and BSP5 represented 62.40% (302/484) and 31.20% (151/484) of isolates in Tibet, 20.41% (69/338) and 17.16% (58/338) of isolates in Taiwan, 23.61% (17/72) and 72.22% (52/72) of isolates in Beijing, and 12.24% (6/49) and 36.73% (18/49) of isolates in Xinjiang. Note that BSPint which represents strains in intermediate position among various BSPs defined, comprised 5.9% (88/1490) of all isolates.

### Mean allelic richness of 24-loci MIRU-VNTRs

**Figure 2:**
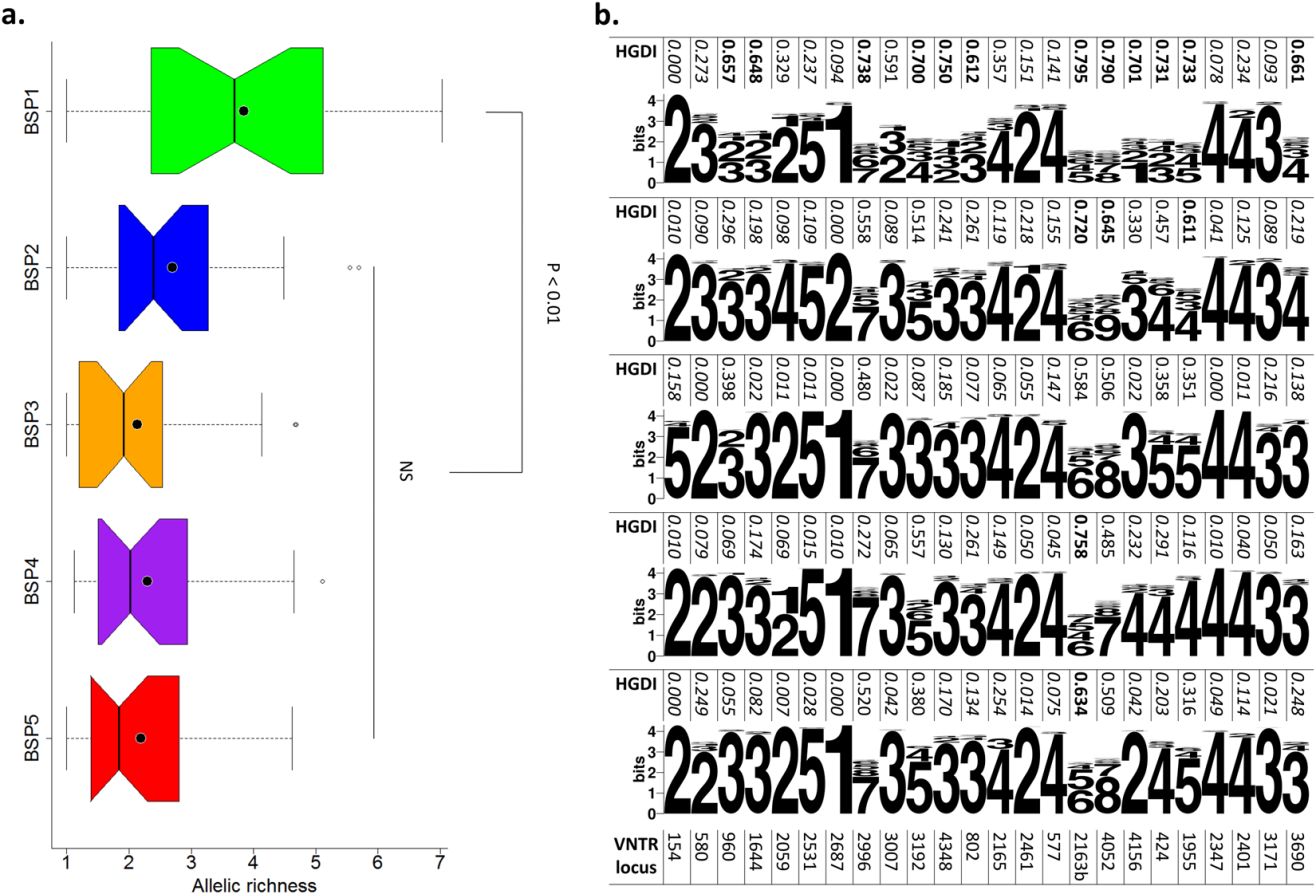
The mean allelic richness of 24-loci MIRU-VNTRs and WebLogo representation to visualize main patterns observed for BSPs. (**a**) Allelic richness of different BSPs. Notched boxes correspond to median values ± quartiles of allelic richness, while the dots and bars within notched boxes correspond to the mean and median values. Dotted lines adjacent to the boxes show the minimum/maximum values. Note that comparison tests as well as P values were estimated based on mean values by t-test. (**b**) WebLogo of allele copy number of 24-loci MIRU-VNTRs loci in each BSP grouping. The allelic diversity of the loci was classified as highly discriminant (HGDI > 0.6, bold in the table), moderately discriminant (0.3 ≤ HGDI ≤ 0.6, normal) and poorly discriminant (HGDI < 0.3, italic).

As illustrated in Fig. 2a, the mean allelic richness of 24-loci MIRU-VNTR loci were calculated for various BSP groupings after correcting for sample size effects; mean values corresponded to 3.85 for BSP1, 2.7 for BSP2, 2.13 for BSP3, 2.3 for BSP4, and 2.19 for BSP5. Thus BSP1 was characterized by a significantly higher allelic richness than that observed for BSP2 to BSP5 (P < 0.01, t-test), suggesting that it was the oldest clonal complex among the five. Interestingly, the mean allelic richness observed for BSP1 was even significantly higher than that observed for the most ancient group CC6 identified recently (mean value of 3.85 vs. 2.6, P < 0.01) by Merker *et al*.^19^. Considering that the time to the most recent common ancestor (TMRCA) was calculated as 6,161 years for CC6, one can presume that BSP1 corresponds to even an older group than the CC6^19^. Note further that the newly-found BSP2 also presented an almost similar allelic richness to CC6 (mean value of 2.7 vs. 2.6). Since the three newly described BSP groupings were phylogeographically restricted to precise geographic locations (BSP1 to Sichuan, BSP2 to Chongqing, and BSP3 to Taiwan), we also compared the mean allelic richness observed for various BSP groupings in our study vs. a similar analysis made on the global Beijing data (n = 4987 strains from 99 countries, including 615 strains from China) of Merker *et al*.^19^; interested readers may refer to Supplementary Figure S2a for a detailed comparison. Briefly, the highest mean allelic richness (mean value, 2.68) was seen in Eastern Asia (which included 615 strains from China). Nonetheless the values observed in Eastern Asia were not statistically different than those observed in Africa, North America, Pacific and Southern Asia, most likely as the strain collection from Eastern Asia was devoid of the BSP1 strains identified for the first time in the present study. Considering significantly higher mean allelic richness for BSP1 in our study than values obtained in each of the geographic regions in Merker’s dataset (P < 0.01, Supplementary Figure S2a), it seems crucial to extend future studies to cover all Chinese provinces to fully comprehend the evolution of Beijing strains in China.

### Main patterns of tandem repeats (WebLogos) and allelic diversity

Based on its allelic diversity, each loci was classified as highly discriminatory (HGDI > 0.6, shown in **bold** font), moderately discriminatory (0.3 ≤ HGDI ≤ 0.6, shown as normal font), or poorly discriminatory (HGDI < 0.3, shown in *italic* font). As illustrated in Supplementary Fig. S3, it allowed to define a total of 6 highly discriminatory loci (loci 4052, 424, 1955, 3192, 2163b, 4156), 8 moderately discriminatory loci (loci 3690, 802, 4348, 2996, 2059, 1644, 960, 580), and 10 poorly discriminatory loci (loci 2401, 2461, 2687, 2531, 3007, 3171, 2165, 154, 577 and 2347) in the Chinese dataset. Next, we drew WebLogos to visualize main patterns of tandem repeats for 24-loci MIRU-VNTRs in each BSP grouping in our dataset (Fig. 2b), as well as on Merker’s global Beijing data in function of different geographic regions worldwide (Supplementary Figure S2b). When the two datasets (Chinese vs. global) were compared, one could notice that: (i) among the 6 highly discriminatory loci in China, locus 4052 was also highly discriminatory in North America, 2163b in Africa, Eastern Asia, North America and Southern Asia, and 4156 only in Eastern Asia. The three remaining loci (424, 1955, and 3192) showed variable HGDI for the geographic regions in the global study but none was highly discriminatory; (ii) among the loci with moderately discriminatory power in China, 3/8 loci were poorly discriminatory in each geographic region in the global sample (580, 1644, and 2059), while the remaining 5 loci showed variable discriminatory power; (iii) among the 10 poorly discriminatory loci in China, 7 loci (2461, 2687, 3007, 3171, 154, 577, and 2347), were also poorly discriminatory worldwide, while 3 others were moderately discriminatory (2401 in South America, 2531 in South and North America, and 2165 in North America).

We further analyzed the genetic characteristics of five BSP groupings described in this investigation (Fig. 2, Supplementary Figures S2b and S3). Briefly, the BSP1 isolates showed significantly greater allelic diversity than other BSPs, noticeably linked to higher HGDI for loci 424, 4348, 802, 960, 3690, 1644, 3007 and 4156 (as can also be seen through a wider range of WebLogos and accompanying highest mean allelic richness). Furthermore, the BSP1 isolates were associated with 3 or less repeats of VNTR 424 vs. 4 or more repeats for other groups (P < 0.01). For BSP2 and BSP3, a total of 3 loci showed a potential to identify specific groups: (i) VNTR 2687 resulted exclusively in 2 repeats in BSP2 isolates vs. a single copy for all other groups, (ii) VNTR 2059 showed contrasted number of repeats between BSP2 (4 repeats in 94.87% isolates, n = 185/195) vs. all other groups where 2 repeats predominated, (iii) VNTR 154 showed predominantly 5 repeats for BSP3 isolates vs. 2 copies for other groups. Lastly, for BSP4 and BSP5, 87.31% (n = 344/394) of BSP4 isolates and 97.87% (n = 278/284) of BSP5 isolates presented the 4 and 2 repeats, respectively, in VNTR 4156.

### Minimum Spanning Tree (MST) based analysis on evolutionary relationships

**Figure 3:**
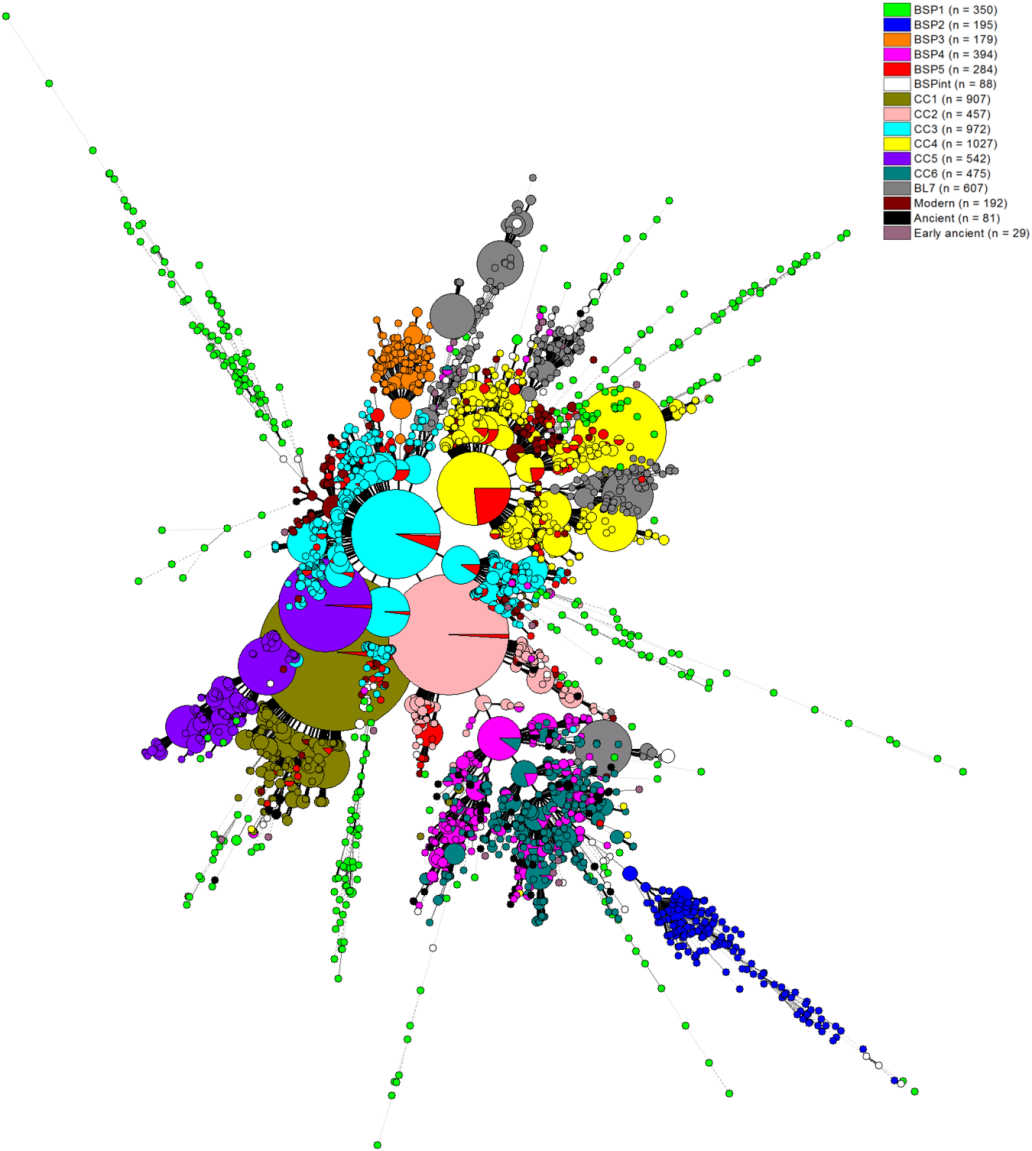
A minimum spanning tree (MST) based on pooled data on Beijing isolates (n = 6779 strains). The combined MST highlights evolutionary relationships of different Beijing groups from the present study on BSP groupings (BSP1 n = 350, BSP2 n = 195, BSP3 n = 179, BSP4 n = 394, BSP5 n = 284, BSPint n = 88; total 1490 strains), classification of a series of clonal complexes (CCs) defined by Merker *et al*.^19^ in a global study (CC1 n = 907, CC2 n = 457, CC3 n = 972, CC4 n = 1027, CC5 n = 542, CC6 n = 475, BL7 n = 607; total n = 4987 strains), and a recent study describing 3 groups based on evolutionary history of Beijing isolates in China countrywide by Yin *et al*.^20^, as Modern n = 192, Ancient n = 81, Early ancient n = 29; total n = 302 strains); the complexity of the lines denotes the number of allele/spacer changes between two patterns: solid lines (1 or 2 or 3 changes), gray dashed lines (4 changes) and gray dotted lines (5 or more changes); the size of the circle is proportional to the total number of isolates sharing same pattern.

**Figure 4:**
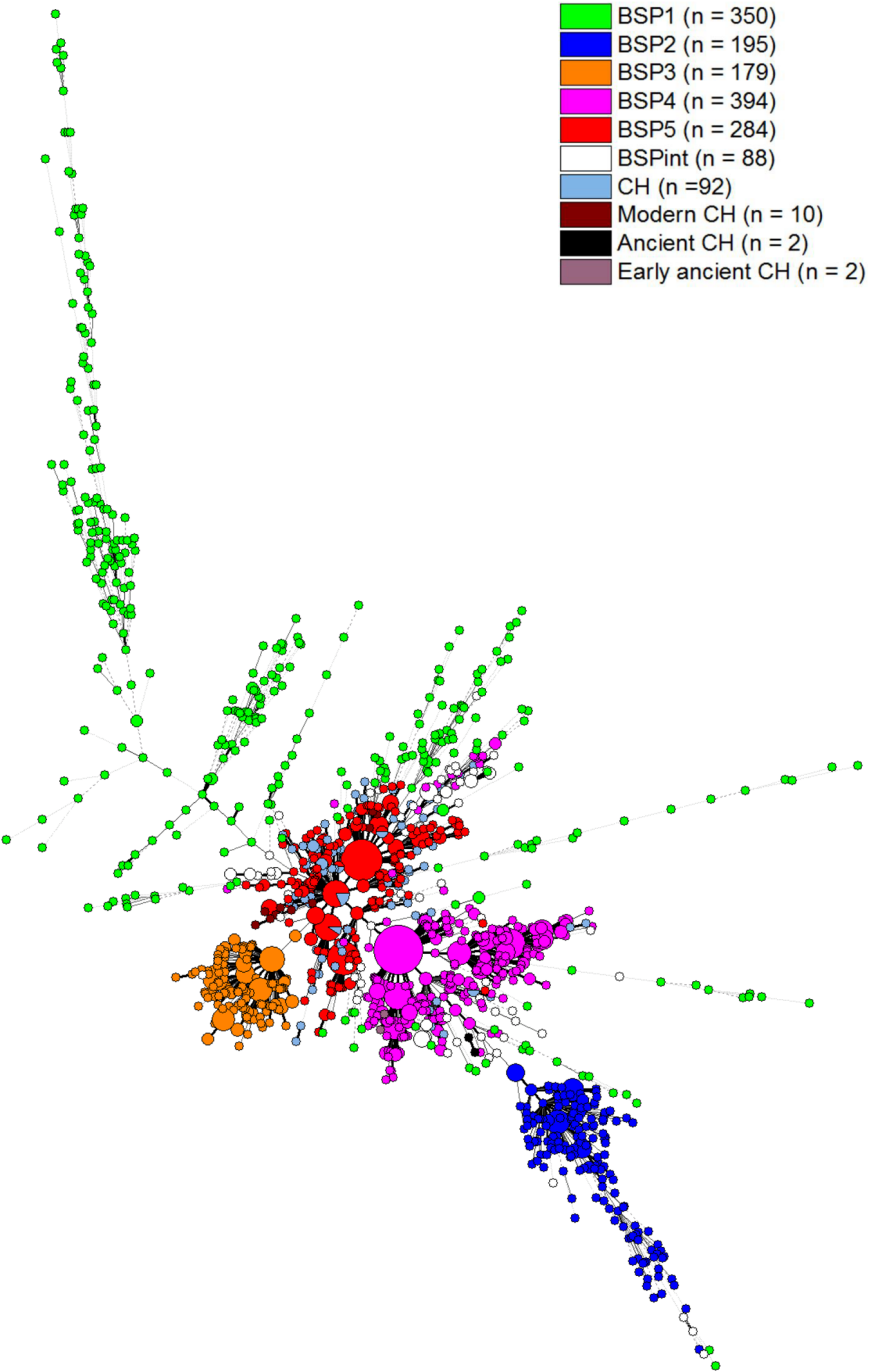
A minimum spanning tree (MST) illustrating evolutionary relationships of cases of clonal heterogeneity observed among Beijing isolates from China versus BSP groupings (n = 1596 strains). The MST was constructed based on 24-loci MIRU-VNTRs on a total of 1490 strains representing different BSP clonal complexes (BSP1 n = 350, BSP2 n = 195, BSP3 n = 179, BSP4 n = 394, BSP5 n = 284, BSPint n = 88), and 106 entries for the group “clonal heterogeneity” representing 53 isolates. Among the latter, 46/53 strains were from our dataset while 7/53 strains were from a recent study by Yin *et al*.^20^, and described as Modern (10 entries from 5 strains), Ancient (2 entries from 1 strain), and Early ancient (2 entries from 1 strain).

A MST (Fig. 3) based on pooled data on all Beijing isolates (n = 6779 strains) was constructed to highlight evolutionary relationships of different Beijing groups described. It included n = 1490 strains from our study on BSP groupings, n = 4987 strains from Merker’s study^19^, and n = 302 Chinese isolates classified as “modern” n = 192, “ancient” n = 81, and “early ancient” n = 29 by Yin *et al*.^20^. It allowed to compare the BSPs described in this paper with the previously described clonal complexes by Merker *et al*.^19^ and Yin *et al*.^20^, revealing that; (i) the three new Beijing clonal complexes described by us as BSP1, BSP2 and BSP3 were not highlighted earlier since these did not overlay with other groups described so far; (ii) among these, BSP2 and BSP3 appeared as tightly-knit groups with remarkable phylogeographical specificities to Chongqing and Taiwan respectively; however, BSP1 which appeared well structured and phylogeographically restricted to Sichuan in the Bayesian STRUCTURE analyses (Fig. 1), was split partially in the MST of Chinese isolates (Fig. 4), and even more dispersed in the global MST without a distinct central node (Fig. 3); this discrepancy in the BSP1 structure needs to be further studied using WGS to understand the finer differences that could explain for phylogeographical restriction of Sichuan BSP1; (iii) BSP4 structured together with CC6 isolates, while the BSP5 was scattered all over major nodes of typical/modern Beijing sublineages CC1 to CC5; (iv) the isolates classified as “modern” by Yin *et al*.^20^ were mostly directly connected to, or close to CC3 and CC4 which were the primary groups of typical/modern Beijing strains in Eastern Asia (Supplementary Figure S2c); (v) two-third (n = 54/81) of isolates classified as “ancient” by Yin *et al*.^20^ overlapped with CC6 and BSP4, while the remaining isolates were mostly associated to, or close to, BL7, BSP1 and Early Ancient isolates; and lastly (vi) the isolates classified as “early ancient” by Yin *et al*.^20^ were scattered over distant branches of loosely-knit BSP1, or close to BL7; nonetheless the number of isolates was too small (n = 29) to conclude.

### The phenomenon of clonal heterogeneity

We further decided to focus on the phenomenon of clonal heterogeneity (CH) defined as “inpatient” microevolution of an infecting clone, as a spin-off of the larger study by adding 24-loci MIRU data on a total of 106 isolates that were defined as cases of clonal heterogeneity^20, 24, 25^. This aimed to explore if a given clonal complex could be more prone to variability (and subsequent geographical adaptability) among the involved isolates. Consequently, a new MST (Supplementary Figure S4) was drawn to investigate the evolutionary relationships of different Beijing subpopulations in the worldwide dataset (n = 6779 strains, including the present study from China) as shown in Fig. 3 above, supplemented with 106 entries corresponding to 53 strains of clonal heterogeneity (total n = 6885). The CH isolates were scattered over the MST especially among the typical/modern Beijing strains (CC1-CC5, limited to BSP5), followed by a few strains linked to atypical ancestral Beijing variants of the CC6/BSP4 group; but none of the CH strains overlapped with the newly described BSP1, BSP2 and BSP3 clonal complexes. Since the CH isolates were all collected from China, we also drew a new MST limited to mainland China and Taiwan (n = 1596 isolates) with different BSP groupings including CH isolates. As shown in Fig. 4, the MST corroborated the fact that all the CH strains are exclusively restricted to BSP4 and BSP5. Indeed, if one considers previous findings concerning modern/ancient/early-ancient Beijing sublineages^19, 20^, it is obvious that BSP5 linked to modern CH isolates (10 entries corresponding to 5 CH strains), while BSP4 linked to ancient and early-ancient CH isolates (2 entries corresponding to 1 CH strain each) in Fig. 4. Thus, the CH isolates from Yin *et al*.^20^ provide a better comprehension of modern vs. ancient Beijing isolates among CH cases, and further suggest why most of CH isolates (without information on modern vs. ancient Beijing strains) appear as connected with BSP5. Lastly, the detection of CH cases among our dataset (n = 92 entries corresponding to 46 strains, Supplementary Figure S3) was optimal with 6 highly discriminatory loci (loci 4052, 424, 1955, 3192, 2163b, 4156); the values being 60.88% for this category vs. 23.90% for the 8 moderately discriminatory loci (loci 3690, 802, 4348, 2996, 2059, 1644, 960, 580), and 15.27% by the 10 poorly discriminatory loci (loci 2401, 2461, 2687, 2531, 3007, 3171, 2165, 154, 577 and 2347).

## Discussion

Based on phylogenetical and Bayesian population structure analyses of 24-loci MIRU-VNTRs, this investigation identified a total of five BSPs in China out of which three clonal complexes (BSP1, BSP2 and BSP3) were described for the first time when the data were compared to previous studies^19, 20^. We showed that the atypical ancestral Beijing isolates (CC6 and BL7) and the typical/modern Beijing isolates (CC1–CC5) were mainly connected together with BSP4 and BSP5, respectively. A recent study revealed that Beijing strains endemic in East Asia were genetically diverse, whereas the globally emerging strains mostly belonged to a highly homogenous “modern” Beijing subpopulation^26^. Both collections by Merker *et al*. and Yin *et al*. primary contained the “globally emerging strains”, which in China correspond to BSP5 as the most homogenous “modern” Beijing, and BSP4 as the globally emerging ancient strains, yet neither included the phylogeographically restricted clonal complexes corresponding to BSP1, BSP2 and BSP3 in our study. Moreover, according to the mean allelic richness as a surrogate indication of diversification time, BSP1 isolates were much older than other BSPs (P < 0.01) as well as the oldest Beijing clonal complex CC6 (P < 0.01) grouped by Merker *et al*.^19^. The age and population expansions of Beijing lineage estimated by Merker *et al*. were challenged by Luo *et al*. who suggested a much earlier time, and such incongruence could be explained by homoplasy affecting MIRU-VNTR markers^26^. However, as evidenced in the Merker *et al*. study^19^, the coalescent analysis based on 24-loci MIRU-VNTRs of Beijing lineage was fairly congruent with the WGS. A more reasonable explanation would be the limitation of sample collection by Merker *et al*., as revealed during the course by our study.

The epidemiology of human TB has been shaped by the long-standing association between MTBC and its human host^27^, hence the different lineages might be adapted to particular human populations. Growing evidence indicate the association of *Mycobacterium tuberculosis* Beijing lineage with drug-resistant, and/or with specific pathobiological or epidemiological manifestations is affected by the existence of substantial intra-lineage biogeographical diversity^19^. Regarding the distribution of BSP clustering (Fig. 1), BSP1, BSP2 and BSP3 showed phylogeographical specificity to Sichuan, Chongqing and Taiwan, respectively, as compared to BSP4 which showed a broader distribution (although highly predominant in Tibet with 76.65% of all isolates). On the other hand, BSP5 was widely distributed in China with the exception of Sichuan and Chongqing. Such geographical restriction as the one described here for BSP1, BSP2 and BSP3 has been previously highlighted recently for several sublineages of lineage 4, and was proposed to correspond to local adaptation of these MTB isolates to local human host populations^28^. Such phenomenon led to the notion of “ecological specialist” in contrast with “generalist pathogen” able to adapt to several human populations. So an important clue to clarify of the phylogeography of these Beijing clonal complexes would be a careful analysis of extrinsic factors as peopling of regions implicated in terms of successive waves of migration vs. region-specific demographics as well as an analysis of intrinsic factors of each clonal complex. Indeed, waves of migrations were successively encouraged by various governments since Yin dynasty (~1600–1000 BC), leading to frequent large-scale migrations in the history of China^29^. The following three sections below briefly review such observations regarding Sichuan, Chongqing, and Taiwan – associated with newly shown BSP clonal complexes.

Sichuan region, which can be divided into three parts: the Sichuan basin, Sichuan northwest plateau and Sichuan southwest mountains, is localized in the southwest of China with indigenous civilizations dating back to at least the 15^th^ century BC. As the most ancient clonal complex in our study, BSP1 exhibited the highest allelic diversity, most diverging branches, and presented relatively lower repeats when compared with other subpopulations, especially in VNTR 424, 4348, 1644, 3007 and 4156 (Fig. 2b and Supplementary Figure S3). The decreasing trend in the number of repeats from modern Beijing isolates to ancestral Beijing in several loci, such as 424, was reported previously and attributed to the evolutionary history of Beijing isolates, and might involve the same events in the related regions^30^. Overall, the BSP1 isolates in Sichuan have been associated with elevated drug resistance^31^, related with heteroresistance and stable coexistence of Manu isolates as mixed in a single host^22^. Nonetheless, whether it can be attributed to their particular population structure remains a question of debate. Sichuan has been inhabited by multiple ethnic groups linked to massive population resettlements in the past, e.g., (i) around 263, during the six dynasties period of Chinese disunity, the non-Han ethnic minority (such as Gelao people from the Yunnan–Guizhou Plateau), began to populate Sichuan where the Han were indigenous; (ii) in the middle of the 17^th^ century, people from the neighboring provinces moved and resettled massively in Sichuan which suffered from a fall in population due to years of turmoil during the Ming-Qing transition^32, 33^. Overall, more people poured in than went out in its history, resulting in the total of 55 ethnic groups with a population of more than 4 million now in Sichuan, probably accounting for BSP1 phylogeographical specificity and genetic diversity.

Chongqing, as the only one municipality in inland China sharing the fertile “Sichuan basin”, was separated from Sichuan in 1997. Similar to Sichuan, it is striking that both BSP4 and BSP5 are not represented in Chongqing, and that BSP2 represent around 97.49% of Beijing isolates in this region. Moreover, VNTR 580, as one of the poorly discriminatory loci worldwide (Supplementary Figure S2b), showed a moderate discriminatory power in our dataset just caused by BSP1 and BSP2 isolates (Fig. 2b and Supplementary Figure S3). One may notice that BSP1 and BSP2 shared same primary pattern for VNTR 580 with 3 repeats at this marker (84.86%, n = 297/350; 95.38%, n = 186/195 respectively; Fig. 2, Supplementary Figures S2b and S3), vs. 2 repeats for other groups. Nonetheless, BSP2 isolates can be clearly discriminated from BSP1 strains by the acquisition of additional copy numbers in VNTR 2059 and 2687. Such a geographical delimitation between BSP1 and BPS2 could indicate contrasted host–pathogen association histories in these regions. Nevertheless, one may notice that Chongqing presents a history of mixed populations contradicting such hypothesis; indeed, later to the massive population resettlements in Sichuan described above, Chongqing underwent additional population changes, e.g., (i) Chongqing became the first inland commerce port open to foreigners, and the British, French, German, US and Japanese consulates were opened in Chongqing in 1890–1904; (ii) the city served as the provisional capital of the Republic of China as well as a partially recognized Korean capital-in-exile, making it the focus of bombing by force, and many factories and universities were relocated from eastern China to Chongqing during the Second World War, transforming this city from inland port to a heavily industrialized city. Whether these contrasted characteristics are sufficient to explain for the phylogeographical restriction of BSP2 isolates in Chongqing (such as selective advantage under the evolutionary pressure putatively linked to the specificity trade and war), remains a matter of debate.

Regarding Taiwan, three main clonal complexes were described: BSP3 accounted for 52.96% (179/338), BSP4 for 20.41% (69/338) and BSP5 for 17.16% (58/338) of all isolates in Taiwan. Focusing on BSP3, restriction to Taiwan could be hypothetically explained easily considering insularity of this region. However, the circulation of several clonal complexes could be linked to successive waves of migrations that happened during the period of the colonial rule and wars and/or to different ethnic and migratory populations in Taiwan. For example, (i) the aborigines inhabited before the 17^th^ century; (ii) the Han Chinese began migrating from Mainland China in the 17^th^ century during the Ming dynasty when the Dutch colonized southern Taiwan; (iii) members of the military, veterans, and some civilians moved from Mainland China between 1945 and 1950 due to the civil war^34^. A chronological trend among Beijing isolates from the three groups was apparent: Beijing isolates from the aborigines had signatures compatible with ancient strains and those from the latter two populations with modern strains^34^. The prevalence of different Beijing isolates in specific ethnic/migratory populations suggested that *M*. *tuberculosis* transmission was limited and restricted to close contact^34, 35^. As one of the main clonal complexes in Taiwan and an atypical ancestral Beijing group in our study, BSP4 was proposed predominantly prevalent in the aboriginal patients. Considering that the expansions of CC1–5 dates back some 200–700 years^19^, the Ming voyages of Zheng He^36^ could have contributed to BSP5 expansion. Thus, it would be interesting in future studies to confirm if BSP5 isolates are more common in the population of Han Chinese whose ancestors migrated to Taiwan during the Ming dynasty. As the most recent clonal complex in this study, BSP3 with specific copy numbers in VNTR 424 (5 repeats in 78.77% isolates, n = 141/179), 3192 (3 repeats in 95.53%, n = 171/179) and 154 (5 repeats in 91.62%, n = 164/179) (Fig. 2, Supplementary Figures S2b and S3) might be related to the latest massive population migration due to the Republic of China policy.

Hence, it would be now of prime interest to perform Bayesian Skyline Plot analyses^37^ using WGS data to reconstruct population size through time and then to estimate demographic history of each Beijing subpopulations within China in comparison with human migrations and populations. Concerning intrinsic factors it would be relevant to use whole genome data of so called “specialist” vs. “generalist” Lineage 2 sublineages in order to decipher underlying genetic mechanisms driving this ecological separation. It was shown before as for example^28^ a contrasted diversity in T cell epitopes in the specialist sublineage L4.6.1/Uganda when compared to generalist sublineages associated with host adaptation and immune escape. Such phenomenon should be now further explored on Beijing clonal complexes.

In agreement with previous suggestions that the differential virulence of modern Beijing vs. ancestral groups might have contributed to their differential spread^38, 39^ (as summarized in Supplementary Figure S2c), one may presume a similar explanation for BSP4 versus BSP5. Indeed, the majority of BSP4 isolates are confined to Tibet, and cluster together (Supplementary Figure S4) with atypical/ancestral CC6 and BL7 clonal complexes described by Merker *et al*.^19^ (mainly represented in Eastern Asia and North America), as well as with the Ancient Beijing isolates identified by Yin *et al*.^20^ (also reportedly prevalent in Tibet). Concerning the Early Ancient Beijing lineage, these isolates were absent in Tibet, an observation that could be linked to the early history of Tibet being devoid of any significant Han Chinese human influx^20, 26^. Regarding BSP5, these strains were scattered all over major nodes of typical/modern Beijing clonal complexes CC1 to CC5, and corresponded to the only clonal complex characterized primarily by 2 copies in VNTR 4156 in the global dataset (Fig. 2b, Supplementary Figures S2b). Interestingly, BSP5 strains were clearly associated with CH isolates in phylogenetic analysis (Supplementary Figure S4), with whom they also shared the characteristic 2 copies in VNTR 4156 (97.89%, n = 278/284 vs. 79.35%, n = 73/92, respectively; Fig. 2b, Supplementary Figure S3). Considering a recent evidence that within patient microevolution of *M*. *tuberculosis* may lead to differential drug-resistance patterns and a heterogeneous response to treatment between lesions^40^, our findings on putative association of BSP clonal complex and CH strains regarding Beijing isolates should be considered cautiously.

## Conclusion

This study compared the structuration of *M*. *tuberculosis* Beijing isolates in mainland China and Taiwan using 24-loci MIRU-VNTR data against published worldwide data. Among the total of five BSPs, three new clonal complexes called BSP1, BSP2 and BSP3 were highlighted for the first time. These three new clonal complexes are characterized by phylogeographical specificities to respectively Sichuan, Chongqing and Taiwan. BSP4 and BSP5 could be regarded as the epitomes of reported global ancient and modern Beijing sublineages in China, respectively. The relationship between BSP5 and CH revealed in our study may have contributed to further global expansion. It is now of prime interest to use WGS data in order to decipher evolutionary histories of these clonal complexes and to explore underlying extrinsic and intrinsic mechanisms explaining geographical restriction of these “ecological specialist” in contrast with global circulation of “generalist pathogen”.

## Methods

### Data collection

The study is based on genotyping data of an initial collection of MTBC clinical isolates (n = 16090) classified as Beijing lineage from mainland China and Taiwan, recovered either from the SITVIT2 database^11^ or from published literature (detailed in Supplementary Table S1). Briefly, our collection contained unpublished genotyping data of 193 isolates from our laboratory; genotyping data of 420 isolates from the SITVIT2 database; and 15477 from published literature, including 2652 also provided in the SITVIT2 database. Available genotyping data comprised spoligotyping and/or MIRU-VNTRs^41–43^. The 24-loci MIRU-VNTR data (n = 1490 Beijing isolates) were recovered from 6 regions, including Tibet, Sichuan, Chongqing, Beijing, Xinjiang and Taiwan. In addition, 68 isolates with clonal heterogeneity were collected in this study, which were identified from a Chinese national survey including 3929 cases from all over the country^24, 25^. Each of the 68 isolates with clonal heterogeneity was divided into 2 distinct patterns (due to twin values for variable loci) bringing their total number to 136 entries for the group “clonal heterogeneity”. Since the 55 isolates from Xinjiang and 68 isolates from the Chinese national survey were without spoligotyping data, these were subjected to MIRU-VNTRplus web tool^41^ for lineage classification, and 49/55 and 46/68 isolates (corresponding to 92/136 entries due to twin values for variable loci) respectively were identified as Beijing genotype. Additionally, we also used published 24-loci MIRU-VNTR data from two recent studies to construct a global Beijing Minimum Spanning Tree (MST); the first set comprised a global collection of 4987 Beijing isolates from 99 countries^19^, while the second set comprised data on 302 Beijing strains from China country-wide^20^. Besides, the latter study also contained 7 cases of clonal heterogeneity (corresponding to 14 entries due to twin values for variable loci) that was further analyzed for studying the phenomenon of clonal heterogeneity.

### Ethics statements

Genotyping data were already published or extracted as anonymized data from the SITVIT2 database (Supplementary Tables S1 and S2).

### Phylogenetic inferences

MST algorithm was applied on global 24-loci MIRU-VNTR data using BioNumerics software 6.6 (Applied Maths, Sint-Martens-Latem, Belgium) in order to infer the potential evolutionary relationships between strains. The identical MIRU-VNTR haplotypes in the MST were pooled as a single node representing a cluster, and the rate of clustered strains was considered as an indicator for the extent of recent transmission^19, 44^.

### Population structure analyses

The STRUCTURE software (version 2.3) was used to confirm the inferences by using an admixture model which can deal with complexities of data considering that individuals with mixed ancestry may have inherited part of their genome from ancestors in population K. Posterior estimates for the parameters of interest were computed by using a Markov chain Monte Carlo (MCMC) algorithm in ten parallel chains with a burn-in of 100,000 iterations and a run length of 10^6^. The Evanno method was used to calculate the delta K in the program STRUCTURE HARVESTER^45, 46^. To guarantee the optimum clustering, medians were calculated from 10 replicates for K by using the FullSearch algorithm implemented in CLUMPP 1.1.2 software^47^, and a cutoff of 0.6 was fixed for clustering of isolates. Results of admixture coefficients were then displayed spatially by an interpolation technique called universal kriging: Q-matrix were represented on separate maps (ETOPO1 map produced by NOAA^48^ freely available as indicated here: https://www.ngdc.noaa.gov/mgg/global/dem_faq.html#sec-2.4) for each K by using the script ‘plot.admixture.r’ (available through TESS website: http://membres-timc.imag.fr/Olivier.Francois/tess.html) using R software^49^.

### Genetic characteristics

Mean allelic richness in each MTB clonal complexes or geographical regions was estimated using a rarefaction procedure implemented in the software HP-RARE 1.0 which compensates for sampling disparities^50^; differences were analyzed using t-test. Comparison of number of repeats at each VNTR locus for each population was studied by Pearson’s chi-square exact test (two-tailed). The data were analyzed using the Stata statistical software (version 12; Stata Corporation, College Town, TX, USA) and statistical significance was considered for P values < 0.05. WebLogo^51^ was used to visualize main patterns of tandem repeats for 24-loci MIRU-VNTRs. The Hunter-Gaston discriminatory index (HGDI) was calculated as described previously^52^, and the allelic diversity of the loci was classified as highly discriminatory loci (HGDI > 0.6), moderately discriminatory loci (0.3 ≤ HGDI ≤ 0.6) and poorly discriminatory loci (HGDI < 0.3) according to Sola *et al*.^53^.

## Electronic supplementary material

Supplementary Information

## References

[1] Global tuberculosis report, World Health Organization, Geneva, Switzerland (2015).

[2] Gagneux S (2006). Variable host-pathogen compatibility in *Mycobacterium tuberculosis*. Proc. Natl. Acad. Sci..

[3] Bifani PJ, Mathema B, Kurepina NE, Kreiswirth BN (2002). Global dissemination of the *Mycobacterium tuberculosis* W-Beijing family strains. Trends Microbiol..

[4] Glynn JR (2002). Worldwide occurrence of Beijing/W strains of *Mycobacterium tuberculosis*: a systematic review. Emerg. Infect. Dis..

[5] Hanekom M (2013). Population structure of mixed *Mycobacterium tuberculosis* infection is strain genotype and culture medium dependent. PLoS one..

[6] Cox HS (2005). The Beijing genotype and drug resistant tuberculosis in the Aral Sea region of Central Asia. Respir. Res..

[7] Ford CB (2013). *Mycobacterium tuberculosis* mutation rate estimates from different lineages predict substantial differences in the emergence of drug-resistant tuberculosis. Nat. Genet..

[8] Thomas SK (2011). Modern and ancestral genotypes of *Mycobacterium tuberculosis* from Andhra Pradesh, India. PLoS one..

[9] Niemann S (2010). *Mycobacterium tuberculosis* Beijing lineage favors the spread of multidrug-resistant tuberculosis in the Republic of Georgia. J. Clin. Microbiol..

[10] Brudey K (2006). *Mycobacterium tuberculosis* complex genetic diversity: mining the fourth international spoligotyping database (SpolDB4) for classification, population genetics and epidemiology. BMC. Microbiol..

[11] Couvin D, Rastogi N (2015). Tuberculosis - A global emergency: Tools and methods to monitor, understand, and control the epidemic with specific example of the Beijing lineage. Tuberculosis (Edinb)..

[12] De Beer JL (2013). Comparative study of IS*6110* restriction fragment length polymorphism and variable-number tandem-repeat typing of *Mycobacterium tuberculosis* isolates in the Netherlands, based on a 5-year nationwide survey. J. Clin. Microbiol..

[13] Supply P (2006). Proposal for standardization of optimized mycobacterial interspersed repetitive unit-variable-number tandem repeat typing of *Mycobacterium tuberculosis*. J. Clin. Microbiol..

[14] Liu HC (2016). Molecular typing characteristic and drug susceptibility analysis of *Mycobacterium tuberculosis* isolates from Zigong, China. Biomed. Res. Int..

[15] Chen YY (2014). Distinct modes of transmission of tuberculosis in aboriginal and non-aboriginal populations in Taiwan. PLoS One..

[16] Cohen T (2011). Mixed-strain *Mycobacterium tuberculosis* infections among patients dying in a hospital in KwaZulu-Natal, South Africa. J. Clin. Microbiol..

[17] Iwamoto T (2012). Genetic diversity and transmission characteristics of Beijing family strains of *Mycobacterium tuberculosis* in Peru. PLoS One..

[18] Comas I (2009). Genotyping of genetically monomorphic bacteria: DNA sequencing in *Mycobacterium tuberculosis* highlights the limitations of current methodologies. PLoS one..

[19] Merker M (2015). Evolutionary history and global spread of the *Mycobacterium tuberculosis* Beijing lineage. Nat. Genet..

[20] Yin Q (2016). Evolutionary history and ongoing transmission of phylogenetic sublineages of *Mycobacterium tuberculosis* Beijing Genotype in China. Sci. Rep..

[21] Streit E, Millet J, Rastogi N (2015). *Mycobacterium tuberculosis* polyclonal infections and microevolution identified by MIRU-VNTRs in an epidemiological study. Int. J. Mycobacteriol..

[22] Zheng C (2015). Mixed infections and rifampin heteroresistance among *Mycobacterium tuberculosis* clinical isolates. J. Clin. Microbiol..

[23] Demay C (2012). SITVITWEB–a publicly available international multimarker database for studying *Mycobacterium tuberculosis* genetic diversity and molecular epidemiology. Infect. Genet. Evol..

[24] Pang Y (2015). Prevalence and risk factors of mixed *Mycobacterium tuberculosis* complex infections in China. J. Infect..

[25] Zhao Y (2012). National survey of drug-resistant tuberculosis in China. N. Engl. J. Med..

[26] Luo T (2015). Southern East Asian origin and coexpansion of *Mycobacterium tuberculosis* Beijing family with Han Chinese. Proc. Natl. Acad. Sci..

[27] Gagneux S (2012). Host-pathogen coevolution in human tuberculosis. Philos. Trans. R. Soc. Lond. B. Biol. Sci..

[28] Stucki D (2016). *Mycobacterium tuberculosis* lineage 4 comprises globally distributed and geographically restricted sublineages. Nat. Genet..

[29] LaPolla, R. J. *The role of migration and language contact in the development of the Sino-Tibetan language family*. In areal diffusion and genetic inheritance: case studies in language change (eds Dixon R. M. W. & A. Y. Aikhenvald). (Oxford University Press, 1999).

[30] Chen YY (2012). Genetic Diversity of the *Mycobacterium tuberculosis* Beijing family based on SNP and VNTR typing profiles in Asian countries. PLos one..

[31] Zheng C (2014). Suitability of IS*6110*-RFLP and MIRU-VNTR for differentiating spoligotyped drug-resistant *Mycobacterium tuberculosis* clinical isolates from Sichuan in China. Biomed. Res. Int..

[32] Parsons JB (1957). The culmination of a Chinese peasant rebellion: changhsien-chung in Szechwan, 1644–1646. The Journal of Asian Studies..

[33] Dai, Y. *The Sichuan frontier and Tibet: imperial strategy in the early Qing* (University of Washington Press, 2009).

[34] Dou HY (2008). Associations of *Mycobacterium tuberculosis* genotypes with different ethnic and migratory populations in Taiwan. Infect. Genet. Evol..

[35] Dou HY, Chen YY, Kou SC, Su IJ (2015). Prevalence of *Mycobacterium tuberculosis* strain genotypes in Taiwan reveals a close link to ethnic and population migration. J. Formos. Med. Assoc..

[36] Tamura, E. H., Mention, L. K., Lush, N. W., Tsui, F. K. C. & Cohen, W. *China: understanding its past* (University of Hawaii Press, 1997).

[37] Drummond AJ, Rambaut A, Shapiro B, Pybus OG (2005). Bayesian coalescent inference of past population dynamics from molecular sequences. Mol. Biol. Evol..

[38] Aguilar D (2010). *Mycobacterium tuberculosis* strains with the Beijing genotype demonstrate variability in virulence associated with transmission. Tuberculosis..

[39] Ribeiro SC (2014). *Mycobacterium tuberculosis* strains of the modern sublineage of the Beijing family are more likely to display increased virulence than strains of the ancient sublineage. J. Clin. Microbiol..

[40] Liu Q (2015). Within patient microevolution of *Mycobacterium tuberculosis* correlates with heterogeneous responses to treatment. Sci. Rep..

[41] Weniger T (2010). MIRU-VNTRplus: a web tool for polyphasic genotyping of *Mycobacterium tuberculosis* complex bacteria. Nucleic. Acids. Res..

[42] Supply P (2006). Proposal for standardization of optimized mycobacterial interspersed repetitive unit-variable-number tandem repeat typing of *Mycobacterium tuberculosis*. J. Clin. Microbiol..

[43] Kamerbeek J (1997). Simultaneous detection and strain differentiation of *Mycobacterium tuberculosis* for diagnosis and epidemiology. J. Clin. Microbiol..

[44] Reynaud Y, Millet J, Rastogi N (2015). Genetic structuration, demography and evolutionary history of *Mycobacterium tuberculosis* LAM9 sublineage in the Americas as two distinct subpopulations revealed by bayesian analyses. PLoS one..

[45] Evanno G, Regnaut S, Goudet J (2005). Detecting the number of clusters of individuals using the software STRUCTURE: a simulation study. Mol. Ecol..

[46] Earl DA (2011). & vonHoldt, B. M. STRUCTURE HARVESTER: a website and program for visualizing STRUCTURE output and implementing the Evanno method. Conserv. Genet. Resour..

[47] Jakobsson M, Rosenberg NA (2007). CLUMPP: a cluster matching and permutation program for dealing with label switching and multimodality in analysis of population structure. Bioinformatics..

[48] Amante, C. & Eakins, B. W. ETOPO1 1 arc-minute global relief model: procedures, data sources and analysis. In: NOAA Technical Memorandum NESDIS NGDC-24. National Geophysical Data Center, NOAA.

[49] R Core Team. R: a language and environment for statistical computing. https://www.R-project.org (R Foundation for Statistical Computing,Vienna, Austria, 2016).

[50] Kalinowski ST (2005). Hp-rare 1.0: a computer program for performing rarefaction on measures of allelic richness. Mol. Ecol. Notes..

[51] Olsen LR (2013). BlockLogo: visualization of peptide and sequence motif conservation. J. Immun. Methods..

[52] Hunter PR, Gaston MA (1988). Numerical index of the discriminatory ability of typing systems: an application of Simpson’s index of diversity. J. Clin. Microbiol..

[53] Sola C (2003). Genotyping of the *Mycobacterium tuberculosis* complex using MIRUs: association with VNTR and spoligotyping for molecular epidemiology and evolutionary genetics. Infect. Genet. Evol..

