# Supplementary material for: New Mycobacterium tuberculosis Beijing clonal complexes in China revealed by phylogenetic and Bayesian population structure analyses of 24-loci MIRU-VNTRs
Source: Sci Rep. 2017 Jul 20;7:6065. doi: 10.1038/s41598-017-06346-1 (PMC5519585; doi:10.1038/s41598-017-06346-1)
Supplement: Supplementary file 1 — Supplementary Information [file 41598_2017_6346_MOESM1_ESM.pdf]

Supporting Information

Title:

New *Mycobacterium tuberculosis* Beijing clonal complexes in China revealed by phylogenetic and Bayesian population structure analyses of 24-loci MIRU-VNTRs

Authors:

Chao Zheng[1,2], Yann Reynaud[2*], Changsong Zhao[1], Thierry Zozio[2], Song Li[1], Dongxia Luo[3], Qun Sun[1**] and Nalin Rastogi[2*]

Table S1. Data collection references in this study. Please refer to Table S2 for detailed 24-loci MIRU-VNTR data.

Serial No.	No. of isolates	MIRU-VNTR data format				Spoligotyping data	Reference	PubMed ID (PMID)	Data availability in SITVIT2 database
		12 loci	15 loci	24 loci	other sets				
**1**	576 [a]	X [b]	X	X		X	Dong, H. *et al*. Genetic diversity of *Mycobacterium tuberculosis* isolates from Tibetans in Tibet, China. *PLoS one.* **7,** e33904 (2012).	22479472	
**2**	338	X	X	X			Chen, Y. *et al*. Genetic diversity of the *Mycobacterium tuberculosis* Beijing family based on SNP and VNTR typing profiles in asian countries. *PLoS one.* **7,** e39792 (2012).	22808061	
**3**	306	X	X	X		X	Zhao, Y. *et al*. The population structure of drug-resistant *Mycobacterium tuberculosis* clinical isolates from Sichuan in China. *Infect. Genet. Evol.* **12,** 718-724 (2012).	21989209	X
**4**	297	X	X	X		X	Zhang, D. *et al*. Molecular typing and drug susceptibility of *Mycobacterium tuberculosis* isolates from Chongqing municipality, China. *Infect. Genet. Evol.* **13,** 310-316 (2013).	23183314	
**5**	193	X	X	X		X	Unpublished data from Sichuan [c]		
**6**	72	X	X	X			Jiao, W. W. *et al*. Evaluation of new variable-number tandem-repeat systems for typing *Mycobacterium tuberculosis* with Beijing genotype isolates from Beijing, China. *J. Clin. Microbiol.* **46,** 1045-1049 (2008).	18199785	
**7**	55	X	X	X			Zhang, J. *et al*. Genotypes and drug susceptibility of *Mycobacterium tuberculosis* isolates in Shihezi, Xinjiang province, China. *BMC. Res. Notes.* **5,** 309 (2012).	22713520	
**8**	84	X	X	X		X	Hu, Y. *et al*. Acquisition of second-line drug resistance and extensive drug resistance during recent transmission of *Mycobacterium tuberculosis* in rural China. *Clin. Microbiol. Infect.* **21,** 1093.e9-1093.e18 (2015).	26348262	
**9**	372		X		X	X	Yu, Q. *et al*. Genetic diversity of *Mycobacterium tuberculosis* isolates from Inner Mongolia, China. *PLoS one.* **8,** e57660 (2013).	23658680	
**10**	260	X	X		X	X	Liu, Q. *et al*. Molecular typing of *Mycobacterium tuberculosis* isolates circulating in Jiangsu province, China. *BMC. Infect. Dis.* **11,** 288 (2011).	22026819	
**11**	260		X		X	X	Lu, B. *et al*. Genetic diversity of *Mycobacterium tuberculosis* isolates from Beijing, China assessed by spoligotyping, LSPs and VNTR profiles. *BMC. Infect. Dis.* **12,** 372 (2012).	23259861	
**12**	123		X				Yuan, X. *et al*. Genotyping and clinical characteristics of multidrug and extensively drug-resistant tuberculosis in a tertiary care tuberculosis hospital in China. *BMC. Infect. Dis.* **13,** 315 (2013).	23849244	
**13**	123	X			X	X	Chen, L. *et al*. Genetic diversity and drug susceptibility of *Mycobacterium tuberculosis* isolates from Zunyi, one of the highest-incidence-rate areas in China. *J. Clin. Microbiol.* **50,** 1043-1047 (2011).	22205809	X
**14**	364	X					Sun, J. R. *et al*. Epidemiological studies of Beijing strains of *Mycobacterium tuberculosis* from Taipei and other asian cities based on MIRU profiles. *APMIS.* **119,** 581-587 (2011).	21851415	
**15**	315	X					Dou, H. Y. *et al*. Molecular epidemiology and evolutionary genetics of *Mycobacterium tuberculosis* in Taipei. *BMC. Infect. Dis.* **8,** 170 (2008).	19102768	
**15**	349					X	Dou, H. Y. *et al*. Molecular epidemiology and evolutionary genetics of *Mycobacterium tuberculosis* in Taipei. *BMC. Infect. Dis.* **8,** 170 (2008).	19102768	
**16**	211	X					Kam, K. M. *et al*. Utility of mycobacterial interspersed repetitive unit typing for differentiating multidrug-resistant *Mycobacterium tuberculosis* isolates of the Beijing family. *J. Clin. Microbiol.* **43,** 306-313 (2005).	15634987	

**17**	186	X		X	Chen, Y. *et al*. Potential challenges to the stop TB plan for humans in China; cattle maintain *M. bovis* and *M. tuberculosis*. *Tuberculosis.* **89,** 95-100 (2009).	19056318	
**18**	105	X		X	Han, H. *et al*. Utility of mycobacterial interspersed repetitive unit typing for differentiating *Mycobacterium tuberculosis* isolates in Wuhan, China. *J. Med. Microbiol.* **56,** 1219-1223 (2007).	17761486	
**19**	103	X			Xing, L. *et al*. Clinical and genotypic characteristics of childhood tuberculosis in Chongqing, China. *Eur. J. Clin. Microbiol. Infect. Dis.* **31,** 1735-1739 (2012).	22161015	
**20**	89	X			Guo, J. *et al*. Mycobacterial interspersed repetitive unit typing in *Mycobacterium tuberculosis* isolates from Sichuan province in China. *Indian. J. Med. Res.* **134,** 362-368 (2011).	21985820	
**21**	88	X			Pang, Y. *et al*. A novel method based on high resolution melting (HRM) analysis for MIRU-VNTR genotyping of *Mycobacterium tuberculosis*. *J. Microbiol. Methods.* **86,** 291-297 (2011).	21689691	
**22**	71	X			Zhou, A. *et al*. Molecular genotyping of *Mycobacterium tuberculosis* in Xi'an, China, using MIRU-VNTR typing. *Int. J. Tuberc. Lung. Dis.* **15,** 517-522 (2011).	21396212	
**22**	195			X	Zhou, A. *et al*. Molecular genotyping of *Mycobacterium tuberculosis* in Xi'an, China, using MIRU-VNTR typing. *Int. J. Tuberc. Lung. Dis.* **15,** 517-522 (2011).	21396212	
**23**	1586 [d]		X	X	Liu, Y. *et al*. Genotypic diversity analysis of *Mycobacterium tuberculosis* strains collected from Beijing in 2009, using spoligotyping and VNTR typing. *PLoS one.* **9,** e106787 (2014).	25237849	
**24**	467		X	X	Liu, J. *et al*. First insight into the genotypic diversity of clinical *Mycobacterium tuberculosis* isolates from Gansu province, China. *PLoS one.* **9,** e99357 (2014).	24911588	
**25**	69		X	X	Kremer, K. *et al*. Use of variable-number tandem-repeat typing to differentiate *Mycobacterium tuberculosis* Beijing family isolates from Hong Kong and comparison with IS*6110* restriction fragment length polymorphism typing and spoligotyping. *J. Clin. Microbiol.* **43,** 314-320 (2005).	15634988	
**26**	64		X	X	Liu, J. *et al*. Molecular characteristics and drug susceptibility of *Mycobacterium tuberculosis* isolates from patients co-infected with human immunodeficiency virus in Beijing, China. *Biomed. Environ. Sci.* **28,** 222-226 (2015).	25800448	
**27**	3006			X	Pang, Y. *et al*. Spoligotyping and drug resistance analysis of *Mycobacterium tuberculosis* strains from national survey in China. *PLoS one.* **7,** e32976 (2012).	22412962	
**28**	2346			X	Dong, H. *et al*. Spoligotypes of *Mycobacterium tuberculosis* from different provinces of China. *J. Clin. Microbiol.* **48,** 4102-4106 (2010).	20739484	X
**29**	497			X	Lu, W. *et al*. Genotypes of *Mycobacterium tuberculosis* isolates in rural China: using MIRU-VNTR and spoligotyping methods. *Scand. J. Infect. Dis.* **46,** 98-106 (2014).	24359517	
**30**	287			X	Pang, H. *et al*. Molecular characterization and drug-resistance of *Mycobacterium tuberculosis* strains in Xuzhou, China. *Biomed. Environ. Sci.* **27,** 960-964 (2014).	25484012	
**31**	230			X	Li, X. *et al*. Non-Beijing strains of *Mycobacterium tuberculosis* in China. *J. Clin. Microbiol.* **49,** 392-395 (2010).	21068281	X
**32**	207			X	Zhang, D. *et al*. Genetic diversity of multidrug-resistant tuberculosis in a resource-limited region of China. *Int. J. Infect. Dis.* **29,** 7-11 (2014).	25312982	
**33**	206			X	Jiang, Y. *et al*. 19-VNTR loci used in genotyping Chinese clinical *Mycobacterium tuberculosis* complex strains and in association with spoligotyping. *J. Basic. Microbiol.* **53,** 562-580 (2013).	23322548	

**34**	131		X	Hu, Y. *et al*. Extensive transmission of isoniazid resistant *M. tuberculosis* and its association with increased multidrug-resistant TB in two rural counties of eastern China: a molecular epidemiological study. *BMC. Infect. Dis.* **10,** 43 (2010).	20187977	
**35**	89		X	Zhou, A. *et al*. Molecular characterization of isoniazid-resistant *Mycobacterium tuberculosis* isolates from Xi'an, China. *Microb. Drug. Resist.* **17,** 275-281 (2011).	21388297	
**36**	67		X	Data extracted from the SITVIT2 database.		X
**37**	44		X	Wang, J. *et al*. Genotypes and characteristics of clustering and drug susceptibility of *Mycobacterium tuberculosis* isolates collected in Heilongjiang province, China. *J. Clin. Microbiol.* **49,** 1354-1362 (2011).	21325562	
**38**	40		X	Dou, H. Y. *et al*. Associations of *Mycobacterium tuberculosis* genotypes with different ethnic and migratory populations in Taiwan. *Infect. Genet. Evol.* **8,** 323-330 (2008).	18378194	
**39**	33		X	Chen, Y. Y. *et al*. Molecular epidemiology of *Mycobacterium tuberculosis* in aboriginal peoples of Taiwan, 2006-2011. *J. Infect.* **68,** 332-337 (2014).	24370561	
**40**	1586	X		Wan, K. *et al*. Investigation on *Mycobacterium tuberculosis* diversity in China and the origin of the Beijing clade. *PLoS one.* **6,** e29190 (2011).	22220207	

[a] Among 576 isolates, both 24-loci MIRU-VNTRs data and spoligotyping data were available for a total of 517 isolates, and 59 isolates had spoligotyping data only.

[b] The letter "X" denotes availability of data.

[c] Study carried out in Key Laboratory of Bio-resources and Eco-environment of the Ministry of Education, College of Life Sciences, Sichuan University, Chengdu, Sichuan 610065, P. R. China; Contact: Qun sun, E-mail: qunsun@scu.edu.cn

[d] Among 1586 isolates, spoligotyping data were available for all except one strain, and a subset of 1053 isolates was further analyzed by MIRU-VNTRs (including the strain missing spoligotyping data).

Table S2. 24-loci MIRU-VNTRs genotyping results of the *M. tuberculosis* Beijing isolates (n=1490).

Strain Number	Year	Reference & PMID	Population assigned	Region of Isolation	Latitude	Longitude	154	580	960	1644	2059	2531	2687	2996	3007	3192	4348	802	2165	2461	577	2163b	4052	4156	424	1955	2347	2401	3171	3690
B1	2011	Zhang, D. *et al.* & 23183314	BSP2	Chongqing	29.56	106.55	2	3	3	3	4	5	2	6	3	4	3	3	4	2	4	6	9	3	4	6	4	4	3	4
B2	2011	Zhang, D. *et al.* & 23183314	BSP2	Chongqing	29.56	106.55	2	3	3	3	4	5	2	7	3	5	4	3	4	2	4	6	9	3	4	4	4	4	3	4
B3	2011	Zhang, D. *et al.* & 23183314	BSP2	Chongqing	29.56	106.55	2	3	3	3	4	5	2	7	3	5	3	3	4	2	4	6	9	3	4	1	4	4	3	4
B4	2011	Zhang, D. *et al.* & 23183314	BSP2	Chongqing	29.56	106.55	2	3	3	3	4	5	2	5	3	5	3	4	4	2	3	6	9	3	4	4	4	4	3	4
B5	2011	Zhang, D. *et al.* & 23183314	BSP2	Chongqing	29.56	106.55	2	3	3	2	4	5	2	7	3	5	3	4	4	2	4	6	5	3	4	4	4	4	3	4
B6	2011	Zhang, D. *et al.* & 23183314	BSP2	Chongqing	29.56	106.55	2	3	3	2	4	5	2	7	3	5	3	3	4	2	4	6	5	3	4	4	4	4	3	4
B7	2011	Zhang, D. *et al.* & 23183314	BSP2	Chongqing	29.56	106.55	2	3	3	1	4	5	2	7	3	5	3	3	4	2	4	6	9	3	4	5	4	4	3	4
B8	2011	Zhang, D. *et al.* & 23183314	BSP2	Chongqing	29.56	106.55	2	3	3	3	4	5	2	7	3	5	3	3	4	2	3	6	9	3	4	4	4	4	3	4
B9	2011	Zhang, D. *et al.* & 23183314	BSP2	Chongqing	29.56	106.55	2	3	3	1	4	5	2	8	3	5	3	3	4	2	4	6	9	3	4	4	4	4	3	2
B10	2011	Zhang, D. *et al.* & 23183314	BSP2	Chongqing	29.56	106.55	2	3	3	2	4	5	2	7	3	4	3	3	4	2	4	6	9	3	4	4	4	4	3	4
B11	2011	Zhang, D. *et al.* & 23183314	BSP2	Chongqing	29.56	106.55	2	3	3	3	4	6	2	7	3	5	3	3	4	2	4	6	9	3	4	4	4	4	3	4
B12	2011	Zhang, D. *et al.* & 23183314	BSP2	Chongqing	29.56	106.55	2	1	3	3	4	5	2	7	3	5	3	4	4	2	4	5	5	3	4	4	4	4	3	4
B13	2011	Zhang, D. *et al.* & 23183314	BSP2	Chongqing	29.56	106.55	2	3	3	3	4	5	2	7	3	5	3	3	4	2	4	4	7	3	4	4	4	4	3	4
B14	2011	Zhang, D. *et al.* & 23183314	BSP2	Chongqing	29.56	106.55	2	3	3	3	4	5	2	7	3	5	3	3	4	2	4	6	8	3	4	4	4	4	3	4
B15	2011	Zhang, D. *et al.* & 23183314	BSP2	Chongqing	29.56	106.55	2	3	3	2	4	5	2	5	3	3	3	3	4	1	4	4	8	3	4	3	4	4	3	4
B16	2011	Zhang, D. *et al.* & 23183314	BSP2	Chongqing	29.56	106.55	2	3	2	3	4	5	2	7	3	5	3	3	4	2	4	6	8	3	5	4	4	4	3	4
B17	2011	Zhang, D. *et al.* & 23183314	BSP2	Chongqing	29.56	106.55	2	3	3	3	4	5	2	7	3	5	3	3	4	2	4	4	9	3	4	4	4	4	3	2
B18	2011	Zhang, D. *et al.* & 23183314	BSP2	Chongqing	29.56	106.55	2	3	2	3	4	5	2	7	3	4	3	4	3	2	4	4	9	3	4	4	4	4	3	2
B19	2011	Zhang, D. *et al.* & 23183314	BSP2	Chongqing	29.56	106.55	2	3	2	3	4	5	2	6	3	5	3	4	4	2	4	6	ND	3	4	4	4	4	3	4
B20	2011	Zhang, D. *et al.* & 23183314	BSP2	Chongqing	29.56	106.55	2	3	3	3	4	5	2	6	3	3	3	3	4	2	4	3	9	3	4	3	4	4	3	4
B21	2011	Zhang, D. *et al.* & 23183314	BSP2	Chongqing	29.56	106.55	2	3	3	3	4	5	2	6	3	5	3	3	4	2	4	6	9	3	4	6	4	4	3	4
B22	2011	Zhang, D. *et al.* & 23183314	BSP2	Chongqing	29.56	106.55	2	3	3	3	4	5	2	5	3	3	3	4	4	2	4	4	8	3	4	3	4	4	3	4
B23	2011	Zhang, D. *et al.* & 23183314	BSP2	Chongqing	29.56	106.55	2	3	3	3	4	5	2	7	3	5	3	3	4	2	4	4	8	3	4	4	4	4	3	4
B24	2011	Zhang, D. *et al.* & 23183314	BSP2	Chongqing	29.56	106.55	2	3	3	3	4	5	2	7	3	5	3	3	4	2	4	6	7	3	4	4	4	4	3	4
B25	2011	Zhang, D. *et al.* & 23183314	BSP2	Chongqing	29.56	106.55	2	2	3	3	4	5	2	6	3	5	3	3	4	2	4	7	9	3	4	4	4	4	3	4
B26	2011	Zhang, D. *et al.* & 23183314	BSP2	Chongqing	29.56	106.55	2	3	2	3	4	5	2	5	3	4	2	3	4	2	4	2	8	3	4	3	4	4	3	3
B27	2011	Zhang, D. *et al.* & 23183314	BSP2	Chongqing	29.56	106.55	2	3	2	2	4	5	2	7	2	5	3	5	4	2	4	5	9	3	4	4	4	4	3	4
B28	2011	Zhang, D. *et al.* & 23183314	BSP2	Chongqing	29.56	106.55	2	3	2	3	3	5	2	4	3	3	3	3	4	2	4	3	6	3	4	3	4	4	3	4
B29	2011	Zhang, D. *et al.* & 23183314	BSP2	Chongqing	29.56	106.55	2	3	2	3	4	4	2	8	3	5	3	3	4	2	4	6	9	3	4	5	4	4	3	4
B30	2011	Zhang, D. *et al.* & 23183314	BSP2	Chongqing	29.56	106.55	2	3	2	3	4	5	2	5	3	3	3	5	4	2	4	5	9	3	4	4	4	4	3	4
B31	2011	Zhang, D. *et al.* & 23183314	BSP2	Chongqing	29.56	106.55	2	3	3	3	4	5	2	7	3	5	3	3	4	2	4	5	5	3	4	4	4	4	3	4
B32	2011	Zhang, D. *et al.* & 23183314	BSP2	Chongqing	29.56	106.55	2	3	3	3	4	5	2	7	3	5	3	3	4	2	2	7	7	5	4	4	4	4	3	2
B33	2011	Zhang, D. *et al.* & 23183314	BSP2	Chongqing	29.56	106.55	2	3	3	3	4	5	2	5	3	3	3	3	3	2	4	2	7	3	5	3	4	4	3	2
B34	2011	Zhang, D. *et al.* & 23183314	BSP2	Chongqing	29.56	106.55	2	3	1	3	4	5	2	8	3	5	3	3	2	2	4	6	9	3	4	4	4	4	3	4
B35	2011	Zhang, D. *et al.* & 23183314	BSP2	Chongqing	29.56	106.55	2	3	3	3	4	5	2	7	3	4	3	3	4	2	4	5	7	3	4	5	4	4	3	4
B36	2011	Zhang, D. *et al.* & 23183314	BSP2	Chongqing	29.56	106.55	2	3	3	3	3	5	2	5	3	4	3	5	4	2	4	5	9	3	4	3	4	4	3	4
B37	2011	Zhang, D. *et al.* & 23183314	BSP2	Chongqing	29.56	106.55	2	3	3	3	4	5	2	7	3	5	3	3	4	2	4	5	7	3	6	5	4	4	3	4
B38	2011	Zhang, D. *et al.* & 23183314	BSP2	Chongqing	29.56	106.55	2	3	3	3	4	5	2	7	3	6	3	3	4	2	3	4	7	5	4	4	4	4	3	4

B39	2011	Zhang, D. *et al.* & 23183314	BSP2	Chongqing	29.56	106.55	2	3	3	3	4	5	2	7	3	4	3	3	4	2	4	3	9	3	4	3	4	4	3	4
B40	2011	Zhang, D. *et al.* & 23183314	BSP2	Chongqing	29.56	106.55	2	3	3	3	4	5	2	7	3	4	3	3	4	2	4	3	9	3	4	3	4	4	3	4
B41	2011	Zhang, D. *et al.* & 23183314	BSP2	Chongqing	29.56	106.55	2	3	2	2	4	5	2	8	3	4	3	3	4	2	4	1	9	3	4	5	4	4	3	4
B42	2011	Zhang, D. *et al.* & 23183314	BSP2	Chongqing	29.56	106.55	2	3	3	3	4	5	2	8	3	5	3	3	4	2	4	6	9	3	4	4	4	4	3	4
B43	2011	Zhang, D. *et al.* & 23183314	BSP2	Chongqing	29.56	106.55	2	3	3	3	4	5	2	8	3	5	3	3	4	2	4	5	9	3	4	6	4	4	3	4
B44	2011	Zhang, D. *et al.* & 23183314	BSP2	Chongqing	29.56	106.55	2	3	3	3	4	5	2	8	3	4	3	4	4	2	4	6	9	5	6	4	4	4	3	2
B45	2011	Zhang, D. *et al.* & 23183314	BSP2	Chongqing	29.56	106.55	2	3	3	3	4	5	2	7	3	5	3	3	4	2	4	5	9	3	6	5	4	4	3	4
B46	2011	Zhang, D. *et al.* & 23183314	BSP2	Chongqing	29.56	106.55	2	3	3	3	4	5	2	7	3	5	3	3	4	2	4	3	9	4	6	5	4	4	3	4
B47	2011	Zhang, D. *et al.* & 23183314	BSP2	Chongqing	29.56	106.55	2	3	3	3	4	5	2	7	3	5	3	3	4	2	4	5	7	3	6	6	4	4	3	4
B48	2011	Zhang, D. *et al.* & 23183314	BSP2	Chongqing	29.56	106.55	2	3	3	3	4	5	2	7	3	5	3	3	4	2	4	6	7	3	6	4	4	4	3	4
B49	2011	Zhang, D. *et al.* & 23183314	BSP2	Chongqing	29.56	106.55	2	3	3	3	4	5	2	7	3	5	3	3	4	2	3	6	9	3	4	5	4	4	3	4
B50	2011	Zhang, D. *et al.* & 23183314	BSP2	Chongqing	29.56	106.55	2	3	2	3	4	5	2	5	2	3	3	5	4	1	4	4	9	3	4	4	4	4	3	4
B51	2011	Zhang, D. *et al.* & 23183314	BSP2	Chongqing	29.56	106.55	2	3	3	3	4	5	2	7	3	5	3	3	4	2	4	6	9	3	4	5	4	4	3	4
B52	2011	Zhang, D. *et al.* & 23183314	BSP2	Chongqing	29.56	106.55	2	3	3	3	4	5	2	7	3	5	3	3	4	2	4	6	9	5	5	5	4	4	3	4
B53	2011	Zhang, D. *et al.* & 23183314	BSP2	Chongqing	29.56	106.55	2	3	3	3	4	5	2	7	3	4	3	3	4	2	4	3	9	3	3	3	4	4	3	4
B54	2011	Zhang, D. *et al.* & 23183314	BSP2	Chongqing	29.56	106.55	2	3	3	3	4	5	2	8	3	5	3	4	4	2	4	6	9	3	5	5	4	4	3	4
B55	2011	Zhang, D. *et al.* & 23183314	BSP2	Chongqing	29.56	106.55	2	3	2	2	4	5	2	7	3	5	3	3	4	2	3	4	8	5	5	4	4	4	3	4
B56	2011	Zhang, D. *et al.* & 23183314	BSP2	Chongqing	29.56	106.55	2	3	3	3	4	5	2	7	3	5	3	3	4	2	4	6	6	3	5	4	4	4	3	4
B57	2011	Zhang, D. *et al.* & 23183314	BSP2	Chongqing	29.56	106.55	2	3	3	3	4	5	2	7	3	5	3	3	4	2	4	6	9	4	5	5	4	4	3	5
B58	2011	Zhang, D. *et al.* & 23183314	BSP2	Chongqing	29.56	106.55	2	3	2	3	4	5	2	7	3	5	3	1	4	2	4	6	8	3	4	4	4	4	3	5
B59	2011	Zhang, D. *et al.* & 23183314	BSP2	Chongqing	29.56	106.55	2	3	3	3	4	5	2	7	3	5	3	3	4	2	4	5	8	3	4	4	4	4	3	4
B60	2011	Zhang, D. *et al.* & 23183314	BSP2	Chongqing	29.56	106.55	2	3	1	3	4	2	2	7	3	5	3	3	4	2	4	7	9	5	4	4	4	4	3	4
B61	2011	Zhang, D. *et al.* & 23183314	BSP2	Chongqing	29.56	106.55	2	3	3	3	4	5	2	7	3	5	3	3	4	2	4	6	9	3	4	5	4	4	3	4
B62	2011	Zhang, D. *et al.* & 23183314	BSP2	Chongqing	29.56	106.55	2	3	3	3	4	5	2	6	3	5	3	3	4	2	4	6	9	3	4	4	4	4	3	4
B63	2011	Zhang, D. *et al.* & 23183314	BSP2	Chongqing	29.56	106.55	2	3	3	3	4	5	2	5	3	3	2	3	4	1	4	1	9	3	3	3	4	4	3	4
B64	2011	Zhang, D. *et al.* & 23183314	BSP2	Chongqing	29.56	106.55	2	3	2	3	4	5	2	7	3	4	3	4	4	2	4	6	9	3	4	4	4	4	3	4
B65	2011	Zhang, D. *et al.* & 23183314	BSP2	Chongqing	29.56	106.55	2	3	3	3	4	5	2	4	2	5	3	3	4	2	4	6	8	3	4	4	4	4	3	4
B66	2011	Zhang, D. *et al.* & 23183314	BSP2	Chongqing	29.56	106.55	2	3	3	3	4	5	2	7	3	5	3	3	4	2	4	6	9	5	6	4	4	4	3	4
B67	2011	Zhang, D. *et al.* & 23183314	BSP2	Chongqing	29.56	106.55	2	2	3	3	4	8	2	5	3	3	2	3	4	1	4	6	9	3	4	3	3	4	3	4
B68	2011	Zhang, D. *et al.* & 23183314	BSP2	Chongqing	29.56	106.55	2	3	3	2	4	5	2	5	3	3	2	3	4	1	4	6	9	3	4	3	4	4	3	4
B69	2011	Zhang, D. *et al.* & 23183314	BSP2	Chongqing	29.56	106.55	2	3	3	3	4	5	2	7	3	5	1	3	4	2	4	6	9	5	4	4	4	4	3	4
B70	2011	Zhang, D. *et al.* & 23183314	BSP2	Chongqing	29.56	106.55	2	3	3	3	3	5	2	4	3	3	2	2	4	1	4	6	9	3	4	3	4	2	3	4
B71	2011	Zhang, D. *et al.* & 23183314	BSP2	Chongqing	29.56	106.55	2	3	3	3	3	5	2	7	3	5	3	3	4	2	4	6	7	3	4	4	4	4	3	4
B72	2011	Zhang, D. *et al.* & 23183314	BSP2	Chongqing	29.56	106.55	2	3	2	3	4	5	2	5	3	4	3	3	2	1	4	6	9	3	4	2	3	4	3	4
B73	2011	Zhang, D. *et al.* & 23183314	BSP2	Chongqing	29.56	106.55	2	3	3	4	4	5	2	7	3	5	3	3	4	2	4	6	9	3	4	5	4	4	3	4
B74	2011	Zhang, D. *et al.* & 23183314	BSP2	Chongqing	29.56	106.55	2	3	3	3	4	5	2	5	3	3	2	1	4	1	4	3	9	3	4	3	4	2	3	4
B75	2011	Zhang, D. *et al.* & 23183314	BSP2	Chongqing	29.56	106.55	2	3	3	3	3	5	2	6	3	5	3	3	4	2	4	3	9	5	4	4	4	4	3	4
B76	2011	Zhang, D. *et al.* & 23183314	BSP2	Chongqing	29.56	106.55	2	3	3	3	4	5	2	7	3	5	3	3	4	2	4	5	5	4	4	4	4	4	3	5
B77	2011	Zhang, D. *et al.* & 23183314	BSP2	Chongqing	29.56	106.55	2	3	2	3	4	5	2	7	3	5	3	3	4	2	4	5	9	3	4	6	4	4	3	4
B78	2011	Zhang, D. *et al.* & 23183314	BSP2	Chongqing	29.56	106.55	2	3	3	3	4	5	2	7	3	5	3	3	4	2	4	6	9	3	4	4	4	4	3	3
B79	2011	Zhang, D. *et al.* & 23183314	BSP2	Chongqing	29.56	106.55	2	2	3	3	4	5	2	5	3	3	2	3	4	1	5	1	8	3	4	3	4	4	3	4

B80	2011	Zhang, D. *et al. &* 23183314	BSP2	Chongqing	29.56	106.55	2	3	3	3	4	5	2	7	3	4	3	3	4	2	4	1	9	3	4	5	4	4	3	4
B81	2011	Zhang, D. *et al. &* 23183314	BSP2	Chongqing	29.56	106.55	2	3	3	3	4	5	2	7	3	5	3	3	2	2	4	6	8	5	4	4	4	4	3	5
B82	2011	Zhang, D. *et al. &* 23183314	BSP2	Chongqing	29.56	106.55	2	3	3	3	4	5	2	5	3	4	2	3	4	1	4	3	6	3	4	3	4	4	3	4
B83	2011	Zhang, D. *et al. &* 23183314	BSP2	Chongqing	29.56	106.55	2	3	2	3	4	5	2	5	3	3	2	3	4	1	2	3	6	3	4	3	4	4	2	4
B84	2011	Zhang, D. *et al. &* 23183314	BSP2	Chongqing	29.56	106.55	2	3	3	3	4	5	2	7	3	4	3	3	4	2	4	7	9	4	4	4	4	4	3	5
B85	2011	Zhang, D. *et al. &* 23183314	BSP2	Chongqing	29.56	106.55	2	3	3	3	4	5	2	5	4	3	3	3	4	2	4	3	9	3	4	3	4	2	3	4
B86	2011	Zhang, D. *et al. &* 23183314	BSP2	Chongqing	29.56	106.55	2	3	3	3	4	5	2	7	3	5	3	3	4	2	4	5	9	5	4	4	4	4	3	4
B87	2011	Zhang, D. *et al. &* 23183314	BSP2	Chongqing	29.56	106.55	2	3	3	3	4	5	2	7	1	5	3	3	4	2	4	6	7	5	4	4	4	4	3	4
B88	2011	Zhang, D. *et al. &* 23183314	BSP2	Chongqing	29.56	106.55	2	3	3	3	4	5	2	7	3	5	3	3	4	2	4	6	7	3	4	4	4	4	3	4
B89	2011	Zhang, D. *et al. &* 23183314	BSP2	Chongqing	29.56	106.55	2	3	3	3	4	5	2	7	3	5	3	3	4	2	4	4	7	3	4	4	4	4	3	4
B90	2011	Zhang, D. *et al. &* 23183314	BSP2	Chongqing	29.56	106.55	2	3	3	3	4	5	2	7	3	4	3	3	1	2	4	4	9	3	4	5	4	4	3	4
B91	2011	Zhang, D. *et al. &* 23183314	BSP2	Chongqing	29.56	106.55	2	3	3	2	4	5	2	7	3	5	3	3	4	2	4	4	9	3	4	4	4	4	3	4
B92	2011	Zhang, D. *et al. &* 23183314	BSP2	Chongqing	29.56	106.55	2	3	3	3	4	5	2	7	3	5	3	3	4	2	4	6	9	3	4	5	4	4	3	4
B93	2011	Zhang, D. *et al. &* 23183314	BSP2	Chongqing	29.56	106.55	2	3	3	3	4	5	2	5	3	3	3	3	4	1	4	3	6	3	4	4	4	4	3	4
B94	2011	Zhang, D. *et al. &* 23183314	BSP1	Chongqing	29.56	106.55	2	5	2	1	4	5	2	5	2	3	2	2	3	2	4	4	6	3	3	1	4	2	3	2
B95	2011	Zhang, D. *et al. &* 23183314	BSP2	Chongqing	29.56	106.55	2	3	3	3	4	5	2	6	3	5	3	3	4	2	4	5	9	3	4	4	4	4	3	4
B96	2011	Zhang, D. *et al. &* 23183314	BSP2	Chongqing	29.56	106.55	2	3	3	4	4	5	2	7	3	5	3	4	4	2	4	6	6	5	4	4	4	4	3	4
B97	2011	Zhang, D. *et al. &* 23183314	BSP2	Chongqing	29.56	106.55	2	3	3	3	4	5	2	4	3	3	3	3	4	1	4	4	6	3	4	3	4	2	3	4
B98	2011	Zhang, D. *et al. &* 23183314	BSP2	Chongqing	29.56	106.55	2	3	3	3	4	5	2	7	3	5	3	3	4	2	4	3	8	4	4	6	4	4	3	4
B99	2011	Zhang, D. *et al. &* 23183314	BSP2	Chongqing	29.56	106.55	2	3	3	3	4	5	2	5	3	3	3	3	4	1	4	3	6	3	4	3	4	4	3	4
B100	2011	Zhang, D. *et al. &* 23183314	BSP2	Chongqing	29.56	106.55	2	3	3	3	4	5	2	7	3	5	3	3	4	2	4	4	7	3	4	4	4	4	3	4
B101	2011	Zhang, D. *et al. &* 23183314	BSP2	Chongqing	29.56	106.55	2	3	3	3	4	5	2	7	3	5	3	3	4	2	4	4	7	3	4	4	4	4	3	4
B102	2011	Zhang, D. *et al. &* 23183314	BSP2	Chongqing	29.56	106.55	2	3	3	3	4	5	2	7	3	5	3	3	4	2	4	6	8	3	4	4	4	4	3	5
B103	2011	Zhang, D. *et al. &* 23183314	BSP2	Chongqing	29.56	106.55	2	3	3	3	4	5	2	7	3	2	3	3	4	2	4	4	9	4	5	4	4	4	3	4
B104	2011	Zhang, D. *et al. &* 23183314	BSP2	Chongqing	29.56	106.55	2	3	3	3	4	5	2	7	3	5	3	3	4	2	4	6	6	3	4	4	4	4	3	4
B105	2011	Zhang, D. *et al. &* 23183314	BSP2	Chongqing	29.56	106.55	2	3	3	3	4	5	2	7	3	5	3	3	4	2	4	6	9	3	4	4	4	4	3	4
B106	2011	Zhang, D. *et al. &* 23183314	BSP2	Chongqing	29.56	106.55	2	3	3	3	4	5	2	5	3	3	2	3	4	1	2	3	6	3	3	3	4	4	3	4
B107	2011	Zhang, D. *et al. &* 23183314	BSP2	Chongqing	29.56	106.55	2	3	2	3	4	6	2	7	3	3	2	2	4	2	4	4	9	4	4	4	4	4	3	3
B108	2011	Zhang, D. *et al. &* 23183314	BSP2	Chongqing	29.56	106.55	2	3	3	3	4	5	2	7	3	5	3	3	4	2	4	6	9	3	4	5	4	4	3	4
B109	2011	Zhang, D. *et al. &* 23183314	BSP2	Chongqing	29.56	106.55	2	3	3	3	4	5	2	5	3	5	3	3	4	2	4	6	7	4	5	4	4	4	3	4
B110	2011	Zhang, D. *et al. &* 23183314	BSP2	Chongqing	29.56	106.55	2	3	2	3	4	6	2	7	3	5	3	3	4	2	4	4	9	3	4	5	4	4	3	4
B111	2011	Zhang, D. *et al. &* 23183314	BSP2	Chongqing	29.56	106.55	2	3	3	2	4	5	2	7	3	5	3	3	4	2	4	7	9	5	4	4	4	4	3	4
B112	2011	Zhang, D. *et al. &* 23183314	BSP2	Chongqing	29.56	106.55	2	3	3	3	4	5	2	7	3	5	3	3	4	2	4	6	7	3	4	4	4	4	3	5
B113	2011	Zhang, D. *et al. &* 23183314	BSP2	Chongqing	29.56	106.55	2	3	2	3	4	5	2	4	3	3	3	3	4	1	4	4	9	3	4	3	4	2	3	4
B114	2011	Zhang, D. *et al. &* 23183314	BSP2	Chongqing	29.56	106.55	2	3	3	2	4	5	2	7	3	5	3	3	4	2	4	5	9	3	4	4	4	4	3	4
B115	2011	Zhang, D. *et al. &* 23183314	BSP2	Chongqing	29.56	106.55	2	3	1	3	4	5	2	5	3	5	3	3	4	2	4	6	9	3	4	4	4	4	3	4
B116	2011	Zhang, D. *et al. &* 23183314	BSP2	Chongqing	29.56	106.55	2	3	2	2	4	5	2	5	3	3	2	3	4	1	4	3	5	3	4	3	4	4	3	4
B117	2011	Zhang, D. *et al. &* 23183314	BSP2	Chongqing	29.56	106.55	2	3	3	3	4	4	2	5	3	3	4	1	4	2	4	5	9	4	5	4	4	4	3	4
B118	2011	Zhang, D. *et al. &* 23183314	BSP2	Chongqing	29.56	106.55	2	3	3	3	4	5	2	7	3	5	3	3	4	2	4	6	ND	3	6	4	4	4	3	4
B119	2011	Zhang, D. *et al. &* 23183314	BSP2	Chongqing	29.56	106.55	2	3	1	3	4	5	2	6	3	5	3	3	4	2	4	6	9	5	6	4	4	4	3	4
B120	2011	Zhang, D. *et al. &* 23183314	BSP2	Chongqing	29.56	106.55	2	3	3	3	4	5	2	7	3	5	3	3	4	2	4	6	ND	3	6	4	4	4	3	4

B121	2011	Zhang, D. *et al.* & 23183314	BSP2	Chongqing	29.56	106.55	2	3	3	3	4	5	2	7	3	5	3	3	4	2	4	6	ND	3	6	4	4	4	3	4
B122	2011	Zhang, D. *et al.* & 23183314	BSP2	Chongqing	29.56	106.55	1	3	3	3	4	5	2	7	3	5	3	3	4	2	4	6	ND	3	6	4	4	4	3	4
B123	2011	Zhang, D. *et al.* & 23183314	BSP2	Chongqing	29.56	106.55	2	3	2	3	4	5	2	4	3	3	3	3	4	1	3	2	7	3	4	3	4	4	3	3
B124	2011	Zhang, D. *et al.* & 23183314	BSP2	Chongqing	29.56	106.55	2	3	2	3	4	5	2	5	3	4	2	3	4	1	2	3	5	3	4	3	4	4	3	4
B125	2011	Zhang, D. *et al.* & 23183314	BSP2	Chongqing	29.56	106.55	2	3	3	3	4	5	2	6	3	6	3	3	4	2	4	6	9	4	4	4	4	4	3	4
B126	2011	Zhang, D. *et al.* & 23183314	BSP2	Chongqing	29.56	106.55	2	3	3	2	4	5	2	7	3	5	3	3	4	2	4	5	9	3	5	5	4	4	2	4
B127	2011	Zhang, D. *et al.* & 23183314	BSP2	Chongqing	29.56	106.55	2	3	3	3	4	5	2	7	3	5	3	3	4	2	4	6	ND	3	6	4	4	4	3	4
B128	2011	Zhang, D. *et al.* & 23183314	BSP2	Chongqing	29.56	106.55	2	3	3	4	4	5	2	7	3	5	3	3	4	2	4	7	8	5	5	4	4	4	3	4
B129	2011	Zhang, D. *et al.* & 23183314	BSP2	Chongqing	29.56	106.55	2	3	3	3	4	5	2	7	2	5	3	3	4	2	4	6	8	3	5	4	4	4	3	4
B130	2011	Zhang, D. *et al.* & 23183314	BSP2	Chongqing	29.56	106.55	2	3	3	3	4	5	2	4	3	4	3	3	4	1	2	3	5	3	4	4	4	4	3	4
B131	2011	Zhang, D. *et al.* & 23183314	BSP2	Chongqing	29.56	106.55	2	3	3	3	4	5	2	7	3	5	3	3	4	2	4	4	7	3	4	4	4	4	3	4
B132	2011	Zhang, D. *et al.* & 23183314	BSP2	Chongqing	29.56	106.55	2	1	3	3	4	5	2	7	3	5	3	3	4	2	4	4	9	3	5	3	4	4	3	4
B133	2011	Zhang, D. *et al.* & 23183314	BSP2	Chongqing	29.56	106.55	2	3	3	3	4	5	2	5	3	3	3	3	4	2	4	2	8	3	4	3	4	4	3	4
B134	2011	Zhang, D. *et al.* & 23183314	BSP2	Chongqing	29.56	106.55	2	3	3	3	4	5	2	7	3	5	3	3	4	2	4	6	9	3	5	5	4	4	2	4
B135	2011	Zhang, D. *et al.* & 23183314	BSP2	Chongqing	29.56	106.55	2	3	3	3	4	5	2	7	2	5	3	3	2	2	4	6	9	3	6	4	4	4	3	4
B136	2011	Zhang, D. *et al.* & 23183314	BSP2	Chongqing	29.56	106.55	2	3	3	3	4	5	2	7	3	5	3	3	4	2	4	5	9	3	6	5	4	4	3	4
B137	2011	Zhang, D. *et al.* & 23183314	BSP2	Chongqing	29.56	106.55	2	2	3	3	4	5	2	7	3	5	3	3	4	2	4	5	8	5	6	4	4	4	3	4
B138	2011	Zhang, D. *et al.* & 23183314	BSP2	Chongqing	29.56	106.55	2	3	2	3	4	5	2	4	3	3	3	3	4	2	4	4	9	3	4	4	4	2	3	4
B139	2011	Zhang, D. *et al.* & 23183314	BSP2	Chongqing	29.56	106.55	2	3	3	3	4	5	2	5	3	3	3	3	4	2	4	4	9	3	4	4	2	4	3	4
B140	2011	Zhang, D. *et al.* & 23183314	BSP2	Chongqing	29.56	106.55	2	3	3	3	4	5	2	5	3	5	3	3	4	2	4	6	ND	3	4	4	4	4	3	4
B141	2011	Zhang, D. *et al.* & 23183314	BSP2	Chongqing	29.56	106.55	2	3	3	3	4	5	2	7	3	5	3	3	4	2	4	6	9	3	4	5	4	4	3	4
B142	2011	Zhang, D. *et al.* & 23183314	BSP2	Chongqing	29.56	106.55	2	3	3	3	4	5	2	5	3	5	4	3	4	2	3	6	6	3	4	4	4	2	3	4
B143	2011	Zhang, D. *et al.* & 23183314	BSP2	Chongqing	29.56	106.55	2	4	3	3	4	5	2	5	2	3	3	1	2	1	4	3	ND	3	4	4	4	2	3	4
B144	2011	Zhang, D. *et al.* & 23183314	BSP2	Chongqing	29.56	106.55	2	3	3	3	4	5	2	7	3	5	3	3	4	2	4	6	4	3	4	4	4	4	3	4
B145	2011	Zhang, D. *et al.* & 23183314	BSP2	Chongqing	29.56	106.55	2	3	3	3	4	5	2	7	3	5	3	3	4	2	4	7	9	5	6	4	4	4	3	5
B146	2011	Zhang, D. *et al.* & 23183314	BSP2	Chongqing	29.56	106.55	2	4	2	3	4	5	2	5	3	3	3	3	1	2	4	4	9	3	4	4	4	4	2	4
B147	2011	Zhang, D. *et al.* & 23183314	BSP2	Chongqing	29.56	106.55	2	3	3	3	4	5	2	7	3	5	3	3	4	2	4	6	9	5	6	4	4	4	3	4
B148	2011	Zhang, D. *et al.* & 23183314	BSP2	Chongqing	29.56	106.55	2	3	3	3	4	5	2	7	3	3	3	3	4	2	4	5	9	3	6	5	4	4	3	4
B149	2011	Zhang, D. *et al.* & 23183314	BSP2	Chongqing	29.56	106.55	2	3	3	3	4	5	2	7	3	5	3	3	4	2	4	6	9	5	6	4	4	4	3	4
B150	2011	Zhang, D. *et al.* & 23183314	BSP2	Chongqing	29.56	106.55	2	3	3	3	4	5	2	7	3	5	3	3	4	2	4	6	9	5	6	4	4	4	3	4
B151	2011	Zhang, D. *et al.* & 23183314	BSP2	Chongqing	29.56	106.55	2	3	3	3	4	5	2	7	3	5	3	3	4	2	4	6	ND	3	6	4	4	4	3	4
B152	2011	Zhang, D. *et al.* & 23183314	BSP2	Chongqing	29.56	106.55	2	3	3	3	4	5	2	7	3	5	3	3	4	2	4	6	ND	3	6	4	4	4	3	4
B153	2011	Zhang, D. *et al.* & 23183314	BSP2	Chongqing	29.56	106.55	2	3	3	3	4	5	2	7	3	5	3	3	4	2	4	5	9	3	6	5	4	4	3	4
B154	2011	Zhang, D. *et al.* & 23183314	BSP2	Chongqing	29.56	106.55	2	3	3	3	4	5	2	7	3	5	3	3	4	2	4	6	9	3	4	5	4	4	3	4
B155	2011	Zhang, D. *et al.* & 23183314	BSP2	Chongqing	29.56	106.55	2	3	3	3	4	5	2	7	3	5	3	3	4	2	4	5	9	3	6	5	4	4	3	4
B156	2011	Zhang, D. *et al.* & 23183314	BSPint	Chongqing	29.56	106.55	2	4	2	3	4	5	2	4	2	2	3	2	3	2	4	1	6	3	4	1	4	2	3	2
B157	2011	Zhang, D. *et al.* & 23183314	BSP2	Chongqing	29.56	106.55	2	3	3	3	4	5	2	7	3	5	3	3	4	2	4	4	9	3	6	4	4	4	3	4
B158	2011	Zhang, D. *et al.* & 23183314	BSP2	Chongqing	29.56	106.55	2	3	3	3	4	5	2	7	3	5	3	3	4	2	4	5	9	3	6	5	4	4	3	4
B159	2011	Zhang, D. *et al.* & 23183314	BSP2	Chongqing	29.56	106.55	2	3	2	3	4	5	2	5	3	3	3	4	4	2	4	3	6	3	4	3	4	4	3	4
B160	2011	Zhang, D. *et al.* & 23183314	BSP2	Chongqing	29.56	106.55	2	3	3	3	4	2	2	7	3	5	3	3	4	2	4	4	9	3	6	4	4	4	3	4
B161	2011	Zhang, D. *et al.* & 23183314	BSP2	Chongqing	29.56	106.55	2	3	3	3	4	2	2	7	3	5	3	3	4	2	4	4	9	3	6	4	4	4	3	4

B162	2011	Zhang, D.<br>*et al.* &<br>23183314	BSP2	Chongqing	29.56	106.55	2	3	3	3	4	5	2	5	3	3	3	3	4	2	4	4	ND	3	4	4	4	2	3	4
B163	2011	Zhang, D.<br>*et al.* &<br>23183314	BSP2	Chongqing	29.56	106.55	2	3	3	3	3	5	2	5	3	3	2	3	4	1	4	2	8	3	4	3	4	4	3	4
B164	2011	Zhang, D.<br>*et al.* &<br>23183314	BSP2	Chongqing	29.56	106.55	2	5	3	3	4	5	2	5	2	3	3	2	3	2	4	6	5	3	4	1	4	2	2	2
B165	2011	Zhang, D.<br>*et al.* &<br>23183314	BSP2	Chongqing	29.56	106.55	2	3	3	3	4	5	2	7	3	5	3	3	4	2	4	6	9	5	4	4	4	4	3	4
B166	2011	Zhang, D.<br>*et al.* &<br>23183314	BSP2	Chongqing	29.56	106.55	2	3	3	3	4	5	2	5	3	3	3	3	4	2	4	4	9	3	4	3	4	4	2	4
B167	2011	Zhang, D.<br>*et al.* &<br>23183314	BSP2	Chongqing	29.56	106.55	2	3	2	3	4	5	2	7	3	5	4	4	4	2	4	6	9	3	6	4	4	4	3	4
B168	2011	Zhang, D.<br>*et al.* &<br>23183314	BSP2	Chongqing	29.56	106.55	2	3	3	3	4	5	2	7	3	5	3	3	4	2	4	6	ND	3	6	4	4	4	3	4
B169	2011	Zhang, D.<br>*et al.* &<br>23183314	BSP2	Chongqing	29.56	106.55	2	3	3	3	4	5	2	7	3	5	4	3	4	2	4	7	9	5	6	4	4	4	3	4
B170	2011	Zhang, D.<br>*et al.* &<br>23183314	BSP2	Chongqing	29.56	106.55	2	3	3	3	3	6	2	4	3	3	2	1	4	1	4	3	8	3	4	2	4	2	3	3
B171	2011	Zhang, D.<br>*et al.* &<br>23183314	BSP2	Chongqing	29.56	106.55	2	3	2	3	4	5	2	7	3	4	3	1	4	2	4	6	9	3	4	5	4	4	3	4
B172	2011	Zhang, D.<br>*et al.* &<br>23183314	BSP2	Chongqing	29.56	106.55	2	3	3	3	4	5	2	7	3	5	3	3	4	2	4	6	9	3	4	5	4	4	3	4
B173	2011	Zhang, D.<br>*et al.* &<br>23183314	BSP2	Chongqing	29.56	106.55	2	3	2	3	4	5	2	5	3	3	1	2	3	2	4	3	ND	3	4	2	4	2	3	4
B174	2011	Zhang, D.<br>*et al.* &<br>23183314	BSP2	Chongqing	29.56	106.55	2	3	3	3	4	5	2	7	3	5	3	3	4	2	4	2	8	5	6	4	4	4	3	4
B175	2011	Zhang, D.<br>*et al.* &<br>23183314	BSP2	Chongqing	29.56	106.55	2	3	3	2	4	5	2	7	3	5	1	3	4	2	4	5	9	3	6	5	4	4	3	4
B176	2011	Zhang, D.<br>*et al.* &<br>23183314	BSP2	Chongqing	29.56	106.55	2	3	3	3	3	5	2	3	3	3	3	3	4	2	4	5	8	3	4	3	4	4	3	4
B177	2011	Zhang, D.<br>*et al.* &<br>23183314	BSP2	Chongqing	29.56	106.55	2	3	3	2	4	5	2	7	3	5	3	3	4	2	4	5	4	3	4	4	4	4	3	3
B178	2011	Zhang, D.<br>*et al.* &<br>23183314	BSP2	Chongqing	29.56	106.55	2	3	3	3	4	5	2	7	3	5	3	3	4	2	4	6	9	3	4	5	4	4	3	4
B179	2011	Zhang, D.<br>*et al.* &<br>23183314	BSP2	Chongqing	29.56	106.55	2	3	3	3	4	5	2	7	3	5	3	3	4	2	4	6	9	3	4	5	4	4	3	4
B180	2011	Zhang, D.<br>*et al.* &<br>23183314	BSP2	Chongqing	29.56	106.55	2	3	3	3	4	5	2	7	3	5	3	3	3	2	4	6	9	5	6	4	4	4	2	4
B181	2011	Zhang, D.<br>*et al.* &<br>23183314	BSP2	Chongqing	29.56	106.55	2	3	3	3	4	5	2	7	3	5	3	3	4	2	4	6	ND	3	6	4	4	4	3	4
B182	2011	Zhang, D.<br>*et al.* &<br>23183314	BSP2	Chongqing	29.56	106.55	2	3	3	3	4	5	2	3	3	5	3	1	4	2	2	6	9	5	4	4	4	4	2	4
B183	2011	Zhang, D.<br>*et al.* &<br>23183314	BSP2	Chongqing	29.56	106.55	2	3	3	3	4	5	2	7	3	5	3	4	4	2	4	6	8	5	4	4	4	4	3	4
B184	2011	Zhang, D.<br>*et al.* &<br>23183314	BSP2	Chongqing	29.56	106.55	2	3	3	2	4	5	2	5	3	4	2	3	4	2	4	3	9	3	4	3	4	4	3	3
B185	2011	Zhang, D.<br>*et al.* &<br>23183314	BSP2	Chongqing	29.56	106.55	2	3	3	3	3	5	2	7	3	5	3	3	4	2	2	3	9	3	4	5	4	4	3	4
B186	2011	Zhang, D.<br>*et al.* &<br>23183314	BSPint	Chongqing	29.56	106.55	2	5	2	3	4	5	2	5	2	3	3	2	3	2	4	3	4	3	4	1	4	2	3	2
B187	2011	Zhang, D.<br>*et al.* &<br>23183314	BSP2	Chongqing	29.56	106.55	2	3	3	3	4	5	2	7	3	5	3	3	4	2	4	6	9	3	4	2	4	4	3	4
B188	2011	Zhang, D.<br>*et al.* &<br>23183314	BSP2	Chongqing	29.56	106.55	2	3	3	3	4	5	2	4	3	5	4	3	4	2	4	6	7	3	4	4	3	4	3	4
B189	2011	Zhang, D.<br>*et al.* &<br>23183314	BSP1	Chongqing	29.56	106.55	2	5	2	3	3	5	2	7	2	3	2	2	4	2	4	3	6	3	4	1	3	2	3	2
B190	2011	Zhang, D.<br>*et al.* &<br>23183314	BSPint	Chongqing	29.56	106.55	2	5	2	3	4	5	2	5	2	3	3	2	4	2	4	3	4	3	4	1	4	2	3	2
B191	2011	Zhang, D.<br>*et al.* &<br>23183314	BSP2	Chongqing	29.56	106.55	2	3	2	3	4	5	2	7	3	3	3	3	4	2	4	1	9	3	4	3	4	2	3	4
B192	2011	Zhang, D.<br>*et al.* &<br>23183314	BSP2	Chongqing	29.56	106.55	2	3	2	3	4	5	2	6	3	3	3	3	4	2	4	2	9	3	4	3	4	4	3	4
B193	2011	Zhang, D.<br>*et al.* &<br>23183314	BSP2	Chongqing	29.56	106.55	2	3	3	3	4	6	2	7	3	5	3	3	4	2	4	6	ND	3	6	4	4	4	3	4
B194	2011	Zhang, D.<br>*et al.* &<br>23183314	BSP2	Chongqing	29.56	106.55	2	3	3	3	4	5	2	4	3	3	2	3	4	2	4	3	9	3	4	3	4	4	2	3
B195	2011	Zhang, D.<br>*et al.* &<br>23183314	BSP2	Chongqing	29.56	106.55	2	3	3	3	4	5	2	7	3	5	3	3	4	2	4	6	6	3	4	4	4	4	3	4
B196	2011	Zhang, D.<br>*et al.* &<br>23183314	BSP2	Chongqing	29.56	106.55	2	3	3	3	4	5	2	6	3	5	3	3	4	2	4	6	9	3	4	4	4	4	3	4
B197	2011	Zhang, D.<br>*et al.* &<br>23183314	BSP2	Chongqing	29.56	106.55	2	3	3	3	4	5	2	7	3	5	3	3	4	2	4	5	9	3	6	5	4	4	3	4
B198	2011	Zhang, D.<br>*et al.* &<br>23183314	BSP2	Chongqing	29.56	106.55	2	3	3	3	4	5	2	4	3	3	3	3	4	3	4	5	8	3	4	2	4	4	3	4
B199	2011	Zhang, D.<br>*et al.* &<br>23183314	BSP2	Chongqing	29.56	106.55	2	3	3	3	4	5	2	7	3	6	3	3	4	2	3	3	8	5	4	4	4	4	3	4
B200	2003-<br>2007	Chen, Y.<br>*et al.* &<br>22808061	BSP4	Taiwan	23.70	120.96	2	2	3	3	2	5	1	4	3	5	3	3	4	2	4	4	9	4	3	4	4	4	3	3
B201	2003-<br>2007	Chen, Y.<br>*et al.* &<br>22808061	BSP4	Taiwan	23.70	120.96	2	2	3	3	2	5	1	7	3	5	2	3	4	2	4	3	7	3	3	4	4	4	3	3
B202	2003-<br>2007	Chen, Y.<br>*et al.* &<br>22808061	BSP4	Taiwan	23.70	120.96	2	2	3	3	2	5	1	7	3	5	2	3	4	2	4	4	8	3	3	4	4	4	3	3

B203	2003-2007	Chen, Y. *et al.* & 22808061	BSP4	Taiwan	23.70	120.96	2	2	3	3	2	5	1	7	3	5	2	3	4	2	4	3	7	3	3	4	4	4	3	3
B204	2003-2007	Chen, Y. *et al.* & 22808061	BSPint	Taiwan	23.70	120.96	2	2	3	3	2	5	1	6	3	4	3	3	4	2	4	5	9	3	3	4	4	4	3	3
B205	2003-2007	Chen, Y. *et al.* & 22808061	BSPint	Taiwan	23.70	120.96	2	2	3	3	2	5	1	7	3	5	3	3	4	2	4	5	9	3	3	4	4	4	3	5
B206	2003-2007	Chen, Y. *et al.* & 22808061	BSPint	Taiwan	23.70	120.96	2	2	3	3	2	5	1	5	3	5	1	1	4	2	4	6	ND	3	3	4	4	4	3	3
B207	2003-2007	Chen, Y. *et al.* & 22808061	BSPint	Taiwan	23.70	120.96	2	2	3	3	2	5	1	6	3	4	3	3	4	2	4	5	9	3	3	4	4	4	3	3
B208	2003-2007	Chen, Y. *et al.* & 22808061	BSP4	Taiwan	23.70	120.96	2	2	3	3	2	5	1	6	3	5	3	3	4	2	4	6	7	3	3	4	4	2	3	3
B209	2003-2007	Chen, Y. *et al.* & 22808061	BSP4	Taiwan	23.70	120.96	2	2	3	3	2	5	1	6	3	5	3	3	4	2	4	5	5	3	3	4	4	4	3	3
B210	2003-2007	Chen, Y. *et al.* & 22808061	BSPint	Taiwan	23.70	120.96	2	2	3	3	2	5	1	7	3	5	3	3	4	2	4	5	9	3	3	4	4	4	3	5
B211	2003-2007	Chen, Y. *et al.* & 22808061	BSPint	Taiwan	23.70	120.96	2	2	3	3	2	5	1	7	3	5	3	3	4	2	4	5	9	3	3	4	4	4	3	5
B212	2003-2007	Chen, Y. *et al.* & 22808061	BSPint	Taiwan	23.70	120.96	2	2	3	3	2	5	1	7	3	5	3	3	4	2	4	5	9	3	3	4	4	4	3	5
B213	2003-2007	Chen, Y. *et al.* & 22808061	BSPint	Taiwan	23.70	120.96	2	2	3	3	2	5	1	7	3	5	3	3	4	2	4	5	9	3	3	4	4	4	3	5
B214	2003-2007	Chen, Y. *et al.* & 22808061	BSPint	Taiwan	23.70	120.96	2	2	3	3	2	5	1	5	3	5	3	1	4	2	4	5	8	3	3	4	4	4	3	7
B215	2003-2007	Chen, Y. *et al.* & 22808061	BSP1	Taiwan	23.70	120.96	2	2	3	2	2	5	1	7	3	4	3	2	4	2	4	3	9	3	3	5	4	4	3	3
B216	2003-2007	Chen, Y. *et al.* & 22808061	BSPint	Taiwan	23.70	120.96	2	2	3	3	2	5	1	7	3	2	3	2	4	2	4	6	ND	3	3	3	4	4	3	4
B217	2003-2007	Chen, Y. *et al.* & 22808061	BSPint	Taiwan	23.70	120.96	2	2	3	3	2	5	1	7	3	2	3	2	4	2	4	6	ND	3	3	3	4	4	3	4
B218	2003-2007	Chen, Y. *et al.* & 22808061	BSPint	Taiwan	23.70	120.96	2	2	3	3	2	5	1	7	3	2	3	2	4	2	4	6	ND	3	3	3	4	4	3	4
B219	2003-2007	Chen, Y. *et al.* & 22808061	BSPint	Taiwan	23.70	120.96	2	2	3	3	2	5	1	7	3	2	3	2	4	2	4	6	8	3	3	3	4	4	3	4
B220	2003-2007	Chen, Y. *et al.* & 22808061	BSPint	Taiwan	23.70	120.96	2	2	3	3	2	5	1	7	3	2	3	2	4	2	4	6	ND	3	3	3	4	4	3	4
B221	2003-2007	Chen, Y. *et al.* & 22808061	BSPint	Taiwan	23.70	120.96	2	2	3	3	2	5	1	7	3	2	3	2	4	2	4	6	ND	3	3	3	4	4	3	4
B222	2003-2007	Chen, Y. *et al.* & 22808061	BSP1	Taiwan	23.70	120.96	2	2	4	3	2	5	1	7	3	2	3	2	4	2	4	6	ND	3	3	3	4	4	3	4
B223	2003-2007	Chen, Y. *et al.* & 22808061	BSPint	Taiwan	23.70	120.96	2	2	5	2	2	5	1	3	3	5	3	1	4	2	4	6	9	3	3	4	4	4	3	3
B224	2003-2007	Chen, Y. *et al.* & 22808061	BSP4	Taiwan	23.70	120.96	2	2	3	3	2	5	1	6	3	5	3	1	4	2	4	4	9	3	3	4	4	4	2	3
B225	2003-2007	Chen, Y. *et al.* & 22808061	BSP4	Taiwan	23.70	120.96	2	2	3	3	2	5	1	7	3	5	3	3	4	2	4	4	6	3	3	4	4	4	3	3
B226	2003-2007	Chen, Y. *et al.* & 22808061	BSP4	Taiwan	23.70	120.96	2	2	3	3	2	5	1	7	3	5	3	2	4	2	4	4	8	3	3	4	4	4	3	3
B227	2003-2007	Chen, Y. *et al.* & 22808061	BSP5	Taiwan	23.70	120.96	2	2	3	3	2	6	1	7	3	5	4	3	4	2	4	5	8	2	4	5	4	4	3	3
B228	2003-2007	Chen, Y. *et al.* & 22808061	BSP5	Taiwan	23.70	120.96	2	2	3	3	2	5	1	7	3	5	3	3	3	2	4	5	8	2	4	4	4	4	3	3
B229	2003-2007	Chen, Y. *et al.* & 22808061	BSP5	Taiwan	23.70	120.96	2	2	3	3	2	5	1	7	3	5	2	4	3	2	4	5	8	2	4	4	4	4	3	3
B230	2003-2007	Chen, Y. *et al.* & 22808061	BSP5	Taiwan	23.70	120.96	2	2	3	3	2	5	1	7	3	5	3	3	3	2	4	5	8	2	4	5	4	2	3	3
B231	2003-2007	Chen, Y. *et al.* & 22808061	BSP5	Taiwan	23.70	120.96	2	2	3	3	2	5	1	7	3	5	3	3	3	2	4	5	8	2	4	5	4	2	3	3
B232	2003-2007	Chen, Y. *et al.* & 22808061	BSP5	Taiwan	23.70	120.96	2	2	3	3	2	5	1	7	3	5	3	3	3	2	4	5	8	2	4	5	4	2	3	3
B233	2003-2007	Chen, Y. *et al.* & 22808061	BSP5	Taiwan	23.70	120.96	2	2	3	4	2	5	1	7	3	5	3	3	3	2	4	4	8	2	3	5	4	4	3	4
B234	2003-2007	Chen, Y. *et al.* & 22808061	BSP5	Taiwan	23.70	120.96	2	2	3	3	2	5	1	7	3	5	3	3	4	2	4	5	8	2	4	5	4	4	3	3
B235	2003-2007	Chen, Y. *et al.* & 22808061	BSP5	Taiwan	23.70	120.96	2	0	3	3	2	5	1	1	3	5	3	3	4	2	4	5	8	2	4	5	4	4	3	3
B236	2003-2007	Chen, Y. *et al.* & 22808061	BSP5	Taiwan	23.70	120.96	2	0	3	3	2	5	1	1	3	5	3	3	4	2	4	5	8	2	4	5	4	4	3	3
B237	2003-2007	Chen, Y. *et al.* & 22808061	BSP5	Taiwan	23.70	120.96	2	2	3	3	2	5	1	7	3	5	3	3	3	2	4	5	8	2	4	5	4	4	3	3
B238	2003-2007	Chen, Y. *et al.* & 22808061	BSP5	Taiwan	23.70	120.96	2	2	3	3	2	5	1	7	3	5	3	3	3	2	4	5	8	2	4	5	4	4	3	3
B239	2003-2007	Chen, Y. *et al.* & 22808061	BSP5	Taiwan	23.70	120.96	2	2	3	3	2	5	1	7	3	5	3	3	3	2	4	5	8	2	4	5	4	4	3	3
B240	2003-2007	Chen, Y. *et al.* & 22808061	BSP5	Taiwan	23.70	120.96	2	2	3	3	2	5	1	7	3	5	2	3	3	2	4	5	8	2	4	4	4	4	3	3
B241	2003-2007	Chen, Y. *et al.* & 22808061	BSP5	Taiwan	23.70	120.96	2	2	3	3	2	5	1	7	3	5	2	3	3	2	4	5	8	2	4	4	4	4	3	3
B242	2003-2007	Chen, Y. *et al.* & 22808061	BSP5	Taiwan	23.70	120.96	2	2	3	3	2	5	1	7	3	5	3	3	3	2	4	5	8	2	4	5	4	4	3	3
B243	2003-2007	Chen, Y. *et al.* & 22808061	BSP5	Taiwan	23.70	120.96	2	2	3	3	2	5	1	7	3	5	3	3	3	2	4	5	8	2	4	5	4	2	3	3

B244	2003-2007	Chen, Y. *et al.* & 22808061	BSP5	Taiwan	23.70	120.96	2	2	3	3	2	5	1	7	3	5	3	3	3	2	4	5	8	2	4	4	4	4	3	3
B245	2003-2007	Chen, Y. *et al.* & 22808061	BSP5	Taiwan	23.70	120.96	2	2	3	3	2	5	1	7	3	5	3	3	3	2	4	5	8	2	4	4	4	4	3	3
B246	2003-2007	Chen, Y. *et al.* & 22808061	BSP5	Taiwan	23.70	120.96	2	2	3	3	2	5	1	7	3	5	3	3	3	2	4	5	8	2	3	4	4	4	3	3
B247	2003-2007	Chen, Y. *et al.* & 22808061	BSP5	Taiwan	23.70	120.96	2	2	2	3	2	5	1	7	3	5	3	3	3	2	4	5	8	2	4	4	4	2	3	3
B248	2003-2007	Chen, Y. *et al.* & 22808061	BSP5	Taiwan	23.70	120.96	2	2	2	3	2	5	1	7	3	5	3	3	3	2	4	5	8	2	4	4	4	2	3	3
B249	2003-2007	Chen, Y. *et al.* & 22808061	BSP5	Taiwan	23.70	120.96	2	2	3	3	2	5	1	7	3	5	3	3	4	2	4	5	8	2	4	5	4	4	3	3
B250	2003-2007	Chen, Y. *et al.* & 22808061	BSP5	Taiwan	23.70	120.96	2	2	3	3	2	5	1	7	3	5	2	3	3	2	4	5	8	2	4	4	4	4	3	3
B251	2003-2007	Chen, Y. *et al.* & 22808061	BSP5	Taiwan	23.70	120.96	2	2	3	3	2	5	1	7	3	5	3	3	4	2	4	ND	8	ND	4	5	4	4	3	3
B252	2003-2007	Chen, Y. *et al.* & 22808061	BSP5	Taiwan	23.70	120.96	2	2	3	3	2	5	1	7	3	5	3	3	3	2	4	5	4	2	5	5	4	4	3	3
B253	2003-2007	Chen, Y. *et al.* & 22808061	BSP5	Taiwan	23.70	120.96	2	2	3	3	2	5	1	7	3	5	3	3	4	2	4	5	8	2	4	5	4	4	3	4
B254	2003-2007	Chen, Y. *et al.* & 22808061	BSP5	Taiwan	23.70	120.96	2	2	3	3	2	5	1	7	3	5	3	3	4	2	4	5	8	2	4	5	4	4	3	3
B255	2003-2007	Chen, Y. *et al.* & 22808061	BSP5	Taiwan	23.70	120.96	2	2	3	3	2	5	1	7	3	5	3	3	3	2	4	5	8	2	4	5	4	2	3	3
B256	2003-2007	Chen, Y. *et al.* & 22808061	BSP5	Taiwan	23.70	120.96	2	1	3	3	2	5	1	7	3	5	3	3	3	2	4	5	8	2	4	5	4	4	3	3
B257	2003-2007	Chen, Y. *et al.* & 22808061	BSP5	Taiwan	23.70	120.96	2	1	3	3	2	5	1	7	3	5	3	3	3	2	4	5	8	2	4	5	4	4	3	3
B258	2003-2007	Chen, Y. *et al.* & 22808061	BSP5	Taiwan	23.70	120.96	2	2	3	3	2	5	1	7	3	5	3	3	3	2	4	4	8	2	4	3	4	4	3	3
B259	2003-2007	Chen, Y. *et al.* & 22808061	BSP5	Taiwan	23.70	120.96	2	2	2	3	2	5	1	7	3	5	3	3	3	2	4	5	8	2	4	5	4	2	3	3
B260	2003-2007	Chen, Y. *et al.* & 22808061	BSP5	Taiwan	23.70	120.96	2	2	3	3	2	5	1	8	3	5	3	3	3	2	4	2	7	2	4	5	4	4	3	3
B261	2003-2007	Chen, Y. *et al.* & 22808061	BSP5	Taiwan	23.70	120.96	2	2	3	3	2	5	1	7	3	5	3	1	4	2	4	5	9	2	4	5	4	4	3	3
B262	2003-2007	Chen, Y. *et al.* & 22808061	BSP5	Taiwan	23.70	120.96	2	2	3	3	2	5	1	7	3	5	3	3	4	2	4	5	8	2	4	7	4	4	3	3
B263	2003-2007	Chen, Y. *et al.* & 22808061	BSP5	Taiwan	23.70	120.96	2	2	3	3	2	5	1	7	4	5	3	3	4	2	4	5	8	2	4	5	4	4	3	3
B264	2003-2007	Chen, Y. *et al.* & 22808061	BSP4	Taiwan	23.70	120.96	2	2	3	3	2	5	1	7	3	7	3	3	4	2	4	7	2	4	1	5	4	4	3	3
B265	2003-2007	Chen, Y. *et al.* & 22808061	BSP5	Taiwan	23.70	120.96	2	2	3	3	2	5	1	7	4	5	3	3	4	2	4	5	8	2	4	5	4	4	3	3
B266	2003-2007	Chen, Y. *et al.* & 22808061	BSP5	Taiwan	23.70	120.96	2	2	3	3	2	5	1	7	3	5	3	3	4	2	4	5	8	2	4	5	4	4	3	3
B267	2003-2007	Chen, Y. *et al.* & 22808061	BSP5	Taiwan	23.70	120.96	2	3	2	3	2	5	1	7	3	5	3	3	4	2	4	5	8	2	3	5	4	4	3	3
B268	2003-2007	Chen, Y. *et al.* & 22808061	BSP5	Taiwan	23.70	120.96	2	2	3	3	2	5	1	7	3	5	3	3	4	2	4	5	8	2	4	5	4	4	3	3
B269	2003-2007	Chen, Y. *et al.* & 22808061	BSP5	Taiwan	23.70	120.96	2	2	3	3	2	5	1	7	3	5	3	3	4	2	4	ND	8	2	4	5	4	4	2	3
B270	2003-2007	Chen, Y. *et al.* & 22808061	BSP5	Taiwan	23.70	120.96	2	2	3	3	2	5	1	7	3	5	3	3	4	2	4	5	8	2	4	5	4	4	3	3
B271	2003-2007	Chen, Y. *et al.* & 22808061	BSP5	Taiwan	23.70	120.96	2	2	3	3	2	5	1	7	3	5	3	3	4	2	4	5	8	2	4	5	4	4	3	3
B272	2003-2007	Chen, Y. *et al.* & 22808061	BSP5	Taiwan	23.70	120.96	2	2	3	3	2	5	1	7	3	5	3	3	3	2	4	5	8	2	4	5	4	2	3	3
B273	2003-2007	Chen, Y. *et al.* & 22808061	BSP5	Taiwan	23.70	120.96	2	2	3	3	2	5	1	7	3	4	2	3	3	2	4	5	8	2	4	4	4	4	3	3
B274	2003-2007	Chen, Y. *et al.* & 22808061	BSP5	Taiwan	23.70	120.96	2	2	3	3	2	5	1	7	3	5	3	3	4	2	4	5	8	2	4	5	4	4	3	3
B275	2003-2007	Chen, Y. *et al.* & 22808061	BSP5	Taiwan	23.70	120.96	2	0	3	3	2	5	1	7	3	5	3	3	4	2	4	5	8	2	4	5	4	4	3	4
B276	2003-2007	Chen, Y. *et al.* & 22808061	BSP4	Taiwan	23.70	120.96	2	2	3	3	2	5	1	7	3	5	3	3	4	2	4	5	7	3	4	4	4	2	2	3
B277	2003-2007	Chen, Y. *et al.* & 22808061	BSP4	Taiwan	23.70	120.96	2	2	3	3	2	5	1	4	3	5	3	3	4	2	4	3	8	4	4	4	4	4	3	3
B278	2003-2007	Chen, Y. *et al.* & 22808061	BSP4	Taiwan	23.70	120.96	2	2	1	3	1	5	1	7	3	5	3	3	4	2	4	3	7	4	4	4	4	4	3	3
B279	2003-2007	Chen, Y. *et al.* & 22808061	BSP4	Taiwan	23.70	120.96	2	2	1	3	2	5	1	7	3	5	3	3	4	2	4	6	8	4	3	4	4	4	3	5
B280	2003-2007	Chen, Y. *et al.* & 22808061	BSP4	Taiwan	23.70	120.96	2	2	1	3	2	5	1	7	3	5	3	3	4	2	4	6	8	4	3	4	4	4	3	5
B281	2003-2007	Chen, Y. *et al.* & 22808061	BSP4	Taiwan	23.70	120.96	2	2	2	3	2	5	1	7	3	5	3	3	4	2	4	7	8	4	4	4	4	4	3	3
B282	2003-2007	Chen, Y. *et al.* & 22808061	BSPint	Taiwan	23.70	120.96	2	2	3	3	2	5	1	6	3	5	3	3	4	2	4	3	8	3	4	4	4	2	3	3
B283	2003-2007	Chen, Y. *et al.* & 22808061	BSP4	Taiwan	23.70	120.96	2	2	1	3	2	5	1	7	3	5	3	3	4	2	4	7	8	4	4	4	4	4	3	3
B284	2003-2007	Chen, Y. *et al.* & 22808061	BSP4	Taiwan	23.70	120.96	2	2	1	3	2	5	1	7	3	5	3	3	4	2	4	4	9	4	3	4	4	4	3	3

B285	2003-2007	Chen, Y. *et al.* & 22808061	BSP4	Taiwan	23.70	120.96	2	2	3	3	2	5	1	4	3	5	3	3	4	2	4	3	8	4	4	4	4	4	3	3
B286	2003-2007	Chen, Y. *et al.* & 22808061	BSP4	Taiwan	23.70	120.96	2	2	1	3	2	5	1	7	3	7	3	3	4	2	4	7	8	4	4	4	4	4	3	3
B287	2003-2007	Chen, Y. *et al.* & 22808061	BSPint	Taiwan	23.70	120.96	2	2	3	3	2	5	1	6	3	3	3	3	4	2	4	3	7	4	5	4	4	2	3	4
B288	2003-2007	Chen, Y. *et al.* & 22808061	BSPint	Taiwan	23.70	120.96	2	2	3	3	2	5	1	6	3	3	3	3	4	2	4	3	7	4	5	4	4	2	3	4
B289	2003-2007	Chen, Y. *et al.* & 22808061	BSP1	Taiwan	23.70	120.96	2	2	7	2	2	5	1	6	3	4	3	1	5	3	4	6	8	1	4	4	4	2	3	1
B290	2003-2007	Chen, Y. *et al.* & 22808061	BSP4	Taiwan	23.70	120.96	2	2	3	3	2	5	1	7	3	5	3	3	4	2	4	5	9	3	3	4	4	4	3	3
B291	2003-2007	Chen, Y. *et al.* & 22808061	BSPint	Taiwan	23.70	120.96	2	2	3	4	2	5	1	4	3	5	3	1	4	2	4	5	9	3	2	4	4	4	3	3
B292	2003-2007	Chen, Y. *et al.* & 22808061	BSP1	Taiwan	23.70	120.96	2	5	4	3	2	6	2	2	3	4	3	2	4	6	4	7	5	1	1	ND	3	2	3	2
B293	2003-2007	Chen, Y. *et al.* & 22808061	BSPint	Taiwan	23.70	120.96	2	2	3	3	2	5	1	8	3	5	3	3	4	2	4	2	9	3	4	4	2	2	3	3
B294	2003-2007	Chen, Y. *et al.* & 22808061	BSPint	Taiwan	23.70	120.96	2	2	3	3	2	5	1	6	3	5	3	1	4	2	4	ND	8	5	2	4	4	4	3	3
B295	2003-2007	Chen, Y. *et al.* & 22808061	BSPint	Taiwan	23.70	120.96	2	2	3	3	2	5	1	4	3	5	3	3	4	2	4	4	9	2	4	4	4	4	3	3
B296	2003-2007	Chen, Y. *et al.* & 22808061	BSP4	Taiwan	23.70	120.96	2	2	3	3	2	5	1	7	3	5	3	3	2	2	4	3	7	1	4	4	4	2	3	3
B297	2003-2007	Chen, Y. *et al.* & 22808061	BSP4	Taiwan	23.70	120.96	2	2	3	3	2	5	2	7	3	5	3	3	3	2	4	6	8	4	4	4	4	4	2	3
B298	2003-2007	Chen, Y. *et al.* & 22808061	BSP4	Taiwan	23.70	120.96	2	2	3	3	2	5	2	7	3	5	3	3	3	2	4	6	8	4	4	4	4	4	2	3
B299	2003-2007	Chen, Y. *et al.* & 22808061	BSP4	Taiwan	23.70	120.96	2	2	3	3	2	5	1	ND	3	5	2	3	4	2	4	5	8	4	4	4	4	4	3	3
B300	2003-2007	Chen, Y. *et al.* & 22808061	BSP4	Taiwan	23.70	120.96	2	2	3	2	2	5	1	7	3	5	3	3	4	2	4	5	8	4	4	4	4	4	3	3
B301	2003-2007	Chen, Y. *et al.* & 22808061	BSP4	Taiwan	23.70	120.96	2	2	4	3	2	5	1	7	3	5	3	3	3	2	4	7	7	4	5	4	4	4	3	3
B302	2003-2007	Chen, Y. *et al.* & 22808061	BSP4	Taiwan	23.70	120.96	2	2	3	4	2	5	1	7	3	5	3	1	4	2	4	7	6	4	4	4	4	4	3	3
B303	2003-2007	Chen, Y. *et al.* & 22808061	BSP4	Taiwan	23.70	120.96	2	2	3	3	2	5	1	4	3	5	3	3	4	2	4	1	8	4	4	4	4	4	3	3
B304	2003-2007	Chen, Y. *et al.* & 22808061	BSP5	Taiwan	23.70	120.96	2	2	3	3	2	5	1	7	3	5	3	3	4	2	4	5	8	2	4	5	4	4	3	3
B305	2003-2007	Chen, Y. *et al.* & 22808061	BSP5	Taiwan	23.70	120.96	2	2	3	3	2	5	1	7	3	5	3	3	4	2	4	4	8	2	4	7	4	4	3	3
B306	2003-2007	Chen, Y. *et al.* & 22808061	BSP5	Taiwan	23.70	120.96	2	2	3	3	2	5	1	7	3	5	3	3	4	2	4	4	8	2	4	5	4	4	3	3
B307	2003-2007	Chen, Y. *et al.* & 22808061	BSP5	Taiwan	23.70	120.96	2	2	3	3	2	5	1	7	3	5	3	3	4	2	4	5	8	2	4	5	4	4	3	3
B308	2003-2007	Chen, Y. *et al.* & 22808061	BSP3	Taiwan	23.70	120.96	5	2	2	3	2	5	1	7	3	3	3	3	4	2	4	4	8	3	5	5	4	4	3	3
B309	2003-2007	Chen, Y. *et al.* & 22808061	BSP3	Taiwan	23.70	120.96	5	2	2	3	2	5	1	7	3	3	3	3	4	2	4	4	8	3	5	5	4	4	3	3
B310	2003-2007	Chen, Y. *et al.* & 22808061	BSP3	Taiwan	23.70	120.96	5	2	3	3	2	5	1	7	3	3	3	3	4	2	4	4	8	3	5	5	4	4	3	3
B311	2003-2007	Chen, Y. *et al.* & 22808061	BSP3	Taiwan	23.70	120.96	5	2	3	3	2	5	1	7	3	3	3	3	4	2	4	4	8	3	5	5	4	4	3	3
B312	2003-2007	Chen, Y. *et al.* & 22808061	BSP3	Taiwan	23.70	120.96	5	2	3	3	2	5	1	7	3	3	3	3	4	2	4	5	8	3	5	5	4	4	3	3
B313	2003-2007	Chen, Y. *et al.* & 22808061	BSP3	Taiwan	23.70	120.96	5	2	3	3	2	5	1	7	3	3	3	3	4	2	4	5	8	3	5	5	4	4	3	3
B314	2003-2007	Chen, Y. *et al.* & 22808061	BSP3	Taiwan	23.70	120.96	5	2	3	3	2	5	1	7	3	3	3	3	4	2	4	5	8	3	5	5	4	4	3	3
B315	2003-2007	Chen, Y. *et al.* & 22808061	BSP3	Taiwan	23.70	120.96	5	2	3	3	2	5	1	7	3	3	3	3	4	2	4	5	8	3	5	5	4	4	3	3
B316	2003-2007	Chen, Y. *et al.* & 22808061	BSP3	Taiwan	23.70	120.96	5	2	3	3	2	5	1	7	3	3	3	3	4	2	4	6	8	3	5	4	4	4	3	3
B317	2003-2007	Chen, Y. *et al.* & 22808061	BSP3	Taiwan	23.70	120.96	5	2	3	3	2	5	1	7	3	3	3	3	4	2	4	6	8	3	5	4	4	4	3	3
B318	2003-2007	Chen, Y. *et al.* & 22808061	BSP3	Taiwan	23.70	120.96	5	2	3	3	2	5	1	7	3	3	3	3	4	2	4	6	8	3	5	4	4	4	3	3
B319	2003-2007	Chen, Y. *et al.* & 22808061	BSP3	Taiwan	23.70	120.96	5	2	2	3	2	5	1	7	3	3	3	3	4	2	4	6	8	3	5	5	4	4	3	3
B320	2003-2007	Chen, Y. *et al.* & 22808061	BSP3	Taiwan	23.70	120.96	5	2	2	3	2	5	1	7	3	3	3	3	4	2	4	6	8	3	5	5	4	4	3	3
B321	2003-2007	Chen, Y. *et al.* & 22808061	BSP3	Taiwan	23.70	120.96	5	2	2	3	2	5	1	6	3	3	4	3	4	2	4	6	6	3	5	5	4	4	4	3
B322	2003-2007	Chen, Y. *et al.* & 22808061	BSP3	Taiwan	23.70	120.96	5	2	2	3	2	5	1	6	3	3	4	3	4	2	4	6	6	3	5	5	4	4	4	3
B323	2003-2007	Chen, Y. *et al.* & 22808061	BSP3	Taiwan	23.70	120.96	5	2	2	3	2	5	1	6	3	3	4	3	4	2	4	6	6	3	5	5	4	4	4	3
B324	2003-2007	Chen, Y. *et al.* & 22808061	BSP3	Taiwan	23.70	120.96	5	2	3	3	2	5	1	7	3	3	3	3	4	2	4	6	7	3	5	5	4	4	3	3
B325	2003-2007	Chen, Y. *et al.* & 22808061	BSP3	Taiwan	23.70	120.96	5	2	3	3	2	5	1	7	3	3	3	3	4	2	4	6	7	3	5	5	4	4	3	3

B326	2003-2007	Chen, Y. *et al.* & 22808061	BSP3	Taiwan	23.70	120.96	5	2	3	3	2	5	1	7	3	3	3	3	4	2	5	5	8	3	5	5	4	4	3	3
B327	2003-2007	Chen, Y. *et al.* & 22808061	BSP3	Taiwan	23.70	120.96	5	2	3	3	2	5	1	7	3	3	3	3	4	2	5	5	8	3	5	5	4	4	3	3
B328	2003-2007	Chen, Y. *et al.* & 22808061	BSP3	Taiwan	23.70	120.96	5	2	3	3	2	5	1	7	3	3	3	3	4	2	4	5	8	3	5	5	4	4	3	3
B329	2003-2007	Chen, Y. *et al.* & 22808061	BSP3	Taiwan	23.70	120.96	5	2	3	3	2	5	1	7	3	3	3	3	4	2	5	5	8	3	5	5	4	4	3	3
B330	2003-2007	Chen, Y. *et al.* & 22808061	BSP3	Taiwan	23.70	120.96	5	2	3	3	2	5	1	7	3	3	3	3	4	2	3	6	8	3	5	5	4	4	3	3
B331	2003-2007	Chen, Y. *et al.* & 22808061	BSP3	Taiwan	23.70	120.96	5	2	3	3	2	5	1	7	3	3	3	3	4	2	3	6	8	3	5	5	4	4	3	3
B332	2003-2007	Chen, Y. *et al.* & 22808061	BSP3	Taiwan	23.70	120.96	5	2	3	3	2	5	1	7	3	3	3	3	4	2	4	6	8	3	5	5	4	4	3	3
B333	2003-2007	Chen, Y. *et al.* & 22808061	BSP3	Taiwan	23.70	120.96	5	2	3	3	2	5	1	7	3	3	3	3	4	2	4	6	8	3	5	5	4	4	3	3
B334	2003-2007	Chen, Y. *et al.* & 22808061	BSP3	Taiwan	23.70	120.96	5	2	3	3	2	5	1	7	3	3	3	3	4	2	4	6	8	3	5	5	4	4	3	3
B335	2003-2007	Chen, Y. *et al.* & 22808061	BSP3	Taiwan	23.70	120.96	5	2	3	3	2	5	1	7	3	3	3	3	4	2	4	6	8	3	5	5	4	4	3	3
B336	2003-2007	Chen, Y. *et al.* & 22808061	BSP3	Taiwan	23.70	120.96	5	2	3	3	2	5	1	7	3	3	3	3	4	2	4	6	8	3	5	5	4	4	3	3
B337	2003-2007	Chen, Y. *et al.* & 22808061	BSP3	Taiwan	23.70	120.96	5	2	3	3	2	5	1	7	3	3	3	3	4	2	4	6	8	3	5	5	4	4	3	3
B338	2003-2007	Chen, Y. *et al.* & 22808061	BSP3	Taiwan	23.70	120.96	5	2	3	3	2	5	1	7	3	3	3	3	4	2	4	6	8	3	5	5	4	4	3	3
B339	2003-2007	Chen, Y. *et al.* & 22808061	BSP3	Taiwan	23.70	120.96	5	2	2	3	2	5	1	7	3	3	3	3	4	2	4	6	8	3	5	5	4	4	3	3
B340	2003-2007	Chen, Y. *et al.* & 22808061	BSP3	Taiwan	23.70	120.96	5	2	2	3	2	5	1	7	3	3	3	3	4	2	4	6	8	3	5	5	4	4	3	3
B341	2003-2007	Chen, Y. *et al.* & 22808061	BSP3	Taiwan	23.70	120.96	5	2	2	3	2	5	1	7	3	3	4	3	4	2	4	6	8	3	5	5	4	4	3	3
B342	2003-2007	Chen, Y. *et al.* & 22808061	BSP3	Taiwan	23.70	120.96	5	2	3	3	2	5	1	7	3	3	3	3	4	2	5	5	8	3	5	5	4	4	3	3
B343	2003-2007	Chen, Y. *et al.* & 22808061	BSP3	Taiwan	23.70	120.96	5	2	3	3	2	5	1	7	3	3	3	3	4	2	5	5	8	3	5	5	4	4	3	3
B344	2003-2007	Chen, Y. *et al.* & 22808061	BSP3	Taiwan	23.70	120.96	5	2	3	3	2	5	1	7	3	3	3	3	4	2	5	5	8	3	5	5	4	4	3	3
B345	2003-2007	Chen, Y. *et al.* & 22808061	BSP3	Taiwan	23.70	120.96	5	2	3	3	2	5	1	7	3	3	3	3	4	2	5	5	8	3	5	5	4	4	3	3
B346	2003-2007	Chen, Y. *et al.* & 22808061	BSP3	Taiwan	23.70	120.96	5	2	3	3	2	5	1	7	3	3	3	3	4	2	4	6	9	3	5	5	4	4	3	3
B347	2003-2007	Chen, Y. *et al.* & 22808061	BSP3	Taiwan	23.70	120.96	5	2	3	3	2	5	1	7	3	3	3	3	4	2	4	5	8	3	5	5	4	4	3	3
B348	2003-2007	Chen, Y. *et al.* & 22808061	BSP3	Taiwan	23.70	120.96	5	2	3	3	2	5	1	7	3	3	3	3	4	2	5	5	8	3	5	5	4	4	3	3
B349	2003-2007	Chen, Y. *et al.* & 22808061	BSP3	Taiwan	23.70	120.96	5	2	3	3	2	5	1	7	3	3	3	3	4	2	5	5	8	3	5	5	4	4	3	3
B350	2003-2007	Chen, Y. *et al.* & 22808061	BSP3	Taiwan	23.70	120.96	5	2	2	3	2	5	1	7	3	3	3	3	4	2	4	6	8	3	5	5	4	4	3	3
B351	2003-2007	Chen, Y. *et al.* & 22808061	BSP3	Taiwan	23.70	120.96	5	2	2	3	2	5	1	7	3	3	3	3	4	2	4	6	8	3	5	5	4	4	3	3
B352	2003-2007	Chen, Y. *et al.* & 22808061	BSP3	Taiwan	23.70	120.96	5	2	2	3	2	5	1	7	3	3	3	3	4	2	4	6	8	3	5	5	4	4	3	3
B353	2003-2007	Chen, Y. *et al.* & 22808061	BSP3	Taiwan	23.70	120.96	5	2	2	3	2	5	1	7	3	3	3	3	4	2	4	6	8	3	5	5	4	4	3	3
B354	2003-2007	Chen, Y. *et al.* & 22808061	BSP3	Taiwan	23.70	120.96	5	2	3	3	2	5	1	7	3	3	3	3	4	2	4	6	8	3	5	5	4	4	3	3
B355	2003-2007	Chen, Y. *et al.* & 22808061	BSP3	Taiwan	23.70	120.96	5	2	2	3	2	5	1	7	3	3	3	3	4	2	4	6	8	3	5	5	4	4	3	3
B356	2003-2007	Chen, Y. *et al.* & 22808061	BSP3	Taiwan	23.70	120.96	5	2	2	3	2	5	1	6	3	3	4	3	4	2	4	6	6	3	5	5	4	4	4	3
B357	2003-2007	Chen, Y. *et al.* & 22808061	BSP3	Taiwan	23.70	120.96	5	2	2	3	2	5	1	6	3	3	4	3	4	2	4	6	6	3	5	5	4	4	4	3
B358	2003-2007	Chen, Y. *et al.* & 22808061	BSP3	Taiwan	23.70	120.96	5	2	2	3	2	5	1	6	3	3	4	3	4	2	4	6	6	3	5	5	4	4	4	3
B359	2003-2007	Chen, Y. *et al.* & 22808061	BSP3	Taiwan	23.70	120.96	5	2	3	3	2	5	1	7	3	3	3	3	4	2	4	6	6	3	4	5	4	4	3	3
B360	2003-2007	Chen, Y. *et al.* & 22808061	BSP3	Taiwan	23.70	120.96	5	2	3	3	2	5	1	7	3	3	3	3	4	2	4	6	6	3	4	5	4	4	3	3
B361	2003-2007	Chen, Y. *et al.* & 22808061	BSP3	Taiwan	23.70	120.96	5	2	3	3	2	5	1	7	3	3	3	3	4	2	4	6	7	3	5	5	4	4	3	3
B362	2003-2007	Chen, Y. *et al.* & 22808061	BSP3	Taiwan	23.70	120.96	5	2	3	3	2	5	1	7	3	3	3	3	4	2	4	6	7	3	5	5	4	4	3	3
B363	2003-2007	Chen, Y. *et al.* & 22808061	BSP3	Taiwan	23.70	120.96	5	2	2	3	2	5	1	6	3	3	4	3	4	2	4	6	6	3	5	4	4	4	4	3
B364	2003-2007	Chen, Y. *et al.* & 22808061	BSP3	Taiwan	23.70	120.96	5	2	2	3	2	5	1	6	3	3	4	3	4	2	4	6	6	3	5	4	4	4	4	3
B365	2003-2007	Chen, Y. *et al.* & 22808061	BSP3	Taiwan	23.70	120.96	5	2	3	3	2	5	1	7	3	4	3	3	4	2	4	6	8	3	4	5	4	4	3	3
B366	2003-2007	Chen, Y. *et al.* & 22808061	BSP3	Taiwan	23.70	120.96	5	2	3	3	2	5	1	7	3	4	3	3	4	2	4	6	8	3	4	5	4	4	3	3

B367	2003-2007	Chen, Y. *et al.* & 22808061	BSP3	Taiwan	23.70	120.96	5	2	2	3	2	5	1	7	3	3	4	3	4	2	4	6	8	3	4	5	4	4	3	3
B368	2003-2007	Chen, Y. *et al.* & 22808061	BSP3	Taiwan	23.70	120.96	5	2	2	3	2	5	1	7	3	3	4	3	4	2	4	6	8	3	4	5	4	4	3	3
B369	2003-2007	Chen, Y. *et al.* & 22808061	BSP3	Taiwan	23.70	120.96	5	2	2	3	2	5	1	7	3	3	4	3	4	2	4	6	8	3	4	5	4	4	3	3
B370	2003-2007	Chen, Y. *et al.* & 22808061	BSP3	Taiwan	23.70	120.96	4	2	3	3	2	5	1	7	3	3	3	3	4	2	4	5	8	3	5	4	4	4	3	3
B371	2003-2007	Chen, Y. *et al.* & 22808061	BSP3	Taiwan	23.70	120.96	4	2	3	3	2	5	1	7	3	3	3	3	4	2	4	5	8	3	5	4	4	4	3	3
B372	2003-2007	Chen, Y. *et al.* & 22808061	BSP3	Taiwan	23.70	120.96	5	2	3	3	2	5	1	8	3	3	3	3	4	2	4	4	8	3	5	5	4	4	3	3
B373	2003-2007	Chen, Y. *et al.* & 22808061	BSP3	Taiwan	23.70	120.96	5	2	3	3	2	5	1	8	3	3	3	3	4	2	4	4	8	3	5	5	4	4	3	3
B374	2003-2007	Chen, Y. *et al.* & 22808061	BSP3	Taiwan	23.70	120.96	5	2	3	3	2	5	1	7	3	3	3	3	4	2	4	6	6	3	5	5	4	4	3	3
B375	2003-2007	Chen, Y. *et al.* & 22808061	BSP3	Taiwan	23.70	120.96	5	2	3	3	2	5	1	7	3	3	3	3	4	2	4	6	6	3	5	5	4	4	3	3
B376	2003-2007	Chen, Y. *et al.* & 22808061	BSP3	Taiwan	23.70	120.96	5	2	3	3	2	5	1	7	3	3	3	3	4	2	4	6	8	3	5	5	4	4	3	3
B377	2003-2007	Chen, Y. *et al.* & 22808061	BSP3	Taiwan	23.70	120.96	5	2	3	3	2	5	1	7	3	3	3	3	4	2	4	6	8	3	5	5	4	4	3	3
B378	2003-2007	Chen, Y. *et al.* & 22808061	BSP3	Taiwan	23.70	120.96	5	2	2	3	2	5	1	7	3	3	3	3	4	2	4	3	8	3	5	5	4	4	3	3
B379	2003-2007	Chen, Y. *et al.* & 22808061	BSP3	Taiwan	23.70	120.96	5	2	2	3	2	5	1	7	3	3	3	3	4	2	4	3	8	3	5	5	4	4	3	3
B380	2003-2007	Chen, Y. *et al.* & 22808061	BSP3	Taiwan	23.70	120.96	5	2	2	3	2	5	1	7	3	3	3	3	4	2	4	3	8	3	5	5	4	4	3	3
B381	2003-2007	Chen, Y. *et al.* & 22808061	BSP3	Taiwan	23.70	120.96	5	2	2	3	2	5	1	9	3	2	3	3	4	2	4	6	8	3	5	5	4	4	3	3
B382	2003-2007	Chen, Y. *et al.* & 22808061	BSP3	Taiwan	23.70	120.96	5	2	3	3	2	5	1	7	3	3	3	3	4	2	4	8	8	3	4	5	4	4	3	3
B383	2003-2007	Chen, Y. *et al.* & 22808061	BSP3	Taiwan	23.70	120.96	5	2	3	3	2	5	1	7	3	3	3	1	4	2	4	6	8	3	4	5	4	4	3	3
B384	2003-2007	Chen, Y. *et al.* & 22808061	BSP3	Taiwan	23.70	120.96	5	2	2	3	2	5	1	6	3	3	4	3	4	2	4	6	6	3	5	5	4	4	4	3
B385	2003-2007	Chen, Y. *et al.* & 22808061	BSP3	Taiwan	23.70	120.96	5	2	3	3	2	5	1	7	3	3	3	3	4	2	4	6	8	3	5	5	4	3	3	3
B386	2003-2007	Chen, Y. *et al.* & 22808061	BSP3	Taiwan	23.70	120.96	5	2	3	3	2	5	1	5	3	3	3	4	4	2	4	6	3	3	5	4	4	4	3	3
B387	2003-2007	Chen, Y. *et al.* & 22808061	BSP3	Taiwan	23.70	120.96	5	2	4	3	2	5	1	6	3	5	3	3	4	2	4	6	8	3	5	5	4	4	3	3
B388	2003-2007	Chen, Y. *et al.* & 22808061	BSP3	Taiwan	23.70	120.96	5	2	2	3	2	5	1	6	3	3	3	3	4	2	4	6	8	3	5	4	4	4	3	3
B389	2003-2007	Chen, Y. *et al.* & 22808061	BSP3	Taiwan	23.70	120.96	5	2	2	3	2	5	1	6	3	3	3	3	4	2	4	6	8	3	5	5	4	4	2	3
B390	2003-2007	Chen, Y. *et al.* & 22808061	BSP3	Taiwan	23.70	120.96	4	2	3	3	2	5	1	9	3	2	3	3	4	2	4	6	8	3	5	5	4	4	3	2
B391	2003-2007	Chen, Y. *et al.* & 22808061	BSP3	Taiwan	23.70	120.96	5	2	3	3	2	5	1	7	3	3	3	3	4	2	4	6	8	3	5	5	4	4	2	3
B392	2003-2007	Chen, Y. *et al.* & 22808061	BSP3	Taiwan	23.70	120.96	5	2	3	3	2	5	1	6	3	3	3	3	4	2	4	6	8	3	5	8	4	4	3	3
B393	2003-2007	Chen, Y. *et al.* & 22808061	BSP3	Taiwan	23.70	120.96	5	2	3	3	2	5	1	4	3	3	3	3	3	2	4	6	8	3	5	5	4	4	5	3
B394	2003-2007	Chen, Y. *et al.* & 22808061	BSP3	Taiwan	23.70	120.96	5	2	3	3	2	5	1	5	3	3	3	3	4	2	4	6	8	3	5	4	4	4	3	3
B395	2003-2007	Chen, Y. *et al.* & 22808061	BSP3	Taiwan	23.70	120.96	5	2	3	3	2	5	1	5	3	3	3	3	4	2	4	6	8	3	5	5	4	4	3	3
B396	2003-2007	Chen, Y. *et al.* & 22808061	BSP3	Taiwan	23.70	120.96	5	2	3	3	2	5	1	8	3	3	3	3	4	2	4	6	8	3	4	5	4	4	3	4
B397	2003-2007	Chen, Y. *et al.* & 22808061	BSP3	Taiwan	23.70	120.96	5	2	3	3	2	5	1	8	3	2	3	3	3	2	4	6	8	3	5	5	4	4	3	3
B398	2003-2007	Chen, Y. *et al.* & 22808061	BSP3	Taiwan	23.70	120.96	5	2	3	3	2	5	1	7	3	3	3	3	4	2	4	6	8	3	3	5	4	4	3	3
B399	2003-2007	Chen, Y. *et al.* & 22808061	BSP3	Taiwan	23.70	120.96	5	2	4	3	2	5	1	7	3	3	3	3	4	2	4	6	8	3	4	5	4	4	3	3
B400	2003-2007	Chen, Y. *et al.* & 22808061	BSP3	Taiwan	23.70	120.96	5	2	3	3	2	5	1	7	3	3	3	3	4	2	4	6	8	3	4	4	4	4	3	4
B401	2003-2007	Chen, Y. *et al.* & 22808061	BSP3	Taiwan	23.70	120.96	5	2	3	3	2	5	1	6	3	3	3	3	4	2	4	6	8	3	5	4	4	4	3	3
B402	2003-2007	Chen, Y. *et al.* & 22808061	BSP3	Taiwan	23.70	120.96	5	2	2	3	2	5	1	6	3	3	3	3	4	2	4	6	8	3	5	5	4	4	3	3
B403	2003-2007	Chen, Y. *et al.* & 22808061	BSP3	Taiwan	23.70	120.96	5	2	2	3	2	5	1	7	3	3	3	3	4	2	4	6	8	3	4	5	4	4	3	4
B404	2003-2007	Chen, Y. *et al.* & 22808061	BSP3	Taiwan	23.70	120.96	6	2	2	3	2	5	1	6	3	3	3	3	4	2	4	6	8	3	4	5	4	4	3	3
B405	2003-2007	Chen, Y. *et al.* & 22808061	BSP3	Taiwan	23.70	120.96	4	2	3	3	2	5	1	7	3	3	3	3	4	2	4	6	8	3	5	5	4	4	3	3
B406	2003-2007	Chen, Y. *et al.* & 22808061	BSP3	Taiwan	23.70	120.96	5	2	3	3	2	5	1	4	3	3	3	3	4	2	4	6	8	3	5	5	4	4	3	3
B407	2003-2007	Chen, Y. *et al.* & 22808061	BSP3	Taiwan	23.70	120.96	5	2	3	3	2	5	1	7	3	3	2	3	4	3	4	6	8	3	5	5	4	4	3	4

B408	2003-2007	Chen, Y. *et al.* & 22808061	BSP3	Taiwan	23.70	120.96	5	2	3	3	2	5	1	7	3	3	1	3	4	2	4	6	8	3	4	5	4	4	3	3
B409	2003-2007	Chen, Y. *et al.* & 22808061	BSP3	Taiwan	23.70	120.96	5	2	3	3	2	5	1	6	3	3	3	3	4	2	4	6	8	3	4	5	4	4	3	5
B410	2003-2007	Chen, Y. *et al.* & 22808061	BSP3	Taiwan	23.70	120.96	5	2	3	3	2	5	1	6	3	3	3	3	4	2	5	5	8	3	5	5	4	4	3	3
B411	2003-2007	Chen, Y. *et al.* & 22808061	BSP3	Taiwan	23.70	120.96	5	2	2	3	2	5	1	8	3	3	3	3	4	2	4	5	8	3	5	5	4	4	4	3
B412	2003-2007	Chen, Y. *et al.* & 22808061	BSP3	Taiwan	23.70	120.96	5	2	3	4	2	5	1	6	3	3	3	3	4	2	4	5	8	3	5	5	4	4	2	3
B413	2003-2007	Chen, Y. *et al.* & 22808061	BSP3	Taiwan	23.70	120.96	5	2	3	3	2	5	1	7	3	3	3	3	4	2	4	5	8	3	5	4	4	4	3	3
B414	2003-2007	Chen, Y. *et al.* & 22808061	BSP3	Taiwan	23.70	120.96	5	2	3	3	2	5	1	7	3	3	3	3	4	1	4	5	8	3	5	5	4	4	3	3
B415	2003-2007	Chen, Y. *et al.* & 22808061	BSP3	Taiwan	23.70	120.96	5	2	3	3	2	5	1	7	3	3	3	3	4	2	4	5	8	3	6	5	4	4	3	3
B416	2003-2007	Chen, Y. *et al.* & 22808061	BSP3	Taiwan	23.70	120.96	5	2	3	3	2	5	1	6	3	3	3	3	4	2	4	5	8	3	5	5	4	4	3	3
B417	2003-2007	Chen, Y. *et al.* & 22808061	BSP3	Taiwan	23.70	120.96	5	2	3	3	2	5	1	7	3	3	3	3	4	2	4	5	8	3	6	5	4	4	3	3
B418	2003-2007	Chen, Y. *et al.* & 22808061	BSP3	Taiwan	23.70	120.96	5	2	3	3	2	5	1	7	3	3	3	3	4	2	4	5	8	3	4	6	4	4	3	3
B419	2003-2007	Chen, Y. *et al.* & 22808061	BSP3	Taiwan	23.70	120.96	5	2	3	3	1	5	1	7	3	3	3	3	3	2	4	5	8	3	5	5	4	4	3	3
B420	2003-2007	Chen, Y. *et al.* & 22808061	BSP3	Taiwan	23.70	120.96	5	2	3	3	2	5	1	6	3	3	3	3	4	2	4	4	8	3	3	8	4	4	2	3
B421	2003-2007	Chen, Y. *et al.* & 22808061	BSP3	Taiwan	23.70	120.96	5	2	2	3	2	5	1	6	3	3	3	3	4	2	4	4	8	3	5	5	4	4	3	4
B422	2003-2007	Chen, Y. *et al.* & 22808061	BSP3	Taiwan	23.70	120.96	5	2	3	3	2	5	1	6	3	3	3	3	4	2	4	4	8	3	5	5	4	4	3	3
B423	2003-2007	Chen, Y. *et al.* & 22808061	BSP3	Taiwan	23.70	120.96	5	2	3	3	2	5	1	8	3	3	3	3	4	2	4	4	8	3	5	5	4	4	3	3
B424	2003-2007	Chen, Y. *et al.* & 22808061	BSP3	Taiwan	23.70	120.96	5	2	3	3	2	5	1	7	3	3	3	3	4	2	4	6	6	3	4	5	4	4	3	3
B425	2003-2007	Chen, Y. *et al.* & 22808061	BSP3	Taiwan	23.70	120.96	5	2	3	3	2	5	1	7	3	3	3	3	4	2	4	6	7	3	4	5	4	4	4	3
B426	2003-2007	Chen, Y. *et al.* & 22808061	BSP3	Taiwan	23.70	120.96	5	2	3	3	2	5	1	8	3	3	3	3	4	2	4	6	7	3	3	5	4	4	3	3
B427	2003-2007	Chen, Y. *et al.* & 22808061	BSP3	Taiwan	23.70	120.96	5	2	3	4	2	5	1		3	3	3	3	4	2	4	6	7	3	5	5	4	4	3	3
B428	2003-2007	Chen, Y. *et al.* & 22808061	BSPint	Taiwan	23.70	120.96	3	2	3	3	2	5	1	7	3	3	3	4	4	2	4	6	7	3	4	5	4	4	3	3
B429	2003-2007	Chen, Y. *et al.* & 22808061	BSP3	Taiwan	23.70	120.96	5	2	3	3	2	5	1	7	3	3	3	3	4	2	4	6	7	3	4	4	4	4	3	3
B430	2003-2007	Chen, Y. *et al.* & 22808061	BSP3	Taiwan	23.70	120.96	5	2	3	3	2	5	1	6	3	3	3	3	4	2	4	6	7	3	5	4	4	4	3	3
B431	2003-2007	Chen, Y. *et al.* & 22808061	BSP3	Taiwan	23.70	120.96	5	2	3	3	2	5	1	7	3	3	3	3	4	2	4	6	7	3	4	5	4	4	3	3
B432	2003-2007	Chen, Y. *et al.* & 22808061	BSP3	Taiwan	23.70	120.96	5	2	3	3	2	5	1	7	3	3	3	3	4	2	4	6	7	3	4	4	4	4	3	4
B433	2003-2007	Chen, Y. *et al.* & 22808061	BSP3	Taiwan	23.70	120.96	4	2	3	3	2	5	1	7	3	3	3	3	4	2	4	6	7	3	5	5	4	4	3	3
B434	2003-2007	Chen, Y. *et al.* & 22808061	BSP3	Taiwan	23.70	120.96	5	2	3	3	2	5	1	7	3	3	3	3	4	2	4	3	8	3	3	5	4	4	3	3
B435	2003-2007	Chen, Y. *et al.* & 22808061	BSP3	Taiwan	23.70	120.96	6	2	2	3	2	2	1	7	3	3	3	3	4	2	4	3	8	3	5	4	4	4	3	3
B436	2003-2007	Chen, Y. *et al.* & 22808061	BSP3	Taiwan	23.70	120.96	5	2	3	3	2	5	1	7	3	3	3	3	4	2	4	3	8	3	5	7	4	4	3	3
B437	2003-2007	Chen, Y. *et al.* & 22808061	BSP3	Taiwan	23.70	120.96	5	2	3	3	2	5	1	7	3	3	3	3	4	2	4	3	8	3	3	5	4	4	3	3
B438	2003-2007	Chen, Y. *et al.* & 22808061	BSP3	Taiwan	23.70	120.96	5	2	2	3	2	5	1	7	3	3	3	3	4	2	4	3	8	3	5	5	4	4	3	3
B439	2003-2007	Chen, Y. *et al.* & 22808061	BSP3	Taiwan	23.70	120.96	5	2	3	3	2	5	1	8	3	3	3	3	4	2	4	4	8	3	5	4	4	4	3	3
B440	2003-2007	Chen, Y. *et al.* & 22808061	BSP3	Taiwan	23.70	120.96	5	2	3	3	2	5	1	6	3	2	3	3	4	2	4	5	7	3	5	3	4	4	2	3
B441	2003-2007	Chen, Y. *et al.* & 22808061	BSP3	Taiwan	23.70	120.96	5	2	3	3	2	5	1	7	3	3	3	3	4	2	4	8	7	3	5	5	4	4	3	3
B442	2003-2007	Chen, Y. *et al.* & 22808061	BSP3	Taiwan	23.70	120.96	5	2	2	3	2	5	1	6	3	3	3	3	4	2	4	6	4	3	4	5	4	4	3	3
B443	2003-2007	Chen, Y. *et al.* & 22808061	BSP3	Taiwan	23.70	120.96	5	2	3	3	2	5	1	7	3	3	3	3	4	2	4	6	8	5	5	4	4	4	3	3
B444	2003-2007	Chen, Y. *et al.* & 22808061	BSP3	Taiwan	23.70	120.96	5	2	3	3	2	5	1	7	3	3	3	3	4	2	4	5	4	3	5	8	4	4	3	3
B445	2003-2007	Chen, Y. *et al.* & 22808061	BSP3	Taiwan	23.70	120.96	5	2	3	3	2	5	1	7	3	3	3	3	4	2	4	ND	8	3	5	5	4	4	3	3
B446	2003-2007	Chen, Y. *et al.* & 22808061	BSP3	Taiwan	23.70	120.96	5	2	3	3	2	5	1	7	3	3	3	3	4	2	2	4	8	3	6	5	4	4	3	3
B447	2003-2007	Chen, Y. *et al.* & 22808061	BSP3	Taiwan	23.70	120.96	5	2	3	3	2	5	1	5	3	3	3	3	4	2	4	5	ND	3	5	4	4	4	3	3
B448	2003-2007	Chen, Y. *et al.* & 22808061	BSP3	Taiwan	23.70	120.96	5	2	3	3	2	5	1	7	3	3	3	3	4	2	4	6	9	3	5	5	4	4	3	3

B449	2003-2007	Chen, Y. *et al.* & 22808061	BSP3	Taiwan	23.70	120.96	5	2	2	3	2	5	1	7	3	3	3	2	4	2	4	6	9	3	5	5	4	4	3	4
B450	2003-2007	Chen, Y. *et al.* & 22808061	BSP3	Taiwan	23.70	120.96	5	2	3	3	2	5	1	7	3	3	3	3	3	3	4	6	9	3	5	5	4	4	3	3
B451	2003-2007	Chen, Y. *et al.* & 22808061	BSP3	Taiwan	23.70	120.96	5	2	3	3	2	5	1	7	3	3	3	2	4	2	4	5	7	3	5	4	4	4	3	3
B452	2003-2007	Chen, Y. *et al.* & 22808061	BSP3	Taiwan	23.70	120.96	4	2	3	3	2	5	1	7	3	3	3	5	4	2	4	5	7	3	4	5	4	4	3	3
B453	2003-2007	Chen, Y. *et al.* & 22808061	BSP3	Taiwan	23.70	120.96	5	2	3	3	2	5	1	7	3	3	3	3	4	2	4	6	6	3	5	4	4	4	3	3
B454	2003-2007	Chen, Y. *et al.* & 22808061	BSP3	Taiwan	23.70	120.96	5	2	2	3	2	5	1	6	3	3	4	3	4	2	4	6	6	3	5	5	4	4	4	3
B455	2003-2007	Chen, Y. *et al.* & 22808061	BSP3	Taiwan	23.70	120.96	5	2	3	3	2	5	1	7	3	3	3	3	4	2	4	5	8	5	5	5	4	4	4	3
B456	2003-2007	Chen, Y. *et al.* & 22808061	BSP3	Taiwan	23.70	120.96	5	2	3	3	2	5	1	7	3	3	3	3	4	2	4	2	8	3	5	4	4	4	3	3
B457	2003-2007	Chen, Y. *et al.* & 22808061	BSP3	Taiwan	23.70	120.96	4	2	3	3	2	5	1	7	3	3	3	1	4	2	4	3	8	3	5	8	4	4	3	3
B458	2003-2007	Chen, Y. *et al.* & 22808061	BSP3	Taiwan	23.70	120.96	5	2	3	3	2	5	1	7	1	3	3	1	4	2	4	9	ND	3	5	4	4	4	3	3
B459	2003-2007	Chen, Y. *et al.* & 22808061	BSP3	Taiwan	23.70	120.96	5	2	3	3	2	5	1	7	3	3	3	3	4	2	4	2	7	3	4	5	4	4	3	4
B460	2003-2007	Chen, Y. *et al.* & 22808061	BSP3	Taiwan	23.70	120.96	4	2	3	3	2	5	1	7	3	3	1	3	4	2	4	5	9	3	5	5	4	4	3	3
B461	2003-2007	Chen, Y. *et al.* & 22808061	BSP3	Taiwan	23.70	120.96	5	2	2	3	2	5	1	6	3	3	3	3	4	1	4	4	7	3	5	5	4	4	3	4
B462	2003-2007	Chen, Y. *et al.* & 22808061	BSP3	Taiwan	23.70	120.96	5	2	3	3	2	5	1	6	3	3	3	3	4	2	4	6	8	3	5	5	4	4	3	3
B463	2003-2007	Chen, Y. *et al.* & 22808061	BSP3	Taiwan	23.70	120.96	5	2	3	3	2	5	1	8	3	3	3	3	4	2	4	6	8	3	5	5	4	4	3	3
B464	2003-2007	Chen, Y. *et al.* & 22808061	BSP3	Taiwan	23.70	120.96	4	2	3	3	2	5	1	4	3	3	3	3	3	2	4	6	8	3	5	5	4	4	5	3
B465	2003-2007	Chen, Y. *et al.* & 22808061	BSP3	Taiwan	23.70	120.96	5	2	2	3	2	5	1	7	3	3	3	3	4	2	4	6	8	3	3	4	4	4	3	5
B466	2003-2007	Chen, Y. *et al.* & 22808061	BSP3	Taiwan	23.70	120.96	5	2	3	3	2	5	1	7	3	3	3	3	4	2	4	6	8	3	5	5	4	4	3	4
B467	2003-2007	Chen, Y. *et al.* & 22808061	BSP3	Taiwan	23.70	120.96	5	2	3	3	2	5	1	ND	3	3	3	3	4	2	4	6	8	3	5	9	4	4	3	3
B468	2003-2007	Chen, Y. *et al.* & 22808061	BSP3	Taiwan	23.70	120.96	5	2	2	3	2	5	1	7	3	3	4	3	4	2	4	6	8	3	5	5	4	4	3	3
B469	2003-2007	Chen, Y. *et al.* & 22808061	BSP3	Taiwan	23.70	120.96	5	2	3	3	2	5	1	7	3	3	3	3	4	2	4	6	8	3	5	6	4	4	3	3
B470	2003-2007	Chen, Y. *et al.* & 22808061	BSP3	Taiwan	23.70	120.96	5	2	5	3	2	5	1	6	2	3	3	3	4	2	4	6	9	3	5	5	4	4	3	3
B471	2003-2007	Chen, Y. *et al.* & 22808061	BSP3	Taiwan	23.70	120.96	5	2	3	3	2	5	1	6	3	3	3	3	4	2	4	6	9	3	5	6	4	4	3	3
B472	2003-2007	Chen, Y. *et al.* & 22808061	BSP3	Taiwan	23.70	120.96	5	2	3	3	2	5	1	8	3	3	3	3	4	3	4	6	9	3	5	5	4	4	3	3
B473	2003-2007	Chen, Y. *et al.* & 22808061	BSP3	Taiwan	23.70	120.96	5	2	3	3	2	5	1	7	3	3	3	3	4	2	4	6	9	3	3	5	4	4	3	3
B474	2003-2007	Chen, Y. *et al.* & 22808061	BSP3	Taiwan	23.70	120.96	5	2	2	3	2	5	1	7	3	3	3	3	4	2	4	6	9	3	5	5	4	4	3	3
B475	2003-2007	Chen, Y. *et al.* & 22808061	BSP3	Taiwan	23.70	120.96	5	2	3	3	2	5	1	7	3	3	3	3	4	2	4	6	9	3	3	5	4	4	3	3
B476	2003-2007	Chen, Y. *et al.* & 22808061	BSP3	Taiwan	23.70	120.96	6	2	3	3	2	5	1	7	3	3	3	3	4	2	4	6	7	3	5	5	4	4	3	3
B477	2003-2007	Chen, Y. *et al.* & 22808061	BSP3	Taiwan	23.70	120.96	5	2	3	3	2	5	1	7	3	3	3	3	4	2	4	5	4	3	5	5	4	4	3	3
B478	2003-2007	Chen, Y. *et al.* & 22808061	BSP3	Taiwan	23.70	120.96	4	2	3	3	2	5	1	8	3	3	3	3	4	2	4	7	9	3	5	5	4	4	3	3
B479	2003-2007	Chen, Y. *et al.* & 22808061	BSP3	Taiwan	23.70	120.96	5	2	3	3	2	5	1	7	3	3	3	3	4	2	4	5	4	3	5	5	4	4	3	3
B480	2003-2007	Chen, Y. *et al.* & 22808061	BSP3	Taiwan	23.70	120.96	5	2	3	3	2	5	1	7	3	3	3	3	3	2	4	3	8	3	5	5	4	4	3	3
B481	2003-2007	Chen, Y. *et al.* & 22808061	BSP3	Taiwan	23.70	120.96	5	2	3	3	2	5	1	7	3	3	3	3	4	2	4	6	9	3	5	5	4	4	3	3
B482	2003-2007	Chen, Y. *et al.* & 22808061	BSP3	Taiwan	23.70	120.96	8	2	3	3	2	5	1	7	3	3	3	3	4	2	4	4	8	3	5	5	4	4	2	3
B483	2003-2007	Chen, Y. *et al.* & 22808061	BSP3	Taiwan	23.70	120.96	5	2	3	3	2	5	1	7	3	1	3	3	4	2	4	8	8	3	5	1	4	4	3	3
B484	2003-2007	Chen, Y. *et al.* & 22808061	BSP3	Taiwan	23.70	120.96	5	2	3	3	2	5	1	7	3	3	3	3	4	2	4	5	5	3	4	5	4	4	3	3
B485	2003-2007	Chen, Y. *et al.* & 22808061	BSP3	Taiwan	23.70	120.96	5	2	3	3	2	5	1	8	3	3	3	3	4	2	4	4	8	3	5	5	4	4	3	3
B486	2003-2007	Chen, Y. *et al.* & 22808061	BSP3	Taiwan	23.70	120.96	6	2	3	3	2	5	1	7	3	3	3	3	4	2	4	6	8	3	6	4	4	4	3	3
B487	2003-2007	Chen, Y. *et al.* & 22808061	BSP3	Taiwan	23.70	120.96	5	2	3	3	2	5	1	7	3	3	3	3	4	2	5	5	8	3	5	5	4	4	3	3
B488	2003-2007	Chen, Y. *et al.* & 22808061	BSP4	Taiwan	23.70	120.96	2	2	2	3	2	5	1	7	3	5	3	4	4	2	4	7	8	5	4	4	4	4	3	3
B489	2003-2007	Chen, Y. *et al.* & 22808061	BSP4	Taiwan	23.70	120.96	2	2	3	4	2	5	1	7	3	5	3	3	4	2	4	4	8	4	4	4	4	4	3	3

B490	2003-2007	Chen, Y. *et al.* & 22808061	BSP4	Taiwan	23.70	120.96	2	2	3	3	2	5	1	7	3	5	3	3	4	2	4	6	7	4	4	3	4	4	3	3
B491	2003-2007	Chen, Y. *et al.* & 22808061	BSP4	Taiwan	23.70	120.96	2	2	3	3	2	5	1	7	3	5	3	3	4	1	4	6	8	4	4	4	4	4	3	3
B492	2003-2007	Chen, Y. *et al.* & 22808061	BSP4	Taiwan	23.70	120.96	2	2	3	3	2	5	1	7	3	5	3	3	4	2	4	7	7	4	4	4	4	4	3	3
B493	2003-2007	Chen, Y. *et al.* & 22808061	BSP4	Taiwan	23.70	120.96	2	2	3	3	2	5	1	7	3	5	3	3	4	2	4	6	9	4	4	4	4	4	3	3
B494	2003-2007	Chen, Y. *et al.* & 22808061	BSP4	Taiwan	23.70	120.96	2	2	3	3	2	5	1	7	3	5	3	3	4	2	4	5	8	4	5	4	4	4	3	3
B495	2003-2007	Chen, Y. *et al.* & 22808061	BSP4	Taiwan	23.70	120.96	2	2	3	3	2	5	1	7	3	6	3	3	4	2	4	4	3	4	4	4	4	4	3	3
B496	2003-2007	Chen, Y. *et al.* & 22808061	BSP4	Taiwan	23.70	120.96	2	2	3	3	2	5	1	7	3	6	3	3	4	2	4	4	3	4	4	4	4	4	3	3
B497	2003-2007	Chen, Y. *et al.* & 22808061	BSP5	Taiwan	23.70	120.96	2	2	3	3	2	5	1	6	3	5	3	3	4	2	4	6	6	2	4	4	4	4	3	3
B498	2003-2007	Chen, Y. *et al.* & 22808061	BSPint	Taiwan	23.70	120.96	2	2	2	3	2	5	1	7	3	4	3	1	4	2	4	6	8	4	4	3	4	4	3	1
B499	2003-2007	Chen, Y. *et al.* & 22808061	BSP5	Taiwan	23.70	120.96	2	2	3	3	2	5	1	5	3	4	3	3	4	2	4	7	ND	2	4	4	4	4	3	3
B500	2003-2007	Chen, Y. *et al.* & 22808061	BSP4	Taiwan	23.70	120.96	2	2	3	2	2	5	1	ND	3	5	2	3	4	2	4	7	4	4	4	4	4	4	3	3
B501	2003-2007	Chen, Y. *et al.* & 22808061	BSP4	Taiwan	23.70	120.96	2	2	3	3	2	5	1	7	3	5	3	4	4	2	2	6	8	4	5	4	4	4	3	3
B502	2003-2007	Chen, Y. *et al.* & 22808061	BSP4	Taiwan	23.70	120.96	2	2	3	2	2	5	1	ND	3	5	2	3	4	2	4	7	4	4	4	4	4	4	3	3
B503	2003-2007	Chen, Y. *et al.* & 22808061	BSP4	Taiwan	23.70	120.96	2	2	3	3	2	5	1	7	3	5	3	4	4	2	2	6	8	4	5	4	4	4	3	3
B504	2003-2007	Chen, Y. *et al.* & 22808061	BSP4	Taiwan	23.70	120.96	2	2	3	3	2	5	1	7	3	5	3	3	4	2	4	6	8	4	4	4	4	4	3	3
B505	2003-2007	Chen, Y. *et al.* & 22808061	BSP4	Taiwan	23.70	120.96	2	2	3	3	2	5	1	6	3	5	3	2	4	2	4	ND	8	4	2	4	4	4	3	3
B506	2003-2007	Chen, Y. *et al.* & 22808061	BSP4	Taiwan	23.70	120.96	2	2	3	3	2	5	1	7	3	5	3	3	4	1	4	6	8	4	4	4	4	4	3	3
B507	2003-2007	Chen, Y. *et al.* & 22808061	BSP4	Taiwan	23.70	120.96	2	2	3	3	2	5	1	7	3	5	3	3	4	2	4	7	7	1	3	4	4	4	3	3
B508	2003-2007	Chen, Y. *et al.* & 22808061	BSP4	Taiwan	23.70	120.96	2	2	3	3	2	5	1	7	3	5	3	3	4	2	4	7	7	4	4	4	4	4	3	3
B509	2003-2007	Chen, Y. *et al.* & 22808061	BSP4	Taiwan	23.70	120.96	2	2	3	3	2	5	1	6	3	5	3	3	4	2	4	7	ND	4	3	4	4	4	3	3
B510	2003-2007	Chen, Y. *et al.* & 22808061	BSP4	Taiwan	23.70	120.96	2	2	3	3	2	5	1	7	3	5	3	3	4	2	4	6	8	4	1	4	4	4	3	4
B511	2003-2007	Chen, Y. *et al.* & 22808061	BSP4	Taiwan	23.70	120.96	2	2	3	3	2	5	1	7	3	5	3	3	4	2	4	6	8	4	4	4	4	4	3	3
B512	2003-2007	Chen, Y. *et al.* & 22808061	BSP4	Taiwan	23.70	120.96	6	2	3	3	2	5	1	7	3	4	3	3		2	4	7	8	4	4	4	4	4	3	3
B513	2003-2007	Chen, Y. *et al.* & 22808061	BSP4	Taiwan	23.70	120.96	2	2	3	3	2	5	1	7	3	5	3	3	4	2	4	6	7	4	2	4	4	4	3	3
B514	2003-2007	Chen, Y. *et al.* & 22808061	BSP4	Taiwan	23.70	120.96	2	2	3	3	2	5	1	7	3	5	3	3	4	2	4	6	7	4	2	4	4	4	3	3
B515	2003-2007	Chen, Y. *et al.* & 22808061	BSPint	Taiwan	23.70	120.96	2	2	3	3	2	5	1	6	3	4	4	2	4	2	4	6	8	4	4	2	4	4	3	3
B516	2003-2007	Chen, Y. *et al.* & 22808061	BSPint	Taiwan	23.70	120.96	2	2	3	3	2	5	1	6	3	4	4	2	4	2	4	6	8	4	4	2	4	4	3	3
B517	2003-2007	Chen, Y. *et al.* & 22808061	BSP4	Taiwan	23.70	120.96	2	2	3	3	2	5	1	7	3	5	3	1	4	2	4	6	7	4	3	4	4	4	3	3
B518	2003-2007	Chen, Y. *et al.* & 22808061	BSP4	Taiwan	23.70	120.96	2	2	3	2	2	5	1	7	3	5	3	3	4	2	4	7	8	4	2	4	4	4	3	3
B519	2003-2007	Chen, Y. *et al.* & 22808061	BSPint	Taiwan	23.70	120.96	2	2	3	3	2	5	1	6	3	4	4	2	4	2	4	6	8	4	4	2	4	4	3	3
B520	2003-2007	Chen, Y. *et al.* & 22808061	BSP4	Taiwan	23.70	120.96	2	2	3	3	2	5	1	7	3	4	3	3	4	2	4	5	9	4	4	4	4	4	3	3
B521	2003-2007	Chen, Y. *et al.* & 22808061	BSP4	Taiwan	23.70	120.96	2	2	3	3	2	5	1	7	3	6	3	3	4	2	4	7	ND	4	4	4	4	4	3	3
B522	2003-2007	Chen, Y. *et al.* & 22808061	BSP4	Taiwan	23.70	120.96	2	2	3	3	2	5	1	7	3	6	3	3	4	2	4	7	ND	4	4	4	4	4	3	3
B523	2003-2007	Chen, Y. *et al.* & 22808061	BSP5	Taiwan	23.70	120.96	2	2	3	3	2	5	1	5	3	4	3	4	4	2	4	7	9	2	4	4	4	4	3	3
B524	2003-2007	Chen, Y. *et al.* & 22808061	BSP4	Taiwan	23.70	120.96	2	2	3	3	2	5	1	7	3	5	3	3	4	2	3	6	8	4	4	4	4	4	3	3
B525	2003-2007	Chen, Y. *et al.* & 22808061	BSP4	Taiwan	23.70	120.96	2	2	3	2	2	5	1	6	3	5	3	2	4	2	4	7	8	4	4	4	4	4	3	3
B526	2003-2007	Chen, Y. *et al.* & 22808061	BSP5	Taiwan	23.70	120.96	2	2	3	3	2	5	1	5	3	5	3	3	4	2	4	5	ND	2	4	4	4	4	3	3
B527	2003-2007	Chen, Y. *et al.* & 22808061	BSP4	Taiwan	23.70	120.96	2	2	3	3	2	5	1	7	3	5	3	3	4	2	4	6	8	4	3	4	4	4	3	3
B528	2003-2007	Chen, Y. *et al.* & 22808061	BSP4	Taiwan	23.70	120.96	3	2	3	3	2	5	1	7	3	5	3	3	4	2	4	7	5	4	3	3	4	4	3	3
B529	2003-2007	Chen, Y. *et al.* & 22808061	BSP4	Taiwan	23.70	120.96	2	2	3	3	2	5	1	7	3	3	3	3	4	1	4	6	8	4	3	4	4	4	3	3
B530	2003-2007	Chen, Y. *et al.* & 22808061	BSP4	Taiwan	23.70	120.96	2	2	3	3	2	5	1	6	3	4	3	3	4	2	4	4	6	4	1	4	4	4	3	3

B531	2003-2007	Chen, Y. *et al.* & 22808061	BSP4	Taiwan	23.70	120.96	2	2	3	3	2	5	1	7	3	5	3	4	4	2	4	7	8	4	3	4	4	4	3	3
B532	2003-2007	Chen, Y. *et al.* & 22808061	BSP5	Taiwan	23.70	120.96	2	2	3	3	2	5	1	7	3	5	3	4	3	2	4	3	8	2	1	5	4	4	3	3
B533	2003-2007	Chen, Y. *et al.* & 22808061	BSP4	Taiwan	23.70	120.96	2	2	3	3	2	5	1	9	3	5	3	3	4	2	4	4	8	5	4	4	4	4	3	3
B534	2003-2007	Chen, Y. *et al.* & 22808061	BSP4	Taiwan	23.70	120.96	2	2	3	3	2	5	1	7	3	5	3	3	4	2	4	7	ND	4	4	4	4	4	3	3
B535	2003-2007	Chen, Y. *et al.* & 22808061	BSP4	Taiwan	23.70	120.96	2	2	3	4	2	5	1	7	3	5	3	3	4	2	4	7	2	4	3	4	4	4	3	3
B536	2003-2007	Chen, Y. *et al.* & 22808061	BSP5	Taiwan	23.70	120.96	2	2	3	3	2	5	1	5	3	5	2	3	3	2	4	6	8	2	4	4	4	4	3	3
B537	2003-2007	Chen, Y. *et al.* & 22808061	BSP4	Taiwan	23.70	120.96	2	4	3	3	2	5	1	7	3	5	3	4	4	2	4	3	7	4	4	4	4	4	3	4
B538	2008	Zhao, Y. *et al.* & 21989209	BSP1	Sichuan	30.65	104.08	2	3	3	3	2	5	1	7	2	4	3	3	3	2	4	4	7	1	4	7	4	2	3	4
B539	2008	Zhao, Y. *et al.* & 21989209	BSP1	Sichuan	30.65	104.08	2	3	3	3	2	5	1	7	2	4	4	3	4	2	4	4	8	1	4	6	4	4	3	4
B540	2008	Zhao, Y. *et al.* & 21989209	BSP1	Sichuan	30.65	104.08	2	3	3	3	2	5	1	7	2	4	3	5	3	2	4	4	8	1	5	7	4	2	3	4
B541	2008	Zhao, Y. *et al.* & 21989209	BSPint	Sichuan	30.65	104.08	2	3	3	3	2	5	1	7	2	4	3	3	4	2	4	6	8	2	4	4	2	4	3	4
B542	2008	Zhao, Y. *et al.* & 21989209	BSPint	Sichuan	30.65	104.08	2	3	3	3	2	5	1	7	3	4	3	3	4	2	4	6	ND	2	4	3	4	4	3	4
B543	2008	Zhao, Y. *et al.* & 21989209	BSP1	Sichuan	30.65	104.08	2	3	3	3	2	5	1	4	4	4	4	3	4	2	4	2	8	2	4	4	4	4	3	4
B544	2008	Zhao, Y. *et al.* & 21989209	BSP1	Sichuan	30.65	104.08	2	3	3	3	2	5	1	9	4	4	4	3	4	2	5	2	6	2	4	4	4	6	3	5
B545	2008	Zhao, Y. *et al.* & 21989209	BSP1	Sichuan	30.65	104.08	2	3	2	3	2	5	1	7	3	4	4	5	4	2	4	6	7	3	2	4	4	4	3	4
B546	2008	Zhao, Y. *et al.* & 21989209	BSP1	Sichuan	30.65	104.08	2	3	3	4	2	5	1	3	2	4	4	5	4	2	5	2	6	4	3	4	4	4	3	4
B547	2008	Zhao, Y. *et al.* & 21989209	BSP1	Sichuan	30.65	104.08	2	3	2	1	2	5	1	7	2	4	3	5	4	2	4	6	8	2	2	4	4	4	3	4
B548	2008	Zhao, Y. *et al.* & 21989209	BSP1	Sichuan	30.65	104.08	2	3	2	3	2	5	1	7	2	5	4	5	4	2	4	7	7	4	4	5	4	4	3	5
B549	2008	Zhao, Y. *et al.* & 21989209	BSP1	Sichuan	30.65	104.08	2	3	2	3	2	5	1	7	2	5	4	5	4	2	4	6	3	2	4	6	4	4	3	5
B550	2008	Zhao, Y. *et al.* & 21989209	BSP1	Sichuan	30.65	104.08	2	3	2	3	2	5	1	7	2	4	3	5	4	2	4	7	7	4	0	5	4	4	3	5
B551	2008	Zhao, Y. *et al.* & 21989209	BSP1	Sichuan	30.65	104.08	2	3	2	3	2	5	1	7	3	5	4	5	4	2	4	6	8	1	4	5	4	4	3	4
B552	2008	Zhao, Y. *et al.* & 21989209	BSP1	Sichuan	30.65	104.08	2	3	2	3	2	5	1	6	2	5	4	5	4	2	4	7	8	4	2	5	4	4	2	5
B553	2008	Zhao, Y. *et al.* & 21989209	BSP1	Sichuan	30.65	104.08	2	3	2	3	2	8	1	2	1	5	3	5	4	2	4	5	6	2	2	5	4	4	3	4
B554	2008	Zhao, Y. *et al.* & 21989209	BSP1	Sichuan	30.65	104.08	2	3	2	2	2	5	1	7	2	4	2	3	4	2	4	3	8	1	4	5	4	4	3	4
B555	2008	Zhao, Y. *et al.* & 21989209	BSP1	Sichuan	30.65	104.08	2	3	2	3	2	5	1	7	2	4	1	3	4	2	4	6	8	1	2	5	4	4	3	4
B556	2008	Zhao, Y. *et al.* & 21989209	BSP1	Sichuan	30.65	104.08	2	3	3	2	2	5	1	8	2	4	1	2	4	2	4	6	8	1	3	5	4	4	3	4
B557	2008	Zhao, Y. *et al.* & 21989209	BSP1	Sichuan	30.65	104.08	2	3	3	2	2	5	1	8	2	4	2	2	4	2	4	5	2	3	2	4	5	4	3	4
B558	2008	Zhao, Y. *et al.* & 21989209	BSP1	Sichuan	30.65	104.08	2	3	3	2	2	5	1	6	2	4	2	2	4	2	4	6	8	1	3	5	4	4	3	4
B559	2008	Zhao, Y. *et al.* & 21989209	BSP1	Sichuan	30.65	104.08	2	3	3	2	2	5	1	8	2	4	1	2	4	2	4	6	7	1	3	5	4	4	3	3
B560	2008	Zhao, Y. *et al.* & 21989209	BSP1	Sichuan	30.65	104.08	2	3	3	2	2	5	1	8	2	4	2	2	4	2	4	6	ND	1	6	3	4	4	3	4
B561	2008	Zhao, Y. *et al.* & 21989209	BSP1	Sichuan	30.65	104.08	2	3	2	2	2	5	1	3	2	4	1	1	4	2	4	3	6	1	2	5	4	4	3	4
B562	2008	Zhao, Y. *et al.* & 21989209	BSP1	Sichuan	30.65	104.08	2	3	3	2	2	5	1	4	2	4	2	1	4	2	4	4	6	1	2	4	4	4	3	4
B563	2008	Zhao, Y. *et al.* & 21989209	BSP1	Sichuan	30.65	104.08	2	3	3	2	2	5	1	7	2	4	2	3	4	2	4	3	2	3	2	4	4	4	3	4
B564	2008	Zhao, Y. *et al.* & 21989209	BSP1	Sichuan	30.65	104.08	2	3	3	2	2	5	1	4	2	4	2	2	4	2	4	3	6	1	2	5	4	4	3	4
B565	2008	Zhao, Y. *et al.* & 21989209	BSP1	Sichuan	30.65	104.08	2	3	3	1	2	5	1	1	2	3	1	3	4	2	4	6	9	1	2	4	4	4	3	3
B566	2008	Zhao, Y. *et al.* & 21989209	BSP1	Sichuan	30.65	104.08	2	3	3	1	2	5	1	7	1	4	1	3	4	2	4	4	8	1	2	5	4	4	3	4
B567	2008	Zhao, Y. *et al.* & 21989209	BSP1	Sichuan	30.65	104.08	2	3	3	1	2	5	1	7	2	4	1	4	4	2	4	4	8	1	2	5	4	4	3	4
B568	2008	Zhao, Y. *et al.* & 21989209	BSP1	Sichuan	30.65	104.08	2	3	3	2	2	5	1	8	2	4	2	3	3	2	4	6	7	3	1	4	4	4	3	4
B569	2008	Zhao, Y. *et al.* & 21989209	BSP1	Sichuan	30.65	104.08	2	3	3	2	2	5	1	6	2	4	3	2	4	2	4	5	7	1	2	5	4	4	3	5
B570	2008	Zhao, Y. *et al.* & 21989209	BSP1	Sichuan	30.65	104.08	2	3	2	2	2	5	1	6	2	4	2	3	4	2	4	6	8	4	3	4	2	4	3	3
B571	2008	Zhao, Y. *et al.* & 21989209	BSP1	Sichuan	30.65	104.08	2	3	2	2	2	5	1	4	2	4	2	3	4	2	4	5	8	1	2	5	2	4	3	3

B572	2008	Zhao, Y. *et al.* & 21989209	BSP1	Sichuan	30.65	104.08	2	3	2	1	2	5	1	5	2	3	2	3	4	2	4	5	8	1	3	5	4	4	3	3
B573	2008	Zhao, Y. *et al.* & 21989209	BSP1	Sichuan	30.65	104.08	2	3	2	2	2	5	1	6	2	4	2	4	4	2	4	5	8	1	3	5	4	5	3	3
B574	2008	Zhao, Y. *et al.* & 21989209	BSP1	Sichuan	30.65	104.08	2	3	2	1	2	5	1	6	1	4	2	3	4	2	4	6	8	3	3	4	2	4	3	3
B575	2008	Zhao, Y. *et al.* & 21989209	BSP1	Sichuan	30.65	104.08	2	3	1	1	2	5	1	4	1	3	3	ND	4	2	4	5	6	1	3	5	4	4	3	3
B576	2008	Zhao, Y. *et al.* & 21989209	BSP1	Sichuan	30.65	104.08	2	3	2	1	2	5	1	6	1	1	2	3	4	2	4	5	8	1	3	5	4	4	3	2
B577	2008	Zhao, Y. *et al.* & 21989209	BSP1	Sichuan	30.65	104.08	2	3	2	1	2	5	1	5	2	3	2	3	4	2	4	5	8	1	3	5	4	4	3	3
B578	2008	Zhao, Y. *et al.* & 21989209	BSP1	Sichuan	30.65	104.08	2	3	3	2	2	7	1	7	3	5	1	3	4	2	4	ND	7	0	4	4	4	4	3	4
B579	2008	Zhao, Y. *et al.* & 21989209	BSPint	Sichuan	30.65	104.08	2	2	3	2	2	5	1	7	3	4	3	3	4	2	4	4	5	0	4	5	4	4	3	3
B580	2008	Zhao, Y. *et al.* & 21989209	BSP1	Sichuan	30.65	104.08	2	3	3	2	2	5	1	5	3	5	3	2	4	2	4	4	7	1	3	4	4	4	3	4
B581	2008	Zhao, Y. *et al.* & 21989209	BSP1	Sichuan	30.65	104.08	2	3	3	2	2	5	1	7	3	4	3	3	3	2	4	4	7	0	3	6	4	2	3	3
B582	2008	Zhao, Y. *et al.* & 21989209	BSP1	Sichuan	30.65	104.08	2	3	3	3	2	5	1		3	4	3	2	3	2	4	4	7	0	3	4	4	4	3	3
B583	2008	Zhao, Y. *et al.* & 21989209	BSP1	Sichuan	30.65	104.08	2	3	1	2	2	5	1	7	3	5	3	2	4	2	4	6	7	0	4	3	4	4	3	3
B584	2008	Zhao, Y. *et al.* & 21989209	BSP1	Sichuan	30.65	104.08	2	3	2	2	2	5	1	7	4	4	2	2	4	2	4	4	6	0	3	5	4	4	3	3
B585	2008	Zhao, Y. *et al.* & 21989209	BSP1	Sichuan	30.65	104.08	2	3	2	1	2	5	1	7	3	4	2	2	4	2	4	5	8	0	3	5	4	4	3	2
B586	2008	Zhao, Y. *et al.* & 21989209	BSP1	Sichuan	30.65	104.08	2	3	1	1	2	5	1	7	3	4	3	2	3	2	4	3	7	0	3	2	4	2	3	3
B587	2008	Zhao, Y. *et al.* & 21989209	BSP1	Sichuan	30.65	104.08	2	3	2	3	2	5	1	4	3	3	2	1	ND	2	4	3	7	0	1	2	4	4	3	5
B588	2008	Zhao, Y. *et al.* & 21989209	BSP1	Sichuan	30.65	104.08	2	3	3	1	2	4	1	7	3	3	3	3	4	2	4	4	7	1	2	5	4	4	3	3
B589	2008	Zhao, Y. *et al.* & 21989209	BSP1	Sichuan	30.65	104.08	2	3	2	1	2	4	1	6	3	3	3	3	4	2	4	5	7	1	2	6	2	4	3	3
B590	2008	Zhao, Y. *et al.* & 21989209	BSP1	Sichuan	30.65	104.08	2	3	2	1	2	4	1	8	3	3	3	3	4	2	4	5	6	1	2	7	4	4	3	4
B591	2008	Zhao, Y. *et al.* & 21989209	BSP1	Sichuan	30.65	104.08	2	3	2	1	2	4	1	7	3	3	3	3	2	2	4	4	6	2	2	5	4	4	2	3
B592	2008	Zhao, Y. *et al.* & 21989209	BSP1	Sichuan	30.65	104.08	2	3	2	0	2	4	1	8	3	3	3	3	ND	2	4	5	7	1	1	9	4	4	3	3
B593	2008	Zhao, Y. *et al.* & 21989209	BSP1	Sichuan	30.65	104.08	2	3	2	1	2	4	1	8	3	3	3	2	4	2	4	4	8	1	2	6	4	4	3	3
B594	2008	Zhao, Y. *et al.* & 21989209	BSP1	Sichuan	30.65	104.08	2	2	2	2	2	4	1	3	3	5	3	3	4	2	4	3	8	1	2	4	4	4	3	4
B595	2008	Zhao, Y. *et al.* & 21989209	BSP1	Sichuan	30.65	104.08	2	3	3	3	2	5	1	6	3	4	3	3	4	2	4	5	7	1	3	4	4	4	3	4
B596	2008	Zhao, Y. *et al.* & 21989209	BSPint	Sichuan	30.65	104.08	2	2	3	3	2	5	1	7	2	5	3	3	4	2	4	4	7	3	4	5	4	4	3	3
B597	2008	Zhao, Y. *et al.* & 21989209	BSP5	Sichuan	30.65	104.08	2	2	3	3	2	5	1	7	3	5	4	3	3	2	4	4	7	2	3	5	4	4	3	2
B598	2008	Zhao, Y. *et al.* & 21989209	BSP1	Sichuan	30.65	104.08	2	2	3	3	2	5	1	7	3	5	4	4	4	2	4	3	1	3	3	4	4	4	3	1
B599	2008	Zhao, Y. *et al.* & 21989209	BSP1	Sichuan	30.65	104.08	2	2	4	3	2	5	1	4	3	5	4	2	4	2	4	3	5	2	3	5	4	4	3	3
B600	2008	Zhao, Y. *et al.* & 21989209	BSP1	Sichuan	30.65	104.08	2	2	4	3	2	5	1	4	3	6	4	3	4	2	4	5	7	2	1	5	4	4	3	2
B601	2008	Zhao, Y. *et al.* & 21989209	BSP1	Sichuan	30.65	104.08	2	2	4	4	2	5	1	6	4	6	6	2	4	2	4	4	5	1	1	4	4	4	3	2
B602	2008	Zhao, Y. *et al.* & 21989209	BSP1	Sichuan	30.65	104.08	2	3	3	3	2	5	1	7	2	4	4	3	4	2	4	4	8	1	4	6	4	4	3	4
B603	2008	Zhao, Y. *et al.* & 21989209	BSP1	Sichuan	30.65	104.08	2	3	3	3	2	5	1	7	1	4	4	3	4	2	4	4	8	1	4	6	4	4	3	3
B604	2008	Zhao, Y. *et al.* & 21989209	BSP1	Sichuan	30.65	104.08	2	3	3	3	2	5	1	7	2	4	4	3	4	2	4	5	8	1	4	6	4	4	3	3
B605	2008	Zhao, Y. *et al.* & 21989209	BSP1	Sichuan	30.65	104.08	2	3	3	4	2	5	1	3	2	4	4	3	4	2	5	2	6	2	3	4	4	4	3	3
B606	2008	Zhao, Y. *et al.* & 21989209	BSP1	Sichuan	30.65	104.08	2	3	3	3	2	5	1	7	2	4	4	3	4	2	4	ND	8	2	4	4	4	4	3	3
B607	2008	Zhao, Y. *et al.* & 21989209	BSP1	Sichuan	30.65	104.08	2	3	3	3	2	5	1	5	2	4	5	2	4	2	4	4	6	1	4	4	4	4	3	3
B608	2008	Zhao, Y. *et al.* & 21989209	BSP1	Sichuan	30.65	104.08	2	3	3	3	2	5	1	7	1	5	4	1	4	2	4	5	9	1	3	4	4	4	3	4
B609	2008	Zhao, Y. *et al.* & 21989209	BSP1	Sichuan	30.65	104.08	2	3	3	3	2	5	1	7	2	4	4	3	3	2	4	3	7	0	3	5	4	4	3	3
B610	2008	Zhao, Y. *et al.* & 21989209	BSP1	Sichuan	30.65	104.08	2	3	3	3	2	5	1	4	2	4	4	2	4	2	4	3	5	0	4	5	4	4	3	3
B611	2008	Zhao, Y. *et al.* & 21989209	BSP1	Sichuan	30.65	104.08	2	3	3	3	2	5	1	4	2	2	3	3	3	2	4	5	7	2	4	4	4	4	3	4
B612	2008	Zhao, Y. *et al.* & 21989209	BSPint	Sichuan	30.65	104.08	2	3	3	3	2	5	1	7	2	4	2	3	4	2	4	6	7	2	4	4	4	4	3	3

B613	2008	Zhao, Y. *et al.* & 21989209	BSP1	Sichuan	30.65	104.08	2	3	3	3	2	5	1	6	3	4	4	5	4	2	4	5	8	1	4	6	4	4	3	4
B614	2008	Zhao, Y. *et al.* & 21989209	BSP1	Sichuan	30.65	104.08	2	3	3	3	2	5	1	7	2	4	3	5	3	2	4	5	7	2	2	7	4	2	3	4
B615	2008	Zhao, Y. *et al.* & 21989209	BSP1	Sichuan	30.65	104.08	2	3	2	4	2	5	1	6	1	4	3	4	4	2	4	4	8	4	2	6	4	4	3	4
B616	2008	Zhao, Y. *et al.* & 21989209	BSP1	Sichuan	30.65	104.08	2	3	3	3	2	5	1	7	2	5	3	5	4	2	4	7	8	4	2	4	4	4	2	5
B617	2008	Zhao, Y. *et al.* & 21989209	BSP1	Sichuan	30.65	104.08	2	3	2	3	2	5	1	7	2	4	3	5	4	2	4	6	8	3	2	6	4	4	3	4
B618	2008	Zhao, Y. *et al.* & 21989209	BSP1	Sichuan	30.65	104.08	2	3	0	3	2	5	1	7	2	4	3	5	4	2	4	6	8	4	4	5	4	4	3	5
B619	2008	Zhao, Y. *et al.* & 21989209	BSP1	Sichuan	30.65	104.08	2	3	1	3	2	5	1	6	2	4	3	5	4	2	4	6	8	5	1	7	4	4	3	4
B620	2008	Zhao, Y. *et al.* & 21989209	BSP1	Sichuan	30.65	104.08	2	3	1	3	2	5	1	7	2	5	4	4	4	2	4	6	8	2	3	5	4	4	3	5
B621	2008	Zhao, Y. *et al.* & 21989209	BSP1	Sichuan	30.65	104.08	2	3	2	2	2	5	1	6	1	5	3	5	4	2	4	5	7	1	3	6	4	4	3	4
B622	2008	Zhao, Y. *et al.* & 21989209	BSP1	Sichuan	30.65	104.08	2	4	2	1	2	5	1	5	2	4	2	3	4	2	4	6	8	1	3	5	4	4	3	4
B623	2008	Zhao, Y. *et al.* & 21989209	BSP1	Sichuan	30.65	104.08	2	3	3	2	2	5	1	6	2	4	2	3	4	2	4	ND	6	2	2	4	4	4	3	4
B624	2008	Zhao, Y. *et al.* & 21989209	BSP1	Sichuan	30.65	104.08	2	3	2	2	2	5	1	7	2	4	1	2	4	2	4	6	8	1	3	5	4	4	3	4
B625	2008	Zhao, Y. *et al.* & 21989209	BSP1	Sichuan	30.65	104.08	2	3	2	2	2	5	1	8	2	4	1	2	4	2	3	6	4	1	2	5	4	4	2	4
B626	2008	Zhao, Y. *et al.* & 21989209	BSP1	Sichuan	30.65	104.08	2	3	1	2	2	5	1	7	2	4	1	3	4	2	4	6	8	1	2	5	4	4	3	4
B627	2008	Zhao, Y. *et al.* & 21989209	BSP1	Sichuan	30.65	104.08	2	3	3	2	2	5	1	8	2	4	2	2	4	2	4	6	7	1	3	5	4	4	3	4
B628	2008	Zhao, Y. *et al.* & 21989209	BSP1	Sichuan	30.65	104.08	2	3	3	2	2	5	1	8	2	4	2	1	4	2	4	4	8	1	3	5	4	4	3	4
B629	2008	Zhao, Y. *et al.* & 21989209	BSP1	Sichuan	30.65	104.08	2	3	3	2	2	5	1	8	2	4	2	2	4	2	4	6	8	1	3	5	4	4	3	4
B630	2008	Zhao, Y. *et al.* & 21989209	BSP1	Sichuan	30.65	104.08	2	3	1	3	2	5	1	6	2	4	1	2	4	2	4	5	8	3	2	4	4	4	3	4
B631	2008	Zhao, Y. *et al.* & 21989209	BSP1	Sichuan	30.65	104.08	2	3	2	1	2	5	1	7	2	4	1	3	4	2	4	6	8	1	2	5	4	4	3	4
B632	2008	Zhao, Y. *et al.* & 21989209	BSP1	Sichuan	30.65	104.08	2	3	3	2	2	5	1	8	2	3	2	3	4	2	4	4	8	1	2	5	4	4	3	4
B633	2008	Zhao, Y. *et al.* & 21989209	BSP1	Sichuan	30.65	104.08	2	3	1	2	1	5	1	8	3	4	2	3	4	2	4	9	9	6	2	5	4	4	3	4
B634	2008	Zhao, Y. *et al.* & 21989209	BSP1	Sichuan	30.65	104.08	2	3	2	2	2	5	1	6	1	3	2	1	4	2	4	5	9	3	2	4	4	4	3	3
B635	2008	Zhao, Y. *et al.* & 21989209	BSP1	Sichuan	30.65	104.08	2	3	2	1	2	5	1	6	2	4	2	4	4	2	4	5	8	1	2	5	4	4	3	3
B636	2008	Zhao, Y. *et al.* & 21989209	BSP1	Sichuan	30.65	104.08	2	3	2	1	2	5	1	7	2	4	2	3	4	2	4	ND	5	2	2	4	4	4	3	3
B637	2008	Zhao, Y. *et al.* & 21989209	BSP1	Sichuan	30.65	104.08	2	3	2	2	2	5	1	6	1	4	2	3	4	2	4	3	8	3	3	4	4	4	3	3
B638	2008	Zhao, Y. *et al.* & 21989209	BSP1	Sichuan	30.65	104.08	2	3	2	1	2	5	1	6	1	4	2	3	4	2	4	5	9	1	3	5	4	4	3	3
B639	2008	Zhao, Y. *et al.* & 21989209	BSP1	Sichuan	30.65	104.08	2	3	2	1	2	5	1	6	1	4	2	3	4	2	4	4	8	1	3	5	4	4	3	2
B640	2008	Zhao, Y. *et al.* & 21989209	BSP1	Sichuan	30.65	104.08	2	3	2	1	2	5	1	7	1	4	2	3	4	2	4	6	ND	2	3	4	4	4	3	2
B641	2008	Zhao, Y. *et al.* & 21989209	BSP1	Sichuan	30.65	104.08	2	3	3	2	2	5	1	7	3	5	3	3	4	2	4	5	7	0	2	5	4	4	3	3
B642	2008	Zhao, Y. *et al.* & 21989209	BSP1	Sichuan	30.65	104.08	2	3	2	2	2	5	1	7	3	3	2	2	2	2	4	6	7	1	3	3	4	4	3	3
B643	2008	Zhao, Y. *et al.* & 21989209	BSP1	Sichuan	30.65	104.08	2	3	2	2	2	5	1	8	3	4	2	2	4	2	4	5	7	1	2	3	4	4	3	3
B644	2008	Zhao, Y. *et al.* & 21989209	BSP1	Sichuan	30.65	104.08	2	3	2	1	2	5	1	7	3	4	2	2	4	2	4	5	7	0	1	4	4	4	3	3
B645	2008	Zhao, Y. *et al.* & 21989209	BSP1	Sichuan	30.65	104.08	2	3	2	2	2	5	1	6	3	3	2	1	4	2	4	6	1	1	2	5	4	4	3	3
B646	2008	Zhao, Y. *et al.* & 21989209	BSP1	Sichuan	30.65	104.08	2	3	2	2	2	5	1	5	3	2	2	1	4	2	4	6	1	1	3	3	4	4	3	3
B647	2008	Zhao, Y. *et al.* & 21989209	BSP1	Sichuan	30.65	104.08	2	3	2	1	2	5	1	6	3	3	2	3	3	2	4	4	7	0	3	5	4	2	3	3
B648	2008	Zhao, Y. *et al.* & 21989209	BSP1	Sichuan	30.65	104.08	2	3	2	1	2	4	1	8	3	3	4	2	4	2	4	4	2	1	3	6	4	4	3	3
B649	2008	Zhao, Y. *et al.* & 21989209	BSP1	Sichuan	30.65	104.08	2	3	2	1	2	4	1	5	3	3	3	2	4	2	4	5	1	3	1	6	4	4	1	3
B650	2008	Zhao, Y. *et al.* & 21989209	BSP1	Sichuan	30.65	104.08	2	3	2	1	2	4	1	6	3	3	3	3	4	2	4	4	7	1	1	6	4	4	3	4
B651	2008	Zhao, Y. *et al.* & 21989209	BSP1	Sichuan	30.65	104.08	2	3	2	1	2	4	1	6	3	3	3	3	4	2	4	5	7	3	2	4	4	4	2	3
B652	2008	Zhao, Y. *et al.* & 21989209	BSP1	Sichuan	30.65	104.08	2	3	2	0	2	4	1	7	3	3	3	3	4	2	4	4	7	1	1	5	4	4	3	4
B653	2008	Zhao, Y. *et al.* & 21989209	BSP1	Sichuan	30.65	104.08	2	3	2	1	2	4	1	7	3	3	3	3	2	2	4	5	7	3	2	4	4	4	3	3

B654	2008	Zhao, Y. *et al.* & 21989209	BSP1	Sichuan	30.65	104.08	2	3	2	1	2	4	1	7	3	4	3	3	4	2	4	4	8	1	1	5	4	4	3	4
B655	2008	Zhao, Y. *et al.* & 21989209	BSP1	Sichuan	30.65	104.08	2	1	2	1	2	4	1	7	3	4	3	3	4	2	4	2	7	1	2	5	4	4	3	4
B656	2008	Zhao, Y. *et al.* & 21989209	BSP1	Sichuan	30.65	104.08	2	2	2	2	2	4	1	7	3	5	3	3	4	2	4	5	7	3	2	4	4	4	3	5
B657	2008	Zhao, Y. *et al.* & 21989209	BSP1	Sichuan	30.65	104.08	2	2	2	2	2	4	1	6	3	3	3	3	4	2	4	4	7	1	1	5	4	4	3	4
B658	2008	Zhao, Y. *et al.* & 21989209	BSP1	Sichuan	30.65	104.08	2	3	2	4	2	5	1	6	3	4	3	3	4	2	4	4	7	1	1	7	4	4	3	4
B659	2008	Zhao, Y. *et al.* & 21989209	BSP1	Sichuan	30.65	104.08	2	3	3	3	2	5	1	6	2	3	3	2	4	2	4	5	7	3	3	4	4	4	3	4
B660	2008	Zhao, Y. *et al.* & 21989209	BSP1	Sichuan	30.65	104.08	2	3	3	3	2	5	1	6	3	3	3	3	3	2	4	4	6	1	2	5	4	2	3	4
B661	2008	Zhao, Y. *et al.* & 21989209	BSP5	Sichuan	30.65	104.08	2	2	3	3	2	5	1	7	3	4	3	3	4	2	4	4	7	2	4	5	4	4	3	3
B662	2008	Zhao, Y. *et al.* & 21989209	BSP5	Sichuan	30.65	104.08	2	2	4	3	2	5	1	7	3	5	4	3	3	2	4	4	6	2	4	6	4	2	3	3
B663	2008	Zhao, Y. *et al.* & 21989209	BSPint	Sichuan	30.65	104.08	2	2	3	3	2	5	1	6	3	5	4	3	4	2	4	5	7	7	3	3	4	4	3	3
B664	2008	Zhao, Y. *et al.* & 21989209	BSP1	Sichuan	30.65	104.08	2	2	2	3	2	5	1	6	3	4	3	3	4	2	4	5	7	5	1	6	4	4	3	2
B665	2008	Zhao, Y. *et al.* & 21989209	BSP1	Sichuan	30.65	104.08	2	2	3	4	2	5	1	8	4	6	5	3	4	2	4	4	7	2	2	5	6	4	3	3
B666	2008	Zhao, Y. *et al.* & 21989209	BSP1	Sichuan	30.65	104.08	2	3	3	3	1	5	1	7	2	4	4	3	4	2	4	2	8	1	4	6	4	4	3	4
B667	2008	Zhao, Y. *et al.* & 21989209	BSP1	Sichuan	30.65	104.08	2	3	3	3	2	5	1	7	2	4	4	3	3	2	4	4	8	1	4	6	4	4	2	3
B668	2008	Zhao, Y. *et al.* & 21989209	BSP1	Sichuan	30.65	104.08	2	3	3	3	2	5	1	7	2	4	4	3	4	2	4	6	8	2	4	4	4	4	2	4
B669	2009	Zhao, Y. *et al.* & 21989209	BSP1	Sichuan	30.65	104.08	2	3	3	3	2	5	1	6	3	3	4	2	4	2	4	5	7	1	2	5	4	4	3	4
B670	2009	Zhao, Y. *et al.* & 21989209	BSP1	Sichuan	30.65	104.08	2	3	2	2	2	5	1	7	3	4	3	3	4	2	4	5	7	2	4	4	4	4	3	4
B671	2009	Zhao, Y. *et al.* & 21989209	BSP1	Sichuan	30.65	104.08	2	3	2	2	2	5	1	5	2	5	2	3	4	2	4	6	8	3	3	4	4	4	3	5
B672	2009	Zhao, Y. *et al.* & 21989209	BSP1	Sichuan	30.65	104.08	2	1	2	2	2	5	1	6	1	4	1	3	4	2	4	9	8	1	1	4	4	4	3	4
B673	2009	Zhao, Y. *et al.* & 21989209	BSP1	Sichuan	30.65	104.08	2	3	1	2	2	5	1	6	1	5	1	3	4	2	4	8	8	1	1	4	4	4	3	4
B674	2009	Zhao, Y. *et al.* & 21989209	BSP1	Sichuan	30.65	104.08	2	3	2	2	2	5	1	4	1	4	1	3	4	2	4	ND	9	1	1	3	4	4	3	3
B675	2009	Zhao, Y. *et al.* & 21989209	BSP1	Sichuan	30.65	104.08	2	3	2	2	2	5	1	6	1	4	1	3	4	2	4	ND	8	1	1	4	4	4	3	4
B676	2009	Zhao, Y. *et al.* & 21989209	BSP1	Sichuan	30.65	104.08	2	3	2	2	2	5	1	6	1	5	1	3	4	2	4	9	8	1	1	4	4	4	3	4
B677	2009	Zhao, Y. *et al.* & 21989209	BSP1	Sichuan	30.65	104.08	2	3	2	2	2	5	1	5	1	4	1	3	4	2	4	7	9	1	1	6	4	4	3	3
B678	2009	Zhao, Y. *et al.* & 21989209	BSP1	Sichuan	30.65	104.08	2	3	2	2	2	5	1	5	2	2	2	1	4	2	4	6	2	4	2	4	4	4	2	4
B679	2009	Zhao, Y. *et al.* & 21989209	BSP1	Sichuan	30.65	104.08	2	3	3	2	2	5	1	6	3	5	4	3	4	2	4	5	9	1	2	3	4	4	3	4
B680	2009	Zhao, Y. *et al.* & 21989209	BSP1	Sichuan	30.65	104.08	2	3	3	2	2	5	1	6	3	5	3	3	4	2	4	6	ND	2	3	3	4	4	3	4
B681	2009	Zhao, Y. *et al.* & 21989209	BSP1	Sichuan	30.65	104.08	2	3	3	2	2	5	1	5	3	4	4	2	4	2	4	4	8	2	3	4	4	4	3	4
B682	2009	Zhao, Y. *et al.* & 21989209	BSP1	Sichuan	30.65	104.08	2	3	1	2	2	5	1	6	3	4	4	3	3	2	4	3	8	2	3	3	4	3	3	4
B683	2009	Zhao, Y. *et al.* & 21989209	BSP1	Sichuan	30.65	104.08	2	4	3	3	2	5	1	6	3	5	4	3	4	2	4	5	7	1	3	5	4	4	3	4
B684	2009	Zhao, Y. *et al.* & 21989209	BSP1	Sichuan	30.65	104.08	2	3	3	2	2	5	1	6	3	4	3	3	4	2	4	3	8	1	2	5	4	4	3	4
B685	2009	Zhao, Y. *et al.* & 21989209	BSP1	Sichuan	30.65	104.08	2	3	2	2	2	5	1	6	3	5	4	3	4	2	4	5	9	1	2	3	4	4	3	4
B686	2009	Zhao, Y. *et al.* & 21989209	BSP1	Sichuan	30.65	104.08	2	3	3	2	2	5	1	6	3	4	4	3	4	2	4	5	9	1	3	3	4	4	3	4
B687	2009	Zhao, Y. *et al.* & 21989209	BSP1	Sichuan	30.65	104.08	2	3	3	2	2	5	1	7	4	4	3	3	4	2	5	2	5	2	3	4	4	4	3	5
B688	2009	Zhao, Y. *et al.* & 21989209	BSP1	Sichuan	30.65	104.08	2	3	3	2	2	5	1	4	3	4	3	3	3	2	4	5	7	1	3	5	4	4	3	4
B689	2009	Zhao, Y. *et al.* & 21989209	BSP1	Sichuan	30.65	104.08	2	3	3	2	2	5	1	6	3	4	3	3	4	2	5	5	7	1	3	5	4	4	3	4
B690	2009	Zhao, Y. *et al.* & 21989209	BSP1	Sichuan	30.65	104.08	2	3	3	2	2	5	1	6	3	4	3	3	2	2	4	4	7	2	3	4	4	4	3	4
B691	2009	Zhao, Y. *et al.* & 21989209	BSP1	Sichuan	30.65	104.08	2	3	3	2	2	5	1	7	3	4	3	3	4	2	4	5	7	1	3	5	4	4	3	4
B692	2009	Zhao, Y. *et al.* & 21989209	BSP1	Sichuan	30.65	104.08	2	3	3	2	2	5	1	7	3	4	3	3	4	2	4	5	5	1	3	5	4	4	3	4
B693	2009	Zhao, Y. *et al.* & 21989209	BSP1	Sichuan	30.65	104.08	2	3	3	3	2	5	1	6	3	4	3	4	4	2	4	6	8	3	3	5	4	4	3	6
B694	2009	Zhao, Y. *et al.* & 21989209	BSP1	Sichuan	30.65	104.08	2	3	3	3	2	5	1	6	3	4	2	3	4	2	4	5	8	1	3	5	4	4	3	5

B695	2009	Zhao, Y. *et al.* & 21989209	BSP1	Sichuan	30.65	104.08	2	3	3	3	2	5	1	6	3	4	2	3	4	2	4	5	6	1	3	5	4	4	3	4
B696	2009	Zhao, Y. *et al.* & 21989209	BSP1	Sichuan	30.65	104.08	2	3	3	3	2	5	1	6	3	4	2	3	4	2	4	5	6	1	3	5	4	4	3	4
B697	2009	Zhao, Y. *et al.* & 21989209	BSP1	Sichuan	30.65	104.08	2	3	4	3	2	5	1	7	3	5	4	3	5	2	4	6	8	1	4	6	4	4	3	4
B698	2009	Zhao, Y. *et al.* & 21989209	BSP1	Sichuan	30.65	104.08	2	3	3	3	2	5	1	7	3	4	4	3	5	2	4	6	9	2	8	3	4	4	3	4
B699	2009	Zhao, Y. *et al.* & 21989209	BSP1	Sichuan	30.65	104.08	2	3	3	3	2	5	1	8	3	4	4	3	3	2	4	5	7	1	2	5	4	4	3	4
B700	2009	Zhao, Y. *et al.* & 21989209	BSP1	Sichuan	30.65	104.08	2	3	3	3	2	5	1	6	3	4	4	3	4	2	3	5	5	1	2	7	4	2	3	4
B701	2009	Zhao, Y. *et al.* & 21989209	BSP1	Sichuan	30.65	104.08	2	3	3	4	2	5	1	6	3	3	3	3	3	2	4	5	7	1	2	5	4	4	3	4
B702	2009	Zhao, Y. *et al.* & 21989209	BSP1	Sichuan	30.65	104.08	2	3	3	4	2	5	1	7	3	4	5	3	4	2	4	5	6	2	3	4	4	4	3	3
B703	2009	Zhao, Y. *et al.* & 21989209	BSP1	Sichuan	30.65	104.08	2	3	2	4	2	5	1	6	3	3	3	3	4	2	4	5	7	1	3	4	4	4	3	3
B704	2009	Zhao, Y. *et al.* & 21989209	BSP1	Sichuan	30.65	104.08	2	3	2	3	2	5	1	6	3	3	3	3	4	2	4	4	7	2	3	3	4	4	3	4
B705	2009	Zhao, Y. *et al.* & 21989209	BSP1	Sichuan	30.65	104.08	2	3	2	4	2	5	1	6	3	3	3	3	3	2	4	5	7	2	3	4	4	4	3	3
B706	2009	Zhao, Y. *et al.* & 21989209	BSP1	Sichuan	30.65	104.08	2	1	3	3	2	5	1	6	3	3	3	3	4	2	4	4	6	1	3	4	4	4	3	3
B707	2009	Zhao, Y. *et al.* & 21989209	BSP1	Sichuan	30.65	104.08	2	3	3	4	2	5	1	6	3	3	3	3	4	2	5	5	7	1	3	4	4	4	3	3
B708	2009	Zhao, Y. *et al.* & 21989209	BSP1	Sichuan	30.65	104.08	2	3	2	3	2	6	1	6	3	1	3	2	4	2	4	7	2	5	3	3	4	4	3	3
B709	2009	Zhao, Y. *et al.* & 21989209	BSP1	Sichuan	30.65	104.08	2	3	2	3	2	5	1	7	3	3	3	4	3	2	4	4	7	1	4	5	4	2	3	2
B710	2009	Zhao, Y. *et al.* & 21989209	BSP1	Sichuan	30.65	104.08	2	3	2	2	2	5	1	7	2	4	2	3	4	2	4	3	7	1	2	5	4	4	2	4
B711	2009	Zhao, Y. *et al.* & 21989209	BSP1	Sichuan	30.65	104.08	2	3	2	2	2	5	1	7	1	6	2	3	4	2	5	3	5	3	2	4	4	4	3	5
B712	2009	Zhao, Y. *et al.* & 21989209	BSP1	Sichuan	30.65	104.08	2	3	2	2	2	5	1	6	2	5	1	3	4	2	3	8	7	2	1	4	4	4	3	4
B713	2009	Zhao, Y. *et al.* & 21989209	BSP1	Sichuan	30.65	104.08	2	3	1	2	2	5	1	5	1	4	1	2	4	1	4	9	9	1	1	4	4	4	3	4
B714	2009	Zhao, Y. *et al.* & 21989209	BSP1	Sichuan	30.65	104.08	2	3	2	2	2	5	1	6	1	4	1	3	3	2	4	6	7	1	2	4	4	4	3	4
B715	2009	Zhao, Y. *et al.* & 21989209	BSP1	Sichuan	30.65	104.08	2	3	2	2	2	5	1	6	1	4	1	3	4	3	4	6	8	1	2	5	4	4	3	4
B716	2009	Zhao, Y. *et al.* & 21989209	BSP1	Sichuan	30.65	104.08	2	3	2	2	2	5	1	6	2	3	2	2	4	2	4	6	7	3	1	4	4	4	3	4
B717	2009	Zhao, Y. *et al.* & 21989209	BSP1	Sichuan	30.65	104.08	2	3	2	2	2	5	1	4	2	3	3	2	4	2	4	6	9	1	4	4	4	4	3	3
B718	2009	Zhao, Y. *et al.* & 21989209	BSP1	Sichuan	30.65	104.08	2	3	3	3	2	5	1	7	3	5	4	3	3	2	4	4	7	1	4	6	4	2	3	4
B719	2009	Zhao, Y. *et al.* & 21989209	BSP1	Sichuan	30.65	104.08	2	3	3	2	2	5	1	6	3	4	4	3	4	2	4	5	3	1	3	5	4	4	3	4
B720	2009	Zhao, Y. *et al.* & 21989209	BSP1	Sichuan	30.65	104.08	2	3	3	2	2	5	1	7	3	4	3	3	4	2	4	5	7	1	3	5	4	4	3	4
B721	2009	Zhao, Y. *et al.* & 21989209	BSP1	Sichuan	30.65	104.08	2	3	3	2	2	5	1	5	3	4	4	4	4	2	4	6	9	1	3	4	4	4	3	4
B722	2009	Zhao, Y. *et al.* & 21989209	BSP1	Sichuan	30.65	104.08	2	3	3	3	2	5	1	6	3	4	3	3	4	2	4	4	8	1	3	5	4	4	3	5
B723	2009	Zhao, Y. *et al.* & 21989209	BSP1	Sichuan	30.65	104.08	2	3	3	3	2	5	1	6	3	4	3	3	4	2	4	6	8	3	3	4	4	4	3	5
B724	2009	Zhao, Y. *et al.* & 21989209	BSP1	Sichuan	30.65	104.08	2	3	3	3	2	5	1	6	3	3	3	3	4	2	4	4	8	1	3	5	4	4	3	5
B725	2009	Zhao, Y. *et al.* & 21989209	BSP1	Sichuan	30.65	104.08	2	3	3	3	2	5	1	4	3	4	3	3	4	2	4	5	9	1	3	4	4	4	3	5
B726	2009	Zhao, Y. *et al.* & 21989209	BSP1	Sichuan	30.65	104.08	2	3	3	3	2	5	1	6	3	3	3	3	4	2	4	3	6	2	2	4	4	4	3	5
B727	2009	Zhao, Y. *et al.* & 21989209	BSP1	Sichuan	30.65	104.08	2	3	3	3	2	5	1	6	3	4	3	4	3	2	4	3	7	1	1	5	4	4	3	5
B728	2009	Zhao, Y. *et al.* & 21989209	BSP1	Sichuan	30.65	104.08	2	3	3	3	2	5	1	6	3	4	4	3	4	2	4	5	3	1	3	5	4	4	3	5
B729	2009	Zhao, Y. *et al.* & 21989209	BSP1	Sichuan	30.65	104.08	2	3	3	3	2	5	1	4	3	4	3	3	4	2	4	5	9	1	4	4	4	4	2	4
B730	2009	Zhao, Y. *et al.* & 21989209	BSP1	Sichuan	30.65	104.08	2	3	3	3	2	5	1	4	3	4	3	3	4	2	4	5	9	1	4	4	4	4	2	4
B731	2009	Zhao, Y. *et al.* & 21989209	BSP1	Sichuan	30.65	104.08	2	3	3	3	2	5	1	6	3	4	2	3	4	2	4	3	6	1	3	5	4	4	3	5
B732	2009	Zhao, Y. *et al.* & 21989209	BSP1	Sichuan	30.65	104.08	2	3	3	3	2	5	1	8	3	4	5	3	4	2	4	5	6	3	3	4	4	4	3	4
B733	2009	Zhao, Y. *et al.* & 21989209	BSP1	Sichuan	30.65	104.08	2	3	3	3	2	5	1	6	3	4	2	3	4	2	4	5	6	1	3	5	4	4	3	5
B734	2009	Zhao, Y. *et al.* & 21989209	BSP1	Sichuan	30.65	104.08	2	3	3	3	2	6	1	5	3	4	3	3	4	2	3	5	8	2	2	5	4	4	3	5
B735	2009	Zhao, Y. *et al.* & 21989209	BSP1	Sichuan	30.65	104.08	2	3	3	3	2	5	1	6	3	4	2	3	4	2	4	5	7	1	3	4	4	4	3	4

B736	2009	Zhao, Y. *et al.* & 21989209	BSP1	Sichuan	30.65	104.08	2	3	4	3	2	5	1	8	3	4	4	3	5	2	4	4	8	1	4	6	4	4	3	4
B737	2009	Zhao, Y. *et al.* & 21989209	BSP1	Sichuan	30.65	104.08	2	3	4	3	2	5	1	8	3	5	4	3	3	2	4	4	8	1	4	6	4	2	3	4
B738	2009	Zhao, Y. *et al.* & 21989209	BSP1	Sichuan	30.65	104.08	2	3	5	3	2	5	1	7	3	4	4	3	5	2	4	4	8	1	4	9	4	4	3	4
B739	2009	Zhao, Y. *et al.* & 21989209	BSP1	Sichuan	30.65	104.08	2	3	4	3	2	5	1	7	3	4	4	3	4	2	4	4	8	1	2	6	4	4	2	4
B740	2009	Zhao, Y. *et al.* & 21989209	BSP1	Sichuan	30.65	104.08	2	3	4	3	2	5	1	6	3	4	4	3	5	2	4	6	8	2	4	6	2	4	3	4
B741	2009	Zhao, Y. *et al.* & 21989209	BSP1	Sichuan	30.65	104.08	2	2	4	4	2	5	1	7	3	4	4	3	5	2	4	6	8	1	2	7	4	4	3	4
B742	2009	Zhao, Y. *et al.* & 21989209	BSP1	Sichuan	30.65	104.08	2	3	3	3	2	5	1	7	3	4	3	3	4	2	4	2	8	1	4	6	4	4	3	4
B743	2009	Zhao, Y. *et al.* & 21989209	BSPint	Sichuan	30.65	104.08	2	3	3	3	1	5	1	7	3	4	3	3	4	3	4	4	7	3	4	3	4	4	3	4
B744	2009	Zhao, Y. *et al.* & 21989209	BSP1	Sichuan	30.65	104.08	2	3	2	3	2	5	1	6	3	5	4	3	4	2	4	5	8	1	4	6	4	4	3	4
B745	2009	Zhao, Y. *et al.* & 21989209	BSP1	Sichuan	30.65	104.08	2	3	3	3	2	5	1	7	3	4	4	3	4	2	4	5	9	1	4	6	4	4	3	4
B746	2009	Zhao, Y. *et al.* & 21989209	BSP1	Sichuan	30.65	104.08	2	3	3	3	2	5	1	5	3	4	5	2	4	2	4	4	5	1	4	4	4	4	3	4
B747	2009	Zhao, Y. *et al.* & 21989209	BSP1	Sichuan	30.65	104.08	2	3	3	3	2	5	1	7	3	1	4	3	4	2	4	5	8	1	4	5	4	3	3	4
B748	2009	Zhao, Y. *et al.* & 21989209	BSP1	Sichuan	30.65	104.08	2	3	3	3	2	5	1	7	3	4	4	3	4	2	4	5	8	1	4	5	4	4	3	4
B749	2009	Zhao, Y. *et al.* & 21989209	BSP1	Sichuan	30.65	104.08	2	3	3	3	2	5	1	7	3	2	4	3	4	2	4	4	8	1	3	8	4	4	3	3
B750	2010	This study	BSP1	Sichuan	30.65	104.08	2	3	0	3	1	5	1	6	2	3	2	3	4	2	4	5	7	2	3	5	4	4	3	4
B751	2010	This study	BSP1	Sichuan	30.65	104.08	2	3	1	3	1	5	1	6	2	3	2	3	4	2	4	2	7	2	3	7	4	2	3	4
B752	2010	This study	BSP1	Sichuan	30.65	104.08	2	3	1	3	1	5	1	7	2	3	2	3	4	2	4	5	6	3	3	4	4	4	3	4
B753	2010	This study	BSP1	Sichuan	30.65	104.08	2	3	1	3	1	5	1	8	2	3	2	3	4	2	4	4	7	2	3	4	4	2	3	4
B754	2010	This study	BSP1	Sichuan	30.65	104.08	2	3	1	3	1	5	1	7	2	2	2	3	4	2	4	4	7	2	3	4	4	2	3	4
B755	2010	This study	BSP1	Sichuan	30.65	104.08	2	3	1	3	1	5	1	7	2	3	2	3	3	2	4	4	7	2	3	4	4	3	3	4
B756	2010	This study	BSP1	Sichuan	30.65	104.08	2	3	1	3	1	5	1	7	2	3	2	2	4	2	4	6	8	3	2	3	4	4	3	4
B757	2010	This study	BSP1	Sichuan	30.65	104.08	2	3	1	3	1	5	1	5	2	3	2	2	4	2	4	5	7	1	3	3	4	2	3	4
B758	2010	This study	BSP1	Sichuan	30.65	104.08	2	3	1	3	1	5	1	6	2	2	2	2	4	2	4	ND	4	2	3	3	4	4	3	6
B759	2010	This study	BSP1	Sichuan	30.65	104.08	2	3	1	3	1	5	1	7	2	2	2	2	4	2	4	4	6	1	2	4	4	3	3	4
B760	2010	This study	BSP1	Sichuan	30.65	104.08	2	3	1	3	1	5	1	7	2	4	2	2	3	2	4	5	6	3	2	3	4	4	3	6
B761	2010	This study	BSP1	Sichuan	30.65	104.08	2	3	1	2	1	5	1	6	0	3	2	0	4	2	4	4	6	2	2	3	4	4	3	4
B762	2010	This study	BSP1	Sichuan	30.65	104.08	2	3	1	3	1	5	1	7	2	3	2	2	4	2	4	7	6	1	3	4	4	4	3	4
B763	2010	This study	BSP1	Sichuan	30.65	104.08	2	3	0	3	0	5	1	7	2	3	2	2	4	2	4	3	5	2	2	3	4	4	3	3
B764	2010	This study	BSP1	Sichuan	30.65	104.08	2	3	0	3	1	5	1	7	2	3	2	2	4	2	4	6	6	1	3	5	5	4	3	4
B765	2010	This study	BSP1	Sichuan	30.65	104.08	2	3	1	3	1	5	1	7	1	3	3	2	3	2	4	5	6	1	3	5	4	4	3	4
B766	2010	This study	BSP1	Sichuan	30.65	104.08	2	3	1	3	1	5	1	7	2	3	2	2	3	2	4	5	5	1	3	5	4	4	3	4
B767	2010	This study	BSP1	Sichuan	30.65	104.08	2	3	1	3	1	5	1	7	2	3	2	2	4	2	4	6	6	1	3	4	4	4	3	4
B768	2010	This study	BSP1	Sichuan	30.65	104.08	2	3	0	2	1	5	1	5	2	3	2	3	4	2	4	7	7	3	3	3	4	4	3	4
B769	2010	This study	BSP1	Sichuan	30.65	104.08	2	3	2	1	1	5	1	6	2	4	2	2	4	2	4	5	7	0	2	5	4	4	3	5
B770	2010	This study	BSP1	Sichuan	30.65	104.08	2	3	2	2	1	5	1	6	2	4	2	2	4	2	4	2	7	0	1	5	4	4	3	5
B771	2010	This study	BSP1	Sichuan	30.65	104.08	2	3	2	2	1	5	1	4	1	5	2	2	4	2	4	2	7	1	0	4	2	4	3	4
B772	2010	This study	BSP1	Sichuan	30.65	104.08	2	3	1	2	1	5	1	6	2	5	2	2	4	2	4	6	6	0	2	5	4	4	3	4
B773	2010	This study	BSP1	Sichuan	30.65	104.08	2	3	1	2	1	5	1	6	2	5	2	2	4	2	4	5	6	0	2	5	4	4	3	4
B774	2010	This study	BSP1	Sichuan	30.65	104.08	2	3	2	4	1	5	1	6	2	3	2	2	4	2	4	5	5	1	2	5	4	4	3	4
B775	2010	This study	BSP1	Sichuan	30.65	104.08	2	3	2	2	1	5	1	7	2	4	2	2	4	2	4	5	4	2	2	5	4	4	3	4
B776	2010	This study	BSP1	Sichuan	30.65	104.08	2	3	2	1	1	5	1	7	2	4	1	2	4	2	3	5	6	1	2	3	4	4	3	4
B777	2010	This study	BSP1	Sichuan	30.65	104.08	2	3	2	2	1	5	1	6	2	4	1	2	4	2	4	5	7	0	1	5	4	4	3	4
B778	2010	This study	BSP1	Sichuan	30.65	104.08	2	3	2	2	1	5	1	6	2	1	1	2	4	2	4	5	5	2	2	4	4	4	3	4
B779	2010	This study	BSP1	Sichuan	30.65	104.08	2	3	2	2	1	5	1	6	2	4	1	2	4	2	4	6	6	2	2	3	4	4	3	4
B780	2010	This study	BSP1	Sichuan	30.65	104.08	2	3	2	1	2	5	1	7	2	6	1	3	4	2	4	5	4	2	2	4	4	4	3	4

B781	2010	This study	BSP1	Sichuan	30.65	104.08	2	3	2	2	1	5	1	6	2	6	1	2	4	2	4	4	6	1	2	7	4	4	3	3
B782	2010	This study	BSP1	Sichuan	30.65	104.08	2	3	2	2	1	5	1	3	2	5	0	2	4	2	4	5	7	1	2	6	4	4	3	3
B783	2010	This study	BSP1	Sichuan	30.65	104.08	2	3	2	2	1	5	1	6	2	6	2	3	4	2	5	5	7	2	2	5	4	4	3	4
B784	2010	This study	BSP1	Sichuan	30.65	104.08	2	3	2	2	1	5	1	4	2	5	2	3	4	2	4	2	5	2	2	5	4	4	3	5
B785	2010	This study	BSP1	Sichuan	30.65	104.08	2	3	2	2	1	5	1	6	2	4	2	3	4	2	4	4	7	1	0	6	4	4	3	4
B786	2010	This study	BSP1	Sichuan	30.65	104.08	2	3	2	2	1	5	1	6	2	5	2	3	4	2	4	4	7	1	2	6	4	4	3	4
B787	2010	This study	BSP1	Sichuan	30.65	104.08	2	3	2	2	1	5	1	5	2	6	1	3	4	2	4	4	5	1	2	6	4	4	3	4
B788	2010	This study	BSP1	Sichuan	30.65	104.08	2	3	2	2	1	5	1	6	2	6	2	3	3	2	4	ND	7	1	2	6	4	4	3	4
B789	2010	This study	BSP1	Sichuan	30.65	104.08	2	3	2	2	1	9	1	7	2	3	1	3	4	2	4	3	7	2	3	4	4	4	3	2
B790	2010	This study	BSP1	Sichuan	30.65	104.08	2	3	2	2	2	9	1	7	2	4	3	3	4	2	5	5	3	1	2	5	4	4	3	4
B791	2010	This study	BSP1	Sichuan	30.65	104.08	2	3	1	2	2	9	1	7	2	4	1	3	4	2	4	5	8	1	2	5	4	4	3	4
B792	2010	This study	BSP1	Sichuan	30.65	104.08	2	3	2	2	2	9	1	7	2	4	2	3	4	2	4	5	7	2	2	4	4	4	3	4
B793	2010	This study	BSP1	Sichuan	30.65	104.08	2	3	2	2	3	9	1	7	2	4	2	3	4	2	4	5	6	3	2	4	4	4	3	4
B794	2010	This study	BSP1	Sichuan	30.65	104.08	2	3	2	1	3	9	1	7	2	4	2	3	4	2	4	5	8	3	2	4	4	4	3	3
B795	2010	This study	BSP1	Sichuan	30.65	104.08	2	3	2	2	3	8	1	6	2	4	1	2	4	2	4	4	8	3	3	4	4	4	3	4
B796	2010	This study	BSP1	Sichuan	30.65	104.08	2	3	2	2	2	9	1	7	2	4	2	2	4	2	4	5	8	1	3	5	3	4	3	3
B797	2010	This study	BSP1	Sichuan	30.65	104.08	2	1	2	2	3	9	1	7	2	4	1	3	4	3	4	4	8	1	2	5	4	4	3	3
B798	2010	This study	BSP1	Sichuan	30.65	104.08	2	2	2	1	2	9	1	7	2	4	1	3	4	2	4	2	8	1	2	4	4	4	3	4
B799	2010	This study	BSP1	Sichuan	30.65	104.08	2	3	2	2	2	ND	1	ND	2	4	1	3	4	2	4	5	7	3	ND	4	4	4	3	3
B800	2010	This study	BSP1	Sichuan	30.65	104.08	2	3	2	1	1	9	1	7	2	4	1	3	2	2	4	4	7	3	3	3	4	4	3	3
B801	2010	This study	BSP1	Sichuan	30.65	104.08	2	3	2	2	2	9	1	7	2	3	1	3	2	2	4	6	8	4	3	4	4	4	3	4
B802	2010	This study	BSP1	Sichuan	30.65	104.08	2	3	1	1	3	7	1	5	2	2	0	3	1	1	4	2	5	2	0	3	4	2	3	4
B803	2010	This study	BSP1	Sichuan	30.65	104.08	2	3	2	2	2	9	1	9	2	3	0	3	4	2	4	5	7	1	2	5	4	2	3	4
B804	2010	This study	BSP1	Sichuan	30.65	104.08	2	3	2	2	3	9	1	8	2	4	1	4	4	2	4	1	6	4	ND	4	4	4	3	5
B805	2011	This study	BSP1	Sichuan	30.65	104.08	2	3	2	2	2	9	1	7	2	6	0	4	3	2	4	6	7	4	ND	3	4	4	3	5
B806	2011	This study	BSP1	Sichuan	30.65	104.08	2	3	2	2	2	9	1	7	2	3	1	4	4	2	5	5	3	1	2	5	4	4	3	3
B807	2011	This study	BSP1	Sichuan	30.65	104.08	2	3	1	2	2	7	1	5	2	1	0	7	3	1	3	2	4	1	0	2	4	2	3	5
B808	2010	This study	BSP1	Sichuan	30.65	104.08	2	5	3	1	2	5	1	7	3	3	4	4	4	2	4	4	8	2	4	5	4	4	3	4
B809	2010	This study	BSP1	Sichuan	30.65	104.08	2	5	4	2	2	5	1	8	2	4	3	2	4	2	4	4	9	3	3	4	4	4	3	6
B810	2010	This study	BSP1	Sichuan	30.65	104.08	2	5	4	3	2	5	1	8	2	4	5	4	3	2	4	3	8	2	4	5	4	4	3	6
B811	2010	This study	BSP1	Sichuan	30.65	104.08	2	5	4	2	2	5	1	8	3	4	5	4	4	2	4	5	7	5	3	4	4	4	3	6
B812	2010	This study	BSP1	Sichuan	30.65	104.08	2	5	4	3	2	5	1	8	3	4	5	4	4	2	4	7	6	5	6	4	4	4	3	5
B813	2010	This study	BSP1	Sichuan	30.65	104.08	2	5	4	2	2	5	1	8	3	4	5	4	4	2	4	1	8	2	3	5	4	4	3	6
B814	2010	This study	BSP1	Sichuan	30.65	104.08	2	5	4	2	2	5	0	8	3	4	4	5	4	2	4	3	7	2	4	ND	4	4	3	5
B815	2010	This study	BSP1	Sichuan	30.65	104.08	2	5	3	2	2	5	0	6	2	3	4	3	4	2	4	ND	7	1	1	4	4	4	3	4
B816	2010	This study	BSP1	Sichuan	30.65	104.08	2	5	4	2	2	2	0	8	2	4	4	3	4	2	4	2	7	2	4	ND	4	4	3	4
B817	2010	This study	BSP1	Sichuan	30.65	104.08	2	5	4	2	2	5	0	8	2	3	4	4	4	2	4	4	6	2	4	5	4	4	3	4
B818	2010	This study	BSP1	Sichuan	30.65	104.08	2	5	3	ND	1	5	0	8	2	3	5	4	4	2	4	3	6	4	4	4	4	4	3	4
B819	2010	This study	BSP1	Sichuan	30.65	104.08	2	5	4	3	2	5	0	8	1	4	4	4	4	2	4	3	4	2	4	5	4	4	3	7
B820	2010	This study	BSP1	Sichuan	30.65	104.08	2	5	4	3	2	5	0	8	2	4	4	4	4	2	4	5	6	4	4	4	4	4	3	5
B821	2010	This study	BSP1	Sichuan	30.65	104.08	2	5	3	1	2	5	0	7	2	2	2	4	4	2	4	3	5	3	2	4	4	4	3	3
B822	2010	This study	BSP1	Sichuan	30.65	104.08	2	6	4	2	2	5	0	8	2	2	3	4	4	2	4	4	8	2	4	4	4	4	3	5
B823	2010	This study	BSP1	Sichuan	30.65	104.08	2	5	3	2	2	5	0	8	2	3	4	3	4	2	4	4	8	2	4	4	4	4	3	7
B824	2010	This study	BSP1	Sichuan	30.65	104.08	2	5	4	2	2	5	0	6	2	3	4	4	4	2	4	4	9	2	4	4	4	4	3	8
B825	2010	This study	BSP1	Sichuan	30.65	104.08	2	5	4	1	2	5	1	6	2	2	3	4	4	2	4	3	ND	5	3	6	4	4	3	5
B826	2010	This study	BSP1	Sichuan	30.65	104.08	2	3	3	3	2	5	1	6	2	5	3	3	4	2	4	5	3	1	3	5	4	4	3	4
B827	2010	This study	BSP1	Sichuan	30.65	104.08	2	3	1	3	2	5	1	1	2	3	1	4	2	1	4	3	7	2	0	3	4	2	3	8
B828	2010	This study	BSP1	Sichuan	30.65	104.08	2	4	2	3	2	5	1	3	2	3	1	2	3	1	4	2	8	1	0	3	3	2	3	4

B829	2010	This study	BSP1	Sichuan	30.65	104.08	2	3	3	3	2	5	1	6	2	6	2	3	ND	2	4	3	9	1	3	5	4	4	3	5
B830	2010	This study	BSP1	Sichuan	30.65	104.08	2	3	3	3	2	5	1	6	2	5	1	3	4	2	4	6	8	1	3	6	4	4	3	4
B831	2010	This study	BSP1	Sichuan	30.65	104.08	2	3	3	3	3	5	1	6	2	6	2	3	3	1	4	5	8	1	2	5	4	4	3	5
B832	2010	This study	BSPint	Sichuan	30.65	104.08	2	3	3	3	2	5	1	7	2	6	1	3	4	2	4	7	7	4	ND	4	4	4	3	4
B833	2010	This study	BSP1	Sichuan	30.65	104.08	2	3	3	4	1	5	1	6	2	5	1	5	4	2	4	4	7	2	1	3	4	2	3	6
B834	2010	This study	BSP1	Sichuan	30.65	104.08	2	3	3	3	2	5	1	6	2	6	2	3	4	2	4	6	9	2	1	5	4	4	3	5
B835	2010	This study	BSP1	Sichuan	30.65	104.08	2	3	3	3	2	5	1	6	2	7	2	ND	4	2	4	5	7	2	3	5	4	4	3	6
B836	2010	This study	BSP1	Sichuan	30.65	104.08	2	3	3	3	2	5	1	3	2	6	2	3	4	2	4	7	8	4	3	4	4	4	3	5
B837	2010	This study	BSP1	Sichuan	30.65	104.08	2	3	3	3	2	5	1	6	2	6	2	3	4	2	4	6	8	4	3	4	4	4	3	6
B838	2010	This study	BSP1	Sichuan	30.65	104.08	2	3	3	3	2	5	1	6	2	6	2	3	4	2	4	6	8	4	3	4	4	4	3	7
B839	2010	This study	BSP1	Sichuan	30.65	104.08	2	3	3	3	2	5	1	6	2	6	2	3	4	2	4	1	9	4	3	4	4	4	3	7
B840	2010	This study	BSP1	Sichuan	30.65	104.08	2	3	3	3	2	5	1	8	2	6	2	3	4	2	3	6	4	1	3	5	4	4	2	5
B841	2010	This study	BSP1	Sichuan	30.65	104.08	2	3	3	4	2	5	1	6	2	6	3	3	4	2	4	5	4	4	1	3	4	4	3	8
B842	2010	This study	BSP1	Sichuan	30.65	104.08	2	3	3	3	2	5	1	7	2	6	2	4	4	2	4	6	7	2	ND	5	4	4	3	6
B843	2010	This study	BSP1	Sichuan	30.65	104.08	2	3	3	3	2	5	1	7	2	6	3	4	4	2	4	5	ND	3	ND	4	4	4	3	4
B844	2011	This study	BSP1	Sichuan	30.65	104.08	2	3	2	2	1	5	1	7	3	ND	3	3	2	3	4	6	7	5	4	4	4	4	3	6
B845	2011	This study	BSP1	Sichuan	30.65	104.08	2	3	2	2	0	5	1	7	3	7	3	3	2	3	5	4	7	4	3	4	4	4	3	4
B846	2011	This study	BSP1	Sichuan	30.65	104.08	2	3	2	2	1	5	1	7	3	6	3	3	2	3	5	4	8	2	3	5	4	4	3	2
B847	2011	This study	BSP1	Sichuan	30.65	104.08	2	3	3	1	1	5	1	7	3	6	3	3	2	3		3	8	2	3	5	4	4	3	4
B848	2011	This study	BSP1	Sichuan	30.65	104.08	2	3	2	2	1	5	1	ND	3	6	1	3	2	3	5	4	7	4	3	4	4	4	3	4
B849	2011	This study	BSP1	Sichuan	30.65	104.08	2	3	1	1	1	5	1	5	3	4	2	3	1	2	4	3	9	2	1	3	4	4	3	4
B850	2011	This study	BSP1	Sichuan	30.65	104.08	2	3	2	2	1	5	1	7	3	6	3	3	2	3	5	5	3	2	3	5	4	5	3	4
B851	2011	This study	BSPint	Sichuan	30.65	104.08	2	3	2	2	1	5	1	7	3	5	3	3	2	3	2	6	8	4	3	4	4	4	3	4
B852	2011	This study	BSP1	Sichuan	30.65	104.08	2	3	2	2	1	5	1	6	3	6	3	2	2	3	5	2	8	3	2	4	4	4	3	7
B853	2011	This study	BSP1	Sichuan	30.65	104.08	2	3	2	2	1	5	1	7	1	6	3	1	2	3	5	5	9	3	2	4	4	4	3	4
B854	2011	This study	BSP1	Sichuan	30.65	104.08	2	3	2	2	1	5	1	7	2	6	3	4	2	3	5	4	3	2	3	ND	4	4	3	3
B855	2010	This study	BSP1	Sichuan	30.65	104.08	2	3	3	3	2	5	1	6	3	8	3	3	4	2	4	4	ND	2	3	9	4	4	3	4
B856	2010	This study	BSP1	Sichuan	30.65	104.08	2	3	3	3	2	5	1	6	1	5	2	3	4	2	4	3	ND	4	2	4	4	4	3	4
B857	2010	This study	BSP1	Sichuan	30.65	104.08	2	3	2	3	2	5	1	6	2	3	4	3	4	2	4	4	9	2	ND	5	4	4	3	4
B858	2010	This study	BSP1	Sichuan	30.65	104.08	2	3	3	3	2	5	1	8	2	3	3	3	4	2	4	3	ND	3	3	4	4	4	3	3
B859	2010	This study	BSP1	Sichuan	30.65	104.08	2	3	3	3	2	5	1	ND	3	5	3	3	3	2	4	5	ND	2	2	5	4	4	3	5
B860	2010	This study	BSPint	Sichuan	30.65	104.08	2	3	3	3	2	5	1	7	3	5	3	3	4	2	4	5	ND	2	2	5	4	4	3	5
B861	2010	This study	BSP1	Sichuan	30.65	104.08	2	3	3	3	2	5	1	7	2	4	3	3	4	2	4	5	ND	2	2	4	4	4	3	4
B862	2010	This study	BSP1	Sichuan	30.65	104.08	2	3	3	3	2	5	1	7	3	5	2	2	3	2	4	4	8	1	2	5	4	4	2	1
B863	2010	This study	BSP1	Sichuan	30.65	104.08	2	3	3	3	2	5	1	7	3	5	3	3	4	2	4	5	5	1	2	7	4	4	4	1
B864	2010	This study	BSPint	Sichuan	30.65	104.08	2	3	3	3	2	5	1	7	3	5	3	3	4	2	4	3	7	2	2	5	4	4	3	2
B865	2010	This study	BSP1	Sichuan	30.65	104.08	2	3	3	3	2	5	1	7	2	5	3	1	4	2	4	2	ND	2	2	4	4	4	3	2
B866	2010	This study	BSPint	Sichuan	30.65	104.08	2	3	1	3	2	5	1	5	3	5	3	3	4	2	4	5	9	4	3	4	4	4	3	4
B867	2010	This study	BSP5	Sichuan	30.65	104.08	2	3	3	3	2	5	1	6	3	5	3	3	4	2	4	5	9	2	3	5	2	4	3	2
B868	2010	This study	BSPint	Sichuan	30.65	104.08	2	3	3	3	2	5	1	7	3	5	3	4	4	2	4	5	8	2	3	5	4	5	3	1
B869	2010	This study	BSP1	Sichuan	30.65	104.08	2	3	3	3	2	5	1	7	3	5	3	3	2	2	4	3	7	2	2	5	4	4	3	1
B870	2010	This study	BSP1	Sichuan	30.65	104.08	2	3	3	3	2	5	1	5	3	5	4	2	4	2	4	4	6	ND	3	4	4	4	3	1
B871	2010	This study	BSP1	Sichuan	30.65	104.08	2	3	3	3	2	5	1	7	3	5	1	3	4	2	4	5	7	1	2	5	4	4	3	0
B872	2010	This study	BSPint	Sichuan	30.65	104.08	2	3	3	4	2	5	1	7	2	5	3	3	4	2	4	5	6	4	2	4	4	4	3	3
B873	2010	This study	BSP1	Sichuan	30.65	104.08	2	3	3	3	2	5	1	7	2	5	3	4	4	2	4	3	2	4	3	4	4	4	3	4
B874	2010	This study	BSP5	Sichuan	30.65	104.08	2	3	3	3	2	5	1	5	2	5	3	3	4	2	4	5	7	2	3	5	4	4	3	3
B875	2010	This study	BSP1	Sichuan	30.65	104.08	2	3	3	3	2	5	1	7	2	5	2	3	4	2	4	5	9	2	3	5	4	4	3	3
B876	2010	This study	BSP1	Sichuan	30.65	104.08	2	3	2	3	2	5	1	6	2	5	3	2	4	1	4	5	9	2	3	5	4	4	3	4

B877	2010	This study	BSP1	Sichuan	30.65	104.08	2	3	3	3	2	5	1	5	2	5	2	3	4	2	4	5	8	3	3	4	4	4	3	3
B878	2010	This study	BSP1	Sichuan	30.65	104.08	2	3	3	3	1	6	1	6	2	3	2	2	4	2	4	6	2	4	2	4	4	4	3	2
B879	2010	This study	BSP1	Sichuan	30.65	104.08	2	3	3	3	2	5	1	6	3	3	3	2	4	2	4	6	2	ND	4	4	4	4	3	4
B880	2010	This study	BSPint	Sichuan	30.65	104.08	2	1	3	3	1	5	1	7	3	5	2	3	4	2	4	4	7	2	3	5	4	4	3	2
B881	2010	This study	BSP1	Sichuan	30.65	104.08	2	3	3	3	0	5	1	7	2	5	1	3	4	2	4	5	7	2	3	5	3	4	3	2
B882	2010	This study	BSP1	Sichuan	30.65	104.08	2	3	3	3	1	5	1	7	2	5	2	3	4	2	3	5	5	2	3	7	4	2	3	2
B883	2010	This study	BSP1	Sichuan	30.65	104.08	2	3	2	3	1	5	ND	7	2	5	1	3	4	2	4	3	9	2	2	5	4	4	3	3
B884	2010	This study	BSP1	Sichuan	30.65	104.08	2	3	3	3	1	5	ND	7	2	5	3	3	4	2	4	5	0	ND	ND	3	4	4	3	2
B885	2010	This study	BSP1	Sichuan	30.65	104.08	2	3	3	3	1	5	1	7	2	5	2	3	3	2	4	4	8	3	ND	6	4	2	3	2
B886	2006-2010	Dong, H. *et al.* & 22479472	BSP5	Tibet	29.65	91.12	2	2	3	3	2	5	1	7	3	5	3	3	4	2	4	6	8	2	4	5	4	4	3	3
B887	2006-2010	Dong, H. *et al.* & 22479472	BSP4	Tibet	29.65	91.12	2	ND	3	3	1	5	1	7	3	5	3	3	4	2	4	5	7	4	4	4	4	4	3	3
B888	2006-2010	Dong, H. *et al.* & 22479472	BSP4	Tibet	29.65	91.12	2	2	3	3	2	5	1	7	3	5	3	3	4	2	4	6	7	4	4	4	4	4	3	3
B889	2006-2010	Dong, H. *et al.* & 22479472	BSP4	Tibet	29.65	91.12	2	2	3	3	2	5	1	7	3	5	3	3	4	2	4	6	7	4	4	4	4	4	3	3
B890	2006-2010	Dong, H. *et al.* & 22479472	BSP4	Tibet	29.65	91.12	2	2	3	3	1	5	1	7	3	6	3	4	3	2	4	4	7	4	4	4	4	4	3	3
B891	2006-2010	Dong, H. *et al.* & 22479472	BSP4	Tibet	29.65	91.12	2	2	3	3	2	5	1	7	3	5	3	3	4	2	4	6	7	4	4	4	4	4	3	3
B892	2006-2010	Dong, H. *et al.* & 22479472	BSP4	Tibet	29.65	91.12	2	2	3	3	1	5	1	7	3	6	3	4	3	2	4	4	7	4	4	4	4	4	3	3
B893	2006-2010	Dong, H. *et al.* & 22479472	BSP4	Tibet	29.65	91.12	2	2	3	3	2	5	1	7	3	2	3	3	4	2	4	6	7	4	4	4	4	4	2	3
B894	2006-2010	Dong, H. *et al.* & 22479472	BSP4	Tibet	29.65	91.12	2	2	3	3	2	5	1	7	3	2	3	3	4	2	4	6	7	4	4	4	4	4	3	3
B895	2006-2010	Dong, H. *et al.* & 22479472	BSP4	Tibet	29.65	91.12	2	2	3	3	2	5	1	7	3	2	3	3	4	2	4	6	7	4	4	4	4	4	3	3
B896	2006-2010	Dong, H. *et al.* & 22479472	BSP4	Tibet	29.65	91.12	2	2	3	3	1	5	1	7	3	6	3	3	4	2	4	4	7	4	4	4	4	4	3	3
B897	2006-2010	Dong, H. *et al.* & 22479472	BSP4	Tibet	29.65	91.12	2	2	3	3	2	5	1	7	3	2	3	3	2	2	4	7	7	4	4	4	4	4	3	3
B898	2006-2010	Dong, H. *et al.* & 22479472	BSP5	Tibet	29.65	91.12	2	4	3	3	2	5	1	6	3	5	3	1	4	2	4	5	8	2	4	4	4	4	3	3
B899	2006-2010	Dong, H. *et al.* & 22479472	BSP4	Tibet	29.65	91.12	2	2	3	3	2	5	1	4	3	2	3	2	4	2	4	7	7	4	4	4	4	4	3	3
B900	2006-2010	Dong, H. *et al.* & 22479472	BSP4	Tibet	29.65	91.12	2	2	3	3	2	5	1	7	3	5	3	3	4	2	4	4	7	4	4	4	4	4	3	3
B901	2006-2010	Dong, H. *et al.* & 22479472	BSP4	Tibet	29.65	91.12	2	2	3	3	1	5	1	7	3	6	3	3	4	2	4	4	7	4	4	4	4	4	3	3
B902	2006-2010	Dong, H. *et al.* & 22479472	BSP4	Tibet	29.65	91.12	2	2	3	3	1	5	1	7	3	5	3	3	4	2	4	5	7	4	4	4	4	4	3	3
B903	2006-2010	Dong, H. *et al.* & 22479472	BSP4	Tibet	29.65	91.12	2	2	3	3	2	5	1	7	3	5	3	3	4	2	4	6	7	4	4	4	4	4	3	3
B904	2006-2010	Dong, H. *et al.* & 22479472	BSP4	Tibet	29.65	91.12	2	2	3	3	2	5	1	7	3	5	3	3	4	2	4	6	7	4	4	4	4	4	3	3
B905	2006-2010	Dong, H. *et al.* & 22479472	BSP5	Tibet	29.65	91.12	2	2	3	3	2	5	1	8	3	4	3	3	4	2	4	6	7	2	4	5	4	4	3	3
B906	2006-2010	Dong, H. *et al.* & 22479472	BSP4	Tibet	29.65	91.12	2	2	3	3	2	5	1	7	3	5	3	3	4	2	2	6	7	4	4	4	4	4	3	3
B907	2006-2010	Dong, H. *et al.* & 22479472	BSP4	Tibet	29.65	91.12	2	2	3	3	2	5	1	7	3	5	3	3	4	2	4	6	7	4	4	4	4	4	3	3
B908	2006-2010	Dong, H. *et al.* & 22479472	BSP4	Tibet	29.65	91.12	2	2	3	3	1	5	1	7	3	6	3	3	4	2	4	5	7	4	5	4	4	4	3	3
B909	2006-2010	Dong, H. *et al.* & 22479472	BSP4	Tibet	29.65	91.12	2	2	3	3	2	5	1	7	3	5	3	3	4	2	4	6	7	4	4	4	4	4	3	3
B910	2006-2010	Dong, H. *et al.* & 22479472	BSP4	Tibet	29.65	91.12	2	2	3	3	1	5	1	7	3	6	3	4	4	2	4	4	7	3	4	4	4	4	3	3
B911	2006-2010	Dong, H. *et al.* & 22479472	BSP4	Tibet	29.65	91.12	2	2	3	3	2	5	1	7	5	2	3	3	4	2	4	7	7	4	4	4	4	4	3	3
B912	2006-2010	Dong, H. *et al.* & 22479472	BSP5	Tibet	29.65	91.12	2	2	3	3	2	5	1	6	3	5	3	3	4	2	4	6	9	2	4	6	4	4	3	3
B913	2006-2010	Dong, H. *et al.* & 22479472	BSP4	Tibet	29.65	91.12	2	2	3	3	1	5	1	7	3	4	3	3	4	2	4	4	7	4	4	4	4	4	3	2
B914	2006-2010	Dong, H. *et al.* & 22479472	BSP5	Tibet	29.65	91.12	2	2	3	3	2	5	1	8	3	4	3	3	4	2	4	6	7	2	4	5	4	4	3	3
B915	2006-2010	Dong, H. *et al.* & 22479472	BSP4	Tibet	29.65	91.12	2	2	3	3	1	5	1	7	3	6	4	3	4	2	4	4	7	4	4	4	4	4	3	3
B916	2006-2010	Dong, H. *et al.* & 22479472	BSP4	Tibet	29.65	91.12	2	2	3	3	1	5	1	8	3	2	3	3	4	2	4	6	7	4	4	4	4	4	3	3
B917	2006-2010	Dong, H. *et al.* & 22479472	BSP5	Tibet	29.65	91.12	2	2	3	3	2	5	1	7	3	5	3	3	4	2	4	5	7	2	4	5	4	4	3	3
B918	2006-2010	Dong, H. *et al.* & 22479472	BSP4	Tibet	29.65	91.12	2	2	3	3	2	5	1	8	3	2	3	3	4	2	4	7	7	4	4	4	4	4	3	3
B919	2006-2010	Dong, H. *et al.* &	BSP4	Tibet	29.65	91.12	2	2	3	3	1	5	1	7	3	5	2	3	2	2	4	5	7	4	4	4	4	4	3	3

		22479472																												
B920	2006-2010	Dong, H. *et al.* & 22479472	BSP4	Tibet	29.65	91.12	2	2	3	3	1	5	1	7	3	5	3	1	4	2	4	6	7	2	4	4	4	4	3	3
B921	2006-2010	Dong, H. *et al.* & 22479472	BSP5	Tibet	29.65	91.12	2	2	3	3	2	5	1	7	3	5	3	3	4	2	4	3	8	2	4	5	4	4	3	3
B922	2006-2010	Dong, H. *et al.* & 22479472	BSP4	Tibet	29.65	91.12	2	2	3	3	2	5	1	7	3	2	3	3	4	2	4	7	7	4	4	4	4	4	3	3
B923	2006-2010	Dong, H. *et al.* & 22479472	BSP4	Tibet	29.65	91.12	2	2	3	3	1	5	1	7	3	6	3	4	4	2	4	4	7	4	4	4	4	4	3	3
B924	2006-2010	Dong, H. *et al.* & 22479472	BSP4	Tibet	29.65	91.12	2	2	3	3	1	5	1	7	3	5	3	3	4	2	4	5	7	4	4	4	4	4	3	3
B925	2006-2010	Dong, H. *et al.* & 22479472	BSP4	Tibet	29.65	91.12	2	2	3	3	1	5	1	7	3	2	3	3	4	2	4	6	7	4	4	4	4	4	3	3
B926	2006-2010	Dong, H. *et al.* & 22479472	BSP4	Tibet	29.65	91.12	2	2	3	3	2	5	1	7	3	5	3	3	4	2	4	7	8	4	4	4	4	4	3	4
B927	2006-2010	Dong, H. *et al.* & 22479472	BSP5	Tibet	29.65	91.12	2	2	3	3	2	5	1	7	3	5	3	3	4	2	4	3	3	2	4	4	4	4	3	4
B928	2006-2010	Dong, H. *et al.* & 22479472	BSP4	Tibet	29.65	91.12	2	2	3	2	1	5	1	7	3	5	3	3	2	2	4	5	7	4	4	4	4	4	3	3
B929	2006-2010	Dong, H. *et al.* & 22479472	BSP4	Tibet	29.65	91.12	2	2	3	3	2	5	1	7	3	5	3	3	4	2	4	6	7	4	5	4	4	4	3	3
B930	2006-2010	Dong, H. *et al.* & 22479472	BSP4	Tibet	29.65	91.12	2	2	3	3	2	5	1	7	3	2	3	3	4	2	4	7	7	4	4	4	4	4	3	3
B931	2006-2010	Dong, H. *et al.* & 22479472	BSP4	Tibet	29.65	91.12	2	2	3	3	2	5	1	9	3	5	3	3	4	2	4	8	7	4	4	4	4	4	3	3
B932	2006-2010	Dong, H. *et al.* & 22479472	BSP5	Tibet	29.65	91.12	2	2	3	3	2	5	1	7	3	5	3	3	4	2	4	5	7	2	4	5	4	4	3	3
B933	2006-2010	Dong, H. *et al.* & 22479472	BSP4	Tibet	29.65	91.12	2	2	3	2	2	5	1	7	3	5	3	1	4	2	4	6	2	4	4	4	4	4	3	3
B934	2006-2010	Dong, H. *et al.* & 22479472	BSP4	Tibet	29.65	91.12	2	2	3	3	2	5	1	7	3	2	3	3	4	2	4	6	7	4	4	4	4	4	3	3
B935	2006-2010	Dong, H. *et al.* & 22479472	BSP5	Tibet	29.65	91.12	2	2	3	3	2	5	1	7	3	4	3	3	4	2	4	6	8	2	4	5	4	4	3	3
B936	2006-2010	Dong, H. *et al.* & 22479472	BSP4	Tibet	29.65	91.12	2	2	3	3	1	5	1	7	3	6	3	3	4	2	4	4	7	4	4	3	4	4	3	3
B937	2006-2010	Dong, H. *et al.* & 22479472	BSP4	Tibet	29.65	91.12	2	2	3	3	2	5	1	7	3	2	3	3	4	2	4	6	7	4	4	3	4	4	3	3
B938	2006-2010	Dong, H. *et al.* & 22479472	BSP1	Tibet	29.65	91.12	2	6	2	3	2	5	1	5	2	3	2	2	2	2	4	3	3	2	4	1	4	2	5	1
B939	2006-2010	Dong, H. *et al.* & 22479472	BSP4	Tibet	29.65	91.12	2	2	3	3	2	5	1	8	3	5	3	3	4	2	4	6	7	4	4	4	4	4	3	3
B940	2006-2010	Dong, H. *et al.* & 22479472	BSP5	Tibet	29.65	91.12	2	2	3	3	2	5	1	5	3	5	3	3	4	2	4	6	8	2	4	5	4	4	3	3
B941	2006-2010	Dong, H. *et al.* & 22479472	BSP5	Tibet	29.65	91.12	2	2	3	2	2	5	1	8	3	4	3	3	4	2	4	6	7	2	4	5	4	4	3	3
B942	2006-2010	Dong, H. *et al.* & 22479472	BSP5	Tibet	29.65	91.12	2	2	3	3	2	5	1	7	3	4	3	3	4	2	4	6	8	2	3	5	4	4	3	3
B943	2006-2010	Dong, H. *et al.* & 22479472	BSP4	Tibet	29.65	91.12	2	2	3	2	1	5	1	7	3	5	3	3	4	2	4	5	7	4	4	4	4	4	3	3
B944	2006-2010	Dong, H. *et al.* & 22479472	BSP1	Tibet	29.65	91.12	2	2	3	3	2	6	1	6	3	4	3	2	4	2	4	5	8	2	1	4	4	4	3	4
B945	2006-2010	Dong, H. *et al.* & 22479472	BSP4	Tibet	29.65	91.12	2	2	3	3	1	5	1	7	3	6	3	3	4	2	4	3	8	4	4	4	4	4	2	3
B946	2006-2010	Dong, H. *et al.* & 22479472	BSP4	Tibet	29.65	91.12	2	2	3	3	1	5	1	7	3	5	3	3	4	2	4	5	7	4	4	4	4	4	3	3
B947	2006-2010	Dong, H. *et al.* & 22479472	BSP4	Tibet	29.65	91.12	2	2	3	3	2	5	1	7	3	5	3	3	4	2	4	6	7	4	4	4	4	4	3	3
B948	2006-2010	Dong, H. *et al.* & 22479472	BSP5	Tibet	29.65	91.12	2	2	3	3	2	5	1	7	3	6	3	3	4	2	4	6	8	2	4	4	4	2	3	3
B949	2006-2010	Dong, H. *et al.* & 22479472	BSP4	Tibet	29.65	91.12	2	2	3	3	1	5	1	7	3	6	4	3	4	2	4	4	7	4	4	4	4	4	3	3
B950	2006-2010	Dong, H. *et al.* & 22479472	BSP4	Tibet	29.65	91.12	2	2	3	3	2	5	1	7	3	5	3	3	4	2	4	6	7	4	4	4	4	4	3	3
B951	2006-2010	Dong, H. *et al.* & 22479472	BSP4	Tibet	29.65	91.12	2	2	3	3	1	5	1	7	3	5	3	3	2	2	4	4	8	4	4	4	4	4	3	3
B952	2006-2010	Dong, H. *et al.* & 22479472	BSP4	Tibet	29.65	91.12	2	2	3	3	1	5	1	7	3	5	3	3	4	2	4	5	7	4	4	4	4	4	3	3
B953	2006-2010	Dong, H. *et al.* & 22479472	BSP4	Tibet	29.65	91.12	2	2	3	3	2	5	1	6	3	5	3	3	4	2	4	3	6	3	3	4	4	4	3	3
B954	2006-2010	Dong, H. *et al.* & 22479472	BSP4	Tibet	29.65	91.12	2	2	3	3	1	5	1	7	3	6	3	3	4	2	4	4	6	4	4	4	4	4	3	3
B955	2006-2010	Dong, H. *et al.* & 22479472	BSP5	Tibet	29.65	91.12	2	1	3	2	2	5	1	7	3	5	3	3	4	2	4	3	8	2	3	5	4	4	3	3
B956	2006-2010	Dong, H. *et al.* & 22479472	BSP4	Tibet	29.65	91.12	2	2	3	3	1	5	1	7	3	6	3	4	4	2	4	4	7	4	4	4	4	4	3	3
B957	2006-2010	Dong, H. *et al.* & 22479472	BSP5	Tibet	29.65	91.12	2	2	3	3	2	5	1	7	3	5	3	3	4	2	4	5	7	2	4	5	4	4	3	3
B958	2006-2010	Dong, H. *et al.* & 22479472	BSP5	Tibet	29.65	91.12	2	2	3	3	2	5	1	8	3	4	3	3	4	2	4	6	7	2	4	5	4	4	3	3
B959	2006-2010	Dong, H. *et al.* & 22479472	BSP4	Tibet	29.65	91.12	2	2	3	3	1	5	1	7	3	6	3	3	4	2	4	4	7	4	4	4	4	4	3	3
B960	2006-2010	Dong, H. *et al.* &	BSP4	Tibet	29.65	91.12	2	2	1	3	2	5	1	7	3	5	3	3	4	2	4	5	8	4	3	4	4	4	3	3

		22479472																												
B961	2006-2010	Dong, H. *et al.* & 22479472	BSP4	Tibet	29.65	91.12	2	2	3	3	1	5	1	7	3	6	3	3	4	2	4	4	7	4	4	4	4	4	3	3
B962	2006-2010	Dong, H. *et al.* & 22479472	BSP5	Tibet	29.65	91.12	2	2	3	3	2	5	1	8	3	4	3	3	4	2	4	5	9	2	4	5	4	4	3	2
B963	2006-2010	Dong, H. *et al.* & 22479472	BSP5	Tibet	29.65	91.12	2	2	3	3	2	5	1	8	3	4	3	3	4	2	4	6	7	2	4	5	4	4	3	3
B964	2006-2010	Dong, H. *et al.* & 22479472	BSP4	Tibet	29.65	91.12	2	2	3	3	1	5	1	8	3	5	3	3	4	2	4	5	7	4	4	4	4	4	3	3
B965	2006-2010	Dong, H. *et al.* & 22479472	BSP5	Tibet	29.65	91.12	2	2	3	3	2	5	1	5	3	5	3	3	4	2	4	6	8	2	4	5	4	4	3	3
B966	2006-2010	Dong, H. *et al.* & 22479472	BSP5	Tibet	29.65	91.12	2	2	3	3	2	5	1	5	3	5	3	3	4	2	4	6	8	2	4	5	4	4	3	3
B967	2006-2010	Dong, H. *et al.* & 22479472	BSP5	Tibet	29.65	91.12	2	2	3	3	2	5	1	8	3	4	3	3	4	2	4	6	7	4	4	5	4	4	3	2
B968	2006-2010	Dong, H. *et al.* & 22479472	BSP5	Tibet	29.65	91.12	2	2	3	3	2	5	1	8	3	4	3	3	4	2	4	6	7	2	4	5	4	4	3	3
B969	2006-2010	Dong, H. *et al.* & 22479472	BSP4	Tibet	29.65	91.12	2	2	3	3	1	5	1	7	3	6	3	4	4	2	4	4	7	3	4	4	4	4	3	3
B970	2006-2010	Dong, H. *et al.* & 22479472	BSP4	Tibet	29.65	91.12	2	2	3	3	2	5	1	7	3	2	3	3	4	2	4	8	7	4	4	4	4	4	3	3
B971	2006-2010	Dong, H. *et al.* & 22479472	BSP5	Tibet	29.65	91.12	2	2	3	3	2	5	1	5	3	5	3	3	4	2	4	7	8	2	4	5	4	4	3	3
B972	2006-2010	Dong, H. *et al.* & 22479472	BSP5	Tibet	29.65	91.12	2	2	3	3	2	5	1	7	3	5	2	3	4	2	4	7	8	2	4	6	4	4	3	3
B973	2006-2010	Dong, H. *et al.* & 22479472	BSP5	Tibet	29.65	91.12	2	2	3	3	2	5	1	7	3	5	2	3	4	2	4	7	8	2	4	6	4	4	3	3
B974	2006-2010	Dong, H. *et al.* & 22479472	BSP4	Tibet	29.65	91.12	2	2	3	3	1	5	1	8	3	5	3	3	4	2	4	5	7	4	4	4	4	4	3	3
B975	2006-2010	Dong, H. *et al.* & 22479472	BSP5	Tibet	29.65	91.12	2	2	3	3	2	5	1	7	3	5	3	3	4	2	4	5	7	2	4	5	4	4	3	3
B976	2006-2010	Dong, H. *et al.* & 22479472	BSP4	Tibet	29.65	91.12	2	2	3	3	2	5	1	7	3	5	3	3	4	2	4	6	7	4	4	4	4	4	3	3
B977	2006-2010	Dong, H. *et al.* & 22479472	BSP4	Tibet	29.65	91.12	2	2	3	3	2	5	1	7	3	2	3	3	4	2	4	5	7	4	4	4	4	4	3	3
B978	2006-2010	Dong, H. *et al.* & 22479472	BSP5	Tibet	29.65	91.12	2	2	3	3	2	5	1	8	3	4	3	3	4	2	4	6	7	2	4	5	4	4	3	3
B979	2006-2010	Dong, H. *et al.* & 22479472	BSP4	Tibet	29.65	91.12	2	2	3	3	2	5	1	8	3	2	3	3	4	2	4	6	7	4	4	4	4	4	3	3
B980	2006-2010	Dong, H. *et al.* & 22479472	BSP4	Tibet	29.65	91.12	2	2	3	3	1	5	1	7	3	6	3	3	4	2	4	4	7	4	4	3	4	4	3	3
B981	2006-2010	Dong, H. *et al.* & 22479472	BSP4	Tibet	29.65	91.12	2	2	3	3	1	5	1	7	3	5	3	3	4	2	4	5	7	2	4	4	4	4	3	3
B982	2006-2010	Dong, H. *et al.* & 22479472	BSP5	Tibet	29.65	91.12	2	2	3	3	2	5	1	7	3	4	3	3	4	2	4	5	7	2	4	5	4	4	3	3
B983	2006-2010	Dong, H. *et al.* & 22479472	BSP4	Tibet	29.65	91.12	2	2	3	3	1	5	1	7	3	5	3	3	4	2	4	5	7	4	4	4	4	4	3	3
B984	2006-2010	Dong, H. *et al.* & 22479472	BSP5	Tibet	29.65	91.12	2	2	3	3	2	5	1	7	3	5	2	3	4	2	4	6	8	2	1	6	4	4	3	3
B985	2006-2010	Dong, H. *et al.* & 22479472	BSP5	Tibet	29.65	91.12	2	2	3	3	2	5	1	8	3	6	3	3	4	2	3	6	8	2	4	5	4	4	3	2
B986	2006-2010	Dong, H. *et al.* & 22479472	BSP4	Tibet	29.65	91.12	2	2	3	3	1	5	1	5	3	6	3	3	4	2	4	5	7	4	3	5	4	4	3	2
B987	2006-2010	Dong, H. *et al.* & 22479472	BSP4	Tibet	29.65	91.12	2	2	3	3	1	5	1	7	3	5	3	3	4	3	4	5	7	4	4	4	4	4	3	3
B988	2006-2010	Dong, H. *et al.* & 22479472	BSP5	Tibet	29.65	91.12	2	2	3	3	2	5	1	8	3	4	3	3	4	2	4	6	7	2	4	5	4	4	3	3
B989	2006-2010	Dong, H. *et al.* & 22479472	BSP5	Tibet	29.65	91.12	2	2	3	3	2	5	1	7	3	5	2	3	4	2	4	6	8	2	4	6	4	4	3	3
B990	2006-2010	Dong, H. *et al.* & 22479472	BSP4	Tibet	29.65	91.12	2	2	3	3	1	5	1	7	3	6	3	3	4	2	4	4	6	4	4	4	4	4	3	3
B991	2006-2010	Dong, H. *et al.* & 22479472	BSP4	Tibet	29.65	91.12	2	2	3	3	1	5	1	7	3	6	3	3	4	2	4	4	7	4	4	3	4	4	3	3
B992	2006-2010	Dong, H. *et al.* & 22479472	BSP4	Tibet	29.65	91.12	2	2	3	3	1	5	1	7	3	6	3	3	4	2	4	4	7	4	4	3	4	4	3	3
B993	2006-2010	Dong, H. *et al.* & 22479472	BSP4	Tibet	29.65	91.12	2	2	3	3	2	5	1	7	3	2	3	3	4	2	4	7	7	4	4	4	4	4	3	3
B994	2006-2010	Dong, H. *et al.* & 22479472	BSP5	Tibet	29.65	91.12	2	2	3	3	2	5	1	6	3	5	3	3	4	2	4	6	8	2	4	5	4	4	3	3
B995	2006-2010	Dong, H. *et al.* & 22479472	BSP5	Tibet	29.65	91.12	2	2	3	3	2	5	1	8	3	6	3	3	4	2	3	6	8	2	4	5	4	4	3	2
B996	2006-2010	Dong, H. *et al.* & 22479472	BSP4	Tibet	29.65	91.12	2	2	3	3	2	5	1	7	3	2	3	3	4	2	4	6	7	2	4	4	4	4	3	3
B997	2006-2010	Dong, H. *et al.* & 22479472	BSP4	Tibet	29.65	91.12	2	2	3	3	2	5	1	7	3	5	3	3	4	2	4	6	7	4	4	4	4	4	3	3
B998	2006-2010	Dong, H. *et al.* & 22479472	BSP4	Tibet	29.65	91.12	2	2	3	3	1	5	1	7	3	5	3	3	4	2	4	4	7	4	4	4	4	2	3	3
B999	2006-2010	Dong, H. *et al.* & 22479472	BSP4	Tibet	29.65	91.12	2	2	3	3	1	5	1	7	3	6	3	4	4	2	4	4	7	4	4	4	4	4	3	2
B1000	2006-2010	Dong, H. *et al.* & 22479472	BSP5	Tibet	29.65	91.12	2	2	3	3	2	5	1	7	3	5	3	3	4	2	4	4	8	2	4	5	4	4	3	3
B1001	2006-2010	Dong, H. *et al.* &	BSP4	Tibet	29.65	91.12	2	2	3	3	1	5	1	7	3	6	3	3	4	2	4	4	7	4	4	3	4	4	3	3

		22479472																												
B1002	2006-2010	Dong, H. *et al.* & 22479472	BSP5	Tibet	29.65	91.12	2	2	3	3	2	5	1	7	3	5	3	3	4	2	4	5	8	2	4	5	4	4	3	3
B1003	2006-2010	Dong, H. *et al.* & 22479472	BSP5	Tibet	29.65	91.12	2	2	3	3	2	5	1	7	3	5	3	2	4	2	4	2	8	2	4	5	4	4	4	3
B1004	2006-2010	Dong, H. *et al.* & 22479472	BSP4	Tibet	29.65	91.12	2	2	3	3	1	5	1	8	3	5	3	3	4	2	4	5	7	4	4	4	4	4	3	3
B1005	2006-2010	Dong, H. *et al.* & 22479472	BSP5	Tibet	29.65	91.12	2	2	3	3	2	5	1	7	3	5	3	3	4	2	4	6	8	2	5	5	4	4	3	3
B1006	2006-2010	Dong, H. *et al.* & 22479472	BSP4	Tibet	29.65	91.12	2	2	3	3	1	5	1	7	3	6	3	3	4	2	4	4	7	4	4	4	4	4	3	3
B1007	2006-2010	Dong, H. *et al.* & 22479472	BSP5	Tibet	29.65	91.12	2	2	3	2	2	5	1	7	3	5	3	3	4	2	4	4	8	2	5	5	4	4	3	3
B1008	2006-2010	Dong, H. *et al.* & 22479472	BSP4	Tibet	29.65	91.12	2	2	3	3	1	5	1	7	3	5	3	3	2	2	4	4	8	4	4	4	4	4	3	3
B1009	2006-2010	Dong, H. *et al.* & 22479472	BSP4	Tibet	29.65	91.12	2	2	3	2	2	6	1	7	3	5	3	3	4	2	4	4	7	3	3	4	4	4	3	3
B1010	2006-2010	Dong, H. *et al.* & 22479472	BSP4	Tibet	29.65	91.12	2	2	3	3	1	5	1	7	3	6	3	3	4	2	4	5	7	4	4	4	4	4	3	3
B1011	2006-2010	Dong, H. *et al.* & 22479472	BSP5	Tibet	29.65	91.12	2	2	3	3	2	5	1	8	3	4	3	3	4	2	4	5	9	2	4	5	4	4	3	2
B1012	2006-2010	Dong, H. *et al.* & 22479472	BSP5	Tibet	29.65	91.12	2	2	3	3	2	5	1	5	3	5	3	3	4	2	4	6	8	2	4	5	4	4	3	3
B1013	2006-2010	Dong, H. *et al.* & 22479472	BSP4	Tibet	29.65	91.12	2	2	3	3	1	5	1	7	3	5	3	4	4	2	4	4	7	4	4	4	4	4	3	2
B1014	2006-2010	Dong, H. *et al.* & 22479472	BSP5	Tibet	29.65	91.12	2	2	3	3	2	5	1	8	3	4	3	3	4	2	4	6	7	2	4	5	4	4	3	3
B1015	2006-2010	Dong, H. *et al.* & 22479472	BSP4	Tibet	29.65	91.12	2	2	3	3	2	5	1	7	3	5	3	2	4	2	4	6	7	2	4	4	4	4	3	3
B1016	2006-2010	Dong, H. *et al.* & 22479472	BSP4	Tibet	29.65	91.12	2	2	3	3	2	5	1	8	3	5	3	3	4	2	4	6	5	4	4	4	4	4	3	3
B1017	2006-2010	Dong, H. *et al.* & 22479472	BSP4	Tibet	29.65	91.12	2	2	3	3	1	5	1	7	3	5	3	3	4	2	4	5	7	2	4	4	4	4	3	3
B1018	2006-2010	Dong, H. *et al.* & 22479472	BSP4	Tibet	29.65	91.12	2	2	3	3	2	5	1	7	3	5	3	3	4	2	4	6	7	4	4	4	4	4	3	3
B1019	2006-2010	Dong, H. *et al.* & 22479472	BSP4	Tibet	29.65	91.12	2	2	3	3	2	5	1	7	3	5	3	3	4	2	4	6	7	4	4	4	4	4	3	3
B1020	2006-2010	Dong, H. *et al.* & 22479472	BSPint	Tibet	29.65	91.12	2	2	3	3	2	5	1	6	3	5	3	2	4	2	4	5	9	3	3	4	4	4	3	4
B1021	2006-2010	Dong, H. *et al.* & 22479472	BSP5	Tibet	29.65	91.12	2	2	3	3	2	5	1	8	3	4	3	3	4	2	4	6	7	2	4	5	4	4	3	3
B1022	2006-2010	Dong, H. *et al.* & 22479472	BSP5	Tibet	29.65	91.12	2	2	3	3	2	5	1	7	3	5	3	3	4	2	4	6	8	2	4	5	4	4	3	3
B1023	2006-2010	Dong, H. *et al.* & 22479472	BSP4	Tibet	29.65	91.12	2	2	3	3	1	5	1	6	3	5	3	3	4	2	4	5	7	4	4	4	4	4	3	3
B1024	2006-2010	Dong, H. *et al.* & 22479472	BSP4	Tibet	29.65	91.12	2	2	3	3	1	5	1	7	3	6	3	4	4	2	4	4	4	2	5	4	4	4	3	3
B1025	2006-2010	Dong, H. *et al.* & 22479472	BSP4	Tibet	29.65	91.12	2	2	3	3	1	5	1	7	3	6	3	3	4	2	4	4	7	4	4	3	4	4	3	3
B1026	2006-2010	Dong, H. *et al.* & 22479472	BSP5	Tibet	29.65	91.12	2	2	3	3	2	5	1	7	3	5	3	3	4	2	4	5	9	2	6	5	4	4	3	3
B1027	2006-2010	Dong, H. *et al.* & 22479472	BSP5	Tibet	29.65	91.12	2	2	3	3	2	5	1	8	3	4	3	3	4	2	4	6	7	2	4	5	4	4	3	3
B1028	2006-2010	Dong, H. *et al.* & 22479472	BSP1	Tibet	29.65	91.12	2	3	2	3	2	5	1	5	2	3	1	2	4	2	4	2	7	2	4	1	4	2	3	1
B1029	2006-2010	Dong, H. *et al.* & 22479472	BSP4	Tibet	29.65	91.12	2	2	3	2	1	5	1	7	3	5	3	3	4	2	4	5	7	4	4	4	4	4	3	3
B1030	2006-2010	Dong, H. *et al.* & 22479472	BSP5	Tibet	29.65	91.12	2	2	3	3	2	5	1	8	3	4	3	3	4	2	4	6	7	2	4	5	4	2	3	3
B1031	2006-2010	Dong, H. *et al.* & 22479472	BSP4	Tibet	29.65	91.12	2	2	3	3	2	5	1	8	3	5	4	3	4	2	4	6	9	4	3	4	4	4	3	3
B1032	2006-2010	Dong, H. *et al.* & 22479472	BSP4	Tibet	29.65	91.12	2	2	3	3	1	5	1	7	3	5	3	3	4	2	4	5	8	4	4	4	4	4	3	3
B1033	2006-2010	Dong, H. *et al.* & 22479472	BSP4	Tibet	29.65	91.12	2	2	3	3	2	5	1	8	3	5	4	3	4	2	4	6	9	4	3	4	4	4	3	3
B1034	2006-2010	Dong, H. *et al.* & 22479472	BSP4	Tibet	29.65	91.12	2	2	3	3	1	5	1	7	3	5	3	3	4	2	4	4	6	4	4	4	4	4	3	3
B1035	2006-2010	Dong, H. *et al.* & 22479472	BSP5	Tibet	29.65	91.12	2	2	3	3	2	5	1	7	3	4	3	3	4	2	4	6	8	2	4	5	4	4	3	3
B1036	2006-2010	Dong, H. *et al.* & 22479472	BSP4	Tibet	29.65	91.12	2	2	3	3	2	5	1	7	3	2	3	3	4	2	4	7	6	4	4	4	4	4	3	3
B1037	2006-2010	Dong, H. *et al.* & 22479472	BSP4	Tibet	29.65	91.12	2	2	3	3	2	5	1	7	3	4	3	3	4	2	4	6	7	4	4	4	4	4	3	3
B1038	2006-2010	Dong, H. *et al.* & 22479472	BSP4	Tibet	29.65	91.12	2	2	3	3	1	5	1	7	3	4	3	3	4	2	4	5	6	4	4	4	4	4	3	3
B1039	2006-2010	Dong, H. *et al.* & 22479472	BSP4	Tibet	29.65	91.12	2	2	3	3	2	5	1	7	3	2	3	3	2	2	4	7	7	4	4	4	4	4	3	3
B1040	2006-2010	Dong, H. *et al.* & 22479472	BSP4	Tibet	29.65	91.12	2	2	3	3	2	5	1	7	3	2	3	3	4	2	4	6	7	2	4	4	4	4	3	3
B1041	2006-2010	Dong, H. *et al.* & 22479472	BSP4	Tibet	29.65	91.12	2	2	3	3	2	5	1	7	3	4	3	3	4	2	4	6	7	4	4	4	4	4	3	3
B1042	2006-2010	Dong, H. *et al.* &	BSP5	Tibet	29.65	91.12	2	2	3	3	2	5	1	7	3	4	3	3	4	2	4	4	8	2	4	5	4	4	3	3

		22479472																												
B1043	2006-2010	Dong, H. *et al.* & 22479472	BSP4	Tibet	29.65	91.12	2	2	3	3	2	5	1	7	3	2	3	3	4	2	4	6	7	2	4	4	4	4	3	3
B1044	2006-2010	Dong, H. *et al.* & 22479472	BSP4	Tibet	29.65	91.12	2	2	3	3	2	5	1	7	3	4	3	3	4	2	4	6	7	4	4	4	4	4	3	3
B1045	2006-2010	Dong, H. *et al.* & 22479472	BSP5	Tibet	29.65	91.12	2	2	3	3	2	5	1	7	3	5	3	3	4	2	3	6	8	2	4	5	4	4	3	2
B1046	2006-2010	Dong, H. *et al.* & 22479472	BSPint	Tibet	29.65	91.12	2	2	3	3	2	6	1	5	3	4	3	2	4	2	4	5	8	2	1	4	4	4	3	4
B1047	2006-2010	Dong, H. *et al.* & 22479472	BSP5	Tibet	29.65	91.12	2	2	3	2	2	6	1	6	4	4	3	3	4	2	4	6	7	2	4	5	4	4	3	3
B1048	2006-2010	Dong, H. *et al.* & 22479472	BSP4	Tibet	29.65	91.12	2	2	3	2	1	5	1	6	4	4	3	3	4	2	4	5	7	4	4	4	4	4	3	3
B1049	2006-2010	Dong, H. *et al.* & 22479472	BSPint	Tibet	29.65	91.12	2	2	2	3	2	5	1	4	3	5	3	3	4	2	4	9	8	3	4	4	4	4	3	5
B1050	2006-2010	Dong, H. *et al.* & 22479472	BSP4	Tibet	29.65	91.12	2	2	3	3	2	5	1	6	3	4	3	3	4	2	4	6	7	4	4	4	4	4	3	3
B1051	2006-2010	Dong, H. *et al.* & 22479472	BSPint	Tibet	29.65	91.12	2	2	3	3	2	5	1	7	3	5	3	3	4	2	4	6	6	2	3	3	4	4	3	3
B1052	2006-2010	Dong, H. *et al.* & 22479472	BSP4	Tibet	29.65	91.12	2	2	3	3	2	5	1	9	3	5	3	3	4	2	4	6	7	4	5	4	4	4	3	3
B1053	2006-2010	Dong, H. *et al.* & 22479472	BSP5	Tibet	29.65	91.12	2	2	3	3	2	5	1	7	3	5	3	3	4	2	4	9	8	2	4	5	4	4	3	3
B1054	2006-2010	Dong, H. *et al.* & 22479472	BSP4	Tibet	29.65	91.12	2	2	3	3	1	5	1	7	3	5	3	3	4	2	4	4	7	4	4	4	4	4	3	2
B1055	2006-2010	Dong, H. *et al.* & 22479472	BSP4	Tibet	29.65	91.12	2	2	3	2	2	5	1	7	3	5	3	1	4	2	4	6	2	4	4	4	4	4	3	3
B1056	2006-2010	Dong, H. *et al.* & 22479472	BSP4	Tibet	29.65	91.12	2	2	3	3	2	5	1	8	3	5	3	3	4	2	4	6	7	4	4	4	4	4	3	3
B1057	2006-2010	Dong, H. *et al.* & 22479472	BSP4	Tibet	29.65	91.12	2	2	3	3	2	5	1	7	3	5	3	3	4	2	4	6	7	4	3	4	4	4	3	3
B1058	2006-2010	Dong, H. *et al.* & 22479472	BSPint	Tibet	29.65	91.12	2	2	2	3	1	5	1	6	3	5	3	3	4	2	4	6	7	2	3	5	4	4	3	3
B1059	2006-2010	Dong, H. *et al.* & 22479472	BSP4	Tibet	29.65	91.12	2	2	3	3	2	5	1	7	3	2	3	3	4	2	4	7	6	4	4	4	4	4	3	3
B1060	2006-2010	Dong, H. *et al.* & 22479472	BSPint	Tibet	29.65	91.12	2	2	3	4	2	5	1	6	3	4	2	3	4	2	4	6	7	3	3	4	4	4	3	3
B1061	2006-2010	Dong, H. *et al.* & 22479472	BSP4	Tibet	29.65	91.12	2	2	3	3	1	5	1	7	3	6	3	3	3	2	4	4	7	4	4	5	4	4	3	3
B1062	2006-2010	Dong, H. *et al.* & 22479472	BSP4	Tibet	29.65	91.12	2	2	3	3	1	5	1	7	3	6	3	3	4	2	4	3	8	4	4	4	4	4	2	3
B1063	2006-2010	Dong, H. *et al.* & 22479472	BSP5	Tibet	29.65	91.12	2	2	3	3	2	5	1	7	3	4	3	3	4	2	4	6	8	2	4	5	4	4	3	3
B1064	2006-2010	Dong, H. *et al.* & 22479472	BSP4	Tibet	29.65	91.12	2	2	3	3	1	5	1	7	3	6	3	3	4	2	4	4	7	2	4	4	4	4	3	3
B1065	2006-2010	Dong, H. *et al.* & 22479472	BSP5	Tibet	29.65	91.12	2	2	3	3	2	5	1	7	3	6	3	2	4	2	4	6	7	2	4	5	4	4	3	3
B1066	2006-2010	Dong, H. *et al.* & 22479472	BSP4	Tibet	29.65	91.12	2	2	3	3	1	5	1	7	3	4	3	3	4	2	4	5	7	4	4	4	4	4	3	3
B1067	2006-2010	Dong, H. *et al.* & 22479472	BSP4	Tibet	29.65	91.12	2	2	3	3	1	5	1	7	3	6	3	3	4	2	4	4	7	4	4	4	4	4	3	3
B1068	2006-2010	Dong, H. *et al.* & 22479472	BSP4	Tibet	29.65	91.12	2	2	3	3	2	5	1	7	3	5	3	3	4	2	4	6	7	4	4	4	4	4	3	3
B1069	2006-2010	Dong, H. *et al.* & 22479472	BSP4	Tibet	29.65	91.12	2	2	3	3	1	5	1	7	3	5	3	2	4	2	4	5	7	4	4	4	4	4	3	3
B1070	2006-2010	Dong, H. *et al.* & 22479472	BSP5	Tibet	29.65	91.12	2	2	3	3	2	5	1	7	3	5	3	3	4	2	4	5	7	2	4	5	4	4	3	3
B1071	2006-2010	Dong, H. *et al.* & 22479472	BSP4	Tibet	29.65	91.12	2	2	3	3	2	5	1	8	3	5	4	3	4	2	4	6	8	4	3	4	4	4	3	3
B1072	2006-2010	Dong, H. *et al.* & 22479472	BSP4	Tibet	29.65	91.12	2	2	3	3	2	5	1	8	3	5	4	3	4	2	4	6	8	4	3	4	4	4	3	3
B1073	2006-2010	Dong, H. *et al.* & 22479472	BSP4	Tibet	29.65	91.12	2	2	3	3	2	5	1	7	3	5	3	3	4	2	4	5	7	4	4	4	4	4	3	3
B1074	2006-2010	Dong, H. *et al.* & 22479472	BSP5	Tibet	29.65	91.12	2	2	3	3	2	5	1	5	3	5	3	2	4	2	4	6	8	2	4	5	4	4	3	3
B1075	2006-2010	Dong, H. *et al.* & 22479472	BSP4	Tibet	29.65	91.12	2	2	3	3	2	5	1	7	3	5	3	3	4	2	4	6	7	4	4	4	4	4	3	3
B1076	2006-2010	Dong, H. *et al.* & 22479472	BSP5	Tibet	29.65	91.12	2	2	3	3	2	5	1	7	3	4	3	3	4	2	4	4	8	2	4	5	4	4	3	4
B1077	2006-2010	Dong, H. *et al.* & 22479472	BSP4	Tibet	29.65	91.12	2	2	3	3	2	5	1	7	3	2	2	3	4	2	4	6	7	4	4	4	4	4	3	3
B1078	2006-2010	Dong, H. *et al.* & 22479472	BSP5	Tibet	29.65	91.12	2	2	3	3	2	5	1	8	3	4	2	3	4	2	4	6	7	2	4	5	4	4	3	3
B1079	2006-2010	Dong, H. *et al.* & 22479472	BSP4	Tibet	29.65	91.12	2	2	3	3	1	5	1	7	3	5	3	2	4	2	4	5	7	4	4	4	4	4	3	3
B1080	2006-2010	Dong, H. *et al.* & 22479472	BSP4	Tibet	29.65	91.12	2	2	3	3	1	5	1	7	3	6	3	3	4	2	4	4	7	4	4	4	4	4	3	3
B1081	2006-2010	Dong, H. *et al.* & 22479472	BSP4	Tibet	29.65	91.12	2	2	3	3	2	5	1	7	3	2	3	3	4	2	4	6	7	4	4	4	4	4	3	3
B1082	2006-2010	Dong, H. *et al.* & 22479472	BSP4	Tibet	29.65	91.12	2	2	3	3	1	5	1	7	3	4	3	3	4	2	4	5	7	4	4	4	4	4	3	3
B1083	2006-2010	Dong, H. *et al.* &	BSP5	Tibet	29.65	91.12	2	2	3	3	2	5	1	8	3	4	3	3	4	2	4	4	7	2	4	5	4	4	3	3

		22479472																												
B1084	2006-2010	Dong, H. *et al.* & 22479472	BSP4	Tibet	29.65	91.12	2	2	3	3	2	5	1	7	3	2	3	3	4	2	4	7	6	4	4	4	4	4	3	3
B1085	2006-2010	Dong, H. *et al.* & 22479472	BSP4	Tibet	29.65	91.12	2	2	3	3	1	5	1	7	3	5	3	3	4	2	4	4	7	4	4	3	4	4	3	3
B1086	2006-2010	Dong, H. *et al.* & 22479472	BSP5	Tibet	29.65	91.12	2	2	3	3	2	5	1	5	3	4	3	3	4	2	4	6	8	2	4	5	4	4	3	3
B1087	2006-2010	Dong, H. *et al.* & 22479472	BSP5	Tibet	29.65	91.12	2	2	3	3	2	5	1	7	3	4	3	3	4	2	5	6	3	2	4	5	4	4	3	3
B1088	2006-2010	Dong, H. *et al.* & 22479472	BSP5	Tibet	29.65	91.12	2	2	3	3	2	5	1	7	3	4	4	3	4	2	4	5	7	2	4	5	4	4	3	3
B1089	2006-2010	Dong, H. *et al.* & 22479472	BSP4	Tibet	29.65	91.12	2	2	3	3	1	5	1	7	3	4	3	3	4	2	4	5	7	4	4	4	4	4	3	3
B1090	2006-2010	Dong, H. *et al.* & 22479472	BSP4	Tibet	29.65	91.12	2	2	3	3	1	5	1	7	3	4	3	3	4	2	4	5	7	4	4	4	4	4	3	3
B1091	2006-2010	Dong, H. *et al.* & 22479472	BSP4	Tibet	29.65	91.12	2	2	3	3	1	5	1	7	3	5	3	2	4	2	4	5	7	4	4	4	4	4	3	3
B1092	2006-2010	Dong, H. *et al.* & 22479472	BSP5	Tibet	29.65	91.12	2	2	3	3	2	5	1	7	3	5	3	3	4	2	4	4	8	2	5	5	4	4	3	3
B1093	2006-2010	Dong, H. *et al.* & 22479472	BSP5	Tibet	29.65	91.12	2	2	2	3	2	5	1	7	3	5	3	3	4	2	4	6	8	2	4	5	4	4	3	3
B1094	2006-2010	Dong, H. *et al.* & 22479472	BSP5	Tibet	29.65	91.12	2	2	3	3	2	5	1	7	3	5	3	3	4	2	4	6	ND	2	4	5	4	4	3	3
B1095	2006-2010	Dong, H. *et al.* & 22479472	BSP4	Tibet	29.65	91.12	2	2	3	3	1	5	1	7	3	6	3	3	4	2	4	4	7	4	4	4	4	4	3	3
B1096	2006-2010	Dong, H. *et al.* & 22479472	BSP4	Tibet	29.65	91.12	2	2	3	3	2	5	1	7	3	5	3	3	4	2	4	6	7	4	4	4	4	4	3	3
B1097	2006-2010	Dong, H. *et al.* & 22479472	BSP4	Tibet	29.65	91.12	2	2	3	2	2	5	1	7	3	5	3	1	4	2	4	6	2	4	4	4	4	4	3	3
B1098	2006-2010	Dong, H. *et al.* & 22479472	BSP5	Tibet	29.65	91.12	2	2	3	3	2	5	1	8	3	4	3	3	4	2	4	6	7	2	4	5	4	4	3	3
B1099	2006-2010	Dong, H. *et al.* & 22479472	BSP4	Tibet	29.65	91.12	2	2	3	3	1	5	1	7	3	5	2	3	4	2	4	5	7	4	4	4	4	4	3	3
B1100	2006-2010	Dong, H. *et al.* & 22479472	BSPint	Tibet	29.65	91.12	2	2	2	3	2	5	1	5	3	6	3	3	4	2	4	9	8	3	4	4	4	4	3	5
B1101	2006-2010	Dong, H. *et al.* & 22479472	BSP4	Tibet	29.65	91.12	2	2	3	3	1	5	1	7	3	5	3	2	4	2	4	5	7	4	4	4	4	4	3	3
B1102	2006-2010	Dong, H. *et al.* & 22479472	BSP4	Tibet	29.65	91.12	2	2	3	3	2	5	1	7	3	5	3	3	4	2	4	6	7	4	3	5	4	4	3	3
B1103	2006-2010	Dong, H. *et al.* & 22479472	BSP5	Tibet	29.65	91.12	2	2	3	3	2	5	1	8	3	4	3	3	4	2	4	6	7	2	4	5	4	4	3	3
B1104	2006-2010	Dong, H. *et al.* & 22479472	BSP4	Tibet	29.65	91.12	2	2	3	3	2	5	1	7	3	2	3	3	4	2	4	6	7	4	4	4	4	4	3	3
B1105	2006-2010	Dong, H. *et al.* & 22479472	BSP1	Tibet	29.65	91.12	2	3	2	3	2	5	1	6	3	3	3	4	3	1	4	3	7	2	2	3	4	2	3	4
B1106	2006-2010	Dong, H. *et al.* & 22479472	BSP4	Tibet	29.65	91.12	2	2	3	3	2	5	1	7	3	2	2	3	4	2	3	6	7	4	4	4	4	4	3	3
B1107	2006-2010	Dong, H. *et al.* & 22479472	BSP5	Tibet	29.65	91.12	2	2	3	3	2	5	1	7	3	6	3	3	4	2	4	6	8	2	4	5	4	2	3	3
B1108	2006-2010	Dong, H. *et al.* & 22479472	BSP5	Tibet	29.65	91.12	2	2	3	3	2	5	1	7	3	5	3	3	4	2	4	6	8	2	4	5	4	4	3	3
B1109	2006-2010	Dong, H. *et al.* & 22479472	BSP5	Tibet	29.65	91.12	2	2	3	3	2	5	1	8	3	4	3	3	4	2	4	6	7	2	4	5	4	4	3	3
B1110	2006-2010	Dong, H. *et al.* & 22479472	BSP5	Tibet	29.65	91.12	2	2	3	3	2	5	1	7	3	5	3	3	4	2	4	4	8	2	5	5	4	4	3	3
B1111	2006-2010	Dong, H. *et al.* & 22479472	BSP4	Tibet	29.65	91.12	2	2	3	3	1	5	1	7	3	3	3	3	4	2	4	6	7	4	4	4	4	4	3	3
B1112	2006-2010	Dong, H. *et al.* & 22479472	BSP4	Tibet	29.65	91.12	2	2	3	2	1	5	1	6	3	6	2	3	4	2	4	5	7	4	4	4	4	4	3	3
B1113	2006-2010	Dong, H. *et al.* & 22479472	BSP4	Tibet	29.65	91.12	2	1	3	2	2	5	1	7	3	2	3	3	2	2	4	7	7	4	4	4	4	4	3	3
B1114	2006-2010	Dong, H. *et al.* & 22479472	BSP4	Tibet	29.65	91.12	2	2	3	2	2	5	1	7	3	5	3	3	4	2	4	6	7	4	4	4	4	4	3	3
B1115	2006-2010	Dong, H. *et al.* & 22479472	BSP4	Tibet	29.65	91.12	2	2	3	2	1	5	1	7	3	6	3	4	4	2	4	5	7	1	4	4	4	4	3	3
B1116	2006-2010	Dong, H. *et al.* & 22479472	BSP4	Tibet	29.65	91.12	2	2	3	2	2	5	1	7	3	5	2	3	4	2	4	6	7	4	4	4	4	4	3	3
B1117	2006-2010	Dong, H. *et al.* & 22479472	BSP4	Tibet	29.65	91.12	2	2	3	2	1	5	1	7	3	5	3	3	4	2	4	4	7	4	4	4	4	4	3	2
B1118	2006-2010	Dong, H. *et al.* & 22479472	BSP4	Tibet	29.65	91.12	2	2	3	2	1	5	1	7	3	5	3	3	4	2	4	5	1	4	4	4	4	4	3	3
B1119	2006-2010	Dong, H. *et al.* & 22479472	BSP5	Tibet	29.65	91.12	2	2	3	3	2	5	1	7	3	5	3	4	4	2	4	5	8	2	4	5	4	4	3	3
B1120	2006-2010	Dong, H. *et al.* & 22479472	BSP5	Tibet	29.65	91.12	2	2	3	3	2	5	1	7	3	5	2	3	4	2	4	6	ND	2	4	5	4	4	3	3
B1121	2006-2010	Dong, H. *et al.* & 22479472	BSP4	Tibet	29.65	91.12	2	2	3	3	2	5	1	5	3	5	3	3	4	2	4	6	7	4	4	4	4	4	3	3
B1122	2006-2010	Dong, H. *et al.* & 22479472	BSP4	Tibet	29.65	91.12	2	2	3	3	2	5	1	5	3	5	3	3	4	2	4	6	7	4	4	4	4	4	3	3
B1123	2006-2010	Dong, H. *et al.* & 22479472	BSP4	Tibet	29.65	91.12	2	2	3	3	1	5	1	7	3	6	3	3	3	2	4	4	7	4	4	5	4	4	3	3
B1124	2006-2010	Dong, H. *et al.* &	BSP4	Tibet	29.65	91.12	2	2	3	3	2	5	1	8	3	5	3	3	4	2	4	6	8	4	3	4	4	4	3	3

		22479472																												
B1125	2006-2010	Dong, H. *et al.* & 22479472	BSP4	Tibet	29.65	91.12	2	2	3	3	2	5	1	7	3	2	4	3	4	2	4	7	7	4	4	4	4	4	3	3
B1126	2006-2010	Dong, H. *et al.* & 22479472	BSP4	Tibet	29.65	91.12	2	2	3	3	1	5	1	7	3	5	3	3	5	2	4	5	7	4	4	4	4	4	3	3
B1127	2006-2010	Dong, H. *et al.* & 22479472	BSP4	Tibet	29.65	91.12	2	2	3	3	1	5	1	7	3	5	3	3	4	2	4	5	7	4	4	4	4	4	3	3
B1128	2006-2010	Dong, H. *et al.* & 22479472	BSP4	Tibet	29.65	91.12	2	2	3	3	2	5	1	7	3	2	3	3	4	2	4	7	7	4	3	4	4	4	3	3
B1129	2006-2010	Dong, H. *et al.* & 22479472	BSP4	Tibet	29.65	91.12	2	3	3	3	1	5	1	7	3	5	3	3	4	2	4	4	5	4	4	4	4	4	3	3
B1130	2006-2010	Dong, H. *et al.* & 22479472	BSP4	Tibet	29.65	91.12	2	1	3	3	1	5	1	7	3	2	3	3	4	2	4	6	7	4	4	4	4	4	3	3
B1131	2006-2010	Dong, H. *et al.* & 22479472	BSP4	Tibet	29.65	91.12	2	3	3	3	2	5	1	7	3	5	3	3	4	2	4	6	7	4	4	4	4	4	3	3
B1132	2006-2010	Dong, H. *et al.* & 22479472	BSP5	Tibet	29.65	91.12	2	1	3	3	2	5	1	7	3	5	3	3	4	2	4	5	7	2	4	5	4	4	3	3
B1133	2006-2010	Dong, H. *et al.* & 22479472	BSP4	Tibet	29.65	91.12	2	2	3	3	1	5	1	7	3	4	3	3	4	2	4	5	7	4	4	4	4	4	3	3
B1134	2006-2010	Dong, H. *et al.* & 22479472	BSP4	Tibet	29.65	91.12	2	2	3	3	1	5	1	7	4	5	3	3	4	2	4	4	7	4	4	4	4	4	3	2
B1135	2006-2010	Dong, H. *et al.* & 22479472	BSP5	Tibet	29.65	91.12	2	2	3	3	2	5	1	5	3	5	3	3	4	2	4	6	8	2	4	5	4	4	3	3
B1136	2006-2010	Dong, H. *et al.* & 22479472	BSP1	Tibet	29.65	91.12	2	2	2	3	2	5	1	5	4	3	3	3	4	1	4	4	8	2	2	3	4	2	3	4
B1137	2006-2010	Dong, H. *et al.* & 22479472	BSP4	Tibet	29.65	91.12	2	2	3	3	2	5	1	7	3	5	2	3	4	2	4	6	7	4	4	4	4	4	3	3
B1138	2006-2010	Dong, H. *et al.* & 22479472	BSP4	Tibet	29.65	91.12	2	2	3	3	2	5	1	7	3	2	3	3	4	2	4	7	7	4	4	4	4	4	3	3
B1139	2006-2010	Dong, H. *et al.* & 22479472	BSP4	Tibet	29.65	91.12	2	2	3	3	1	5	1	7	3	6	3	4	4	2	4	5	7	1	4	4	4	4	3	3
B1140	2006-2010	Dong, H. *et al.* & 22479472	BSP4	Tibet	29.65	91.12	2	2	3	3	2	5	1	8	3	5	3	3	3	2	4	6	7	4	4	4	4	4	3	3
B1141	2006-2010	Dong, H. *et al.* & 22479472	BSP4	Tibet	29.65	91.12	2	2	3	3	1	6	1	7	3	5	2	3	4	2	4	9	7	2	4	4	4	4	3	3
B1142	2006-2010	Dong, H. *et al.* & 22479472	BSP4	Tibet	29.65	91.12	2	2	3	3	1	5	1	7	3	5	3	3	4	2	4	5	6	4	4	4	4	4	3	3
B1143	2006-2010	Dong, H. *et al.* & 22479472	BSPint	Tibet	29.65	91.12	2	2	2	3	2	5	1	3	3	6	3	3	4	2	4	5	6	3	4	4	4	4	3	5
B1144	2006-2010	Dong, H. *et al.* & 22479472	BSP5	Tibet	29.65	91.12	2	2	3	3	2	5	1	7	3	4	3	3	4	2	4	6	7	2	4	5	4	4	3	3
B1145	2006-2010	Dong, H. *et al.* & 22479472	BSP5	Tibet	29.65	91.12	2	2	3	3	2	5	1	6	3	5	3	3	4	2	4	6	8	2	4	6	4	4	3	3
B1146	2006-2010	Dong, H. *et al.* & 22479472	BSP4	Tibet	29.65	91.12	2	2	3	3	1	5	1	7	3	6	2	3	4	2	4	4	7	4	4	4	4	4	3	3
B1147	2006-2010	Dong, H. *et al.* & 22479472	BSPint	Tibet	29.65	91.12	2	2	3	3	2	5	1	7	3	5	3	3	4	2	4	6	7	2	4	4	4	4	3	3
B1148	2006-2010	Dong, H. *et al.* & 22479472	BSP5	Tibet	29.65	91.12	2	2	3	3	2	5	1	7	3	6	3	3	4	2	3	6	8	2	4	5	4	4	3	2
B1149	2006-2010	Dong, H. *et al.* & 22479472	BSP4	Tibet	29.65	91.12	2	2	3	3	1	5	1	7	3	2	3	2	4	2	4	6	7	4	4	4	4	4	3	3
B1150	2006-2010	Dong, H. *et al.* & 22479472	BSP4	Tibet	29.65	91.12	2	2	3	3	1	5	1	7	3	5	3	3	4	2	3	5	7	2	4	4	4	4	3	3
B1151	2006-2010	Dong, H. *et al.* & 22479472	BSP4	Tibet	29.65	91.12	2	2	3	3	2	5	1	7	3	2	3	3	4	2	4	7	7	4	4	4	4	4	3	3
B1152	2006-2010	Dong, H. *et al.* & 22479472	BSP4	Tibet	29.65	91.12	2	2	3	3	1	5	1	7	3	5	3	3	4	3	4	5	7	4	4	3	4	4	3	3
B1153	2006-2010	Dong, H. *et al.* & 22479472	BSP5	Tibet	29.65	91.12	2	1	3	3	2	5	1	7	3	5	3	3	4	2	4	6	8	2	4	5	4	4	3	3
B1154	2006-2010	Dong, H. *et al.* & 22479472	BSP4	Tibet	29.65	91.12	2	2	3	4	2	5	1	6	3	5	3	3	4	2	4	7	7	4	4	4	4	4	3	3
B1155	2006-2010	Dong, H. *et al.* & 22479472	BSP5	Tibet	29.65	91.12	2	2	3	3	2	5	1	7	3	4	3	3	4	2	4	5	7	2	4	5	4	4	3	3
B1156	2006-2010	Dong, H. *et al.* & 22479472	BSP4	Tibet	29.65	91.12	2	2	3	3	1	5	1	7	3	5	3	3	4	2	4	4	6	2	4	4	4	4	3	2
B1157	2006-2010	Dong, H. *et al.* & 22479472	BSP5	Tibet	29.65	91.12	2	2	3	3	2	5	1	8	3	6	3	3	4	2	3	6	8	2	4	5	4	4	3	2
B1158	2006-2010	Dong, H. *et al.* & 22479472	BSP4	Tibet	29.65	91.12	2	2	3	3	2	5	1	7	2	5	3	3	4	2	4	5	ND	4	4	4	4	4	3	3
B1159	2006-2010	Dong, H. *et al.* & 22479472	BSP1	Tibet	29.65	91.12	2	2	2	3	2	5	1	5	3	3	3	3	3	1	4	3	8	2	2	3	4	2	3	4
B1160	2006-2010	Dong, H. *et al.* & 22479472	BSP5	Tibet	29.65	91.12	2	1	3	2	2	5	1	7	3	5	2	3	4	2	4	3	8	2	3	5	4	4	3	3
B1161	2006-2010	Dong, H. *et al.* & 22479472	BSP4	Tibet	29.65	91.12	2	2	3	2	1	5	1	7	3	6	2	3	4	2	4	3	8	2	4	4	4	4	2	3
B1162	2006-2010	Dong, H. *et al.* & 22479472	BSP4	Tibet	29.65	91.12	2	2	3	2	1	5	1	7	3	5	3	3	2	2	4	5	7	2	4	4	4	4	3	3
B1163	2006-2010	Dong, H. *et al.* & 22479472	BSP5	Tibet	29.65	91.12	2	2	3	3	2	5	1	5	3	5	3	4	4	2	4	6	8	2	4	5	4	4	3	3
B1164	2006-2010	Dong, H. *et al.* & 22479472	BSPint	Tibet	29.65	91.12	2	2	3	3	1	5	1	7	3	5	3	3	4	2	4	6	1	2	4	5	4	4	3	3
B1165	2006-2010	Dong, H. *et al.* &	BSP5	Tibet	29.65	91.12	2	2	3	3	2	5	1	6	3	5	3	3	4	2	3	6	8	2	4	5	4	4	3	3

		22479472																												
B1166	2006-2010	Dong, H. *et al.* & 22479472	BSP4	Tibet	29.65	91.12	2	2	3	3	2	5	1	7	3	4	3	2	4	2	4	9	6	4	4	4	4	4	3	3
B1167	2006-2010	Dong, H. *et al.* & 22479472	BSP5	Tibet	29.65	91.12	2	2	3	3	2	5	1	5	3	3	2	4	4	2	4	6	8	2	4	5	4	4	3	3
B1168	2006-2010	Dong, H. *et al.* & 22479472	BSP5	Tibet	29.65	91.12	2	2	3	3	2	5	1	4	3	5	3	3	4	2	4	6	8	2	5	5	4	4	3	3
B1169	2006-2010	Dong, H. *et al.* & 22479472	BSP4	Tibet	29.65	91.12	2	2	3	2	1	5	1	7	3	5	3	3	2	2	4	5	7	4	4	4	4	4	3	3
B1170	2006-2010	Dong, H. *et al.* & 22479472	BSP5	Tibet	29.65	91.12	2	2	3	3	2	5	1	7	3	5	3	3	4	2	4	5	8	2	4	5	4	4	3	3
B1171	2006-2010	Dong, H. *et al.* & 22479472	BSP4	Tibet	29.65	91.12	2	2	3	3	2	5	1	7	2	5	3	3	4	2	4	6	6	3	3	4	4	4	3	3
B1172	2006-2010	Dong, H. *et al.* & 22479472	BSP4	Tibet	29.65	91.12	2	2	3	3	2	5	1	7	3	5	3	3	4	2	4	6	7	4	4	4	4	4	3	3
B1173	2006-2010	Dong, H. *et al.* & 22479472	BSP4	Tibet	29.65	91.12	2	2	3	3	1	5	1	7	3	5	3	3	4	2	4	5	7	4	3	4	2	4	3	3
B1174	2006-2010	Dong, H. *et al.* & 22479472	BSP5	Tibet	29.65	91.12	2	2	3	3	2	5	1	7	3	5	3	3	4	2	4	6	6	2	4	5	4	4	3	3
B1175	2006-2010	Dong, H. *et al.* & 22479472	BSP4	Tibet	29.65	91.12	2	2	3	3	1	5	1	7	3	5	3	2	4	2	4	4	7	2	4	4	4	3	3	2
B1176	2006-2010	Dong, H. *et al.* & 22479472	BSP5	Tibet	29.65	91.12	2	4	3	3	2	5	1	6	3	5	3	3	4	2	4	6	8	2	4	4	4	4	3	3
B1177	2006-2010	Dong, H. *et al.* & 22479472	BSP4	Tibet	29.65	91.12	2	2	3	3	2	5	1	7	3	2	3	1	4	2	2	3	7	4	4	4	4	2	3	3
B1178	2006-2010	Dong, H. *et al.* & 22479472	BSP4	Tibet	29.65	91.12	2	2	3	3	2	5	1	6	3	5	4	3	4	2	4	6	7	4	4	4	4	4	3	3
B1179	2006-2010	Dong, H. *et al.* & 22479472	BSP4	Tibet	29.65	91.12	2	2	3	3	1	5	1	7	3	6	2	3	4	2	4	5	7	4	4	4	4	4	3	3
B1180	2006-2010	Dong, H. *et al.* & 22479472	BSP5	Tibet	29.65	91.12	2	2	3	3	2	5	1	7	3	5	3	3	4	2	4	4	8	2	4	5	4	4	3	3
B1181	2006-2010	Dong, H. *et al.* & 22479472	BSP4	Tibet	29.65	91.12	2	2	3	3	2	5	1	7	3	2	3	1	4	2	4	7	7	4	4	4	4	4	3	3
B1182	2006-2010	Dong, H. *et al.* & 22479472	BSP4	Tibet	29.65	91.12	2	2	3	3	2	5	1	7	3	5	3	3	4	2	4	6	7	4	4	4	4	4	3	3
B1183	2006-2010	Dong, H. *et al.* & 22479472	BSP4	Tibet	29.65	91.12	2	2	3	3	1	5	1	7	3	6	3	3	4	2	4	4	7	4	4	4	4	4	3	3
B1184	2006-2010	Dong, H. *et al.* & 22479472	BSP4	Tibet	29.65	91.12	2	2	3	3	2	5	1	7	3	5	3	4	4	2	4	6	8	4	4	4	4	4	3	3
B1185	2006-2010	Dong, H. *et al.* & 22479472	BSP5	Tibet	29.65	91.12	2	2	3	3	2	5	1	7	3	5	3	3	4	2	4	6	8	2	4	5	4	4	3	3
B1186	2006-2010	Dong, H. *et al.* & 22479472	BSP5	Tibet	29.65	91.12	2	2	3	3	2	5	1	7	3	4	3	3	4	2	4	6	8	2	3	5	4	4	3	3
B1187	2006-2010	Dong, H. *et al.* & 22479472	BSP4	Tibet	29.65	91.12	2	2	3	3	2	5	1	7	3	2	3	3	4	2	4	7	7	4	4	4	4	4	3	3
B1188	2006-2010	Dong, H. *et al.* & 22479472	BSP5	Tibet	29.65	91.12	2	2	3	3	2	5	1	7	3	5	2	3	4	2	4	6	8	2	4	6	4	4	3	3
B1189	2006-2010	Dong, H. *et al.* & 22479472	BSP5	Tibet	29.65	91.12	2	2	3	3	2	5	1	8	3	4	3	3	4	2	4	6	7	2	5	5	4	4	3	3
B1190	2006-2010	Dong, H. *et al.* & 22479472	BSP4	Tibet	29.65	91.12	2	2	3	3	1	5	1	7	3	6	3	4	4	2	4	4	7	4	4	4	4	4	3	3
B1191	2006-2010	Dong, H. *et al.* & 22479472	BSP4	Tibet	29.65	91.12	2	2	3	3	2	5	1	7	3	2	3	3	4	2	4	6	7	4	4	3	4	4	3	3
B1192	2006-2010	Dong, H. *et al.* & 22479472	BSP5	Tibet	29.65	91.12	2	2	3	3	2	5	1	8	3	4	3	3	4	2	4	6	7	2	4	5	4	4	2	3
B1193	2006-2010	Dong, H. *et al.* & 22479472	BSP4	Tibet	29.65	91.12	2	2	3	4	2	5	1	6	3	5	3	3	4	2	4	7	7	4	4	4	4	4	3	3
B1194	2006-2010	Dong, H. *et al.* & 22479472	BSP5	Tibet	29.65	91.12	2	2	3	3	2	5	1	5	3	5	3	3	4	2	4	6	8	2	4	5	4	4	3	3
B1195	2006-2010	Dong, H. *et al.* & 22479472	BSP5	Tibet	29.65	91.12	2	2	3	3	2	5	1	4	3	5	3	4	4	2	4	6	8	2	4	5	4	4	3	3
B1196	2006-2010	Dong, H. *et al.* & 22479472	BSP5	Tibet	29.65	91.12	2	2	3	3	2	5	1	7	3	5	3	3	4	2	4	5	8	2	4	5	4	4	3	3
B1197	2006-2010	Dong, H. *et al.* & 22479472	BSP5	Tibet	29.65	91.12	2	2	3	2	2	6	1	5	3	5	3	3	4	2	4	5	ND	4	4	6	4	4	3	3
B1198	2006-2010	Dong, H. *et al.* & 22479472	BSP5	Tibet	29.65	91.12	2	2	3	3	2	5	1	7	3	5	3	3	4	2	4	5	8	2	4	5	4	4	3	3
B1199	2006-2010	Dong, H. *et al.* & 22479472	BSP5	Tibet	29.65	91.12	2	2	3	3	2	5	1	7	3	5	3	3	4	2	4	5	8	2	4	5	4	4	3	3
B1200	2006-2010	Dong, H. *et al.* & 22479472	BSP4	Tibet	29.65	91.12	2	2	3	3	2	5	1	7	3	2	3	3	4	2	4	6	7	4	4	4	4	4	3	3
B1201	2006-2010	Dong, H. *et al.* & 22479472	BSP4	Tibet	29.65	91.12	2	2	3	3	2	5	1	7	3	5	3	3	4	2	4	6	7	4	4	4	4	4	3	3
B1202	2006-2010	Dong, H. *et al.* & 22479472	BSP5	Tibet	29.65	91.12	2	2	3	3	2	5	1	8	3	4	3	3	4	2	4	6	7	2	4	5	4	4	3	3
B1203	2006-2010	Dong, H. *et al.* & 22479472	BSP5	Tibet	29.65	91.12	2	2	3	3	2	5	1	7	3	5	4	3	4	2	5	6	3	2	4	5	4	4	3	3
B1204	2006-2010	Dong, H. *et al.* & 22479472	BSP5	Tibet	29.65	91.12	2	2	3	3	2	5	1	7	3	4	3	3	4	2	4	5	7	2	4	5	4	4	3	3
B1205	2006-2010	Dong, H. *et al.* & 22479472	BSP4	Tibet	29.65	91.12	2	2	3	3	1	5	1	7	3	6	3	3	4	2	4	4	7	4	3	4	4	4	3	3
B1206	2006-2010	Dong, H. *et al.* &	BSP5	Tibet	29.65	91.12	2	2	3	3	2	5	1	8	3	4	3	3	4	2	4	6	7	2	4	4	4	4	3	3

		22479472																												
B1207	2006-2010	Dong, H. *et al.* & 22479472	BSP4	Tibet	29.65	91.12	2	2	3	3	1	5	1	7	3	6	3	4	4	2	4	4	7	4	4	4	4	4	3	3
B1208	2006-2010	Dong, H. *et al.* & 22479472	BSP4	Tibet	29.65	91.12	2	2	3	3	1	5	1	7	3	6	3	4	4	2	4	5	7	2	4	4	4	4	3	3
B1209	2006-2010	Dong, H. *et al.* & 22479472	BSP4	Tibet	29.65	91.12	2	2	3	3	2	5	1	7	3	5	3	3	4	2	4	6	7	4	4	4	4	4	3	3
B1210	2006-2010	Dong, H. *et al.* & 22479472	BSP5	Tibet	29.65	91.12	2	2	3	3	2	5	1	2	3	5	3	3	4	2	4	6	8	2	4	5	4	4	3	3
B1211	2006-2010	Dong, H. *et al.* & 22479472	BSP4	Tibet	29.65	91.12	2	2	3	3	2	5	1	7	3	5	3	3	4	2	4	6	7	4	4	4	4	4	3	3
B1212	2006-2010	Dong, H. *et al.* & 22479472	BSP4	Tibet	29.65	91.12	2	2	3	3	1	5	1	7	3	4	3	3	4	2	4	5	7	4	4	4	4	4	3	3
B1213	2006-2010	Dong, H. *et al.* & 22479472	BSP4	Tibet	29.65	91.12	2	2	3	3	1	5	1	7	3	6	3	3	4	2	4	4	7	4	2	3	4	4	3	3
B1214	2006-2010	Dong, H. *et al.* & 22479472	BSP4	Tibet	29.65	91.12	2	2	3	3	2	5	1	7	3	5	3	3	4	2	4	6	7	4	4	4	4	4	3	3
B1215	2006-2010	Dong, H. *et al.* & 22479472	BSP5	Tibet	29.65	91.12	2	2	3	3	2	5	1	7	3	5	3	3	4	2	4	4	7	2	4	5	4	4	3	3
B1216	2006-2010	Dong, H. *et al.* & 22479472	BSP4	Tibet	29.65	91.12	2	2	3	3	1	5	1	7	3	5	3	3	4	3	4	5	8	4	4	3	4	4	3	3
B1217	2006-2010	Dong, H. *et al.* & 22479472	BSP4	Tibet	29.65	91.12	2	2	3	3	1	5	1	7	3	5	3	3	4	2	4	5	7	4	4	4	4	4	3	3
B1218	2006-2010	Dong, H. *et al.* & 22479472	BSP4	Tibet	29.65	91.12	2	2	3	3	1	5	1	7	3	5	3	3	4	2	4	5	7	2	4	4	4	4	3	3
B1219	2006-2010	Dong, H. *et al.* & 22479472	BSP5	Tibet	29.65	91.12	2	2	3	3	2	5	1	5	3	5	3	3	4	2	4	6	8	2	4	5	4	4	3	3
B1220	2006-2010	Dong, H. *et al.* & 22479472	BSP5	Tibet	29.65	91.12	2	2	3	3	2	5	1	5	3	5	3	3	4	2	4	6	7	2	4	7	4	4	3	3
B1221	2006-2010	Dong, H. *et al.* & 22479472	BSP5	Tibet	29.65	91.12	2	2	3	3	2	5	1	8	3	4	3	3	4	2	4	6	7	2	4	5	4	4	3	3
B1222	2006-2010	Dong, H. *et al.* & 22479472	BSP4	Tibet	29.65	91.12	2	2	3	3	2	5	1	7	3	5	3	3	4	2	4	6	7	4	4	4	4	4	3	3
B1223	2006-2010	Dong, H. *et al.* & 22479472	BSP4	Tibet	29.65	91.12	2	2	3	3	2	5	1	7	3	5	3	3	4	2	4	6	7	4	4	4	4	4	3	3
B1224	2006-2010	Dong, H. *et al.* & 22479472	BSP5	Tibet	29.65	91.12	2	2	3	3	2	5	1	7	3	5	3	3	4	2	4	4	8	2	5	5	4	4	3	3
B1225	2006-2010	Dong, H. *et al.* & 22479472	BSP4	Tibet	29.65	91.12	2	2	3	3	2	5	1	7	3	5	3	3	4	2	4	6	7	4	4	4	4	4	3	3
B1226	2006-2010	Dong, H. *et al.* & 22479472	BSP1	Tibet	29.65	91.12	2	3	2	3	2	5	1	5	2	3	1	2	4	2	4	2	7	2	4	1	4	2	3	1
B1227	2006-2010	Dong, H. *et al.* & 22479472	BSP4	Tibet	29.65	91.12	2	2	3	3	2	5	1	7	3	5	3	3	4	2	4	8	7	4	4	4	4	4	3	3
B1228	2006-2010	Dong, H. *et al.* & 22479472	BSP4	Tibet	29.65	91.12	2	2	3	3	1	5	1	7	3	5	3	3	4	3	4	6	7	4	4	4	4	4	3	3
B1229	2006-2010	Dong, H. *et al.* & 22479472	BSP4	Tibet	29.65	91.12	2	2	3	3	1	5	1	8	3	5	3	3	4	2	4	5	2	4	4	4	4	4	3	3
B1230	2006-2010	Dong, H. *et al.* & 22479472	BSP4	Tibet	29.65	91.12	2	2	3	3	1	5	1	7	3	5	3	3	4	2	4	4	7	4	4	4	4	4	3	2
B1231	2006-2010	Dong, H. *et al.* & 22479472	BSP5	Tibet	29.65	91.12	2	2	3	3	2	5	1	5	3	5	3	3	4	2	4	6	8	2	4	5	4	4	3	3
B1232	2006-2010	Dong, H. *et al.* & 22479472	BSP4	Tibet	29.65	91.12	2	2	3	3	2	5	1	7	3	5	3	3	4	2	4	6	7	4	5	4	4	4	3	3
B1233	2006-2010	Dong, H. *et al.* & 22479472	BSP4	Tibet	29.65	91.12	2	2	3	3	2	5	1	7	3	5	3	3	5	2	4	6	7	2	4	3	4	3	3	3
B1234	2006-2010	Dong, H. *et al.* & 22479472	BSP4	Tibet	29.65	91.12	2	2	3	3	2	5	1	7	3	2	3	3	4	2	4	6	7	4	4	4	4	4	3	3
B1235	2006-2010	Dong, H. *et al.* & 22479472	BSP5	Tibet	29.65	91.12	2	2	3	3	2	5	1	8	3	4	3	3	4	2	4	5	ND	2	4	5	4	4	3	2
B1236	2006-2010	Dong, H. *et al.* & 22479472	BSP4	Tibet	29.65	91.12	2	2	3	3	2	5	1	6	3	5	3	3	4	2	4	6	7	4	4	4	4	4	3	3
B1237	2006-2010	Dong, H. *et al.* & 22479472	BSP5	Tibet	29.65	91.12	2	2	3	3	2	5	1	4	3	5	3	4	4	2	4	6	8	2	4	5	4	4	3	3
B1238	2006-2010	Dong, H. *et al.* & 22479472	BSP5	Tibet	29.65	91.12	2	1	3	2	2	5	1	7	3	5	3	3	4	2	4	3	8	2	3	5	4	4	3	3
B1239	2006-2010	Dong, H. *et al.* & 22479472	BSP4	Tibet	29.65	91.12	2	2	3	4	2	5	1	7	3	5	3	3	4	2	4	6	7	4	4	4	4	4	3	3
B1240	2006-2010	Dong, H. *et al.* & 22479472	BSP5	Tibet	29.65	91.12	2	2	3	3	2	5	1	8	3	6	3	3	4	2	3	6	8	2	4	5	4	4	3	2
B1241	2006-2010	Dong, H. *et al.* & 22479472	BSPint	Tibet	29.65	91.12	2	2	3	4	2	5	1	6	3	4	2	3	4	2	4	6	7	3	3	4	4	4	3	3
B1242	2006-2010	Dong, H. *et al.* & 22479472	BSP4	Tibet	29.65	91.12	2	2	3	3	1	5	1	7	3	4	3	3	4	2	4	5	7	4	3	4	4	4	3	3
B1243	2006-2010	Dong, H. *et al.* & 22479472	BSP4	Tibet	29.65	91.12	2	2	3	3	1	5	1	7	3	6	3	4	4	2	4	4	7	2	4	4	4	4	3	3
B1244	2006-2010	Dong, H. *et al.* & 22479472	BSP4	Tibet	29.65	91.12	2	4	3	3	2	5	1	7	3	5	4	3	4	2	4	6	7	4	4	4	4	4	3	3
B1245	2006-2010	Dong, H. *et al.* & 22479472	BSP4	Tibet	29.65	91.12	2	2	3	3	1	5	1	7	3	6	3	3	4	2	4	3	8	4	4	4	4	4	2	3
B1246	2006-2010	Dong, H. *et al.* & 22479472	BSP4	Tibet	29.65	91.12	2	2	3	3	2	5	1	7	3	4	3	3	4	2	4	8	6	4	4	4	4	4	3	3
B1247	2006-2010	Dong, H. *et al.* &	BSP5	Tibet	29.65	91.12	2	2	3	3	2	5	1	5	3	5	3	3	4	2	4	6	8	2	4	5	4	4	3	3

		22479472																												
B1248	2006-2010	Dong, H. *et al.* & 22479472	BSP4	Tibet	29.65	91.12	2	2	3	3	1	5	1	7	3	5	3	3	2	2	4	4	8	4	4	4	4	4	3	3
B1249	2006-2010	Dong, H. *et al.* & 22479472	BSP4	Tibet	29.65	91.12	2	2	3	3	2	5	1	7	2	5	3	3	4	2	4	6	7	4	4	4	4	4	3	4
B1250	2006-2010	Dong, H. *et al.* & 22479472	BSP4	Tibet	29.65	91.12	2	2	3	4	1	5	1	7	3	5	3	2	4	2	4	5	7	4	4	4	4	4	3	3
B1251	2006-2010	Dong, H. *et al.* & 22479472	BSP5	Tibet	29.65	91.12	2	2	3	3	2	5	1	8	3	4	3	3	4	2	4	6	7	2	4	5	4	4	3	3
B1252	2006-2010	Dong, H. *et al.* & 22479472	BSP4	Tibet	29.65	91.12	2	2	3	4	2	5	1	7	3	2	3	3	4	2	4	6	7	4	4	4	4	4	3	5
B1253	2006-2010	Dong, H. *et al.* & 22479472	BSP4	Tibet	29.65	91.12	2	2	3	3	1	5	1	7	3	6	3	3	4	2	4	4	8	4	4	4	4	4	3	3
B1254	2006-2010	Dong, H. *et al.* & 22479472	BSPint	Tibet	29.65	91.12	2	2	3	3	2	5	1	6	3	4	3	3	4	2	4	4	6	3	3	4	4	4	3	3
B1255	2006-2010	Dong, H. *et al.* & 22479472	BSP4	Tibet	29.65	91.12	2	2	3	3	2	5	1	7	2	5	3	3	4	2	4	6	7	4	4	4	4	4	3	4
B1256	2006-2010	Dong, H. *et al.* & 22479472	BSP4	Tibet	29.65	91.12	2	2	3	3	2	5	1	7	3	5	3	3	4	2	4	6	7	4	4	4	4	4	3	3
B1257	2006-2010	Dong, H. *et al.* & 22479472	BSP5	Tibet	29.65	91.12	2	4	3	3	2	5	1	6	3	5	3	1	4	2	4	6	8	2	4	4	4	4	3	3
B1258	2006-2010	Dong, H. *et al.* & 22479472	BSP5	Tibet	29.65	91.12	2	2	3	3	2	5	1	7	3	5	3	3	4	2	4	5	7	2	4	5	4	4	3	3
B1259	2006-2010	Dong, H. *et al.* & 22479472	BSP4	Tibet	29.65	91.12	2	2	3	3	2	5	1	ND	3	5	3	3	4	2	4	7	8	4	4	4	4	4	3	3
B1260	2006-2010	Dong, H. *et al.* & 22479472	BSP1	Tibet	29.65	91.12	2	2	2	3	2	5	1	4	3	3	2	4	3	1	4	3	7	2	2	3	4	2	3	5
B1261	2006-2010	Dong, H. *et al.* & 22479472	BSP4	Tibet	29.65	91.12	2	2	3	3	1	5	1	7	3	5	3	3	4	2	4	4	7	4	4	4	4	4	3	2
B1262	2006-2010	Dong, H. *et al.* & 22479472	BSP5	Tibet	29.65	91.12	2	2	3	3	2	5	1	7	3	5	3	3	4	2	4	5	8	2	4	5	4	4	3	3
B1263	2006-2010	Dong, H. *et al.* & 22479472	BSP5	Tibet	29.65	91.12	2	2	3	3	2	5	1	7	3	5	3	3	4	2	4	5	8	2	4	5	4	4	3	3
B1264	2006-2010	Dong, H. *et al.* & 22479472	BSP5	Tibet	29.65	91.12	2	2	3	3	2	5	1	7	3	5	3	3	4	2	4	5	8	2	4	5	4	4	3	3
B1265	2006-2010	Dong, H. *et al.* & 22479472	BSP1	Tibet	29.65	91.12	2	2	3	3	2	6	1	6	3	4	3	2	4	2	4	5	8	2	1	4	4	4	3	4
B1266	2006-2010	Dong, H. *et al.* & 22479472	BSP4	Tibet	29.65	91.12	2	2	3	3	2	5	1	7	3	5	3	3	4	2	4	6	7	4	4	4	4	4	3	3
B1267	2006-2010	Dong, H. *et al.* & 22479472	BSP4	Tibet	29.65	91.12	2	2	3	3	1	5	1	7	3	5	3	3	4	2	4	4	7	4	4	4	4	4	3	3
B1268	2006-2010	Dong, H. *et al.* & 22479472	BSP4	Tibet	29.65	91.12	2	2	3	4	2	5	1	6	3	5	3	3	4	2	4	7	7	4	4	4	4	4	3	3
B1269	2006-2010	Dong, H. *et al.* & 22479472	BSP4	Tibet	29.65	91.12	2	2	3	3	2	6	1	7	3	2	3	3	4	2	4	6	7	4	4	4	4	4	3	3
B1270	2006-2010	Dong, H. *et al.* & 22479472	BSP4	Tibet	29.65	91.12	2	2	3	3	2	5	1	4	3	5	3	3	4	2	4	6	7	4	4	4	4	4	3	3
B1271	2006-2010	Dong, H. *et al.* & 22479472	BSP4	Tibet	29.65	91.12	2	2	3	3	2	5	1	7	3	5	3	3	4	2	4	6	7	4	4	4	4	4	3	3
B1272	2006-2010	Dong, H. *et al.* & 22479472	BSP4	Tibet	29.65	91.12	2	2	3	3	2	5	1	7	5	2	3	3	4	2	4	7	7	4	4	4	4	4	3	3
B1273	2006-2010	Dong, H. *et al.* & 22479472	BSP5	Tibet	29.65	91.12	2	2	3	3	2	5	1	7	3	5	3	3	4	2	4	5	8	2	4	5	2	4	3	3
B1274	2006-2010	Dong, H. *et al.* & 22479472	BSP4	Tibet	29.65	91.12	2	2	3	3	1	5	1	7	3	6	3	3	4	2	4	4	7	4	4	4	4	4	3	3
B1275	2006-2010	Dong, H. *et al.* & 22479472	BSP4	Tibet	29.65	91.12	2	2	3	3	2	5	1	8	3	2	3	3	4	2	4	6	7	4	4	4	4	4	3	3
B1276	2006-2010	Dong, H. *et al.* & 22479472	BSP1	Tibet	29.65	91.12	2	2	2	3	2	5	1	6	3	2	2	5	3	1	4	3	8	2	1	3	4	2	3	3
B1277	2006-2010	Dong, H. *et al.* & 22479472	BSP4	Tibet	29.65	91.12	2	2	3	2	2	5	1	7	3	5	3	1	4	2	4	6	2	4	4	4	4	4	3	3
B1278	2006-2010	Dong, H. *et al.* & 22479472	BSP4	Tibet	29.65	91.12	2	2	3	3	2	5	1	7	2	5	3	3	4	2	4	6	7	4	4	4	4	4	3	4
B1279	2006-2010	Dong, H. *et al.* & 22479472	BSP4	Tibet	29.65	91.12	2	2	3	3	1	5	1	7	3	5	3	3	2	2	4	4	9	4	4	4	4	4	3	3
B1280	2006-2010	Dong, H. *et al.* & 22479472	BSP4	Tibet	29.65	91.12	2	2	3	3	1	5	1	7	3	6	3	4	4	2	4	4	7	4	4	4	4	4	3	3
B1281	2006-2010	Dong, H. *et al.* & 22479472	BSP5	Tibet	29.65	91.12	2	2	3	3	2	5	1	5	3	5	3	3	4	2	4	6	8	2	4	5	4	4	3	3
B1282	2006-2010	Dong, H. *et al.* & 22479472	BSP4	Tibet	29.65	91.12	2	2	3	3	2	5	1	7	3	5	3	3	4	2	4	6	7	4	4	4	4	4	3	3
B1283	2006-2010	Dong, H. *et al.* & 22479472	BSP4	Tibet	29.65	91.12	2	2	3	3	1	5	1	7	3	5	3	3	4	2	4	5	7	4	3	4	2	4	3	3
B1284	2006-2010	Dong, H. *et al.* & 22479472	BSP1	Tibet	29.65	91.12	2	2	2	3	2	5	1	5	2	3	1	2	4	2	4	2	7	2	4	1	4	2	3	3
B1285	2006-2010	Dong, H. *et al.* & 22479472	BSP4	Tibet	29.65	91.12	2	2	3	3	2	5	1	7	3	5	3	3	4	2	4	6	7	4	4	4	4	4	3	3
B1286	2006-2010	Dong, H. *et al.* & 22479472	BSP5	Tibet	29.65	91.12	2	2	3	3	2	5	1	7	3	4	3	3	4	2	4	6	8	2	3	5	4	4	3	3
B1287	2006-2010	Dong, H. *et al.* & 22479472	BSP4	Tibet	29.65	91.12	2	2	3	3	1	5	1	7	3	5	3	3	4	3	4	5	7	4	4	4	4	4	3	3
B1288	2006-2010	Dong, H. *et al.* &	BSP4	Tibet	29.65	91.12	2	2	3	3	1	5	1	7	3	6	3	4	4	2	4	4	7	2	4	4	4	4	3	3

		22479472																												
B1289	2006-2010	Dong, H. *et al.* & 22479472	BSP5	Tibet	29.65	91.12	2	2	3	3	2	5	1	6	3	3	3	3	4	2	4	6	8	2	4	5	4	4	3	3
B1290	2006-2010	Dong, H. *et al.* & 22479472	BSP4	Tibet	29.65	91.12	2	2	3	3	2	5	1	ND	3	5	3	3	4	2	4	6	7	4	5	4	4	4	3	3
B1291	2006-2010	Dong, H. *et al.* & 22479472	BSP4	Tibet	29.65	91.12	2	2	3	3	1	5	1	7	3	5	3	3	4	2	4	4	7	4	4	4	4	4	3	2
B1292	2006-2010	Dong, H. *et al.* & 22479472	BSP4	Tibet	29.65	91.12	2	2	3	3	2	5	1	9	3	5	3	3	4	2	4	7	7	4	4	4	4	4	3	3
B1293	2006-2010	Dong, H. *et al.* & 22479472	BSP4	Tibet	29.65	91.12	2	2	3	3	1	5	1	7	3	6	3	4	4	2	4	4	7	4	4	4	4	4	3	3
B1294	2006-2010	Dong, H. *et al.* & 22479472	BSP5	Tibet	29.65	91.12	2	2	3	3	2	5	1	7	3	5	3	3	4	2	4	5	8	2	4	5	4	4	3	3
B1295	2006-2010	Dong, H. *et al.* & 22479472	BSP4	Tibet	29.65	91.12	2	2	3	3	1	5	1	7	3	6	3	3	4	2	4	3	7	4	4	4	4	4	3	3
B1296	2006-2010	Dong, H. *et al.* & 22479472	BSP4	Tibet	29.65	91.12	2	2	3	3	1	5	1	7	3	5	3	3	4	3	4	4	7	4	4	5	4	4	3	3
B1297	2006-2010	Dong, H. *et al.* & 22479472	BSP4	Tibet	29.65	91.12	2	2	3	3	2	5	1	7	3	5	3	3	4	2	4	6	7	4	5	4	4	4	3	3
B1298	2006-2010	Dong, H. *et al.* & 22479472	BSP4	Tibet	29.65	91.12	2	3	3	2	1	5	1	7	3	5	3	3	2	2	4	5	7	4	4	4	4	4	3	3
B1299	2006-2010	Dong, H. *et al.* & 22479472	BSPint	Tibet	29.65	91.12	2	2	3	4	2	5	1	6	3	4	2	3	4	2	4	6	7	3	3	4	4	4	3	3
B1300	2006-2010	Dong, H. *et al.* & 22479472	BSP4	Tibet	29.65	91.12	2	2	3	3	2	5	1	7	3	2	3	3	4	2	4	6	7	4	4	4	4	4	3	3
B1301	2006-2010	Dong, H. *et al.* & 22479472	BSP4	Tibet	29.65	91.12	2	2	3	3	2	5	1	5	3	2	3	3	2	2	4	7	7	4	4	4	4	4	3	3
B1302	2006-2010	Dong, H. *et al.* & 22479472	BSP4	Tibet	29.65	91.12	2	2	3	3	1	5	1	7	3	6	3	4	4	2	4	4	7	2	4	4	4	4	3	3
B1303	2006-2010	Dong, H. *et al.* & 22479472	BSP4	Tibet	29.65	91.12	2	2	3	3	2	5	1	6	1	5	3	3	4	2	4	6	7	4	2	4	4	4	3	3
B1304	2006-2010	Dong, H. *et al.* & 22479472	BSP5	Tibet	29.65	91.12	2	2	3	3	2	5	1	7	3	4	3	3	4	2	4	6	8	2	4	5	4	4	3	3
B1305	2006-2010	Dong, H. *et al.* & 22479472	BSP4	Tibet	29.65	91.12	2	2	3	3	2	5	1	7	3	2	3	3	4	2	4	7	7	4	4	4	4	4	3	3
B1306	2006-2010	Dong, H. *et al.* & 22479472	BSP4	Tibet	29.65	91.12	2	2	3	3	1	5	1	7	3	6	3	4	4	2	4	4	7	4	4	4	4	4	3	3
B1307	2006-2010	Dong, H. *et al.* & 22479472	BSP5	Tibet	29.65	91.12	2	2	3	3	2	5	1	7	3	5	3	3	4	2	4	5	8	2	6	5	4	4	3	3
B1308	2006-2010	Dong, H. *et al.* & 22479472	BSP4	Tibet	29.65	91.12	2	2	3	3	1	5	1	7	3	6	3	3	4	2	4	4	7	4	4	4	4	4	3	3
B1309	2006-2010	Dong, H. *et al.* & 22479472	BSP4	Tibet	29.65	91.12	2	2	3	3	1	5	1	7	3	5	3	2	4	2	4	5	7	2	4	4	4	4	3	3
B1310	2006-2010	Dong, H. *et al.* & 22479472	BSP5	Tibet	29.65	91.12	2	2	3	3	2	5	1	7	3	5	3	3	4	2	4	5	8	2	4	4	4	4	3	3
B1311	2006-2010	Dong, H. *et al.* & 22479472	BSP5	Tibet	29.65	91.12	2	2	3	3	2	5	1	8	3	4	3	3	4	2	4	6	7	2	4	5	4	4	3	3
B1312	2006-2010	Dong, H. *et al.* & 22479472	BSP5	Tibet	29.65	91.12	2	2	3	3	2	5	1	7	3	5	2	3	4	2	4	6	8	2	4	6	4	4	3	3
B1313	2006-2010	Dong, H. *et al.* & 22479472	BSP4	Tibet	29.65	91.12	2	2	3	3	1	5	1	7	3	6	3	3	4	2	4	4	7	4	4	4	4	4	3	3
B1314	2006-2010	Dong, H. *et al.* & 22479472	BSP4	Tibet	29.65	91.12	2	2	3	3	1	5	1	7	3	5	3	3	4	2	4	5	7	4	4	4	4	4	3	3
B1315	2006-2010	Dong, H. *et al.* & 22479472	BSP4	Tibet	29.65	91.12	2	2	3	3	1	5	1	7	3	5	3	3	4	2	4	5	7	4	4	4	4	4	3	3
B1316	2006-2010	Dong, H. *et al.* & 22479472	BSP5	Tibet	29.65	91.12	2	2	3	3	2	5	1	7	3	5	3	3	4	2	4	5	8	2	4	5	4	4	3	3
B1317	2006-2010	Dong, H. *et al.* & 22479472	BSP4	Tibet	29.65	91.12	2	2	3	3	2	5	1	7	3	5	3	3	4	2	4	6	7	4	4	4	4	4	3	3
B1318	2006-2010	Dong, H. *et al.* & 22479472	BSP1	Tibet	29.65	91.12	2	2	2	2	2	5	1	5	3	3	2	3	1	1	4	3	5	2	2	3	4	2	3	3
B1319	2006-2010	Dong, H. *et al.* & 22479472	BSP4	Tibet	29.65	91.12	2	2	3	3	2	5	1	7	3	5	3	3	4	2	4	6	8	4	4	4	4	4	3	3
B1320	2006-2010	Dong, H. *et al.* & 22479472	BSP4	Tibet	29.65	91.12	2	2	3	3	2	5	1	7	2	5	3	3	4	2	4	6	7	4	4	4	4	4	3	4
B1321	2006-2010	Dong, H. *et al.* & 22479472	BSP4	Tibet	29.65	91.12	2	2	3	3	2	5	1	7	5	2	3	3	4	2	4	7	7	4	4	4	4	4	3	3
B1322	2006-2010	Dong, H. *et al.* & 22479472	BSP5	Tibet	29.65	91.12	2	2	3	3	2	5	1	8	3	4	3	3	4	2	4	5	ND	2	4	5	4	4	3	3
B1323	2006-2010	Dong, H. *et al.* & 22479472	BSP4	Tibet	29.65	91.12	2	2	3	3	2	5	1	7	3	5	3	3	4	2	4	6	7	4	4	4	4	4	3	3
B1324	2006-2010	Dong, H. *et al.* & 22479472	BSP4	Tibet	29.65	91.12	2	2	3	2	1	5	1	7	3	5	3	3	2	2	4	5	7	4	3	4	4	4	3	3
B1325	2006-2010	Dong, H. *et al.* & 22479472	BSP4	Tibet	29.65	91.12	2	2	3	3	2	5	1	7	3	2	3	3	4	2	4	7	7	4	4	4	4	4	3	3
B1326	2006-2010	Dong, H. *et al.* & 22479472	BSP5	Tibet	29.65	91.12	2	2	3	3	2	5	1	7	3	5	3	3	4	2	4	6	8	2	5	5	4	4	3	3
B1327	2006-2010	Dong, H. *et al.* & 22479472	BSPint	Tibet	29.65	91.12	2	2	3	4	2	5	1	6	3	4	2	3	4	2	4	6	7	3	3	4	4	4	3	3
B1328	2006-2010	Dong, H. *et al.* & 22479472	BSP5	Tibet	29.65	91.12	2	2	3	3	2	5	1	7	3	4	3	3	4	2	4	5	7	2	4	5	4	4	3	3
B1329	2006-2010	Dong, H. *et al.* &	BSP4	Tibet	29.65	91.12	2	2	3	3	1	5	1	7	3	6	3	3	4	2	4	4	7	4	4	4	4	4	3	3

		22479472																												
B1330	2006-2010	Dong, H. *et al.* & 22479472	BSP5	Tibet	29.65	91.12	2	2	3	3	2	5	1	7	3	5	3	3	4	2	4	6	8	2	4	5	4	4	3	3
B1331	2006-2010	Dong, H. *et al.* & 22479472	BSP1	Tibet	29.65	91.12	2	2	2	3	2	5	1	5	3	3	2	3	3	1	4	1	6	2	2	3	4	2	3	4
B1332	2006-2010	Dong, H. *et al.* & 22479472	BSPint	Tibet	29.65	91.12	2	2	3	3	2	5	1	7	3	5	3	3	4	2	4	7	ND	2	4	4	4	4	3	3
B1333	2006-2010	Dong, H. *et al.* & 22479472	BSP5	Tibet	29.65	91.12	2	2	3	3	2	5	1	4	3	5	3	4	4	2	4	6	8	2	4	5	4	4	3	3
B1334	2006-2010	Dong, H. *et al.* & 22479472	BSP5	Tibet	29.65	91.12	2	2	3	3	2	5	1	7	3	4	3	3	4	2	4	6	8	2	4	5	4	4	3	3
B1335	2006-2010	Dong, H. *et al.* & 22479472	BSP4	Tibet	29.65	91.12	2	2	3	3	2	5	1	7	3	5	3	3	4	2	4	6	7	4	4	4	4	4	3	3
B1336	2006-2010	Dong, H. *et al.* & 22479472	BSP4	Tibet	29.65	91.12	2	2	3	3	2	5	1	7	3	5	3	3	4	2	4	6	7	4	4	4	4	4	3	3
B1337	2006-2010	Dong, H. *et al.* & 22479472	BSP5	Tibet	29.65	91.12	2	2	3	3	2	5	1	7	3	5	2	3	4	2	4	6	4	2	4	6	4	4	3	3
B1338	2006-2010	Dong, H. *et al.* & 22479472	BSP5	Tibet	29.65	91.12	2	2	3	3	2	5	1	5	3	5	3	3	4	2	4	6	8	4	4	5	4	4	3	3
B1339	2006-2010	Dong, H. *et al.* & 22479472	BSP4	Tibet	29.65	91.12	2	2	3	3	1	5	1	7	3	6	3	3	4	2	4	5	7	2	4	4	4	4	3	3
B1340	2006-2010	Dong, H. *et al.* & 22479472	BSP5	Tibet	29.65	91.12	2	2	3	3	2	5	1	4	3	5	3	4	4	2	4	6	8	4	4	5	4	4	3	3
B1341	2006-2010	Dong, H. *et al.* & 22479472	BSP4	Tibet	29.65	91.12	2	2	3	3	1	5	1	7	3	5	3	3	4	2	4	5	7	2	4	4	4	4	3	3
B1342	2006-2010	Dong, H. *et al.* & 22479472	BSP4	Tibet	29.65	91.12	2	2	3	3	2	5	1	7	3	5	3	3	4	2	4	6	6	4	4	4	4	4	3	3
B1343	2006-2010	Dong, H. *et al.* & 22479472	BSP5	Tibet	29.65	91.12	2	2	3	3	2	5	1	7	3	5	3	3	4	2	4	5	6	2	4	5	4	4	3	3
B1344	2006-2010	Dong, H. *et al.* & 22479472	BSP4	Tibet	29.65	91.12	2	3	3	2	1	5	1	7	3	5	3	3	2	2	4	5	7	4	4	4	4	4	3	3
B1345	2006-2010	Dong, H. *et al.* & 22479472	BSP4	Tibet	29.65	91.12	2	2	3	3	2	5	1	7	3	5	3	3	4	2	4	6	7	4	4	4	4	4	3	3
B1346	2006-2010	Dong, H. *et al.* & 22479472	BSP4	Tibet	29.65	91.12	2	2	3	3	1	5	1	7	3	6	3	3	4	2	4	4	7	4	4	3	4	4	3	3
B1347	2006-2010	Dong, H. *et al.* & 22479472	BSP4	Tibet	29.65	91.12	2	2	3	3	1	5	1	7	3	6	3	4	4	2	4	4	7	4	4	4	4	4	3	3
B1348	2006-2010	Dong, H. *et al.* & 22479472	BSP5	Tibet	29.65	91.12	2	2	3	3	2	5	1	7	3	5	3	3	4	2	4	5	8	2	4	5	4	4	3	3
B1349	2006-2010	Dong, H. *et al.* & 22479472	BSP4	Tibet	29.65	91.12	2	2	3	3	2	5	1	7	3	2	3	3	4	2	4	6	7	4	4	4	4	4	3	3
B1350	2006-2010	Dong, H. *et al.* & 22479472	BSP5	Tibet	29.65	91.12	2	2	3	3	2	5	1	7	3	5	3	3	4	2	4	6	8	2	4	5	4	4	3	3
B1351	2006-2010	Dong, H. *et al.* & 22479472	BSP4	Tibet	29.65	91.12	2	2	3	3	2	5	1	7	3	5	3	3	4	2	4	6	7	4	4	4	4	4	3	3
B1352	2006-2010	Dong, H. *et al.* & 22479472	BSP4	Tibet	29.65	91.12	2	2	3	3	2	5	1	7	3	2	3	3	4	2	4	6	7	4	4	4	4	4	3	3
B1353	2006-2010	Dong, H. *et al.* & 22479472	BSP4	Tibet	29.65	91.12	2	2	3	3	1	5	1	7	3	6	3	4	4	2	4	5	7	1	4	4	4	4	3	3
B1354	2006-2010	Dong, H. *et al.* & 22479472	BSP1	Tibet	29.65	91.12	2	2	2	3	2	5	1	4	3	3	2	2	3	1	4	3	9	2	2	3	4	2	3	4
B1355	2006-2010	Dong, H. *et al.* & 22479472	BSPint	Tibet	29.65	91.12	2	2	3	3	2	5	1	7	2	5	3	3	4	2	4	6	ND	3	3	4	4	4	3	3
B1356	2006-2010	Dong, H. *et al.* & 22479472	BSP5	Tibet	29.65	91.12	2	1	3	2	2	5	1	7	3	5	3	3	4	2	4	3	8	2	3	5	4	4	3	3
B1357	2006-2010	Dong, H. *et al.* & 22479472	BSP4	Tibet	29.65	91.12	2	2	3	3	2	5	1	7	3	2	3	3	4	2	4	6	7	4	4	4	4	4	3	3
B1358	2006-2010	Dong, H. *et al.* & 22479472	BSP4	Tibet	29.65	91.12	2	2	3	3	2	5	1	7	3	5	3	3	4	2	4	6	7	4	4	4	4	4	3	3
B1359	2006-2010	Dong, H. *et al.* & 22479472	BSP5	Tibet	29.65	91.12	2	2	3	3	2	5	1	7	3	5	3	3	4	2	4	6	ND	2	4	5	4	6	3	3
B1360	2006-2010	Dong, H. *et al.* & 22479472	BSP4	Tibet	29.65	91.12	2	2	3	3	1	5	1	7	3	5	3	3	4	2	4	4	3	4	4	4	4	4	3	2
B1361	2006-2010	Dong, H. *et al.* & 22479472	BSP4	Tibet	29.65	91.12	2	2	3	3	1	5	1	7	3	5	3	3	4	2	4	4	7	4	4	4	4	4	3	2
B1362	2006-2010	Dong, H. *et al.* & 22479472	BSP5	Tibet	29.65	91.12	2	2	3	3	2	5	1	2	3	5	3	3	4	2	4	6	8	2	4	5	4	4	3	3
B1363	2006-2010	Dong, H. *et al.* & 22479472	BSP4	Tibet	29.65	91.12	2	2	3	3	1	5	1	7	3	5	3	3	4	2	4	4	4	4	4	4	4	4	3	2
B1364	2006-2010	Dong, H. *et al.* & 22479472	BSP4	Tibet	29.65	91.12	2	2	3	3	1	5	1	7	3	5	3	3	4	3	4	5	7	4	4	4	4	4	3	3
B1365	2006-2010	Dong, H. *et al.* & 22479472	BSP5	Tibet	29.65	91.12	2	2	3	3	2	5	1	7	3	5	3	3	4	2	4	5	8	2	4	5	4	4	3	3
B1366	2006-2010	Dong, H. *et al.* & 22479472	BSP4	Tibet	29.65	91.12	2	2	3	3	2	5	1	8	3	2	3	3	4	2	4	7	7	4	4	4	4	4	3	3
B1367	2006-2010	Dong, H. *et al.* & 22479472	BSP4	Tibet	29.65	91.12	2	2	3	3	1	5	1	7	3	5	3	3	4	2	4	4	7	4	4	4	4	4	3	2
B1368	2006-2010	Dong, H. *et al.* & 22479472	BSPint	Tibet	29.65	91.12	2	2	3	3	2	5	1	6	3	5	3	3	4	2	4	4	8	3	3	4	4	4	3	4
B1369	2006-2010	Dong, H. *et al.* & 22479472	BSP4	Tibet	29.65	91.12	2	2	3	3	1	5	1	7	3	6	3	3	4	2	4	4	7	4	4	4	4	4	3	3
B1370	2010-2011	Zhang, J. *et al.* &	BSP1	Xinjiang	43.79	87.63	2	3	5	4	2	5	2	6	3	6	2	3	4	2	4	2	7	4	3	4	4	4	3	4

		22713520																												
B1371	2010-2011	Zhang, J. *et al.* & 22713520	BSP1	Xinjiang	43.79	87.63	2	3	3	5	2	5	1	5	3	4	3	2	4	2	4	5	7	4	3	4	4	4	3	4
B1372	2010-2011	Zhang, J. *et al.* & 22713520	BSPint	Xinjiang	43.79	87.63	2	3	2	3	2	5	1	6	3	5	3	3	4	2	4	4	8	2	4	5	4	4	3	4
B1373	2010-2011	Zhang, J. *et al.* & 22713520	BSPint	Xinjiang	43.79	87.63	2	3	2	3	2	5	1	6	3	5	3	3	2	2	4	6	8	2	4	5	4	4	3	4
B1374	2010-2011	Zhang, J. *et al.* & 22713520	BSPint	Xinjiang	43.79	87.63	2	3	3	3	2	5	2	6	3	5	3	3	4	2	4	6	8	2	4	5	4	4	3	4
B1375	2010-2011	Zhang, J. *et al.* & 22713520	BSPint	Xinjiang	43.79	87.63	2	3	3	3	2	5	2	6	2	5	3	3	4	2	4	6	8	2	4	5	4	4	3	4
B1376	2010-2011	Zhang, J. *et al.* & 22713520	BSPint	Xinjiang	43.79	87.63	2	3	3	3	2	5	2	6	3	5	3	3	4	2	4	6	8	2	4	5	4	4	3	4
B1377	2010-2011	Zhang, J. *et al.* & 22713520	BSP5	Xinjiang	43.79	87.63	2	3	3	3	2	5	1	6	3	5	3	3	4	2	4	6	8	2	4	5	4	4	3	4
B1378	2010-2011	Zhang, J. *et al.* & 22713520	BSPint	Xinjiang	43.79	87.63	2	3	3	3	2	5	2	5	3	5	3	4	4	2	4	6	7	2	4	5	4	4	3	4
B1379	2010-2011	Zhang, J. *et al.* & 22713520	BSPint	Xinjiang	43.79	87.63	2	3	3	3	2	5	2	5	3	5	3	4	4	2	4	6	7	2	4	5	4	4	3	4
B1380	2010-2011	Zhang, J. *et al.* & 22713520	BSPint	Xinjiang	43.79	87.63	2	3	3	3	2	5	2	5	3	5	3	4	4	2	4	6	8	2	4	5	4	4	3	4
B1381	2010-2011	Zhang, J. *et al.* & 22713520	BSPint	Xinjiang	43.79	87.63	2	3	3	3	2	5	2	5	3	5	3	4	4	2	4	6	8	2	4	5	4	4	3	4
B1382	2010-2011	Zhang, J. *et al.* & 22713520	BSP5	Xinjiang	43.79	87.63	2	3	3	3	2	5	1	5	3	5	3	3	4	1	4	6	8	2	4	5	4	2	3	4
B1383	2010-2011	Zhang, J. *et al.* & 22713520	BSP5	Xinjiang	43.79	87.63	2	3	3	3	2	5	1	7	3	5	3	3	4	2	4	5	8	2	4	9	4	4	3	4
B1384	2010-2011	Zhang, J. *et al.* & 22713520	BSP5	Xinjiang	43.79	87.63	2	3	3	2	2	5	1	7	3	5	3	3	4	2	4	5	8	2	4	5	4	4	3	4
B1385	2010-2011	Zhang, J. *et al.* & 22713520	BSP5	Xinjiang	43.79	87.63	2	3	3	3	2	5	1	7	1	5	3	3	4	2	4	5	8	2	4	5	4	4	3	4
B1386	2010-2011	Zhang, J. *et al.* & 22713520	BSP5	Xinjiang	43.79	87.63	2	3	3	3	2	5	1	5	3	5	3	3	4	2	4	5	8	2	4	5	4	4	3	4
B1387	2010-2011	Zhang, J. *et al.* & 22713520	BSP5	Xinjiang	43.79	87.63	2	3	3	3	2	5	1	7	3	5	3	3	4	2	4	5	7	2	4	5	4	4	3	4
B1388	2010-2011	Zhang, J. *et al.* & 22713520	BSP5	Xinjiang	43.79	87.63	2	3	3	3	2	5	1	7	3	5	3	3	4	2	4	5	7	2	4	5	4	4	3	4
B1389	2010-2011	Zhang, J. *et al.* & 22713520	BSP5	Xinjiang	43.79	87.63	2	3	3	3	2	5	1	7	3	5	3	3	4	2	4	5	7	2	4	5	4	4	3	4
B1390	2010-2011	Zhang, J. *et al.* & 22713520	BSP5	Xinjiang	43.79	87.63	2	3	3	3	2	5	1	7	3	5	3	3	4	2	4	6	7	2	4	5	4	4	3	4
B1391	2010-2011	Zhang, J. *et al.* & 22713520	BSP5	Xinjiang	43.79	87.63	2	3	3	3	2	5	1	7	3	5	3	3	4	2	4	6	ND	2	4	5	4	4	3	4
B1392	2010-2011	Zhang, J. *et al.* & 22713520	BSP5	Xinjiang	43.79	87.63	2	3	3	3	2	5	1	7	3	5	3	3	4	2	4	3	8	2	4	5	4	4	3	4
B1393	2010-2011	Zhang, J. *et al.* & 22713520	BSP5	Xinjiang	43.79	87.63	2	3	3	3	2	5	1	7	3	5	3	3	4	2	4	4	6	2	4	5	4	4	3	4
B1394	2010-2011	Zhang, J. *et al.* & 22713520	BSP5	Xinjiang	43.79	87.63	2	3	3	3	2	5	1	7	3	5	3	2	4	2	4	4	8	2	4	5	4	4	3	4
B1395	2010-2011	Zhang, J. *et al.* & 22713520	BSP5	Xinjiang	43.79	87.63	2	3	3	3	2	5	1	7	3	5	3	3	3	2	4	5	7	2	4	6	4	4	3	4
B1396	2010-2011	Zhang, J. *et al.* & 22713520	BSP5	Xinjiang	43.79	87.63	2	3	3	3	2	5	1	7	3	5	3	3	3	2	4	5	7	2	4	6	4	4	3	4
B1397	2010-2011	Zhang, J. *et al.* & 22713520	BSP5	Xinjiang	43.79	87.63	2	3	3	3	2	5	1	7	3	5	3	3	3	2	4	5	8	2	4	5	4	4	3	4
B1398	2010-2011	Zhang, J. *et al.* & 22713520	BSP5	Xinjiang	43.79	87.63	2	3	3	3	2	5	1	6	3	5	3	3	4	2	4	6	7	2	4	5	4	4	3	5
B1399	2010-2011	Zhang, J. *et al.* & 22713520	BSPint	Xinjiang	43.79	87.63	2	3	3	3	2	5	2	6	3	5	3	3	4	2	4	6	7	2	3	5	4	4	3	5
B1400	2010-2011	Zhang, J. *et al.* & 22713520	BSPint	Xinjiang	43.79	87.63	2	3	3	3	2	5	1	6	3	5	3	3	4	2	4	6	8	2	3	4	4	4	3	5
B1401	2010-2011	Zhang, J. *et al.* & 22713520	BSPint	Xinjiang	43.79	87.63	2	3	3	3	2	5	1	5	3	5	3	3	4	2	4	6	ND	2	4	4	4	4	3	4
B1402	2010-2011	Zhang, J. *et al.* & 22713520	BSPint	Xinjiang	43.79	87.63	2	3	3	3	2	5	1	5	3	3	3	3	4	2	4	6	ND	2	4	4	4	4	3	4
B1403	2010-2011	Zhang, J. *et al.* & 22713520	BSPint	Xinjiang	43.79	87.63	2	3	3	3	2	5	1	5	3	5	3	3	4	2	4	7	ND	2	4	4	4	4	2	4
B1404	2010-2011	Zhang, J. *et al.* & 22713520	BSPint	Xinjiang	43.79	87.63	1	3	3	3	2	5	1	7	3	4	3	3	4	2	4	6	7	2	4	4	4	4	3	4
B1405	2010-2011	Zhang, J. *et al.* & 22713520	BSP4	Xinjiang	43.79	87.63	2	3	3	3	2	5	1	7	3	5	3	3	4	2	4	7	7	4	4	4	4	4	3	4
B1406	2010-2011	Zhang, J. *et al.* & 22713520	BSP4	Xinjiang	43.79	87.63	2	3	3	3	2	5	1	7	3	5	3	3	4	2	4	7	7	4	4	4	4	4	3	4
B1407	2010-2011	Zhang, J. *et al.* & 22713520	BSP4	Xinjiang	43.79	87.63	2	3	3	3	2	5	1	7	3	5	3	3	4	2	4	7	8	4	4	4	4	4	3	4
B1408	2010-2011	Zhang, J. *et al.* & 22713520	BSP4	Xinjiang	43.79	87.63	2	3	3	3	2	5	1	7	3	5	3	3	4	2	4	7	8	4	4	4	4	4	3	4
B1409	2010-2011	Zhang, J. *et al.* & 22713520	BSP4	Xinjiang	43.79	87.63	2	3	3	3	2	5	1	7	2	5	3	3	4	2	4	7	ND	4	4	4	4	4	3	4
B1410	2010-2011	Zhang, J. *et al.* & 22713520	BSPint	Xinjiang	43.79	87.63	2	3	3	3	2	5	2	7	3	5	3	3	4	2	4	7	7	4	4	4	4	4	3	4
B1411	2010-2011	Zhang, J. *et al.* &	BSPint	Xinjiang	43.79	87.63	2	3	3	3	2	5	2	8	3	4	3	3	4	2	4	7	7	4	4	4	4	4	3	4

		22713520																												
B1412	2010-2011	Zhang, J. *et al.* & 22713520	BSPint	Xinjiang	43.79	87.63	2	3	3	3	2	5	1	7	3	5	3	3	4	2	4	3	7	2	4	4	4	4	1	4
B1413	2010-2011	Zhang, J. *et al.* & 22713520	BSPint	Xinjiang	43.79	87.63	2	3	3	3	2	5	2	7	3	5	3	3	4	2	4	4	7	2	4	4	4	4	3	4
B1414	2010-2011	Zhang, J. *et al.* & 22713520	BSP4	Xinjiang	43.79	87.63	2	3	3	3	2	5	1	8	3	5	3	3	2	2	4	5	7	4	4	4	4	4	3	4
B1415	2010-2011	Zhang, J. *et al.* & 22713520	BSPint	Xinjiang	43.79	87.63	2	3	3	3	2	5	1	6	3	5	3	3	4	2	4	4	8	3	4	4	4	4	3	4
B1416	2010-2011	Zhang, J. *et al.* & 22713520	BSP2	Xinjiang	43.79	87.63	2	3	3	3	2	5	2	7	3	5	3	3	4	2	4	6	7	3	3	4	4	4	3	4
B1417	2010-2011	Zhang, J. *et al.* & 22713520	BSPint	Xinjiang	43.79	87.63	2	3	3	3	2	4	1	7	4	5	3	3	4	2	4	6	8	2	4	3	4	4	3	4
B1418	2010-2011	Zhang, J. *et al.* & 22713520	BSPint	Xinjiang	43.79	87.63	2	3	3	4	2	5	1	7	3	5	3	2	4	2	4	4	9	3	4	4	4	2	3	4
B1419	2002-2005	Jiao, W. *et al.* & 18199785	BSP4	Beijing	39.90	116.41	2	2	1	3	2	5	1	7	3	5	3	3	4	2	4	6	8	4	2	4	4	4	3	3
B1420	2002-2005	Jiao, W. *et al.* & 18199785	BSP4	Beijing	39.90	116.41	2	2	1	3	2	5	1	9	3	5	3	3	4	2	4	6	8	4	3	4	4	4	3	3
B1421	2002-2005	Jiao, W. *et al.* & 18199785	BSP4	Beijing	39.90	116.41	2	2	1	3	2	5	1	7	3	5	3	4	4	2	4	4	8	4	4	4	4	4	3	3
B1422	2002-2005	Jiao, W. *et al.* & 18199785	BSP4	Beijing	39.90	116.41	2	2	1	3	2	5	1	5	3	5	3	3	4	2	4	7	5	4	4	3	4	4	3	6
B1423	2002-2005	Jiao, W. *et al.* & 18199785	BSP4	Beijing	39.90	116.41	2	2	3	3	2	5	1	7	3	5	3	3	4	2	6	6	8	4	4	4	4	4	4	3
B1424	2002-2005	Jiao, W. *et al.* & 18199785	BSP5	Beijing	39.90	116.41	2	2	3	3	2	5	1	7	3	5	3	3	2	2	4	5	8	2	4	5	4	4	3	3
B1425	2002-2005	Jiao, W. *et al.* & 18199785	BSP5	Beijing	39.90	116.41	2	2	3	3	2	5	1	6	3	5	2	3	4	2	4	6	6	2	4	5	4	4	3	3
B1426	2002-2005	Jiao, W. *et al.* & 18199785	BSP5	Beijing	39.90	116.41	2	2	3	3	2	5	1	9	3	5	3	3	4	2	4	4	8	2	5	5	4	4	3	3
B1427	2002-2005	Jiao, W. *et al.* & 18199785	BSP5	Beijing	39.90	116.41	2	2	3	3	2	5	1	7	3	5	3	3	4	2	4	5	8	2	4	5	4	4	3	3
B1428	2002-2005	Jiao, W. *et al.* & 18199785	BSP5	Beijing	39.90	116.41	2	2	3	3	2	5	1	7	3	5	3	3	3	2	4	5	8	2	4	6	4	4	3	3
B1429	2002-2005	Jiao, W. *et al.* & 18199785	BSP5	Beijing	39.90	116.41	2	2	3	3	2	5	1	7	3	4	3	3	4	2	4	5	8	2	4	5	4	4	3	3
B1430	2002-2005	Jiao, W. *et al.* & 18199785	BSP5	Beijing	39.90	116.41	2	2	3	3	2	5	1	5	3	5	3	3	4	2	4	5	8	2	4	5	4	4	3	3
B1431	2002-2005	Jiao, W. *et al.* & 18199785	BSP5	Beijing	39.90	116.41	2	2	3	3	2	5	1	7	3	5	3	3	4	2	4	5	8	2	4	5	4	4	3	3
B1432	2002-2005	Jiao, W. *et al.* & 18199785	BSP5	Beijing	39.90	116.41	2	2	2	3	2	5	1	7	3	5	3	3	4	2	4	6	7	2	4	5	4	4	3	3
B1433	2002-2005	Jiao, W. *et al.* & 18199785	BSP5	Beijing	39.90	116.41	2	2	2	3	2	5	1	7	3	5	3	3	4	2	4	6	8	2	4	5	4	4	3	3
B1434	2002-2005	Jiao, W. *et al.* & 18199785	BSP5	Beijing	39.90	116.41	2	2	3	3	2	5	1	7	3	5	3	3	4	2	4	5	8	2	4	5	4	4	3	3
B1435	2002-2005	Jiao, W. *et al.* & 18199785	BSP5	Beijing	39.90	116.41	2	2	3	3	2	5	1	7	3	5	3	3	4	2	4	5	8	2	4	1	4	4	3	3
B1436	2002-2005	Jiao, W. *et al.* & 18199785	BSP5	Beijing	39.90	116.41	2	2	3	3	2	5	1	7	3	5	3	4	3	2	6	5	6	2	4	1	4	2	3	3
B1437	2002-2005	Jiao, W. *et al.* & 18199785	BSP5	Beijing	39.90	116.41	2	2	3	3	2	5	1	7	3	5	3	3	4	2	4	5	8	2	4	4	4	4	3	3
B1438	2002-2005	Jiao, W. *et al.* & 18199785	BSP5	Beijing	39.90	116.41	2	2	3	3	2	5	1	7	3	5	3	3	4	2	4	6	7	2	4	5	4	4	3	3
B1439	2002-2005	Jiao, W. *et al.* & 18199785	BSP5	Beijing	39.90	116.41	2	2	3	3	2	5	1	7	3	5	3	1	4	2	4	6	8	2	4	5	4	4	3	3
B1440	2002-2005	Jiao, W. *et al.* & 18199785	BSP5	Beijing	39.90	116.41	2	2	3	3	2	5	1	7	3	5	3	3	4	2	4	6	7	2	4	5	2	4	3	3
B1441	2002-2005	Jiao, W. *et al.* & 18199785	BSP5	Beijing	39.90	116.41	2	2	3	3	2	5	1	8	3	5	3	3	4	2	4	6	8	2	4	5	4	4	3	3
B1442	2002-2005	Jiao, W. *et al.* & 18199785	BSP5	Beijing	39.90	116.41	2	2	3	3	2	5	1	8	3	5	3	3	4	2	4	6	8	2	4	5	4	4	3	3
B1443	2002-2005	Jiao, W. *et al.* & 18199785	BSP5	Beijing	39.90	116.41	2	2	3	3	2	5	1	7	3	5	3	3	4	2	4	6	8	2	3	5	4	4	3	1
B1444	2002-2005	Jiao, W. *et al.* & 18199785	BSP5	Beijing	39.90	116.41	2	2	3	3	2	6	1	7	3	5	3	3	4	1	4	6	8	2	4	6	4	4	3	3
B1445	2002-2005	Jiao, W. *et al.* & 18199785	BSP4	Beijing	39.90	116.41	2	2	3	3	2	5	1	7	3	5	3	4	4	2	2	6	7	2	3	4	4	4	3	3
B1446	2002-2005	Jiao, W. *et al.* & 18199785	BSP5	Beijing	39.90	116.41	2	2	3	3	2	5	1	7	3	5	3	3	4	2	4	6	8	2	3	5	4	4	3	1
B1447	2002-2005	Jiao, W. *et al.* & 18199785	BSP5	Beijing	39.90	116.41	2	2	3	3	2	5	1	7	3	6	3	3	4	2	4	6	7	2	4	5	4	4	3	3
B1448	2002-2005	Jiao, W. *et al.* & 18199785	BSPint	Beijing	39.90	116.41	2	2	3	3	2	5	1	7	3	5	3	3	4	2	4	7	8	2	4	4	4	4	3	3
B1449	2002-2005	Jiao, W. *et al.* & 18199785	BSP5	Beijing	39.90	116.41	2	2	3	3	2	5	1	7	3	5	3	3	4	2	4	6	8	2	4	5	4	4	3	3
B1450	2002-2005	Jiao, W. *et al.* & 18199785	BSP5	Beijing	39.90	116.41	2	2	3	3	2	5	1	7	3	5	3	3	4	2	4	6	8	2	4	5	2	4	3	3
B1451	2002-2005	Jiao, W. *et al.* & 18199785	BSP5	Beijing	39.90	116.41	2	2	3	3	2	5	1	7	3	5	3	3	4	2	4	6	7	2	4	5	4	4	3	3
B1452	2002-2005	Jiao, W. *et al.* &	BSP5	Beijing	39.90	116.41	2	2	3	4	2	5	1	9	3	5	3	3	3	2	4	5	8	2	2	5	4	5	3	3

		18199785																												
B1453	2002-2005	Jiao, W. *et al.* & 18199785	BSP5	Beijing	39.90	116.41	2	2	3	3	2	5	1	7	3	5	3	3	4	2	4	6	8	2	5	5	4	4	3	3
B1454	2002-2005	Jiao, W. *et al.* & 18199785	BSP4	Beijing	39.90	116.41	2	2	3	3	2	5	1	7	3	5	3	2	4	2	4	5	8	4	4	4	4	4	3	3
B1455	2002-2005	Jiao, W. *et al.* & 18199785	BSP5	Beijing	39.90	116.41	2	2	3	3	2	5	1	6	3	6	3	3	4	2	4	4	8	2	4	5	4	4	3	3
B1456	2002-2005	Jiao, W. *et al.* & 18199785	BSP5	Beijing	39.90	116.41	2	2	3	3	2	5	1	7	3	5	3	3	4	2	4	4	8	2	4	3	4	4	3	3
B1457	2002-2005	Jiao, W. *et al.* & 18199785	BSP5	Beijing	39.90	116.41	2	2	3	3	2	5	1	7	3	5	3	3	3	2	4	5	7	2	4	5	4	4	3	3
B1458	2002-2005	Jiao, W. *et al.* & 18199785	BSP5	Beijing	39.90	116.41	2	2	3	3	2	5	1	7	3	5	3	3	4	2	4	6	7	2	4	5	4	4	3	3
B1459	2002-2005	Jiao, W. *et al.* & 18199785	BSP5	Beijing	39.90	116.41	2	2	3	2	2	5	1	7	3	5	3	3	4	2	4	6	8	2	4	5	3	4	3	3
B1460	2002-2005	Jiao, W. *et al.* & 18199785	BSP5	Beijing	39.90	116.41	2	2	3	3	1	5	1	7	3	5	3	3	3	2	4	3	3	2	4	5	4	4	3	3
B1461	2002-2005	Jiao, W. *et al.* & 18199785	BSP5	Beijing	39.90	116.41	2	2	3	3	2	5	1	7	3	5	3	3	4	2	4	4	8	2	4	5	3	4	3	3
B1462	2002-2005	Jiao, W. *et al.* & 18199785	BSP5	Beijing	39.90	116.41	2	0	3	3	2	5	1	7	3	5	3	3	4	2	4	5	8	2	4	5	4	4	3	3
B1463	2002-2005	Jiao, W. *et al.* & 18199785	BSP5	Beijing	39.90	116.41	2	2	3	3	2	5	1	7	3	5	3	3	4	2	4	4	ND	2	4	5	4	4	3	3
B1464	2002-2005	Jiao, W. *et al.* & 18199785	BSP5	Beijing	39.90	116.41	2	2	3	3	2	5	1	6	3	5	3	3	3	2	4	5	8	2	4	5	4	4	3	3
B1465	2002-2005	Jiao, W. *et al.* & 18199785	BSP5	Beijing	39.90	116.41	2	0	3	3	2	5	1	7	3	4	3	3	4	2	4	5	8	1	4	5	4	4	3	3
B1466	2002-2005	Jiao, W. *et al.* & 18199785	BSP5	Beijing	39.90	116.41	2	2	3	3	2	5	1	7	3	5	2	3	4	2	4	6	8	2	4	5	4	4	3	2
B1467	2002-2005	Jiao, W. *et al.* & 18199785	BSP5	Beijing	39.90	116.41	2	3	3	3	2	5	1	7	3	6	4	3	4	2	4	6	8	2	4	5	4	4	3	3
B1468	2002-2005	Jiao, W. *et al.* & 18199785	BSP5	Beijing	39.90	116.41	2	2	3	3	2	5	1	3	3	5	3	3	4	2	4	6	7	2	4	5	4	4	3	3
B1469	2002-2005	Jiao, W. *et al.* & 18199785	BSP5	Beijing	39.90	116.41	2	1	3	3	2	5	1	7	2	5	2	3	4	2	4	6	8	2	4	4	4	4	3	3
B1470	2002-2005	Jiao, W. *et al.* & 18199785	BSP5	Beijing	39.90	116.41	2	2	3	3	2	5	1	7	3	5	3	3	4	2	4	6	8	2	4	5	4	4	3	3
B1471	2002-2005	Jiao, W. *et al.* & 18199785	BSP5	Beijing	39.90	116.41	2	2	3	3	2	5	1	7	3	5	3	3	4	2	4	6	7	2	4	5	2	4	3	4
B1472	2002-2005	Jiao, W. *et al.* & 18199785	BSP5	Beijing	39.90	116.41	2	2	3	3	2	5	1	7	3	5	3	3	4	2	4	5	8	2	4	5	4	4	3	3
B1473	2002-2005	Jiao, W. *et al.* & 18199785	BSP5	Beijing	39.90	116.41	2	2	3	3	2	5	1	7	3	5	3	3	4	2	4	5	8	2	4	5	4	4	3	3
B1474	2002-2005	Jiao, W. *et al.* & 18199785	BSP5	Beijing	39.90	116.41	2	2	3	3	2	5	1	7	3	5	3	1	4	2	4	4	8	2	4	5	4	4	3	3
B1475	2002-2005	Jiao, W. *et al.* & 18199785	BSP5	Beijing	39.90	116.41	2	2	3	3	2	5	1	7	3	5	3	3	4	2	4	6	8	2	4	5	4	4	3	3
B1476	2002-2005	Jiao, W. *et al.* & 18199785	BSP5	Beijing	39.90	116.41	2	2	3	3	2	5	1	5	3	5	3	3	4	2	3	6	9	2	4	6	4	4	3	3
B1477	2002-2005	Jiao, W. *et al.* & 18199785	BSP4	Beijing	39.90	116.41	2	2	3	3	2	5	1	6	3	5	4	4	4	2	4	7	9	2	4	4	4	3	3	3
B1478	2002-2005	Jiao, W. *et al.* & 18199785	BSP4	Beijing	39.90	116.41	2	1	3	3	2	5	1	7	3	5	3	3	4	2	4	6	8	4	3	4	4	4	3	3
B1479	2002-2005	Jiao, W. *et al.* & 18199785	BSP5	Beijing	39.90	116.41	2	2	3	3	2	5	1	6	3	5	3	3	4	2	4	6	9	2	4	4	4	4	3	3
B1480	2002-2005	Jiao, W. *et al.* & 18199785	BSP5	Beijing	39.90	116.41	2	2	3	3	2	5	1	6	3	5	3	3	4	2	4	6	9	2	4	4	4	4	3	3
B1481	2002-2005	Jiao, W. *et al.* & 18199785	BSP4	Beijing	39.90	116.41	2	2	3	2	2	5	1	7	3	5	3	3	3	2	4	7	8	4	2	4	4	4	3	3
B1482	2002-2005	Jiao, W. *et al.* & 18199785	BSP4	Beijing	39.90	116.41	2	2	3	3	2	5	1	7	3	5	3	3	4	2	4	7	ND	4	4	4	4	4	3	3
B1483	2002-2005	Jiao, W. *et al.* & 18199785	BSP4	Beijing	39.90	116.41	2	2	3	3	2	5	1	7	3	5	3	3	3	2	4	6	4	4	4	4	4	4	3	3
B1484	2002-2005	Jiao, W. *et al.* & 18199785	BSP4	Beijing	39.90	116.41	2	2	3	3	2	5	1	7	3	5	3	3	4	2	4	6	8	1	4	4	4	4	3	3
B1485	2002-2005	Jiao, W. *et al.* & 18199785	BSP4	Beijing	39.90	116.41	2	2	3	3	2	5	1	7	3	4	3	3	4	2	4	3	8	4	4	4	4	4	3	3
B1486	2002-2005	Jiao, W. *et al.* & 18199785	BSP4	Beijing	39.90	116.41	2	2	3	3	2	5	1	7	3	4	3	3	3	2	4	3	8	4	4	4	4	4	3	3
B1487	2002-2005	Jiao, W. *et al.* & 18199785	BSP4	Beijing	39.90	116.41	2	2	3	3	2	5	1	7	3	5	3	2	4	2	4	5	6	3	4	4	4	4	3	3
B1488	2002-2005	Jiao, W. *et al.* & 18199785	BSP4	Beijing	39.90	116.41	2	2	3	3	2	5	1	7	3	5	3	3	4	2	4	3	7	4	2	6	4	4	3	4
B1489	2002-2005	Jiao, W. *et al.* & 18199785	BSPint	Beijing	39.90	116.41	2	2	3	3	2	5	1	7	3	5	3	3	4	2	4	6	9	3	3	4	4	4	3	4
B1490	2002-2005	Jiao, W. *et al.* & 18199785	BSPint	Beijing	39.90	116.41	2	2	3	3	2	5	1	7	3	5	3	3	4	2	4	5	8	5	3	4	4	4	3	3

ND: not determined.

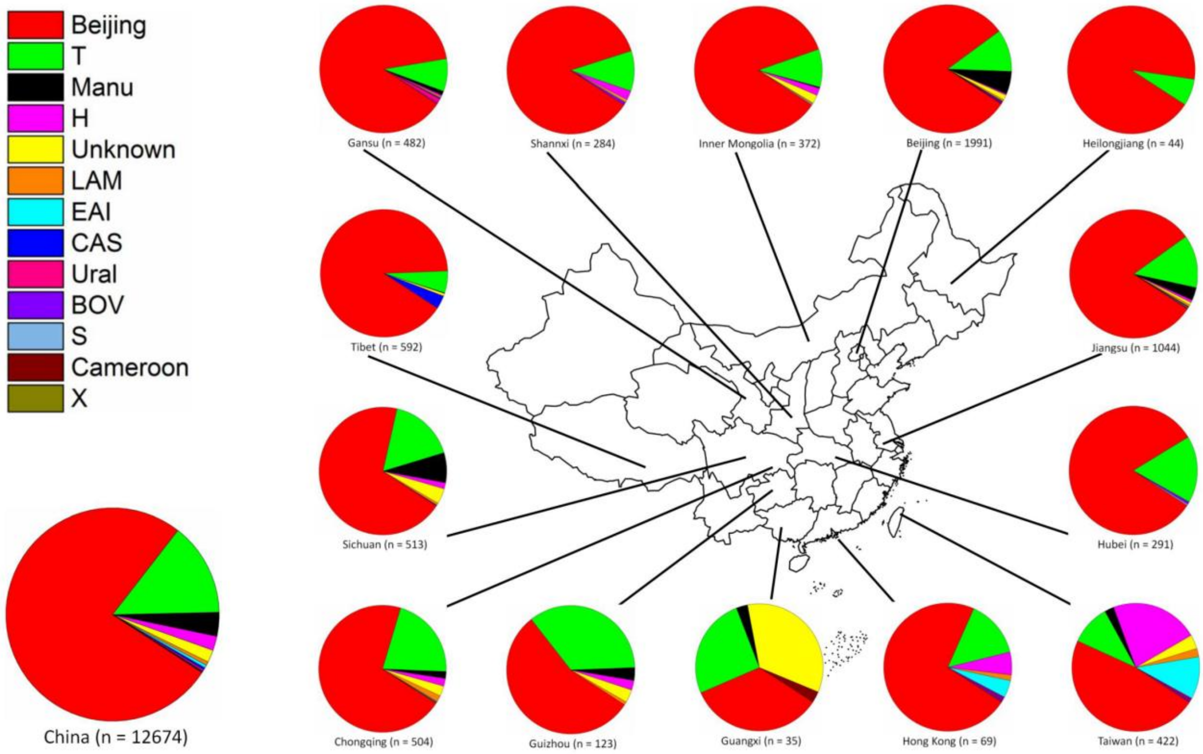

Figure S1. Geographical distribution of *M. tuberculosis* Complex lineages in mainland China and Taiwan, classified by spoligotyping. The map was created specifically for this investigation using the R software (R version 3.2.3, https://www.r-project.org/).

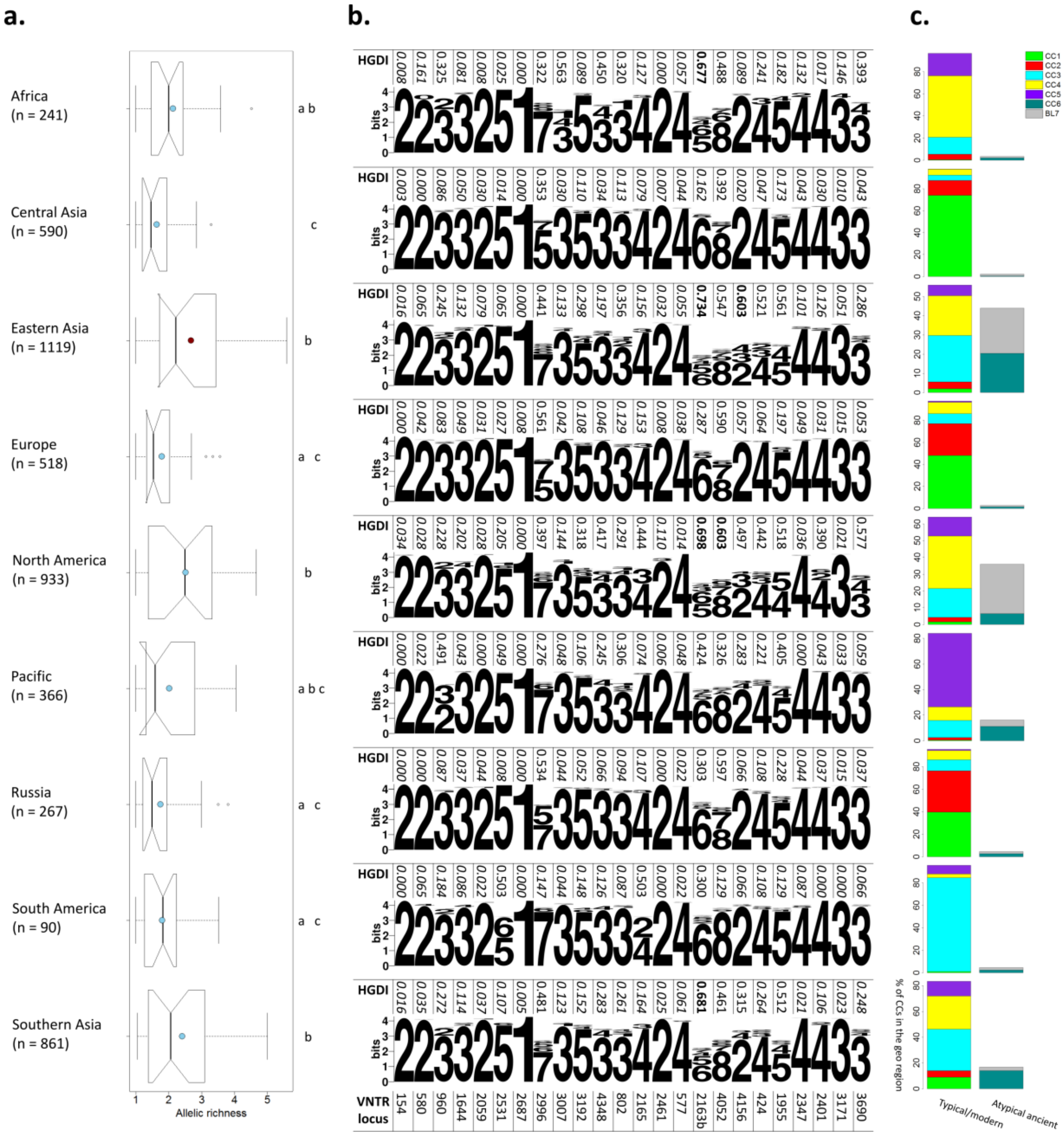

Figure S2. The mean allelic richness of 24-loci MIRU-VNTRs and WebLogo representation to visualize main patterns observed in different geographic regions worldwide and distribution of Beijing clonal complexes. (**a**) Allelic richness in each geographic region. Dots correspond to the mean allelic richness, notched boxes correspond to median values ± quartile, bars correspond to the minimum/maximum values. Comparison tests as well as P values were estimated based on mean values by t-test (groups that have the same letter do not have significantly different means). (**b**) Logo of allele copy number of 24-loci MIRU-VNTRs in each geographic region. The allelic diversity of the loci was classified as highly discriminant (HGDI > 0.6, bold in the table), moderately discriminant (0.3 ⩽ HGDI ⩽ 0.6, normal) and poorly discriminant (HGDI < 0.3, italic). (**c**) Proportions of different CCs (CC1–CC5 comprised typical/modern Beijing strains; CC6 and BL7 comprised atypical ancestral Beijing variants) among the 9 geographic regions. Note that CC groupings and associated data are from Merker *et al.*[19].

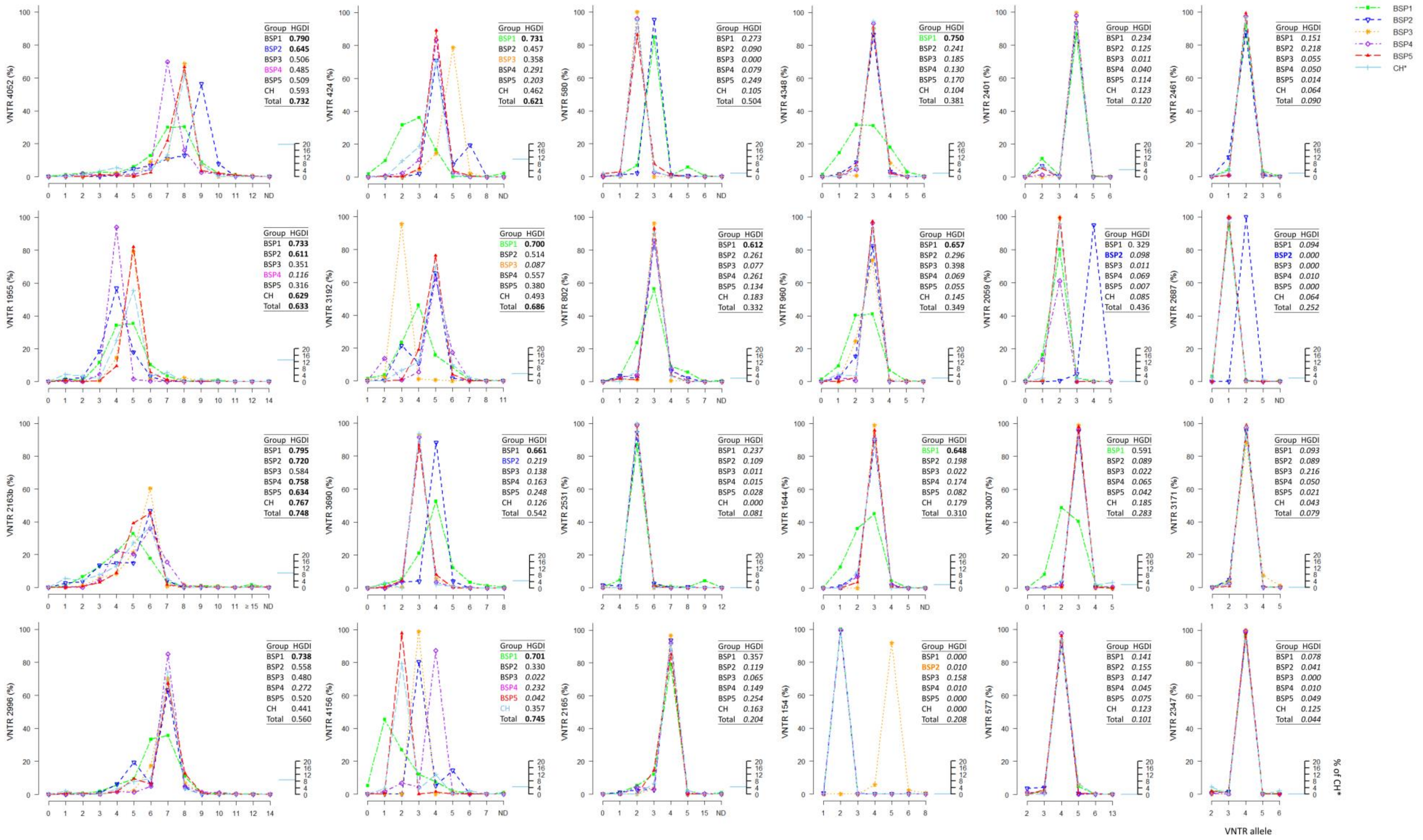

Figure S3. The distribution of allele copy numbers for individual MIRU-VNTR loci among various BSP groupings and reported cases of clonal heterogeneity (n=92) in our dataset from China. The figure shows percentages of Beijing strains belonging to a specific group (y axis) with a given allele copy number (x axis). The dataset analyzed contained n=1402 isolates classified as BSP1 to BSP5 (since it excluded 88/1490 BSPint isolates in undefined or intermediate position), as well as n=92 entries for clonal heterogeneity (CH) observed for 46 isolates. Note that a right y axis represents the detection rate for clonal heterogeneity, i.e., the percentage of total CH isolates that showed double alleles for a given locus. The legend within each inbox shows the Hunter-Gaston discriminatory index (HGDI) for each group. The allelic diversity of the loci was classified as highly discriminant (HGDI > 0.6, shown as “bold” in the inbox), moderately discriminant (0.3 ⩽ HGDI ⩽ 0.6, shown as “normal” in the inbox) and poorly discriminant (HGDI < 0.3, shown as “italic” in the inbox). Lastly, a color-marked group in inbox underlines statistically significant differences with others regarding the proportion of strains with contrasted number of repeats (P < 0.05). Furthermore, if written in bold, a color-marked group indicates its specificity regrading a given repeat number.

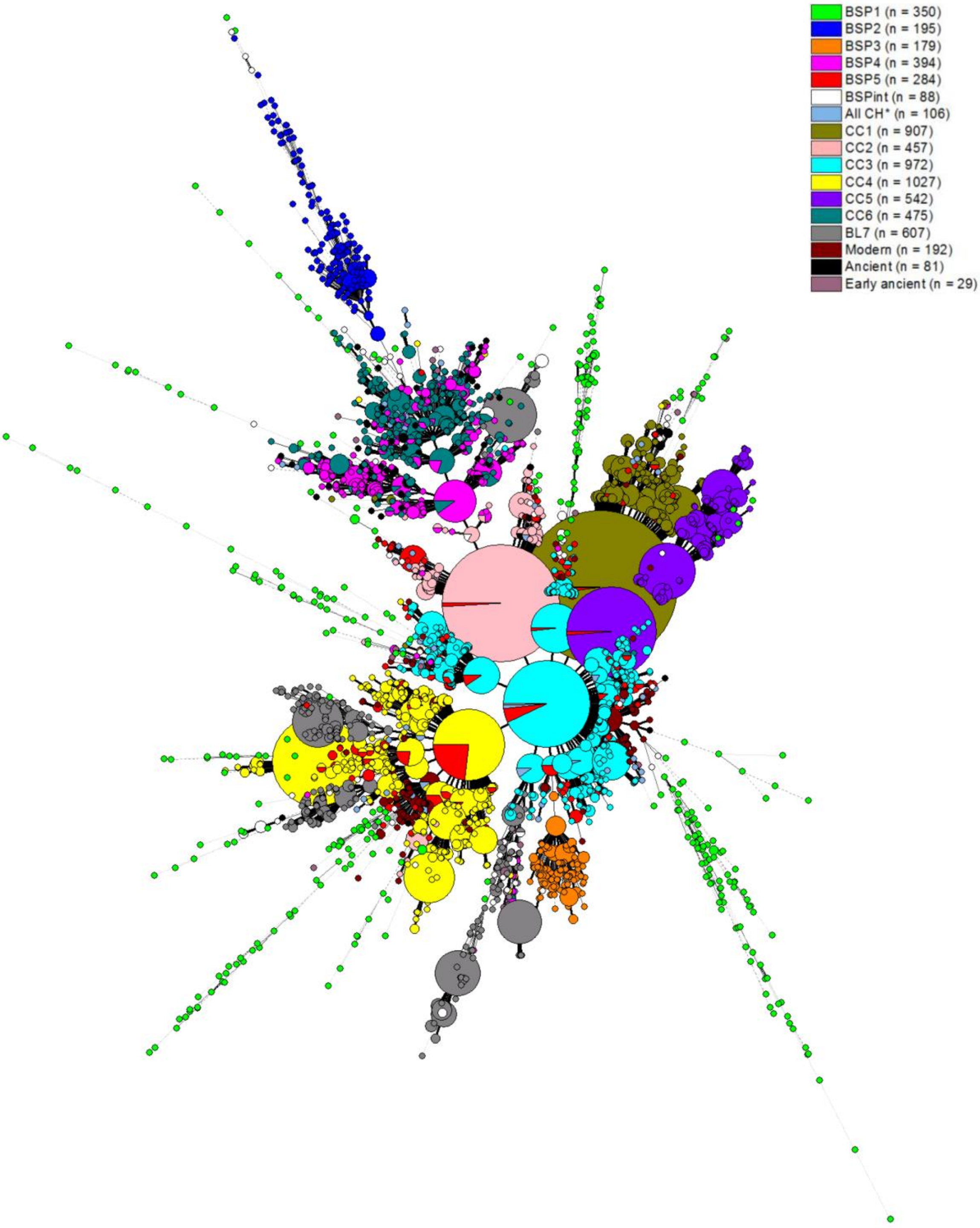

Figure S4. A minimum spanning tree showing evolutionary relationships of different BSP groupings in the present study and Beijing clonal complexes (CCs) defined in a worldwide study, and cases of clonal heterogeneity from China. The MST essentially shows a tree with the same dataset as in Figure 3, i.e. 6779 strains, supplemented with 106 entries corresponding to 53 strains of clonal heterogeneity. Among the latter, 46/53 strains were included in our dataset while 7/53 strains were from a recent study by Yin *et al.*[20], and described as Modern (10 entries from 5 strains), Ancient (2 entries from 1 strain), and Early ancient (2 entries from 1 strain).
